# Bioinformatics and Immunohistochemistry Reveal the Diagnostic and Mechanistic Role of the Cuproptosis‐Related Genes SMOC2/THY1 in Liver Fibrosis

**DOI:** 10.1111/jcmm.71144

**Published:** 2026-04-17

**Authors:** Ranyan Gao, Hongliang Chen, Jiaxin Wang, Lingyi Xu, Fengchun Li, Xinyu Jiang, Xinyu Geng, Ning Li, Ram Prasad Chaulagain, Babalola Deborah Oluwaseun, Wanwei Li, Shizhu Jin

**Affiliations:** ^1^ Department of Gastroenterology and Hepatology, The Second Affiliated Hospital Harbin Medical University Harbin Heilongjiang Province China; ^2^ Digestive Endoscopic Center, The Second Affiliated Hospital Harbin Medical University Harbin Heilongjiang Province China

**Keywords:** cuproptosis, immunohistochemistry, liver fibrosis, molecular docking, molecular dynamics, single‐cell RNA‐seq

## Abstract

Cuproptosis, a newly identified form of programmed cell death, inhibits liver fibrosis in hepatic stellate cells. However, how cuproptosis influences liver fibrosis in other cells remains to be determined. We aimed to develop a diagnostic model for cuproptosis and to further investigate its role in liver fibrosis. We obtained datasets of patients with metabolic dysfunction‐associated fatty liver disease from the Gene Expression Omnibus. A diagnostic model was established using WGCNA and machine learning, and the model demonstrated a satisfied accuracy in the training and validation sets. Single‐cell RNA (scRNA) confirmed that cuproptosis is highly likely to occur in hepatocytes and likely promotes liver fibrosis through communication with macrophages and dendritic cells. Immunohistochemistry analyses revealed that THY1 localized to fibroblast membranes and SMOC2 localized to hepatocytes. Molecular docking and molecular dynamics were used to identify the most suitable therapeutic drugs and suggested that breviscapine may target THY1, which may have a therapeutic effect on liver fibrosis. These findings establish a diagnostic model based on cuproptosis‐related genes for liver fibrosis, identify THY1 and SMOC2 as key diagnostic biomarkers, and propose a model in which the expression of these markers in hepatocyte are associated with fibrosis progression, potentially through immune‐mediated mechanisms.

**Abbreviations**
AUC: area under the curve
DCA: decision curve analysis
DCs: dendritic cells
DEGs: differentially expressed genes
ECM: excessive extracellular matrix
FCGR: Fc gamma receptor
GEO: Gene Expression Omnibus
GMM: gaussian mixture model
GO: gene ontology
GS: gene significance
GSEA: gene set enrichment analysis
GSVA: gene set variation analysis
HC: healthy control
HE: haematoxylin and eosin
HSCs: hepatic stellate cells
IHC: immunohistochemistry
KEGG: Kyoto Encyclopedia of Genes and Genomes
LASSO: least absolute shrinkage and selection operator
LC: liver cirrhosis
LSECs: liver sinusoidal endothelial cells
MASH: metabolic dysfunction‐associated steatohepatitis
MASLD: metabolic dysfunction‐associated steatotic liver disease
MEs: module eigengenes
MM: module membership
mRNA: messenger RNA
NAS: NAFLD Activity Score
NK: natural killer cell
PCA: principal component analysis
SAF: Steatosis‐Activity‐Fibrosis
SiRNA: small interfering RNA
SMOC2: SPARC‐related modular calcium binding protein 2
SPARC: secreted protein acidic and rich in cysteine
SVM: support vector machine
TCA: tricarboxylic acid
THY1: Thy‐1 cell surface antigen
UMAP: uniform manifold approximation and projection
WGCNA: weighted gene co‐expression network analysis

## 1 Introduction

Liver fibrosis, characterized by excessive extracellular matrix (ECM) deposition and collagen accumulation in the space of Disse, results from chronic liver injury triggered by viral infections, alcohol abuse or metabolic disorders. Notably, metabolic dysfunction‐associated steatohepatitis (MASH) is rapidly becoming the leading cause of chronic liver disease worldwide [1]. Inflammation exacerbates ECM deposition, potentially leading to progression to cirrhosis if left untreated. Compared with invasive diagnosis, noninvasive methods dominate safety, reproducibility and dynamic monitoring. A combination usage of elastography and serum scores is helpful for diagnostic accuracy beyond individual modalities. Lifestyle changes and pharmacotherapy are benefit for patients with F2 stage liver fibrosis, whereas patients with F3 stage liver fibrosis have a significantly increase of mortality, which highlight the critical need of early detection and targeted therapeutic strategies presently [2, 3]. Immune microenvironment was regarded as a key role in fibrotic development, however the exact pathogenesis of which remains unclear [4].

Cuproptosis is a novel form of programmed cell death characterized by abnormally high intracellular copper levels and executed primarily in the TCA cycle [5]. Our investigation revealed a significant correlation between negative cuproptosis regulators and immune cell infiltration in liver fibrosis. By performing transcriptome‐level analyses, we developed a diagnostic model comprising six plasma‐detectable genes. Cuproptosis‐driven fibrosis progression was mechanistically linked to Thy‐1 cell surface antigen (THY1)/secreted protein acidic and rich in cysteine (SPARC)‐related modular calcium binding protein 2 (SMOC2) activation and immune‐cell crosstalk, highlighting these genes as potential therapeutic targets.

## 2 Materials and Methods

**FIGURE 1:**
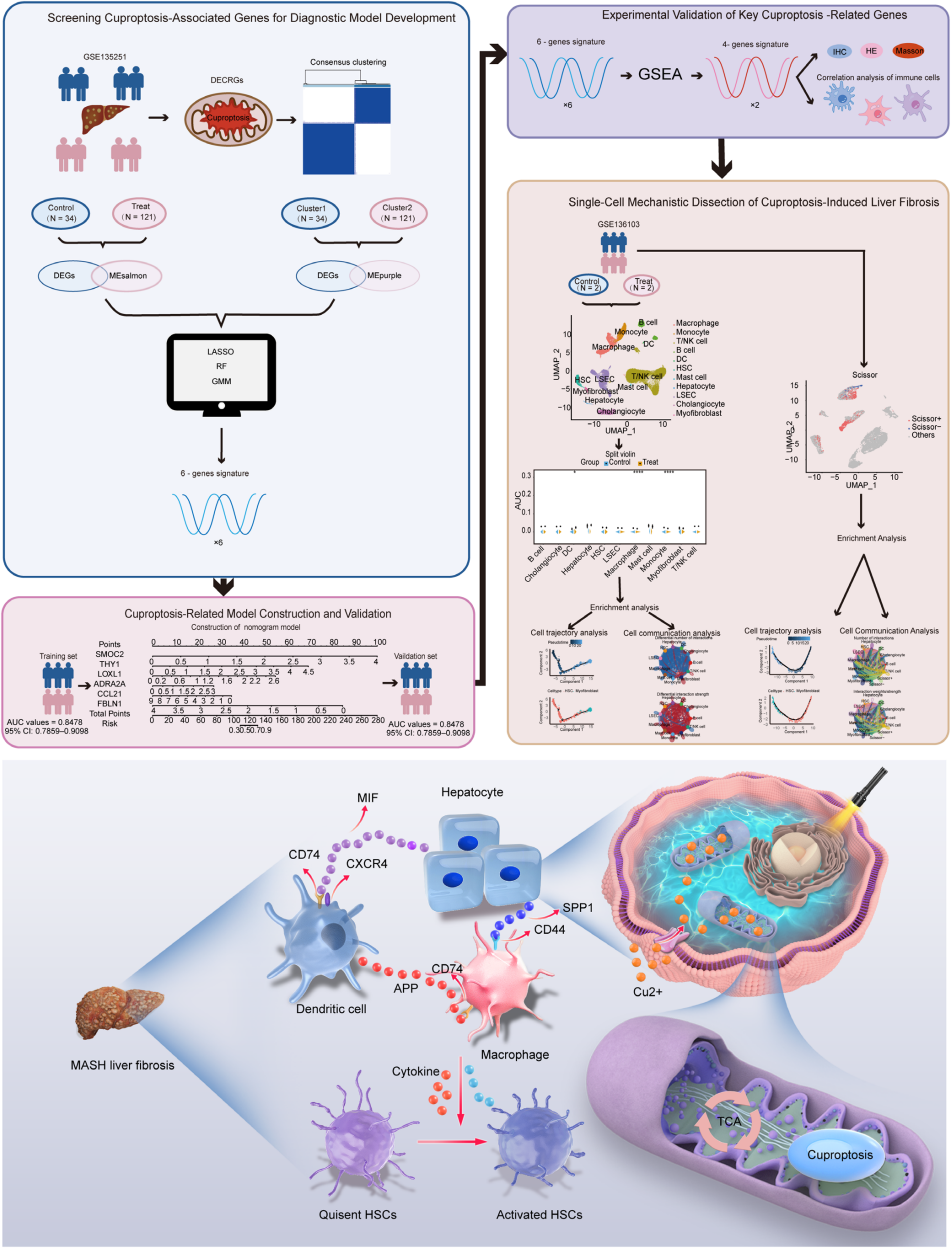
Flowchart and mechanism.

The flowchart of this study is presented in Figure 1.

### 2.1 Data Collection and Preprocessing

#### 2.1.1 Bulk RNA Datasets

Four bulk RNA‐seq datasets were obtained from the Gene Expression Omnibus (GEO) (Table S1) and were normalized to TPM/FPKM. MASH samples of the F0–F1 stages were identified as the ‘control’ group, and MASH samples of the F2–F4 stages were identified as the ‘treat’ group. GSE135251 was used as a training set, including 34 MASH samples of F0‐F1 stages and 121 MASH samples of F3–F4 stages. The GSE162694 was used as a validation set and included 47 MASH samples of F0–F1 stages and 65 MASH samples of F3–F4 stages. The GSE84044 (43 S0 samples, 20 S1 cases, 33 S2 cases, 18 S3 cases and 10 S4 cases) and GSE6764 (10 healthy control [HC] and 10 liver cirrhosis [LC] patients) datasets were used to verify the expression levels of SMOC and THY1.

#### 2.1.2 Single‐Cell RNA Datasets

The single‐cell RNA (scRNA)‐seq dataset GSE136103 (5 HC samples and 5 LC samples) was obtained from GEO (Table S1), and the cells were filtered for 30–6000 genes, < 30% mitochondria, and > 1% ribosomal genes. Harmony batch correction, principal component analysis (PCA) (top 20), clustering (0.8) and uniform manifold approximation and projection (UMAP) visualization were applied. Previous experience in cell type recognition and ACT website annotation were used for marker identification. Sixteen cuproptosis‐related genes were included in this study [5].

### 2.2 Bulk Data Analysis

#### 2.2.1 Consensus Clustering Analysis

To identify distinct subgroups of cuproptosis‐related genes, we employed the ConsensusClusterPlus algorithm, utilizing the Pearson correlation distance as the metric for measuring gene expression similarity. The optimal number of clusters (K) was determined by the consensus heatmaps and delta diagrams. Heatmaps were used to present the stability of cluster assignments across multiple iterations. Delta diagrams were used to identify the point of stabilization via quantify the change in area under the cumulative distribution function curves. To explore the correlations between cuproptosis‐related genes and fibrotic progression, Sankey diagrams were used to illustrate the relationships between fibrotic stages and identified subtypes [6].

#### 2.2.2 Differential Gene Expression Analysis

R package ‘DESeq2’ was used to identify differentially expressed genes (DEGs) between LC patients and HCs, and DEGs between clusters identified by consensus clustering analysis based on cuproptosis‐related genes. DEGs were identified by with a false discovery rate < 0.05 and an absolute log2 fold change (|log2FC|) ≥ 1 as criteria.

#### 2.2.3 Enrichment Analysis

DEGs between the ‘control’ and ‘treat’ group, and DEGs among subtypes based on cuproptosis‐related genes were applied for enrichment analysis. Gene Ontology (GO) and Kyoto Encyclopedia of Genes and Genomes (KEGG) enrichment analyses were performed for the DEGs. Gene set enrichment analysis (GSEA) identified pathways (*p* < 0.05) among the DEGs with the gene sets ‘c5.go.v2023.1.Hs.symbols’, ‘c2.cp.kegg.v2023.1.Hs.symbols’, ‘c2.cp.reactome.v2023.1.Hs.symbols’ and ‘c2.cp.wikipathways.v2023.1.Hs.symbols’ from the Molecular Signatures Database. Gene set variation analysis (GSVA) with MSigDB hallmark gene sets revealed pathway activity variations across cuproptosis subtypes.

#### 2.2.4 Cuproptosis‐Related Gene Expression Pattern Analysis

The ggplot function was used to analyse the expression of 10 key cuproptosis‐related genes across the 2 clusters, with the goal of identifying cuproptosis‐related gene expression patterns. Weighted gene coexpression network analysis (WGCNA) was employed to detect module eigengenes (MEs) with module membership (MM) > 0.8 and gene significance (GS) > 0.2. This involved soft thresholding, construction of a topological overlap matrix and hierarchical clustering, following the removal of outliers.

#### 2.2.5 Construction and Verification of a Cuproptosis Diagnostic Model

A robust feature selection strategy was used to improve the generalizability of diagnostic model and mitigate overfitting and reduce the challenges of high‐dimensional and sparse transcriptomic data. F2–F4 stages fibrosis and cuproptosis subtype‐related genes were used for model construction. Least absolute shrinkage and selection operator (LASSO) regression were used, which incorporates L1 regularization to efficiently handle sparse data structures by compressing the coefficients of noninformative features to zero, thereby reducing noise and computational cost. Moreover, support vector machine (SVM) and Gaussian mixture model (GMM) algorithms were used to select hub genes. The ‘rms’ R package was used to develop a cuproptosis‐related hub genes nomogram model. The GSE135251 dataset was used as internal validation set, and the GSE162694 dataset was used as external validation set. The area under the receiver operating characteristic (AUROC) curve was used to evaluate the discrimination of model. Calibration curves were used to evaluate the calibration of model. Decision curve analysis (DCA) was used to evaluate the clinical unity of model.

#### 2.2.6 Integrated Analysis of Immune Infiltration and Core Gene–Immune Cell Correlations

To investigate immune infiltration patterns in liver tissue, CIBERSORT was used to estimate the relative abundance of various immune cell types within the tissue microenvironment, and immune cell populations with a *p* < 0.05 were included for further analysis. To explore the relationships between the expression of the hub genes and immune cell infiltration, immune cell populations with *p* < 0.05 and a Spearman's rank correlation coefficient exceeding 0.3 were selected for further detailed investigation.

#### 2.2.7 Relationships Among Core Genes and Liver Fibrosis Disease Progression

To verify the significance of the expression of the selected hub genes and stages of liver fibrosis, the GSE84044 and GSE6764 datasets were used as independent validation sets, and the R packages ‘rstatix’ and ‘ggpubr’ were used.

### 2.3 Single‐Cell Data Analysis

#### 2.3.1 Cell‐Type Identification in F2‐F4 Liver Fibrosis Stages by the Scissor Algorithm

The Scissor algorithm was used to batch sample phenotypic correlations to identify single‐cell subsets, distinguishing between positive (Scissor+) and negative (Scissor‐) phenotypic characteristics derived from scRNA‐seq data and bulk RNA‐seq data [7]. Integrating the GSE136103 and GSE135251 datasets, we identified cell subsets linked to liver fibrosis phenotypes.

#### 2.3.2 Cuproptosis Score Calculation

The ‘AUCell’ R package with aucMaxRank = 5% and the area under the curve (AUC) based on gene expression ranks was used to evaluate the expression of cuproptosis‐related genes in cell types to explore the regulation of cuproptosis in different cell types.

#### 2.3.3 Identification of Single‐Cell DEGs and Enrichment Analysis

The R package ‘FindAllMarkers’ was used to identify DEGs in different cell types detected by marker genes for the GSE136103 dataset. GO and KEGG enrichment analyses were performed for the DEGs in each cell type to interpret their biological significance.

#### 2.3.4 Cell Trajectory Analysis

The R package ‘Monocle 2.30.1’ was used to reconstruct cell trajectories to explore cellular differentiation paths, dynamic gene expression patterns and expression trends of genes and enrichment items [8].

#### 2.3.5 Cell Communication Analysis

The R package ‘CellChat’ was used to conduct cell–cell communication networks, identify interactions of receptor‐ligand between cell types. Network maps and heatmaps were used to present the signal pathways and strengths of interaction between cell types [9].

### 2.4 Experimental Validation on Human Liver Tissue

#### 2.4.1 Liver Tissue Collection

Ten patients were recruited from the Second Affiliated Hospital of Harbin Medical University between January 2023 and December 2024 and ethical review approval (KY2024‐115) was obtained from our hospital.

Patients were selected on the basis of the following criteria: (1) Age between 18 and 60 years. (2) MASH confirmed by haematoxylin and eosin (HE) staining combined with liver biopsy using the NAFLD Activity Score (NAS) and the Steatosis‐Activity‐Fibrosis (SAF) score.

The following exclusion criteria were applied: (1) Age 85 years or older. (2) Pregnancy or breastfeeding. (3) Conditions leading to fatty liver disease or abnormal liver function, including excessive alcohol consumption, drug‐induced fatty liver disease, hepatolenticular degeneration, hepatitis C virus infection and malnutrition. (4) Weight change greater more than 5% within 3 months. (5) Uncontrolled diabetes (glycated haemoglobin level > 9.0% at screening).

#### 2.4.2 HE Staining

The tissue sections were deparaffinized, stained with haematoxylin for 5–10 min, rinsed, differentiated in 1% hydrochloric acid, and stained with eosin for 1–3 min. After drying, the sections were dehydrated, cleared and sealed with neutral resin.

#### 2.4.3 Masson Staining

Liver tissue sections were deparaffinized and gradually hydrated by immersion in a series of ethanol solutions (95%, 70% and 30%) before being immersed in distilled water. Staining commenced with a brief 10‐s application of Weigert's haematoxylin. To achieve precise differentiation, the sections were treated with 1% acidic alcohol for 10 s and then rinsed. A quick dip in 0.1% ammonia solution for 10 s was used to intensify the blue hue, after which the sections were rinsed again. Next, the sections were stained with Ponceau fuchsin solution for 5 min, followed by a 1‐min rinse in 0.2% glacial acetic acid. To further enhance the staining intensity, 1% phosphomolybdic acid was added dropwise for 1 min. The sections were stained with 2% aniline blue solution for 2 min and rinsed once more with 2% glacial acetic acid for 1 min. Finally, the stained sections were dehydrated, cleared and mounted.

#### 2.4.4 Immunohistochemistry (IHC)

The tissue sections were dewaxed and immersed in a graded series of ethanol solutions (95%, 80% and 70%) for 2 min each. Endogenous peroxidase activity was quenched with 3% hydrogen peroxide, and the samples were incubated at room temperature for 15 min and then rinsed with PBS. Antigen retrieval was performed by microwaving the sections in EDTA at 100°C for 20 min, cooling them to room temperature, and rinsing them with PBS. The slides were blocked with 5% goat serum for 15 min. Anti‐SMOC2 (Cat No: 162278; ZENBIO, 1:50) and anti‐THY1 (Cat No: 66766–1‐IG; Proteintech, 1:1000) antibodies were added, and the samples were incubated overnight, followed by rinsing with PBS. The corresponding secondary antibody was then added, and the samples were incubated for an additional hour at 37°C, followed by a final PBS wash. The DAB reaction was allowed to develop for 5 min, after which the sections were counterstained with haematoxylin, washed with water, dewaxed and sealed.

### 2.5 Structural Analysis and Drug Therapy Target Prediction of SMOC2 and THY1

#### 2.5.1 Drug Molecular Docking and Small Interfering RNA Prediction

The protein structures of SMOC2 and THY1 were predicted by AlphaFold. The small molecule structures were downloaded from TCMSP (https://www.tcmsp‐e.com). Moreover, we downloaded predicted the protein structures from AlphaFold (https://alphafold.ebi.ac.uk/). The target small molecules were processed via dehydration, deionization and hydrogenation. Docked using Vina and visualized within PyMOL. We acquired messenger RNA (mRNA) from NCBI (https://www.ncbi.nlm.nih.gov/) and designed small interfering RNA (siRNA) with siDirect (https://sidirect2.rnai.jp/).

#### 2.5.2 Molecular Dynamics Simulation

Using Gromacs 2023, a 100 ns molecular dynamics simulation was conducted on a protein‐ligand complex. The protein utilized the CHARMM 36 force field, and the ligand topology was constructed using GAFF2. The simulation was carried out in a cubic box under periodic boundary conditions with solvation using the TIP3P water model. Electrostatic interactions were handled via the particle mesh Ewald method, with the Verlet integrator performing the dynamics. Both van der Waals and Coulombic interactions were set with a cutoff of 1.0 nm. Before the main simulation, 100 ps equilibration was performed over isothermal‐isobaric and isothermal‐isochoric phases using 100,000 steps with a coupling constant of 0.1 ps. Following equilibration, the system was simulated for 100 ns at 300 Kelvin and 1 bar.

## 3 Results

### 3.1 Genetic and Functional Changes in Fibrosis Progression

**FIGURE 2:**
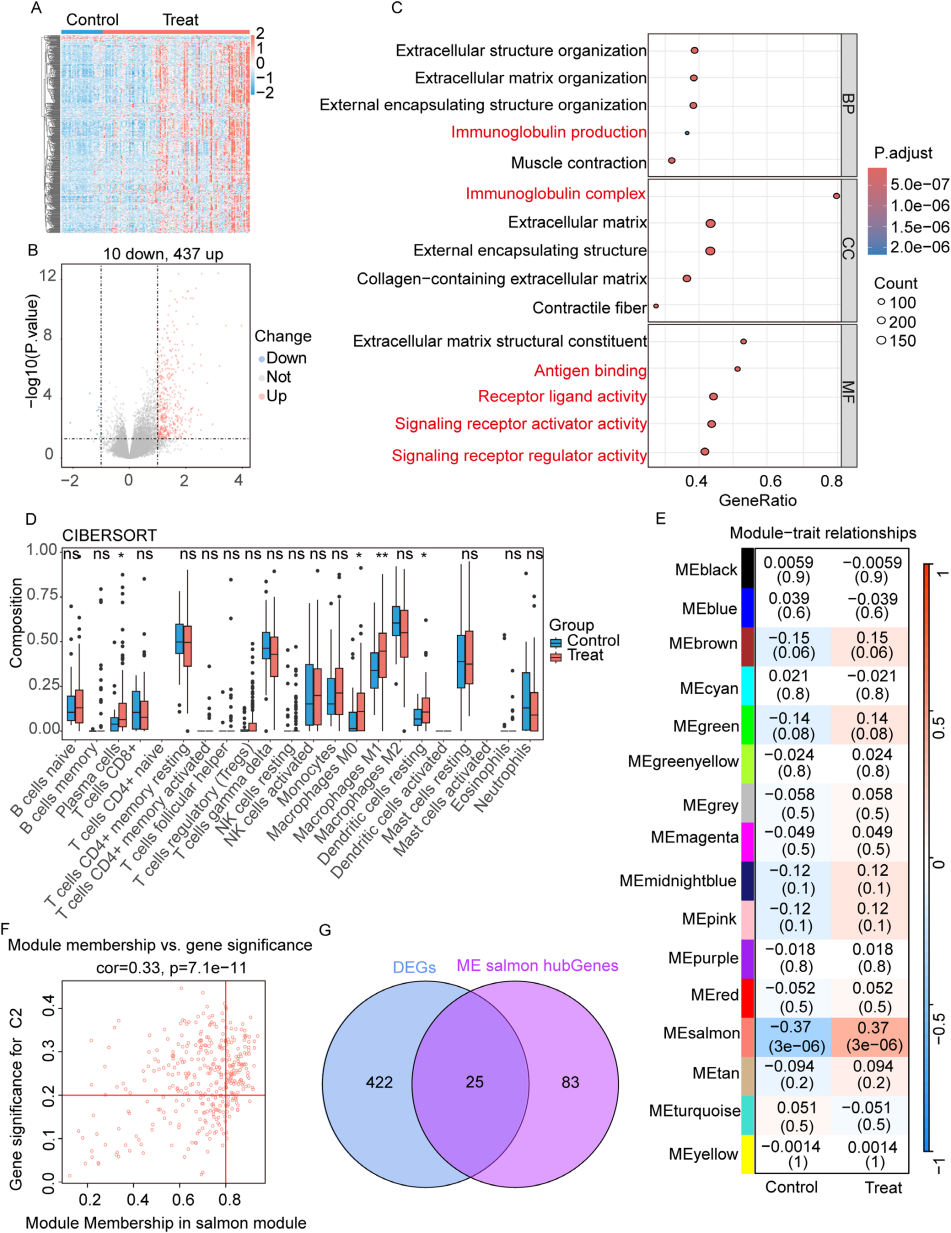
Identification of immune cells in the microenvironment and key genes involved in liver fibrosis. (A) Heatmap of DEGs between the F0–F1 stages and F2–F4 stages liver fibrosis. (B) Volcano plot of the DEGs. (C) Gene set enrichment results. (D) Comparison of immune infiltration (mean ± SD) between the F0–F1 stages group and the F2–F4 stages group. (E) Module gene expression correlated with fibrosis progression. (F) GS/MM scatter plot in the Salmon module. (G) Venn diagram of DEGs and key genes overlapping in the Salmon module.

Differential expression analysis revealed 447 DEGs between the F0–F1 stages and F2–F4 stages liver fibrosis groups (Figure 2A,B). GO enrichment analysis revealed enrichment of DEGs related to immune‐related functions (Figure 2C).

### 3.2 Characterization of Immune Infiltration in Different Stages of Liver Fibrosis

CIBERSORT analysis revealed significant increases in plasma cells, M0/M1 macrophages and resting dendritic cells (DCs) in liver fibrosis patients with F2–F4 stages compared with controls. The number of M2 macrophages tended to decrease (Figure 2D).

### 3.3 Weighted Gene Co‐Expression Network Analysis

WGCNA identified gene modules in the control (F0–F1 stages) and experimental (F2–F4 stages) liver fibrosis groups via weighted co‐expression networks. A soft power of 5 and a dynamic cutoff algorithm identified 15 modules (excluding grey), with the ‘salmon’ module significantly associated with the F2–F4 stages (Figure 2E,F). The Venn diagram showed the DEGs. The Venn diagram shows the intersection of DEGs and MEs, resulting in 25 genes closely associated with liver fibrosis stages F2–F4. This set of 25 genes was named disease WGCNA (Figure 2G).

### 3.4 Unsupervised Consensus Clustering to Identify Cuproptosis Subtypes

**FIGURE 3:**
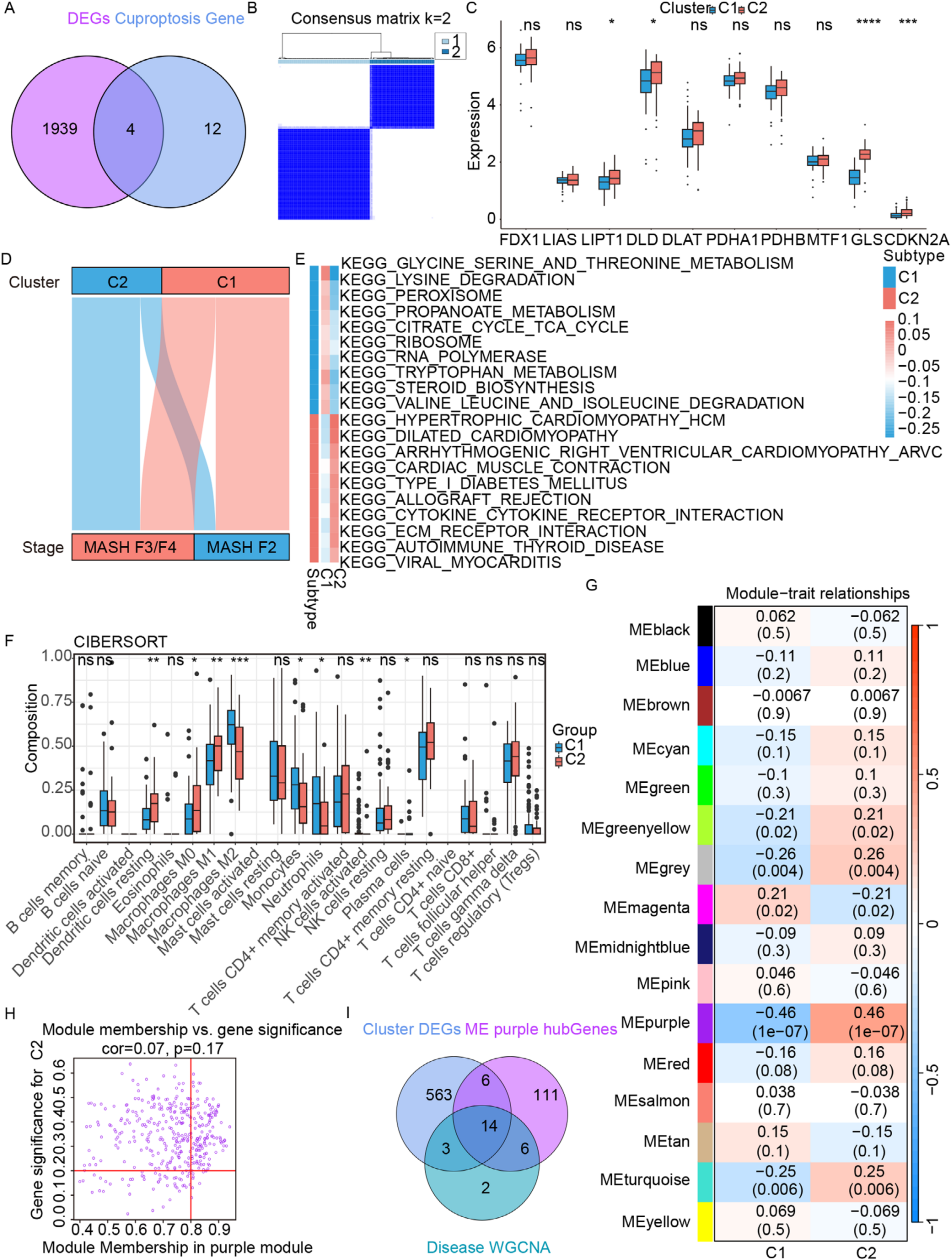
Identification of cuproptosis subtypes and genes related to cuproptosis. (A) Venn diagram of significantly expressed cuproptosis‐related genes. (B) Consensus clustering matrix when *k* = 2. (C) Box plot of cuproptosis gene expression between clusters (mean ± SD). (D) Sankey plot linking cuproptosis subtypes to MASH fibrosis stages. (E) GSVA heatmap of the two cuproptosis subtypes. (F) Box plots comparing immune infiltration between the C1 and C2 groups (mean ± SD). (G) Correlation heatmap of the cuproptosis subtypes and module genes. (H) GS‐MM scatter plot in the purple module. (I) Venn diagram of key disease genes and key subtype genes.

Consensus clustering revealed two cuproptosis patterns (Figure 3A,B, Figure S3). LIPT1, DLD, GLS and CDKN2A were significantly differentially expressed, with LIPT1 and DLD being highly expressed (Figure 3C). Cluster 1 was predominantly linked to Stage 1, whereas Cluster 2 was associated with Stage 2 (Figure 3D). Differential analysis revealed 586 DEGs between clusters (Figure S1C). KEGG enrichment revealed that Cluster 1 genes were associated with the citric acid cycle, whereas Cluster 2 genes were linked to the cytokine receptor interaction pathway (Figure 3E).

### 3.5 Immune Cell Infiltration Characteristics Associated With the Two Cuproptosis Subtypes

Genes that negatively regulate cuproptosis were enriched in immune‐related pathways such as cytokine receptor interaction, Fc gamma receptor (FCGR) activation and FCGR3A‐mediated IL10 synthesis (Figure S1D–G). Compared with those in Cluster 1, the infiltration of Cluster 2 DCs, M0 macrophages and M1 macrophages significantly increased in Cluster C2, whereas the infiltration of M2 macrophages, monocytes and neutrophils significantly decreased in Cluster C2 (Figure 3F).

### 3.6 Subtype Co‐Expression Module Analysis

WGCNA revealed 15 co‐expressed gene modules (excluding grey) using a soft threshold of 8 (Figure S1A,B, Figure 3G). MEpurple was the most strongly correlated with cuproptosis subtypes (cor = 0.46), with its MEs selected for further analysis (Figure 3H). The Venn diagram reveals the intersection of DEGs, MEs and the Disease WGCNA set, identifying 14 genes closely associated with both liver fibrosis stages F2–F4 and cuproptosis subtypes (Figure 3I).

### 3.7 Screening Cuproptosis‐Related Genes By Machine Learning and the Construction of a Diagnostic Model

**FIGURE 4:**
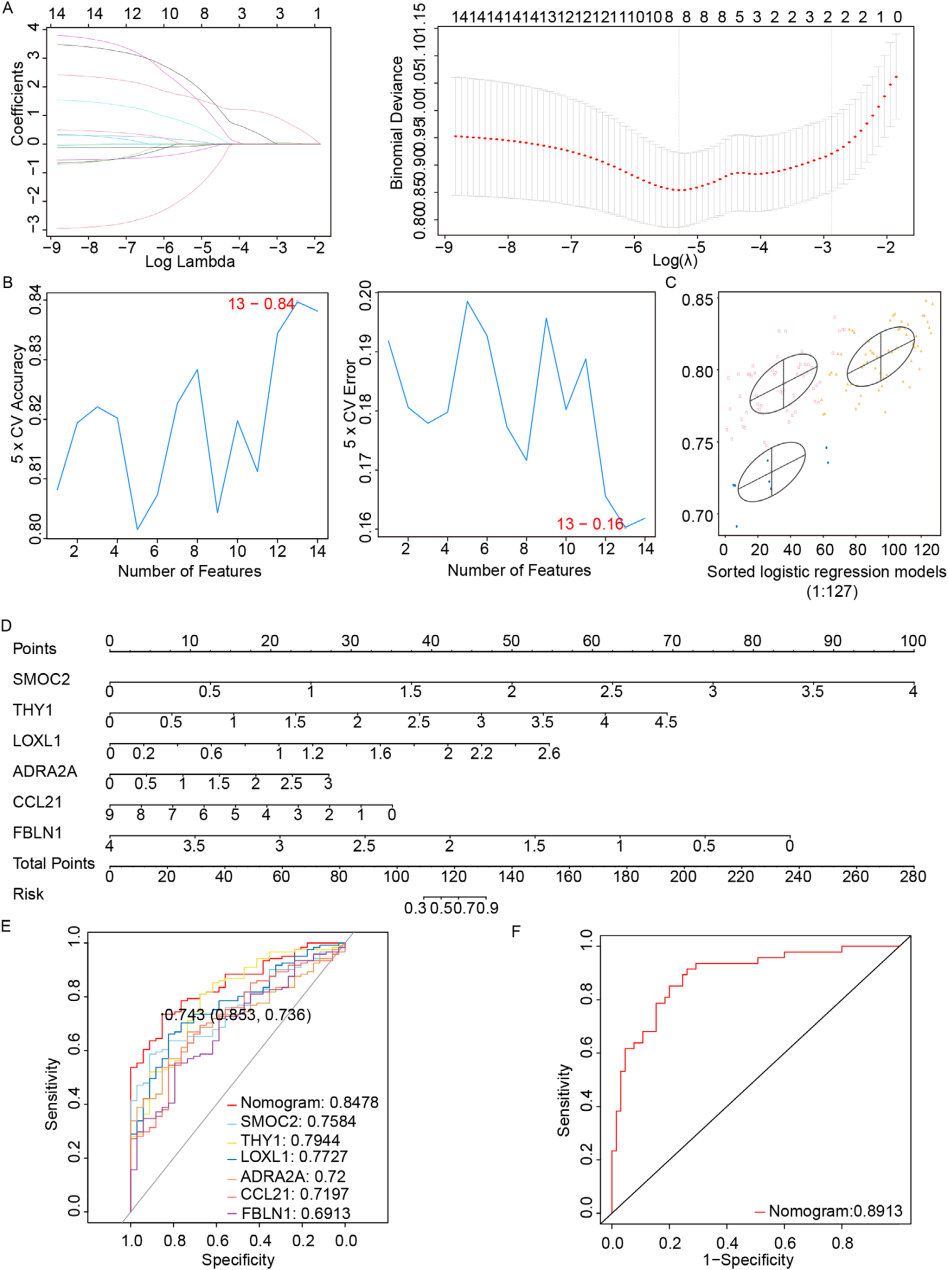
Diagnostic models for genes associated with cuproptosis. (A) LASSO algorithm. (B) SVM screening of candidate genes. (C) The best diagnostic power identified in cluster 3 of the GMM analysis. (D) Nomogram model for cuproptosis‐related genes. (E) The training set ROC curve. (F) Validation set ROC curve.

The LASSO, SVM and GMM algorithms identified six cuproptosis‐related genes (Figure 4A–C). A multivariate logistic regression model was developed using these genes and is presented as a nomogram (Figure 4D). Among the six cuproptosis‐related genes, SMOC2 and THY1 had the highest AUROC values (2 of TOP3). SMOC2 achieved an AUROC of 0.7584, whereas THY1 achieved an AUROC of 0.7944 (Figure 4E). The model achieved high AUC values (0.8478 [95% CI: 0.7859–0.9098] in the training set and 0.8913 [95% CI: 0.8306–0.952] in the external validation set) (Figure 4E,F). DCA confirmed its clinical utility in predicting MASH with F2–F4 stages liver fibrosis (Figure S1A,C). The calibration curve demonstrated that the predicted values were closely aligned with the actual values (mean absolute error = 0.035 in the training set, mean absolute error = 0.022 in the external validation set) (Figure S4B,D).

### 3.8 Exploration of the Functions and Changes of Key Cuproptosis‐Related Diagnostic Genes

**FIGURE 5:**
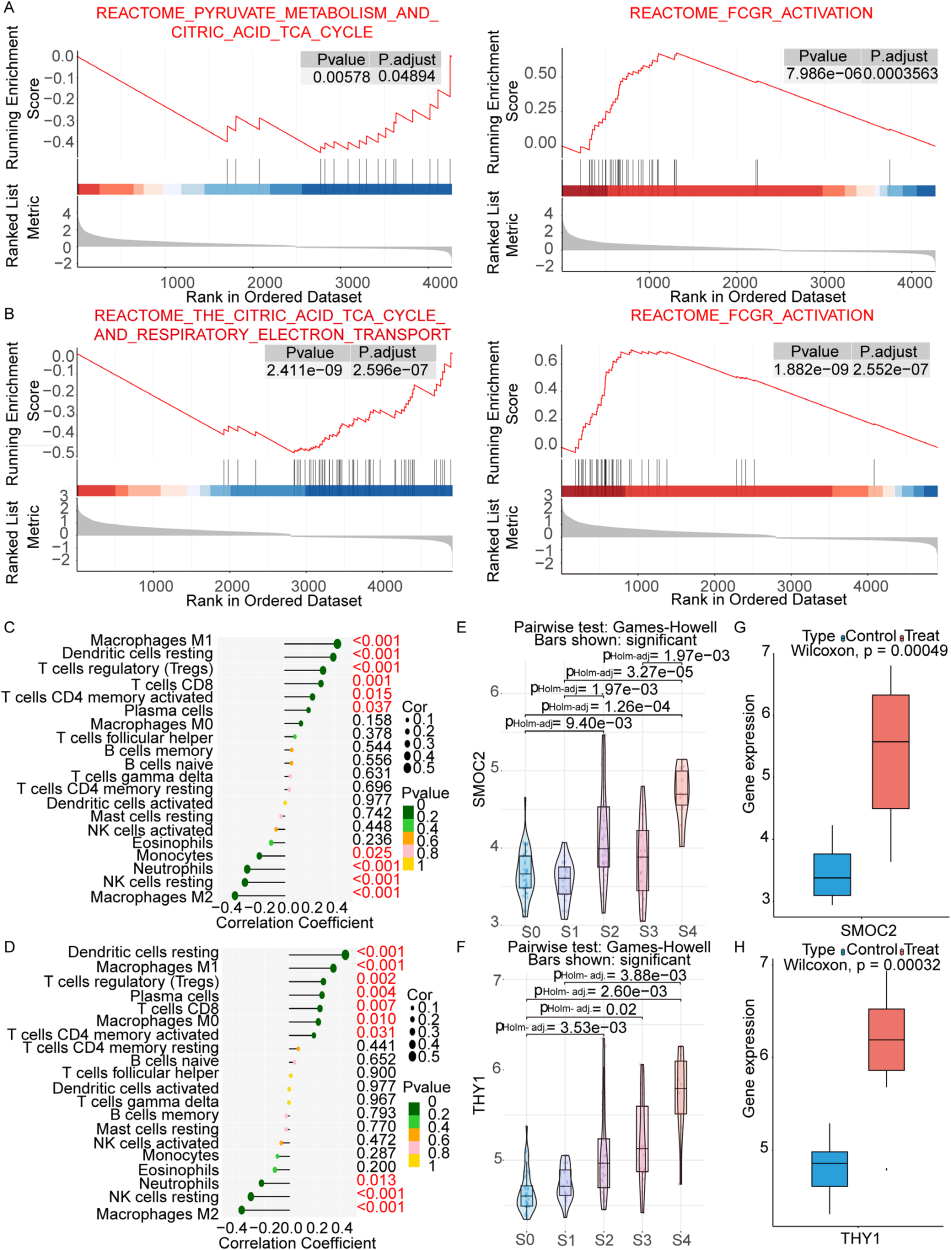
Exploration of the binding capability of THY1 and SMOC2. (A) GSEA of SMOC2. (B) GSEA of THY1. (C) SMOC2‐immune cells associations. (D) THY1‐immune cells associations. (E) Violin plot of SMOC2 expression across liver fibrosis stages. (F) THY1 expression levels in fibrotic progression. (G) SMOC2 expression in HCs and LC patients. (H) THY1 expression in HCs and LC patients.

THY1 and SMOC2 were enriched in the tricarboxylic acid (TCA) cycle and FCGR activation pathways (Figure 5A,B), whereas the other genes were not enriched (Figure S1H–K). Correlation analysis revealed that SMOC2 and THY1 were positively correlated with M1 macrophages and resting DCs, and negatively correlated with resting NK cells (Figure 5C,D). To further assess the link between THY1 and SMOC2 expression and liver fibrosis, the GSE84044 and GSE6764 datasets were analysed. Both genes significantly changed (*p* < 0.05) across different stages of liver fibrosis (Figure 5E–G).

### 3.9 Experimental Validation of Key Genes Related to Cuproptosis

**FIGURE 6:**
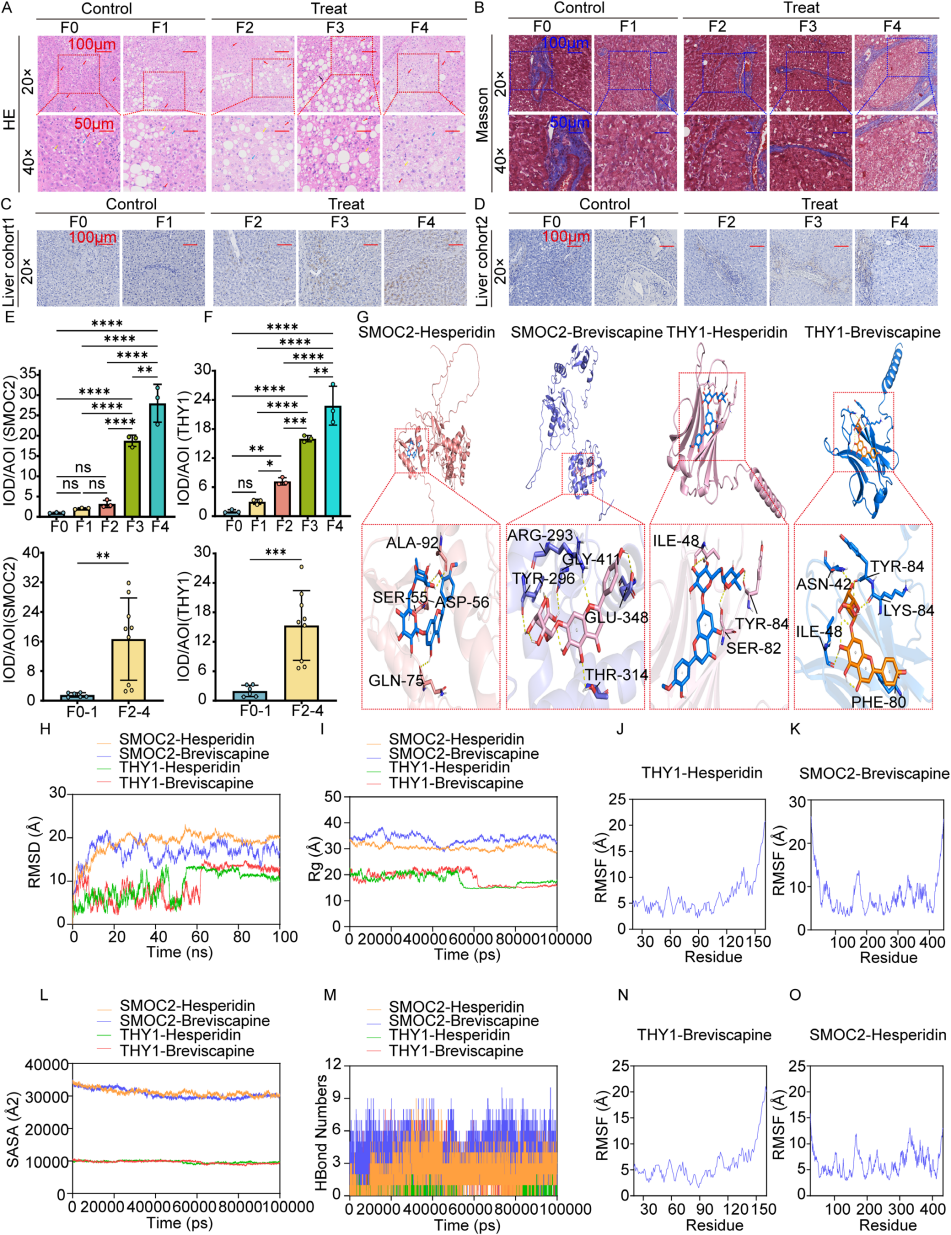
Cuproptosis‐related key genes and targeted drug validation. (A) Steatosis (yellow arrows) and ballooning degeneration (blue arrows) in hepatocytes with lymphocytic infiltration (red arrows) and macrophages (black arrows) in the F0–F4 stages, meeting the NAS and SAF score for MASH diagnosis. (B) Masson staining reveals fibrosis progression: F0 (no fibrosis), F1 (‘chicken‐wire’ pattern), F2 (portal area involvement), F3 (bridging fibrosis) and F4 (pseudobulbs). Scalebar: Top, 100 μm; bottom, 50 μm. (C, E) IHC analysis revealed elevated SMOC2 levels in liver fibrosis stages (scale bars = 100 μm). (D, F) IHC analysis revealed elevated THY1 levels across liver fibrosis stages (scale bars = 100 μm). (G–O) Molecular docking and dynamics parameters of THY1/SMOC2 with hesperidin/breviscapine, which target the cuproptosis‐related pathways. ns *P* ≥ 0.05, * *P* < 0.05, ** *P* < 0.01, *** *P* < 0.001, **** *P* < 0.0001.

The patients were categorized using the NAS and the SAF score (Figure 6A). Fibrosis staging was performed via Masson staining (Figure 6B). SMOC2 expression was low in F0–F1 stages but significantly increased in F2–F4 stages (Figure 6C,E), whereas THY1, which is expressed in fibroblasts, was undetectable in the early stages but was expressed significantly in the F2–F4 stages (Figure 6D,F). These findings suggest that SMOC2 and THY1 may be important biomarkers for MASH disease progression and that their expression is correlated with fibrosis severity.

### 3.10 Hesperidin and Breviscapine Target Cuproptosis‐Related Genes for Liver Fibrosis Therapy

Hesperidin and breviscapine may demonstrate high‐affinity binding to SMOC2 and THY1 (Figure 6G), accompanied by antioxidant and anti‐inflammatory properties. The results of the preliminary siRNA design are shown in Tables S4 and S5. To explore a potential physical interaction between SMOC2 and THY1, protein–protein interaction prediction using AlphaFold‐Multimer was used. However, the model did not predict a high‐confidence direct physical interaction (pTM < 0.6, IPTM < 0.5), suggesting that if they functionally cooperate in liver fibrosis, it is likely through indirect mechanisms or within a larger complex.

### 3.11 Breviscapine Is the Most Valuable Drug Targeting THY1

**FIGURE 7:**
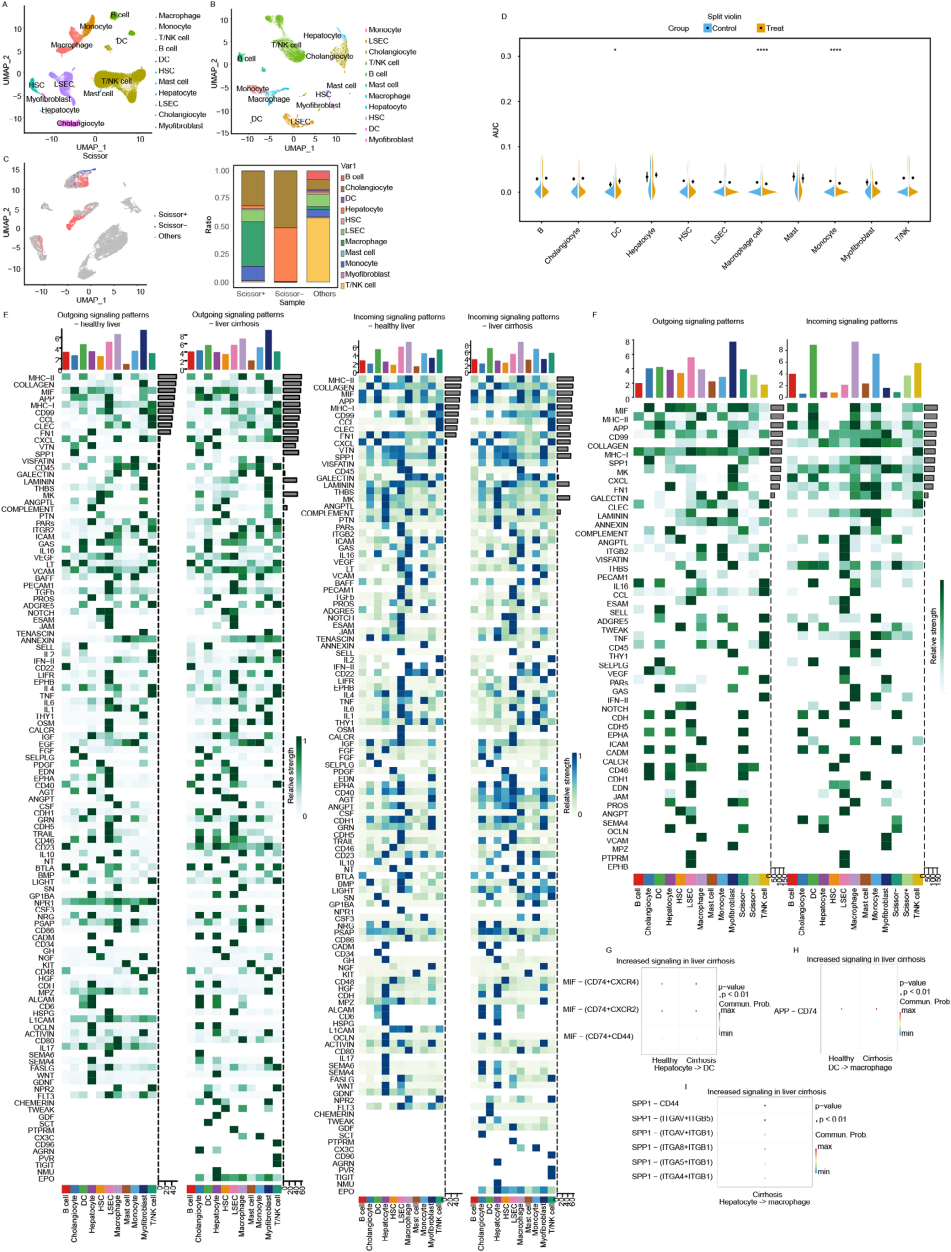
Cuproptosis score and pathway screening between DCs and macrophages. (A) UMAP plot of cell clusters in the LC and HC groups. (B) UMAP plot of cell clusters in LC. (C) Scissor‐based cell selection: Blue (Scissor+) and red (Scissor−) cells correlate with liver fibrosis progression. (D) The levels of cuproptosis in different cells were scored using Seurat's AddModuleScore function. Wilcoxon test, *p < 0.05, **p < 0.01, ***p < 0.001. (E) Outgoing signalling patterns and incoming signalling patterns. (F) Identification of outgoing and incoming signalling pathways based on the Scissor algorithm. (G–I) Key receptors and ligands for intercellular communication in hepatocytes, macrophages and DCs. * *P* < 0.05, **** *P* < 0.0001.

Root mean square deviation analysis revealed that the THY1‐breviscapine and THY1‐hesperidin complexes reached equilibrium at 80 ns, with even smaller fluctuations of 13.6 and 12.8 Å, respectively, than those of the other complexes (Figure 6H). Further analysis revealed that the radius of gyration and solvent accessible surface area of the complex system exhibited minor fluctuations during the dynamic process, indicating that the binding of small molecules induced conformational changes in the target protein (Figure 6I,L). The THY1‐breviscapine, THY1‐hesperidin, SMOC2‐breviscapine and SMOC2‐hesperidin complex systems exhibited relatively high RMSF values (mostly below 9 Å), indicating that the THY1 and SMOC2 target proteins possess significant flexibility (Figure 6J,K,N,O). The THY1‐breviscapine complex exhibited favourable hydrogen bonding interactions. Therefore, breviscapine may demonstrate strong binding efficacy with THY1 (Figure 7J).

### 3.12 Cell Type Identification and Functional Exploration

Eleven cell types in the GSE136103 dataset were identified (Figure 5A) (Tables S2 and S3). Macrophage DEGs were linked to negative cell death, and dendritic cell DEGs were associated with programmed cell death (Figure S2F–G).

### 3.13 Key Gene Localization and Cell Trajectory Analysis

THY1 and SMOC2 were expressed mainly in hepatic stellate cells (HSCs), myofibroblasts and liver sinusoidal endothelial cells (LSECs) (Figure S2A,B). Cell trajectory analysis revealed HSC‐to‐myofibroblast transformation (Figure S2C) and three dynamic gene expression patterns over time (Figure S2D). THY1 expression in cluster 1 increased over time, whereas SMOC2 expression peaked but then decreased (Figure S2E).

### 3.14 Cuproptosis Score at the Single‐Cell Level

AUCell analysis revealed significant differences in the cuproptosis scores across cell types (Figure 7D). Cuproptosis scores were upregulated in DCs, whereas they were downregulated in macrophages and monocytes. Furthermore, hepatocytes had the highest cuproptosis score across all the cell types (Figure 7A). These results revealed the susceptibility of cuproptosis in different cell types.

### 3.15 The Scissor Algorithm Was Used to Identify the Key Cell Types Involved in Fibrosis

Eleven cell types were identified with analysis of the GSE136103 datasets (Figure 7B) (Tables S2 and S3). Based on the Scissor algorithm, macrophages, monocytes and LSECs were identified as fibrosis drivers, whereas cholangiocytes and hepatocytes were identified as inhibitors (Figure 6C). The DEGs were enriched in immune‐related processes and antigen processing pathways (Figure S4G,H), which suggest the immune response and cellular communications in the pathogenesis of liver fibrosis (Figure 7E,F).

### 3.16 Reanalysis of Key Gene Locations and Cell Trajectories at the Cellular Level

UMAP revealed the coordinated spatial distribution patterns of THY1 and SMOC2. THY1 was expressed in HSCs, myofibroblasts and LSECs (Figure S4F). SMOC2 was highly expressed in HSCs and myofibroblasts (Figure S4E). Cell trajectory analysis revealed HSC‐to‐myofibroblast transformation over time, and the cells remained in the first state over pseudotime (Figure S4I). Three clusters were identified (Figure S4J). In cluster 3, THY1 expression fluctuated, whereas in cluster 2, SMOC2 expression showed a similar trend (Figure S4M). The dot plot displays the enrichment analysis results for clusters 2 and 3 (Figure S4K,L).

### 3.17 The Latent Immune Microenvironment Is Regulated by Hepatocyte Cuproptosis

Given the results of the cuproptosis score enrichment analysis at the cellular level, we focused on the interactions between hepatocytes, macrophages and DCs (Figures S2H and S4N). Interaction heatmap analysis (Figure 7E,F) revealed experimental group‐activated pathways that were subsequently mapped onto the Scissor+ cells. We propose that hepatocytes undergoing cuproptosis may trigger liver fibrosis through a multi‐step intercellular cascade (Figure 1). Initially, hepatocytes release the cytokine MIF, which can bind to the CD74‐CXCR4 receptor complex on dendritic cells (DCs), potentially leading to DC activation (Figure 7G, Figures S2I and S4O). Activated DCs in turn secrete APP, which may interact with CD74 receptors on macrophages, priming them toward a pro‐fibrotic phenotype (Figure 7H, Figures S2I and S4P). In parallel, hepatocyte‐derived SPP1 could further amplify macrophage activation by engaging CD44 receptors (Figure 7I, Figures S2K and S4Q). Macrophages receiving these dual signals—APP from DCs and SPP1 from hepatocytes—then promote the activation of hepatic stellate cells (HSCs), thereby driving extracellular matrix deposition and fibrosis progression. This proposed ligand–receptor network offers a novel perspective on the cellular communication underlying liver fibrogenesis.

## 4 Discussion

Early‐stage MASH fibrosis can be completely reversed. Over time, this progressive fibrosis may culminate in cirrhosis. EASL‐EASD‐EASO metabolic dysfunction‐associated steatotic liver disease (MASLD) guidelines emphasize timely advanced fibrosis assessment to mitigate liver/cardiovascular risks [10]. Liver biopsy, while definitive, is invasive with risks (bleeding and infection) and diagnostic limitations (sampling bias and interpathologist variability) [11].

Blood biomarkers and elastography can reliably predict advanced fibrosis, and their combination enhances diagnostic accuracy [12]. Traditional serological markers lack specificity and sensitivity for staging fibrosis. In contrast to the use of single biomarkers, the establishment of a machine learning model has the potential to increase the diagnostic efficiency for liver fibrosis. Resmetirom has been approved by the Food and Drug Administration for treating MASH with moderate to advanced fibrosis [13]. Traditional Chinese medicine therapy for liver fibrosis is gaining increasing acceptance [14].

siRNAs are double‐stranded RNA molecules of 20–25 nucleotides that regulate gene expression by silencing target genes. Compared with small molecules, monoclonal antibodies and macromolecular drugs, siRNA drugs offer the advantages of a relatively short design and development cycle, various therapeutic options, and few side effects [15], which make siRNA drugs promising candidates for treating diseases.

MASH‐related fibrosis arises from complex cellular crosstalk. Hepatocyte stress and death directly trigger HSC activation [16]. Moreover, hepatocyte death modulates immune responses via macrophages/DCs, driving fibrosis [17]. Interactions between HSC amplify fibrosis progression [18]. The hepatic immune microenvironment, comprising diverse immune cells/signalling pathways, can be dysregulated by extrahepatic stressors, initiating fibrosis [19]. Through the modulation of immune cell communication, immunoregulatory therapy has demonstrated therapeutic efficacy in fibrosis suppression [20]. Cuproptosis, a newly identified form of regulated cell death, remains unexplored in liver fibrosis.

By integrating differential gene analysis with WGCNA, we identified critical MEs associated with cuproptosis and MASH‐related fibrosis. A multi‐algorithm approach incorporating LASSO regression, SVM and GMM was used to select hub genes. Diagnostic model was validated by independent external cohort, and hub genes were validated by IHC. Single‐cell transcriptomic data revealed a potential connection between cuproptosis and fibrogenic signalling mediated by macrophages and DCs. Molecular docking and molecular dynamics revealed that they indicated favourable binding affinity and stability for the THY1‐breviscapine complex, highlighting THY1 as a candidate for therapeutic targeting.

THY1 produces CD90, a heavily glycosylated protein anchored to the cell membrane via a glycosylphosphatidylinositol linkage. CD90 plays an essential role in mediating cell adhesion and communication. Its involvement in fibrotic processes has been observed in multiple organs; for example, in cardiac tissue, the absence of THY1 has been shown to exacerbate fibrosis [21]. Clinically, elevated soluble THY1 levels in serum correlate with increased liver fibrosis risk in patients with MASLD, supporting its utility as a serological biomarker for fibrosis staging. Although THY1 knockout in mice confers protection against liver fibrosis [22], our transcriptomic data revealed detectable THY1 mRNA in fibrotic human liver samples. This apparent divergence between species may reflect distinct biological roles or regulatory mechanisms for THY1 in human disease [23].

SMOC2 belongs to the SPARC family of proteins and contains characteristic Kazal and EF‐hand domains. It has been linked to fibrotic remodelling in the kidney, heart and lung [24, 25, 26]. Elevated plasma levels of SMOC2 are also detected in patients with advanced liver fibrosis, indicating its potential involvement in disease progression [27]. Nevertheless, how THY1 and SMOC2 functionally contribute to liver fibrogenesis remains an area requiring further investigation.

The THY1 and SMOC2 genes contributed significantly to the diagnostic model. Although their interaction in liver fibrosis remains unclear, CD90 has been shown to regulate SPARC expression in gastric cancer [28]. SMOC2 and THY1 are co‐expressed in fibroblasts and enriched in the FCGR activation and TCA cycle pathways, suggesting potential molecular interactions. However, whether these interactions occur at the gene level, mRNA level or protein level is unclear.

While THY1 limits fibrosis in mice, it may promote SMOC2 expression in human fibroblasts. IHC analysis revealed that THY1 was localized to the fibroblast periphery and that SMOC2 was near hepatocytes. We infer that THY1 enhances SMOC2 secretion into hepatocytes and that SMOC2 interacts with hepatocytes, exacerbating cuproptosis and immune microenvironment activation (via DCs/macrophages) and thereby accelerating hepatic stellate cell activation and fibrosis progression.

Previous studies have indicated that within specific tumour microenvironments, MIF interacts with the CD74+CXCR4+ receptor complex on myeloid cells, including B cells [29]. Hepatocyte‐derived MIF is known to recruit CD74^+^ macrophages in MASH progression, promoting inflammation and fibrosis [30]. However, the potential involvement of dendritic cells in MIF‐mediated fibrogenic signalling has not been clarified. In contrast, hepatocyte‐secreted SPP1 is well documented to bind CD44 on macrophages, facilitating their migration and contributing to liver fibrosis [31]. Elucidating whether and how MIF influences dendritic cells to modulate macrophage recruitment could therefore provide important insights into the immune mechanisms driving hepatic fibrosis.

In this study, we provide the first evidence linking cuproptosis to liver fibrosis. However, this study has several limitations. While the diagnostic model we developed has demonstrated efficacy using public liver RNA sequencing datasets, its clinical reliability requires validation through large‐scale prospective cohort studies with serological samples. Moreover, it is undeniable that the experimental validation of THY1 and SMOC2 was performed only in MASH‐induced liver fibrosis, and not in fibrosis caused by viral etiologies such as HCV or HBV. Although siRNAs have been shown to be effective in computer algorithms, their practical application needs further evaluation in animal experiments. Moreover, although the PCA and ‘ConsensusClusterPlus’ R package algorithms themselves do not overlap, they are unable to capture the continuous transition states of genes or their multimembership characteristics [32].

Notably, we propose the use of lipid nanoparticle delivery systems for phase 3 clinical trials of siRNAs validated in animal models, aiming to compensate for the instability caused by the lack of chemical modification of computer‐predicted siRNAs.

Through bioinformatics analysis, we hypothesized that cuproptosis exacerbates liver fibrosis. Future studies should validate this hypothesis systematically. (1) Gene knockout experiments targeting key ligand receptors should be conducted to assess immune microenvironment alterations. (2) High‐throughput chromosome conformation capture technology should be utilized to investigate genomic interactions between THY1 and SMOC2. (3) Cell coculture combined with quantitative PCR should be performed to explore RNA‐level interactions. (4) Cell coculture combined with an enzyme‐linked immunosorbent assay should be used to examine protein‐level interactions. (5) High‐resolution sub‐clustering of HSC‐immune cells should be employed to further elucidate the specific subpopulations that execute cuproptosis in the mechanism of liver fibrosis. Finally, the precise mechanism of SMOC2 entry into hepatocytes remains to be elucidated and represents a critical future research direction.

## 5 Conclusions

This study elucidated the relationships between the expression of cuproptosis‐related genes, specifically THY1 and SMOC2, and MASH‐related liver fibrosis. We developed a potential non‐invasive diagnostic model and proposed a novel mechanism through wihch these genes are associated with an immune microenvironment that promotes liver fibrosis.

## Author Contributions

**Ranyan Gao:** conceptualization (equal), data curation (lead), formal analysis (lead), funding acquisition (supporting), writing – original draft (lead), writing – review and editing (equal). **Hongliang Chen:** conceptualization (equal), formal analysis (supporting), writing – original draft (equal), writing – review and editing (equal). **Jiaxin Wang:** conceptualization (supporting), formal analysis (equal), writing – original draft (supporting). **Lingyi Xu:** conceptualization (supporting), data curation (supporting), formal analysis (supporting). **Fengchun Li:** conceptualization (supporting), data curation (supporting), formal analysis (supporting). **Xinyu Jiang:** conceptualization (supporting), data curation (supporting). **Xinyu Geng:** conceptualization (supporting), writing – original draft (supporting). **Ning Li:** conceptualization (supporting), writing – original draft (supporting). **Ram Prasad Chaulagain:** writing – review and editing (equal). **Babalola Deborah Oluwaseun:** writing – review and editing (supporting). **Wanwei Li:** conceptualization (supporting), writing – review and editing (supporting). **Shizhu Jin:** conceptualization (equal), data curation (equal), formal analysis (equal), funding acquisition (lead), writing – review and editing (equal).

## Funding

This work was supported by the National Natural Science Foundation of China, Number. 82470641. Postgraduate Research & Practice Innovation Program of Harbin Medical University, Number. YJSCX2025‐51HYD.

## Ethics Statement

Patient samples were collected with the approval of the Ethics Committee of the Second Affiliated Hospital of Harbin Medical University (KY2024‐115) and patients provided informed consent.

## Conflicts of Interest

The authors declare no conflicts of interest.

## Supporting information


**Figure S1:** WGCNA and enrichment analysis of individual genes. (A) The scale plot of WGCNA was based on liver fibrosis to identify the optimal vector power (cutoff value = 0.85). (B) scale plot of WGCNA was based on the cuproptosis subtype to identify the optimal vector power (cutoff value = 0.85). (C) Volcano plot of the DEGs between the cuproptosis subtypes. (D) KEGG enrichment analysis of GLS. (E) KEGG enrichment analysis of CDKN2A. (F) KEGG enrichment analysis of DBT. (G) KEGG enrichment analysis of GCSH. (H) KEGG enrichment analysis of ADRA2A. (I) KEGG enrichment analysis of CCL21. (J) KEGG enrichment analysis of LOXL1. (K) KEGG enrichment analysis of FBLN1.
**Figure S2:** Macrophages and DCs emerge as critical immune mediators in cuproptosis‐induced liver fibrosis. (A) Distribution of SMOC2 at the single‐cell level. (B) THY1 expression at the single‐cell level. (C) Pseudotemporal trajectory of HSC‐to‐myofibroblast differentiation. (D) Cluster heatmap of gene expression in cell trajectory. (E) Gene expression dynamics along pseudotime trajectory; SMOC2 (top), THY1 (bottom). (F) GSEA of macrophages. (G) GSEA of DCs. (H) Cell interaction network strength and cell counts in the LC and HC groups. (I, J) Circle plots of inferred signalling pathways (MIF, APP, SPP1) among major cell types in the LC and HC groups.
**Figure S3:** Consensus clustering of cuproptosis subtypes in liver cirrhosis. (A) Cumulative distribution function plot. (B) Plot of delta area. (C) Consensus clustering matrix when *k* = 2. (D) Principal component analysis plot of two clusters.
**Figure S4:** Clinical utility assessment of diagnostic models and single‐cell analysis via the scissor algorithm. (A) Training set DCA analysis. (B) Training set calibration curve. (C) Validation set DCA analysis. (D) Validation set calibration curve. (E, F) UMAP of SMOC2/THY1 expression. (G) KEGG enrichment analysis between Scissor+ and Scissor‐ cells. (H) GO enrichment analysis between Scissor+ and Scissor‐ cells. (I) Cell trajectory of HSC‐to‐MF. (J) Visualizing clustered mRNA expression during cellular differentiation paths. (K) Enrichment analysis plot of Cluster 2 genes. (L) Enrichment analysis plot of Cluster 3 genes. (M) Gene expression trajectories during cell fate transition: SMOC2 (top), THY1 (bottom). (N) Cell interaction network strength and cell counts in the LC groups. (O–Q) Circle plots of inferred signalling pathways (MIF, APP, SPP1) among major cell types in LC groups.

**Table S1:** Datasets used in this study and their sample composition.
**Table S2:** Differential gene expression analysis across cell clusters.
**Table S3:** Cell type annotation.
**Table S4:** siRNA predicted sequence of THY1.
**Table S5:** siRNA predicted sequence of SMOC2.

## Data Availability

RNA‐seq datasets were obtained from GEO database, including GSE135251 (https://www.ncbi.nlm.nih.gov/geo/query/acc.cgi?acc=GSE135251), GSE162694 (https://www.ncbi.nlm.nih.gov/geo/query/acc.cgi?acc=GSE162694), GSE84044 (https://www.ncbi.nlm.nih.gov/geo/query/acc.cgi?acc=GSE84044), GSE6764 (https://www.ncbi.nlm.nih.gov/geo/query/acc.cgi?acc=GSE6764) and GSE136103 (https://www.ncbi.nlm.nih.gov/geo/query/acc.cgi?acc=GSE136103). Raw data and code were deposited at Mendeley (Reserved DOI: 10.17632/bkfbkm9hdb.1). Additional information is available from the corresponding author on reasonable request.
