# Supplementary material for: Bioinformatics and Immunohistochemistry Reveal the Diagnostic and Mechanistic Role of the Cuproptosis‐Related Genes SMOC2/THY1 in Liver Fibrosis
Source: J Cell Mol Med. 2026 Apr 17;30(8):e71144. doi: 10.1111/jcmm.71144 (PMC13090161; doi:10.1111/jcmm.71144)

**Bioinformatics and immunohistochemistry reveal the diagnostic and mechanistic role of the cuproptosis-related genes SMOC2/THY1 in liver fibrosis**

Ranyan Gao1^#^, Hongliang Chen1^#^, Jiaxin Wang1, Lingyi Xu1, Fengchun Li1, Xinyu Jiang1, Xinyu Geng1, Ning Li1, Ram Prasad Chaulagain1, Babalola Deborah Oluwaseun1, Wanwei Li2, Shizhu Jin1

**Supplementary Figures**

**Figure S1. WGCNA and enrichment analysis of individual genes.**

**(A)** The scale plot of WGCNA was based on liver fibrosis to identify the optimal vector power (cutoff value=0.85). **(B)** scale plot of WGCNA was based on the cuproptosis subtype to identify the optimal vector power (cutoff value=0.85). **(C)** Volcano plot of the DEGs between the cuproptosis subtypes. **(D)** KEGG enrichment analysis of GLS. **(E)** KEGG enrichment analysis of CDKN2A. **(F)** KEGG enrichment analysis of DBT. **(G)** KEGG enrichment analysis of GCSH. **(H)** KEGG enrichment analysis of ADRA2A. **(I)** KEGG enrichment analysis of CCL21. **(J)** KEGG enrichment analysis of LOXL1. **(K)** KEGG enrichment analysis of FBLN1.

**Figure S2. Macrophages and DCs emerge as critical immune mediators in cuproptosis-induced liver fibrosis.**

**(A)** Distribution of SMOC2 at the single-cell level. **(B)** THY1 expression at the single-cell level. **(C)** Pseudotemporal trajectory of HSC-to-myofibroblast differentiation. **(D)** Cluster heatmap of gene expression in cell trajectory. **(E)** Gene expression dynamics along pseudotime trajectory; SMOC2 (top), THY1 (bottom). **(F)** GSEA of macrophages. **(G)** GSEA of DCs. **(H)** Cell interaction network strength and cell counts in the LC and HC groups. **(I-J)** Circle plots of inferred signaling pathways (MIF, APP, SPP1) among major cell types in the LC and HC groups.

**Figure S3. Consensus clustering of cuproptosis subtypes in liver cirrhosis.**

**(A)** Cumulative distribution function plot. **(B)** Plot of delta area. **(C)** Consensus clustering matrix when k = 2. **(D)** Principal component analysis plot of two clusters.

**Figure S4. Clinical utility assessment of diagnostic models and single-cell analysis via the scissor algorithm.**

**(A)** Training set DCA analysis. **(B)** Training set calibration curve. **(C)** Validation set DCA analysis. **(D)** Validation set calibration curve. **(E-F)** UMAP of SMOC2/THY1 expression. **(G)** KEGG enrichment analysis between Scissor+ and Scissor- cells. **(H)** GO enrichment analysis between Scissor+ and Scissor- cells. **(I)** Cell trajectory of HSC-to-MF. **(J)** Visualizing clustered mRNA expression during cellular differentiation paths. **(K)** Enrichment analysis plot of cluster 2 genes. **(L)** Enrichment analysis plot of cluster 3 genes. **(M)** Gene expression trajectories during cell fate transition: SMOC2 (top), THY1 (bottom). **(N)** Cell interaction network strength and cell counts in the LC groups. **(O-Q)** Circle plots of inferred signaling pathways (MIF, APP, SPP1) among major cell types in LC groups.

**Supplementary Tables**

**Table S1. Datasets used in this study and their sample composition.**

**Table S2. Differential gene expression analysis across cell clusters**

**Table S3. Cell type annotation**

**Table S4. siRNA predicted sequence of THY1.**

**Table S5. siRNA predicted sequence of SMOC2.**

**Figure S1**


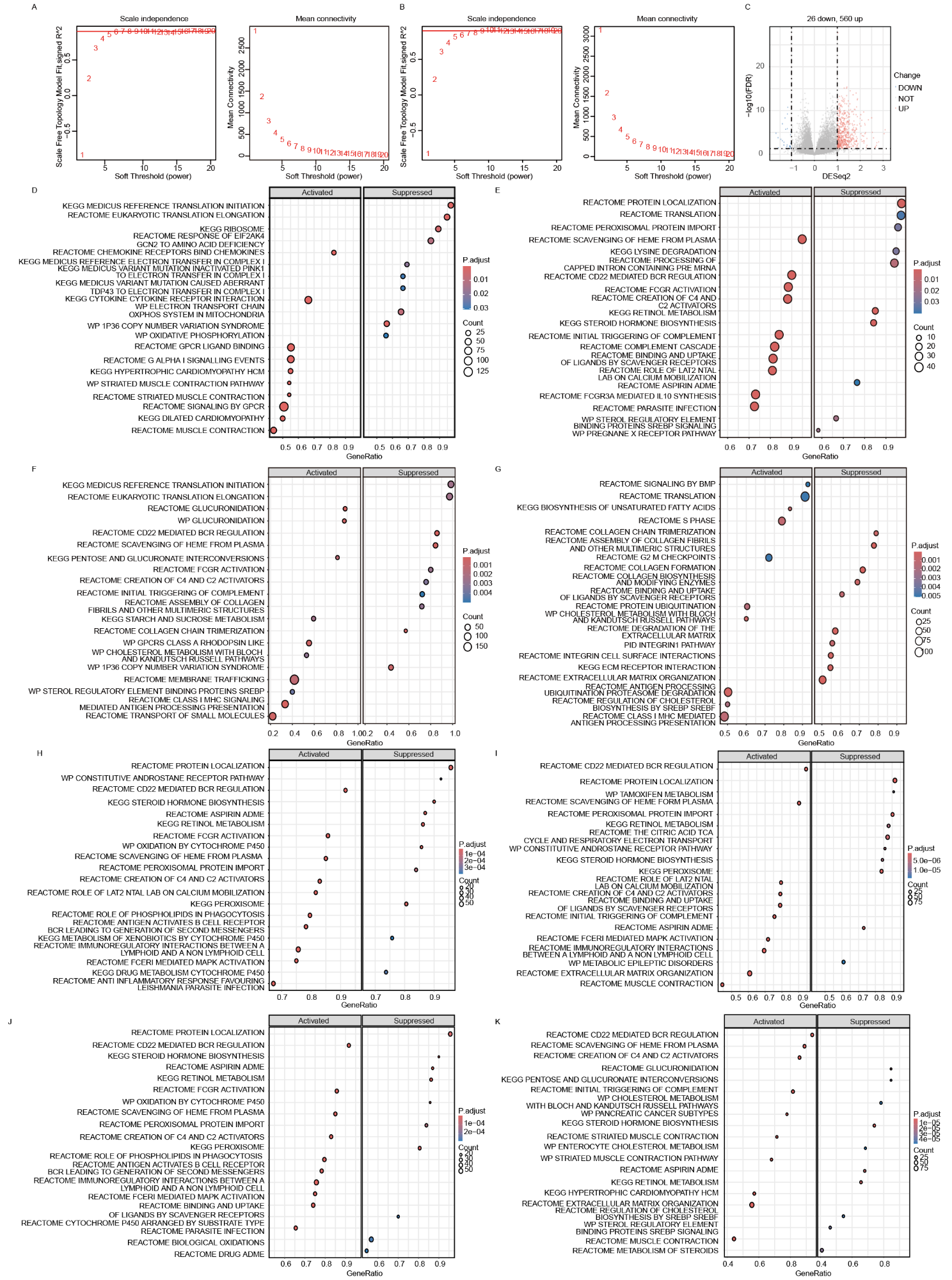


**Figure S2**


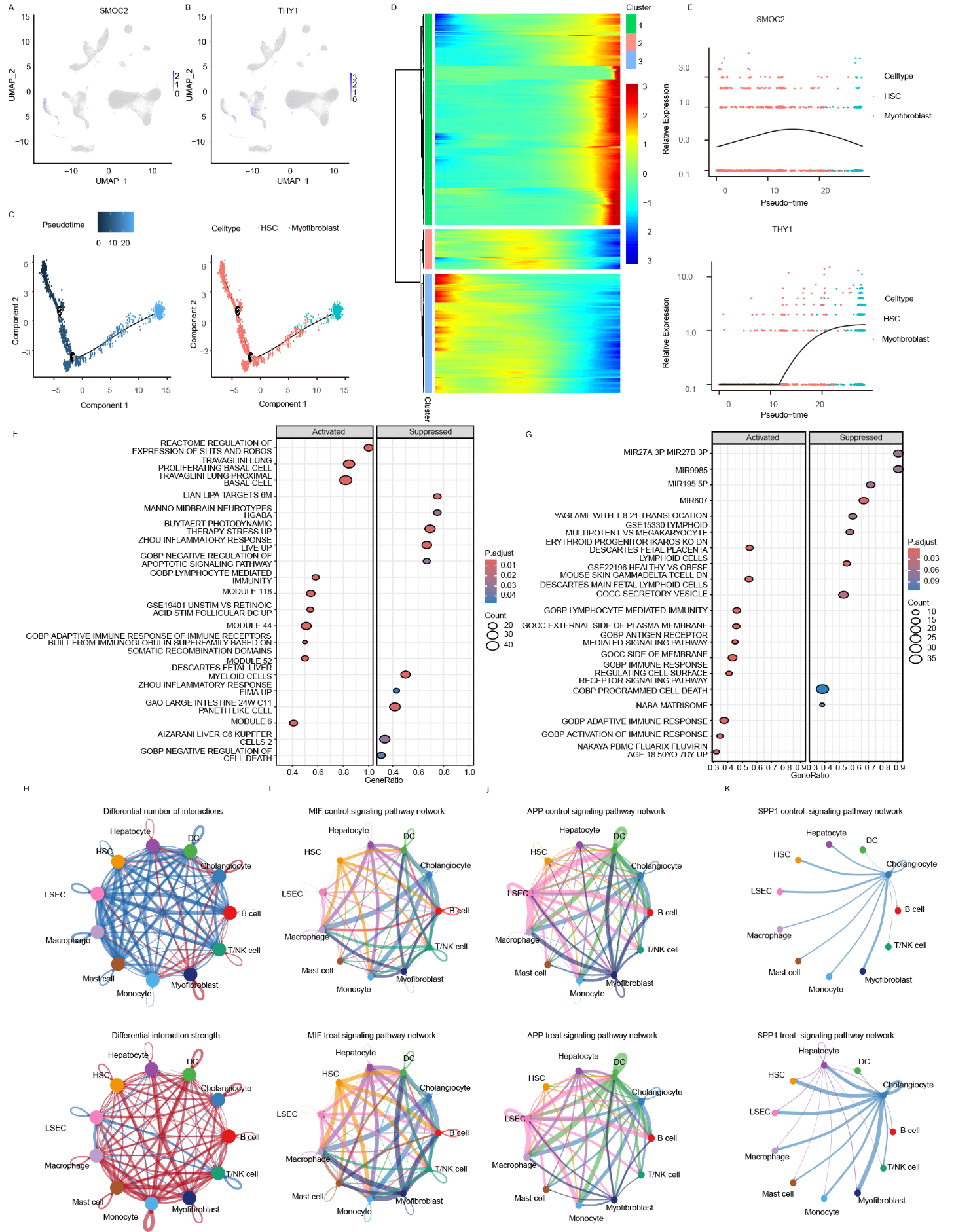


**Figure S3**


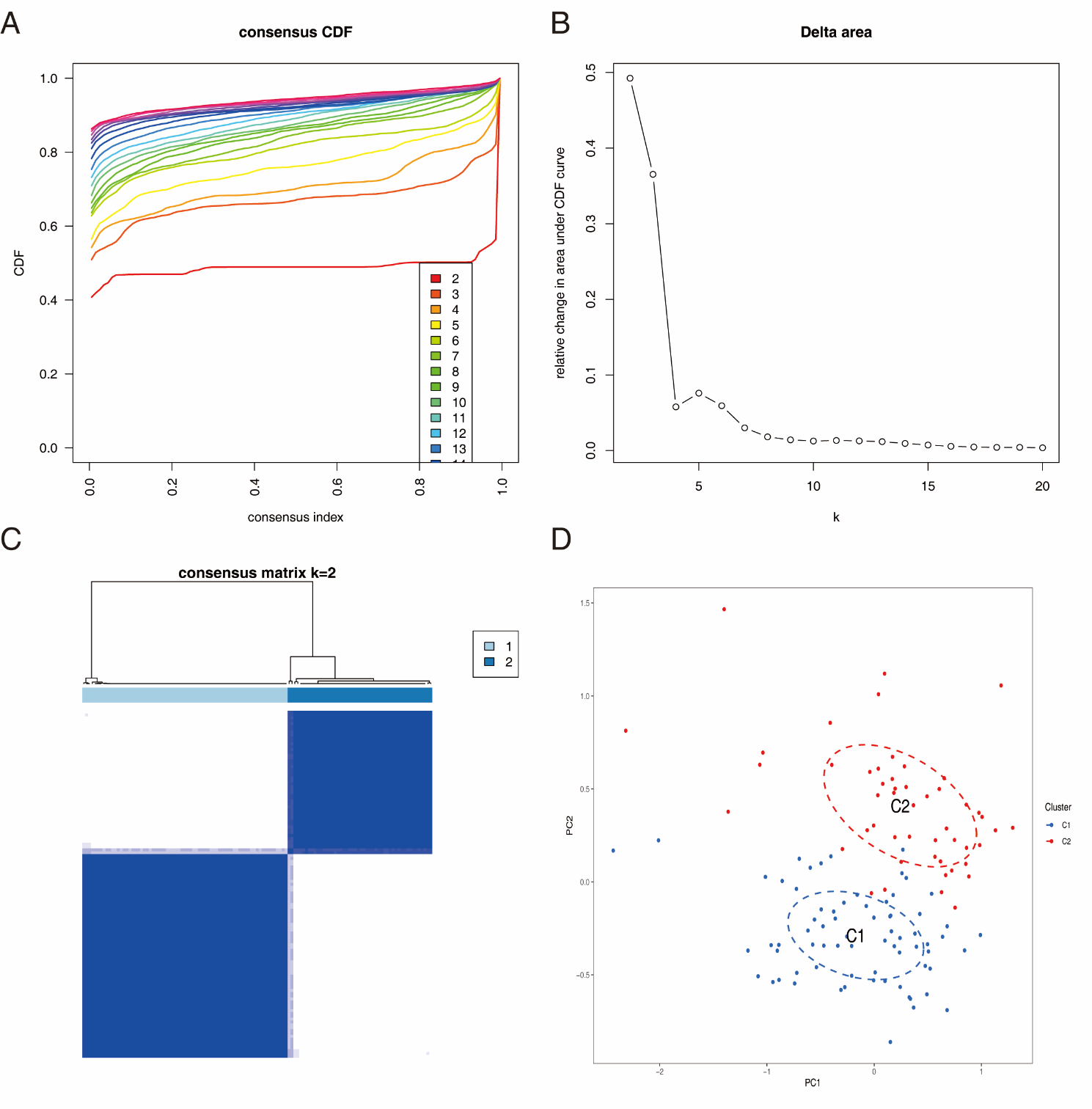


**Figure S4**


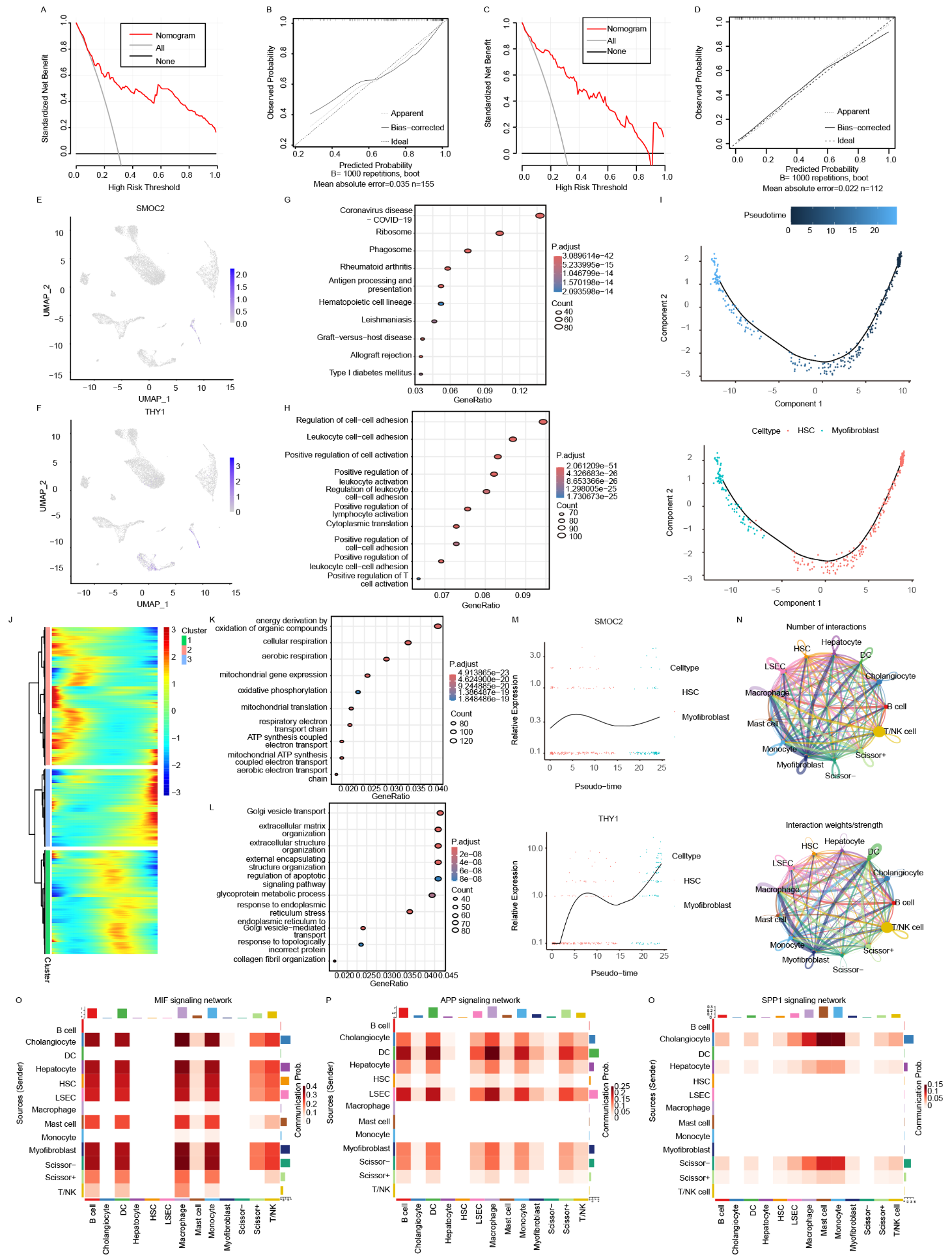

Supplement: Supplementary file 1 — Figure S1: WGCNA and enrichment analysis of individual genes. (A) The scale plot of WGCNA was based on liver fibrosis to identify the optimal vector power (cutoff value = 0.85). (B) scale plot of WGCNA was based on the cuproptosis subtype to identify the optimal vector power (cutoff value = 0.85). (C) Volcano plot of the DEGs between the cuproptosis subtypes. (D) KEGG enrichment analysis of GLS. (E) KEGG enrichment analysis of CDKN2A. (F) KEGG enrichment analysis of DBT. (G) KEGG enrichment analysis of GCSH. (H) KEGG enrichment analysis of ADRA2A. (I) KEGG enrichment analysis of CCL21. (J) KEGG enrichment analysis of LOXL1. (K) KEGG enrichment analysis of FBLN1. Figure S2: Macrophages and DCs emerge as critical immune mediators in cuproptosis‐induced liver fibrosis. (A) Distribution of SMOC2 at the single‐cell level. (B) THY1 expression at the single‐cell level. (C) Pseudotemporal trajectory of HSC‐to‐myofibroblast differentiation. (D) Cluster heatmap of gene expression in cell trajectory. (E) Gene expression dynamics along pseudotime trajectory; SMOC2 (top), THY1 (bottom). (F) GSEA of macrophages. (G) GSEA of DCs. (H) Cell interaction network strength and cell counts in the LC and HC groups. (I, J) Circle plots of inferred signalling pathways (MIF, APP, SPP1) among major cell types in the LC and HC groups. Figure S3: Consensus clustering of cuproptosis subtypes in liver cirrhosis. (A) Cumulative distribution function plot. (B) Plot of delta area. (C) Consensus clustering matrix when k = 2. (D) Principal component analysis plot of two clusters. Figure S4: Clinical utility assessment of diagnostic models and single‐cell analysis via the scissor algorithm. (A) Training set DCA analysis. (B) Training set calibration curve. (C) Validation set DCA analysis. (D) Validation set calibration curve. (E, F) UMAP of SMOC2/THY1 expression. (G) KEGG enrichment analysis between Scissor+ and Scissor‐ cells. (H) GO enrichment analysis between Scissor+ and Scissor‐ cells. (I) Cell tr [file JCMM-30-e71144-s002.docx]
