# Supplementary material for: Bioinformatics and Immunohistochemistry Reveal the Diagnostic and Mechanistic Role of the Cuproptosis‐Related Genes SMOC2/THY1 in Liver Fibrosis
Source: J Cell Mol Med. 2026 Apr 17;30(8):e71144. doi: 10.1111/jcmm.71144 (PMC13090161; doi:10.1111/jcmm.71144)
Supplement: Supplementary file 2 — Table S1: Datasets used in this study and their sample composition. Table S2: Differential gene expression analysis across cell clusters. Table S3: Cell type annotation. Table S4: siRNA predicted sequence of THY1. Table S5: siRNA predicted sequence of SMOC2. [file JCMM-30-e71144-s001.pdf]

Table S1. Datasets used in this study and their sample composition.

Legend:
We downloaded the original datasets from the GEO database and screened the samples based on screening criteria for transcriptome and single-cell analysis.

Table Structure:
- Sheet "screening criteria": Criteria for screening samples for subsequent analysis.
- Sheets "GSE": The original datasets downloaded from the GEO database.

For Peer Review

Table S1a. Screening Criteria for Gene Expression Omnibus Datasets

Dataset	Classification
GSE135251	Bulk RNA-seq
GSE162694	
GSE84044	
GSE6764	
GSE136103	scRNA-seq

[1]The MASH fibrosis grades were divided into F0, F1, F2, F3, and F4 [1][2].

[2]According to the Scheuer scoring system, fibrosis grades of chronic hepatitis were divided into

[1] Govaere O, Cockell S, Tiniakos D, et al. Transcriptomic profiling across the nonalcoholic fa

[2] Pantano L, Agyapong G, Shen Y, et al. Molecular characterization and cell type compositio

[3] Wang M, Gong Q, Zhang J, et al. Characterization of gene expression profiles in HBV-relat

Applying the aforementioned screening criteria, we curated relevant datasets from the GEO database and conducted a targeted statistical analysis aligned with our research objectives.

For Peer Review

Database	Platform	Function
GEO	GPL18573	Training set for the diagnostic model
GEO	GPL21290	Validation set for the diagnostic mode
GEO	GPL570	Validation set for the key genes
GEO	GPL570	Validation set for the key genes
GEO	GPL20301	Single-cell analysis of cuproptosis in

[illegible] S0, S1, S2, S3, and S4[3].

[illegible]atty liver disease spectrum reveals gene signatures for steatohepatitis and fibrosis. *Science translational medicine*. 2020;12(572):eab[illegible]
[illegible]n deconvolution of fibrosis in NAFLD. *Scientific reports*. 2021;11(1):18045. doi:10.1038/s41598-021-96966-5
[illegible]ed liver fibrosis patients and identification of ITGBL1 as a key regulator of fibrogenesis. *Scientific reports*. 2017;7:43446. doi:10.1[illegible]

For Peer Review

Sample
121 cases of F2-F4 MASH and 34 F0-F1 MASH[1]
47 cases of F2-F4 MASH and 65 F0-F1 MASH[1]
43 cases of S0, 20 cases of S1, 33 cases of S2, 18 cases of S3, 10 cases of S4[2]
10 cases of LC and 10 controls
5 cases of LC and 5 controls

ɔa4448. doi:10.1126/scitranslmed.aba4448

038/srep43446

For Peer Review

Table S1b. Sample Information of GSE135251

!Sample_t	!Sample_g	!Sample_s	!Sample_s	!Sample_l	!Sample_t	!Sample_c	!Sample_s	!Sample_o
Liver pat	GSM399816	Public on	Aug 01 20	Apr 28 20	SRA	1	NAFLD_ear	Homo sapi
Liver pat	GSM399816	Public on	Aug 01 20	Apr 28 20	SRA	1	NAFLD_mod	Homo sapi
Liver pat	GSM399816	Public on	Aug 01 20	Apr 28 20	SRA	1	NAFLD_ear	Homo sapi
Liver pat	GSM399817	Public on	Aug 01 20	Apr 28 20	SRA	1	NAFLD_mod	Homo sapi
Liver pat	GSM399817	Public on	Aug 01 20	Apr 28 20	SRA	1	NAFLD_ear	Homo sapi
Liver pat	GSM399817	Public on	Aug 01 20	Apr 28 20	SRA	1	NAFLD_ear	Homo sapi
Liver pat	GSM399817	Public on	Aug 01 20	Apr 28 20	SRA	1	NAFLD_mod	Homo sapi
Liver pat	GSM399817	Public on	Aug 01 20	Apr 28 20	SRA	1	NAFLD_ear	Homo sapi
Liver pat	GSM399817	Public on	Aug 01 20	Apr 28 20	SRA	1	NAFLD_ear	Homo sapi
Liver pat	GSM399817	Public on	Aug 01 20	Apr 28 20	SRA	1	NAFLD_ear	Homo sapi
Liver pat	GSM399817	Public on	Aug 01 20	Apr 28 20	SRA	1	NAFLD_ear	Homo sapi
Liver pat	GSM399817	Public on	Aug 01 20	Apr 28 20	SRA	1	NAFLD_ear	Homo sapi
Liver pat	GSM399817	Public on	Aug 01 20	Apr 28 20	SRA	1	NAFLD_mod	Homo sapi
Liver pat	GSM399818	Public on	Aug 01 20	Apr 28 20	SRA	1	NAFLD_ear	Homo sapi
Liver pat	GSM399818	Public on	Aug 01 20	Apr 28 20	SRA	1	NAFLD_ear	Homo sapi
Liver pat	GSM399818	Public on	Aug 01 20	Apr 28 20	SRA	1	NAFLD_ear	Homo sapi
Liver pat	GSM399818	Public on	Aug 01 20	Apr 28 20	SRA	1	NAFLD_ear	Homo sapi
Liver pat	GSM399818	Public on	Aug 01 20	Apr 28 20	SRA	1	NAFLD_ear	Homo sapi
Liver pat	GSM399818	Public on	Aug 01 20	Apr 28 20	SRA	1	NAFLD_mod	Homo sapi
Liver pat	GSM399818	Public on	Aug 01 20	Apr 28 20	SRA	1	NAFLD_ear	Homo sapi
Liver pat	GSM399818	Public on	Aug 01 20	Apr 28 20	SRA	1	NAFLD_ear	Homo sapi
Liver pat	GSM399818	Public on	Aug 01 20	Apr 28 20	SRA	1	NAFLD_mod	Homo sapi
Liver pat	GSM399818	Public on	Aug 01 20	Apr 28 20	SRA	1	NAFLD_ear	Homo sapi
Liver pat	GSM399819	Public on	Aug 01 20	Apr 28 20	SRA	1	NAFLD_mod	Homo sapi
Liver pat	GSM399819	Public on	Aug 01 20	Apr 28 20	SRA	1	NAFLD_mod	Homo sapi
Liver pat	GSM399819	Public on	Aug 01 20	Apr 28 20	SRA	1	NAFLD_ear	Homo sapi
Liver pat	GSM399819	Public on	Aug 01 20	Apr 28 20	SRA	1	NAFLD_mod	Homo sapi
Liver pat	GSM399819	Public on	Aug 01 20	Apr 28 20	SRA	1	NAFLD_ear	Homo sapi
Liver pat	GSM399819	Public on	Aug 01 20	Apr 28 20	SRA	1	NAFLD_mod	Homo sapi
Liver pat	GSM399819	Public on	Aug 01 20	Apr 28 20	SRA	1	NAFLD_ear	Homo sapi
Liver pat	GSM399819	Public on	Aug 01 20	Apr 28 20	SRA	1	NAFLD_ear	Homo sapi
Liver pat	GSM399819	Public on	Aug 01 20	Apr 28 20	SRA	1	NAFLD_ear	Homo sapi
Liver pat	GSM399819	Public on	Aug 01 20	Apr 28 20	SRA	1	NAFLD_ear	Homo sapi
Liver pat	GSM399820	Public on	Aug 01 20	Apr 28 20	SRA	1	NAFLD_mod	Homo sapi
Liver pat	GSM399820	Public on	Aug 01 20	Apr 28 20	SRA	1	NAFLD_mod	Homo sapi
Liver pat	GSM399820	Public on	Aug 01 20	Apr 28 20	SRA	1	NAFLD_ear	Homo sapi
Liver pat	GSM399820	Public on	Aug 01 20	Apr 28 20	SRA	1	NAFLD_mod	Homo sapi
Liver pat	GSM399820	Public on	Aug 01 20	Apr 28 20	SRA	1	NAFLD_ear	Homo sapi
Liver pat	GSM399820	Public on	Aug 01 20	Apr 28 20	SRA	1	NAFLD_ear	Homo sapi
Liver pat	GSM399820	Public on	Aug 01 20	Apr 28 20	SRA	1	NAFLD_ear	Homo sapi
Liver pat	GSM399820	Public on	Aug 01 20	Apr 28 20	SRA	1	NAFLD_mod	Homo sapi
Liver pat	GSM399820	Public on	Aug 01 20	Apr 28 20	SRA	1	NAFLD_mod	Homo sapi
Liver pat	GSM399820	Public on	Aug 01 20	Apr 28 20	SRA	1	NAFLD_mod	Homo sapi
Liver pat	GSM399821	Public on	Aug 01 20	Apr 28 20	SRA	1	NAFLD_mod	Homo sapi
Liver pat	GSM399821	Public on	Aug 01 20	Apr 28 20	SRA	1	NAFLD_ear	Homo sapi
Liver pat	GSM399821	Public on	Aug 01 20	Apr 28 20	SRA	1	NAFLD_ear	Homo sapi
Liver pat	GSM399821	Public on	Aug 01 20	Apr 28 20	SRA	1	NAFLD_ear	Homo sapi
Liver pat	GSM399821	Public on	Aug 01 20	Apr 28 20	SRA	1	NAFLD_mod	Homo sapi
Liver pat	GSM399821	Public on	Aug 01 20	Apr 28 20	SRA	1	NAFLD_ear	Homo sapi
Liver con	GSM399821	Public on	Aug 01 20	Apr 28 20	SRA	1	Control_c	Homo sapi
Liver con	GSM399821	Public on	Aug 01 20	Apr 28 20	SRA	1	Control_c	Homo sapi
Liver con	GSM399821	Public on	Aug 01 20	Apr 28 20	SRA	1	Control_c	Homo sapi
Liver pat	GSM399821	Public on	Aug 01 20	Apr 28 20	SRA	1	NAFLD_ear	Homo sapi
Liver con	GSM399822	Public on	Aug 01 20	Apr 28 20	SRA	1	Control_c	Homo sapi
Liver con	GSM399822	Public on	Aug 01 20	Apr 28 20	SRA	1	Control_c	Homo sapi
Liver con	GSM399822	Public on	Aug 01 20	Apr 28 20	SRA	1	Control_c	Homo sapi

For Peer Review

Liver	con	GSM399822	Public	on	Aug	01	20	Apr	28	20	SRA	1	Control_c	Homo	sapi
Liver	con	GSM399822	Public	on	Aug	01	20	Apr	28	20	SRA	1	Control_c	Homo	sapi
Liver	pat	GSM399822	Public	on	Aug	01	20	Apr	28	20	SRA	1	NAFLD_mod	Homo	sapi
Liver	pat	GSM399822	Public	on	Aug	01	20	Apr	28	20	SRA	1	NAFLD_mod	Homo	sapi
Liver	pat	GSM399822	Public	on	Aug	01	20	Apr	28	20	SRA	1	NAFLD_mod	Homo	sapi
Liver	pat	GSM399822	Public	on	Aug	01	20	Apr	28	20	SRA	1	NAFLD_ear	Homo	sapi
Liver	pat	GSM399822	Public	on	Aug	01	20	Apr	28	20	SRA	1	NAFLD_mod	Homo	sapi
Liver	pat	GSM399823	Public	on	Aug	01	20	Apr	28	20	SRA	1	NAFLD_mod	Homo	sapi
Liver	pat	GSM399823	Public	on	Aug	01	20	Apr	28	20	SRA	1	NAFLD_ear	Homo	sapi
Liver	pat	GSM399823	Public	on	Aug	01	20	Apr	28	20	SRA	1	NAFLD_ear	Homo	sapi
Liver	pat	GSM399823	Public	on	Aug	01	20	Apr	28	20	SRA	1	NAFLD_mod	Homo	sapi
Liver	pat	GSM399823	Public	on	Aug	01	20	Apr	28	20	SRA	1	NAFLD_ear	Homo	sapi
Liver	pat	GSM399823	Public	on	Aug	01	20	Apr	28	20	SRA	1	NAFLD_ear	Homo	sapi
Liver	pat	GSM399823	Public	on	Aug	01	20	Apr	28	20	SRA	1	NAFLD_ear	Homo	sapi
Liver	pat	GSM399823	Public	on	Aug	01	20	Apr	28	20	SRA	1	NAFLD_ear	Homo	sapi
Liver	pat	GSM399823	Public	on	Aug	01	20	Apr	28	20	SRA	1	NAFLD_ear	Homo	sapi
Liver	pat	GSM399823	Public	on	Aug	01	20	Apr	28	20	SRA	1	NAFLD_mod	Homo	sapi
Liver	pat	GSM399824	Public	on	Aug	01	20	Apr	28	20	SRA	1	NAFLD_ear	Homo	sapi
Liver	pat	GSM399824	Public	on	Aug	01	20	Apr	28	20	SRA	1	NAFLD_mod	Homo	sapi
Liver	pat	GSM399824	Public	on	Aug	01	20	Apr	28	20	SRA	1	NAFLD_mod	Homo	sapi
Liver	pat	GSM399824	Public	on	Aug	01	20	Apr	28	20	SRA	1	NAFLD_ear	Homo	sapi
Liver	pat	GSM399824	Public	on	Aug	01	20	Apr	28	20	SRA	1	NAFLD_ear	Homo	sapi
Liver	pat	GSM399824	Public	on	Aug	01	20	Apr	28	20	SRA	1	NAFLD_ear	Homo	sapi
Liver	pat	GSM399824	Public	on	Aug	01	20	Apr	28	20	SRA	1	NAFLD_ear	Homo	sapi
Liver	pat	GSM399824	Public	on	Aug	01	20	Apr	28	20	SRA	1	NAFLD_ear	Homo	sapi
Liver	pat	GSM399824	Public	on	Aug	01	20	Apr	28	20	SRA	1	NAFLD_ear	Homo	sapi
Liver	pat	GSM399824	Public	on	Aug	01	20	Apr	28	20	SRA	1	NAFLD_ear	Homo	sapi
Liver	pat	GSM399825	Public	on	Aug	01	20	Apr	28	20	SRA	1	NAFLD_mod	Homo	sapi
Liver	pat	GSM399825	Public	on	Aug	01	20	Apr	28	20	SRA	1	NAFLD_mod	Homo	sapi
Liver	pat	GSM399825	Public	on	Aug	01	20	Apr	28	20	SRA	1	NAFLD_ear	Homo	sapi
Liver	pat	GSM399825	Public	on	Aug	01	20	Apr	28	20	SRA	1	NAFLD_mod	Homo	sapi
Liver	pat	GSM399825	Public	on	Aug	01	20	Apr	28	20	SRA	1	NAFLD_ear	Homo	sapi
Liver	pat	GSM399825	Public	on	Aug	01	20	Apr	28	20	SRA	1	NAFLD_ear	Homo	sapi
Liver	pat	GSM399825	Public	on	Aug	01	20	Apr	28	20	SRA	1	NAFLD_ear	Homo	sapi
Liver	pat	GSM399825	Public	on	Aug	01	20	Apr	28	20	SRA	1	NAFLD_ear	Homo	sapi
Liver	pat	GSM399825	Public	on	Aug	01	20	Apr	28	20	SRA	1	NAFLD_mod	Homo	sapi
Liver	pat	GSM399825	Public	on	Aug	01	20	Apr	28	20	SRA	1	NAFLD_ear	Homo	sapi
Liver	pat	GSM399826	Public	on	Aug	01	20	Apr	28	20	SRA	1	NAFLD_ear	Homo	sapi
Liver	pat	GSM399826	Public	on	Aug	01	20	Apr	28	20	SRA	1	NAFLD_mod	Homo	sapi
Liver	pat	GSM399826	Public	on	Aug	01	20	Apr	28	20	SRA	1	NAFLD_mod	Homo	sapi
Liver	pat	GSM399826	Public	on	Aug	01	20	Apr	28	20	SRA	1	NAFLD_ear	Homo	sapi
Liver	pat	GSM399826	Public	on	Aug	01	20	Apr	28	20	SRA	1	NAFLD_ear	Homo	sapi
Liver	pat	GSM399826	Public	on	Aug	01	20	Apr	28	20	SRA	1	NAFLD_mod	Homo	sapi
Liver	pat	GSM399826	Public	on	Aug	01	20	Apr	28	20	SRA	1	NAFLD_ear	Homo	sapi
Liver	pat	GSM399826	Public	on	Aug	01	20	Apr	28	20	SRA	1	NAFLD_ear	Homo	sapi
Liver	pat	GSM399826	Public	on	Aug	01	20	Apr	28	20	SRA	1	NAFLD_mod	Homo	sapi
Liver	pat	GSM399826	Public	on	Aug	01	20	Apr	28	20	SRA	1	NAFLD_ear	Homo	sapi
Liver	pat	GSM399827	Public	on	Aug	01	20	Apr	28	20	SRA	1	NAFLD_ear	Homo	sapi
Liver	pat	GSM399827	Public	on	Aug	01	20	Apr	28	20	SRA	1	NAFLD_ear	Homo	sapi
Liver	pat	GSM399827	Public	on	Aug	01	20	Apr	28	20	SRA	1	NAFLD_ear	Homo	sapi
Liver	pat	GSM399827	Public	on	Aug	01	20	Apr	28	20	SRA	1	NAFLD_ear	Homo	sapi
Liver	pat	GSM399827	Public	on	Aug	01	20	Apr	28	20	SRA	1	NAFLD_mod	Homo	sapi
Liver	pat	GSM399827	Public	on	Aug	01	20	Apr	28	20	SRA	1	NAFLD_mod	Homo	sapi
Liver	pat	GSM399827	Public	on	Aug	01	20	Apr	28	20	SRA	1	NAFLD_ear	Homo	sapi
Liver	pat	GSM399827	Public	on	Aug	01	20	Apr	28	20	SRA	1	NAFLD_ear	Homo	sapi
Liver	pat	GSM399827	Public	on	Aug	01	20	Apr	28	20	SRA	1	NAFLD_ear	Homo	sapi
Liver	pat	GSM399827	Public	on	Aug	01	20	Apr	28	20	SRA	1	NAFLD_ear	Homo	sapi
Liver	pat	GSM399828	Public	on	Aug	01	20	Apr	28	20	SRA	1	NAFLD_mod	Homo	sapi

For Peer Review

Liver pat	GSM399828	Public on Aug 01 20	Apr 28 20	SRA	1	NAFLD_ear	Homo sapi
Liver pat	GSM399828	Public on Aug 01 20	Apr 28 20	SRA	1	NAFLD_mod	Homo sapi
Liver pat	GSM399828	Public on Aug 01 20	Apr 28 20	SRA	1	NAFLD_ear	Homo sapi
Liver pat	GSM399828	Public on Aug 01 20	Apr 28 20	SRA	1	NAFLD_ear	Homo sapi
Liver pat	GSM399828	Public on Aug 01 20	Apr 28 20	SRA	1	NAFLD_mod	Homo sapi
Liver pat	GSM399828	Public on Aug 01 20	Apr 28 20	SRA	1	NAFLD_ear	Homo sapi
Liver pat	GSM399828	Public on Aug 01 20	Apr 28 20	SRA	1	NAFLD_ear	Homo sapi
Liver pat	GSM399828	Public on Aug 01 20	Apr 28 20	SRA	1	NAFLD_ear	Homo sapi
Liver pat	GSM399828	Public on Aug 01 20	Apr 28 20	SRA	1	NAFLD_ear	Homo sapi
Liver pat	GSM399829	Public on Aug 01 20	Apr 28 20	SRA	1	NAFLD_ear	Homo sapi
Liver pat	GSM399829	Public on Aug 01 20	Apr 28 20	SRA	1	NAFLD_ear	Homo sapi
Liver pat	GSM399829	Public on Aug 01 20	Apr 28 20	SRA	1	NAFLD_ear	Homo sapi
Liver pat	GSM399829	Public on Aug 01 20	Apr 28 20	SRA	1	NAFLD_ear	Homo sapi
Liver pat	GSM399829	Public on Aug 01 20	Apr 28 20	SRA	1	NAFLD_ear	Homo sapi
Liver pat	GSM399829	Public on Aug 01 20	Apr 28 20	SRA	1	NAFLD_ear	Homo sapi
Liver pat	GSM399829	Public on Aug 01 20	Apr 28 20	SRA	1	NAFLD_ear	Homo sapi
Liver pat	GSM399829	Public on Aug 01 20	Apr 28 20	SRA	1	NAFLD_ear	Homo sapi
Liver pat	GSM399829	Public on Aug 01 20	Apr 28 20	SRA	1	NAFLD_mod	Homo sapi
Liver pat	GSM399829	Public on Aug 01 20	Apr 28 20	SRA	1	NAFLD_ear	Homo sapi
Liver pat	GSM399830	Public on Aug 01 20	Apr 28 20	SRA	1	NAFLD_ear	Homo sapi
Liver pat	GSM399830	Public on Aug 01 20	Apr 28 20	SRA	1	NAFLD_mod	Homo sapi
Liver pat	GSM399830	Public on Aug 01 20	Apr 28 20	SRA	1	NAFLD_ear	Homo sapi
Liver pat	GSM399830	Public on Aug 01 20	Apr 28 20	SRA	1	NAFLD_ear	Homo sapi
Liver pat	GSM399830	Public on Aug 01 20	Apr 28 20	SRA	1	NAFLD_ear	Homo sapi
Liver pat	GSM399830	Public on Aug 01 20	Apr 28 20	SRA	1	NAFLD_ear	Homo sapi
Liver pat	GSM399830	Public on Aug 01 20	Apr 28 20	SRA	1	NAFLD_ear	Homo sapi
Liver pat	GSM399830	Public on Aug 01 20	Apr 28 20	SRA	1	NAFLD_ear	Homo sapi
Liver pat	GSM399830	Public on Aug 01 20	Apr 28 20	SRA	1	NAFLD_mod	Homo sapi
Liver pat	GSM399830	Public on Aug 01 20	Apr 28 20	SRA	1	NAFLD_mod	Homo sapi
Liver pat	GSM399831	Public on Aug 01 20	Apr 28 20	SRA	1	NAFLD_ear	Homo sapi
Liver pat	GSM399831	Public on Aug 01 20	Apr 28 20	SRA	1	NAFLD_ear	Homo sapi
Liver pat	GSM399831	Public on Aug 01 20	Apr 28 20	SRA	1	NAFLD_ear	Homo sapi
Liver pat	GSM399831	Public on Aug 01 20	Apr 28 20	SRA	1	NAFLD_ear	Homo sapi
Liver pat	GSM399831	Public on Aug 01 20	Apr 28 20	SRA	1	NAFLD_mod	Homo sapi
Liver pat	GSM399831	Public on Aug 01 20	Apr 28 20	SRA	1	NAFLD_ear	Homo sapi
Liver pat	GSM399831	Public on Aug 01 20	Apr 28 20	SRA	1	NAFLD_ear	Homo sapi
Liver pat	GSM399831	Public on Aug 01 20	Apr 28 20	SRA	1	NAFLD_ear	Homo sapi
Liver pat	GSM399831	Public on Aug 01 20	Apr 28 20	SRA	1	NAFLD_ear	Homo sapi
Liver pat	GSM399831	Public on Aug 01 20	Apr 28 20	SRA	1	NAFLD_mod	Homo sapi
Liver pat	GSM399832	Public on Aug 01 20	Apr 28 20	SRA	1	NAFLD_ear	Homo sapi
Liver pat	GSM399832	Public on Aug 01 20	Apr 28 20	SRA	1	NAFLD_ear	Homo sapi
Liver pat	GSM399832	Public on Aug 01 20	Apr 28 20	SRA	1	NAFLD_mod	Homo sapi
Liver pat	GSM399832	Public on Aug 01 20	Apr 28 20	SRA	1	NAFLD_ear	Homo sapi
Liver pat	GSM399832	Public on Aug 01 20	Apr 28 20	SRA	1	NAFLD_mod	Homo sapi
Liver pat	GSM399832	Public on Aug 01 20	Apr 28 20	SRA	1	NAFLD_ear	Homo sapi
Liver pat	GSM399832	Public on Aug 01 20	Apr 28 20	SRA	1	NAFLD_ear	Homo sapi
Liver pat	GSM399832	Public on Aug 01 20	Apr 28 20	SRA	1	NAFLD_mod	Homo sapi
Liver pat	GSM399832	Public on Aug 01 20	Apr 28 20	SRA	1	NAFLD_mod	Homo sapi
Liver pat	GSM399832	Public on Aug 01 20	Apr 28 20	SRA	1	NAFLD_mod	Homo sapi
Liver pat	GSM399833	Public on Aug 01 20	Apr 28 20	SRA	1	NAFLD_mod	Homo sapi
Liver pat	GSM399833	Public on Aug 01 20	Apr 28 20	SRA	1	NAFLD_ear	Homo sapi
Liver pat	GSM399833	Public on Aug 01 20	Apr 28 20	SRA	1	NAFLD_mod	Homo sapi
Liver pat	GSM399833	Public on Aug 01 20	Apr 28 20	SRA	1	NAFLD_mod	Homo sapi
Liver pat	GSM399833	Public on Aug 01 20	Apr 28 20	SRA	1	NAFLD_mod	Homo sapi
Liver pat	GSM399833	Public on Aug 01 20	Apr 28 20	SRA	1	NAFLD_ear	Homo sapi
Liver pat	GSM399833	Public on Aug 01 20	Apr 28 20	SRA	1	NAFLD_ear	Homo sapi
Liver pat	GSM399833	Public on Aug 01 20	Apr 28 20	SRA	1	NAFLD_ear	Homo sapi
Liver pat	GSM399833	Public on Aug 01 20	Apr 28 20	SRA	1	NAFLD_mod	Homo sapi

For Peer Review

Liver pat	GSM399833	Public on	Aug 01 20	Apr 28 20	SRA	1	NAFLD_ear	Homo sapi
Liver pat	GSM399834	Public on	Aug 01 20	Apr 28 20	SRA	1	NAFLD_mod	Homo sapi
Liver con	GSM399834	Public on	Aug 01 20	Apr 28 20	SRA	1	Control_c	Homo sapi
Liver con	GSM399834	Public on	Aug 01 20	Apr 28 20	SRA	1	Control_c	Homo sapi
Liver pat	GSM399834	Public on	Aug 01 20	Apr 28 20	SRA	1	NAFLD_ear	Homo sapi
Liver pat	GSM399834	Public on	Aug 01 20	Apr 28 20	SRA	1	NAFLD_ear	Homo sapi
Liver pat	GSM399834	Public on	Aug 01 20	Apr 28 20	SRA	1	NAFLD_ear	Homo sapi
Liver pat	GSM399834	Public on	Aug 01 20	Apr 28 20	SRA	1	NAFLD_ear	Homo sapi
Liver pat	GSM399834	Public on	Aug 01 20	Apr 28 20	SRA	1	NAFLD_ear	Homo sapi
Liver pat	GSM399834	Public on	Aug 01 20	Apr 28 20	SRA	1	NAFLD_ear	Homo sapi
Liver pat	GSM399834	Public on	Aug 01 20	Apr 28 20	SRA	1	NAFLD_ear	Homo sapi
Liver pat	GSM399835	Public on	Aug 01 20	Apr 28 20	SRA	1	NAFLD_ear	Homo sapi
Liver pat	GSM399835	Public on	Aug 01 20	Apr 28 20	SRA	1	NAFLD_ear	Homo sapi
Liver pat	GSM399835	Public on	Aug 01 20	Apr 28 20	SRA	1	NAFLD_ear	Homo sapi
Liver pat	GSM399835	Public on	Aug 01 20	Apr 28 20	SRA	1	NAFLD_ear	Homo sapi
Liver pat	GSM399835	Public on	Aug 01 20	Apr 28 20	SRA	1	NAFLD_mod	Homo sapi
Liver pat	GSM399835	Public on	Aug 01 20	Apr 28 20	SRA	1	NAFLD_ear	Homo sapi
Liver pat	GSM399835	Public on	Aug 01 20	Apr 28 20	SRA	1	NAFLD_ear	Homo sapi
Liver pat	GSM399835	Public on	Aug 01 20	Apr 28 20	SRA	1	NAFLD_ear	Homo sapi
Liver pat	GSM399835	Public on	Aug 01 20	Apr 28 20	SRA	1	NAFLD_ear	Homo sapi
Liver pat	GSM399835	Public on	Aug 01 20	Apr 28 20	SRA	1	NAFLD_mod	Homo sapi
Liver pat	GSM399836	Public on	Aug 01 20	Apr 28 20	SRA	1	NAFLD_ear	Homo sapi
Liver pat	GSM399836	Public on	Aug 01 20	Apr 28 20	SRA	1	NAFLD_ear	Homo sapi
Liver pat	GSM399836	Public on	Aug 01 20	Apr 28 20	SRA	1	NAFLD_ear	Homo sapi
Liver pat	GSM399836	Public on	Aug 01 20	Apr 28 20	SRA	1	NAFLD_ear	Homo sapi
Liver pat	GSM399836	Public on	Aug 01 20	Apr 28 20	SRA	1	NAFLD_mod	Homo sapi
Liver pat	GSM399836	Public on	Aug 01 20	Apr 28 20	SRA	1	NAFLD_mod	Homo sapi
Liver pat	GSM399836	Public on	Aug 01 20	Apr 28 20	SRA	1	NAFLD_ear	Homo sapi
Liver pat	GSM399836	Public on	Aug 01 20	Apr 28 20	SRA	1	NAFLD_ear	Homo sapi
Liver pat	GSM399836	Public on	Aug 01 20	Apr 28 20	SRA	1	NAFLD_ear	Homo sapi
Liver pat	GSM399836	Public on	Aug 01 20	Apr 28 20	SRA	1	NAFLD_mod	Homo sapi
Liver pat	GSM399837	Public on	Aug 01 20	Apr 28 20	SRA	1	NAFLD_ear	Homo sapi
Liver pat	GSM399837	Public on	Aug 01 20	Apr 28 20	SRA	1	NAFLD_ear	Homo sapi
Liver pat	GSM399837	Public on	Aug 01 20	Apr 28 20	SRA	1	NAFLD_mod	Homo sapi
Liver pat	GSM399837	Public on	Aug 01 20	Apr 28 20	SRA	1	NAFLD_mod	Homo sapi
Liver pat	GSM399837	Public on	Aug 01 20	Apr 28 20	SRA	1	NAFLD_mod	Homo sapi
Liver pat	GSM399837	Public on	Aug 01 20	Apr 28 20	SRA	1	NAFLD_ear	Homo sapi
Liver pat	GSM399837	Public on	Aug 01 20	Apr 28 20	SRA	1	NAFLD_ear	Homo sapi
Liver pat	GSM399837	Public on	Aug 01 20	Apr 28 20	SRA	1	NAFLD_ear	Homo sapi
Liver pat	GSM399837	Public on	Aug 01 20	Apr 28 20	SRA	1	NAFLD_mod	Homo sapi
Liver pat	GSM399837	Public on	Aug 01 20	Apr 28 20	SRA	1	NAFLD_mod	Homo sapi
Liver pat	GSM399838	Public on	Aug 01 20	Apr 28 20	SRA	1	NAFLD_ear	Homo sapi
Liver pat	GSM399838	Public on	Aug 01 20	Apr 28 20	SRA	1	NAFLD_ear	Homo sapi
Liver pat	GSM399838	Public on	Aug 01 20	Apr 28 20	SRA	1	NAFLD_mod	Homo sapi

For Peer Review

!Sample_c	!Sample_c	!Sample_c	!Sample_c	!Sample_c	!Sample_m	!Sample_e	!Sample_e	!Sample_t
nas score	fibrosis	group in	disease:	Stage: ea	total RNA	Frozen ti	RNA libra	9606
nas score	fibrosis	group in	disease:	Stage: mo	total RNA	Frozen ti	RNA libra	9606
nas score	fibrosis	group in	disease:	Stage: ea	total RNA	Frozen ti	RNA libra	9606
nas score	fibrosis	group in	disease:	Stage: mo	total RNA	Frozen ti	RNA libra	9606
nas score	fibrosis	group in	disease:	Stage: ea	total RNA	Frozen ti	RNA libra	9606
nas score	fibrosis	group in	disease:	Stage: ea	total RNA	Frozen ti	RNA libra	9606
nas score	fibrosis	group in	disease:	Stage: mo	total RNA	Frozen ti	RNA libra	9606
nas score	fibrosis	group in	disease:	Stage: ea	total RNA	Frozen ti	RNA libra	9606
nas score	fibrosis	group in	disease:	Stage: ea	total RNA	Frozen ti	RNA libra	9606
nas score	fibrosis	group in	disease:	Stage: ea	total RNA	Frozen ti	RNA libra	9606
nas score	fibrosis	group in	disease:	Stage: ea	total RNA	Frozen ti	RNA libra	9606
nas score	fibrosis	group in	disease:	Stage: ea	total RNA	Frozen ti	RNA libra	9606
nas score	fibrosis	group in	disease:	Stage: mo	total RNA	Frozen ti	RNA libra	9606
nas score	fibrosis	group in	disease:	Stage: ea	total RNA	Frozen ti	RNA libra	9606
nas score	fibrosis	group in	disease:	Stage: ea	total RNA	Frozen ti	RNA libra	9606
nas score	fibrosis	group in	disease:	Stage: ea	total RNA	Frozen ti	RNA libra	9606
nas score	fibrosis	group in	disease:	Stage: ea	total RNA	Frozen ti	RNA libra	9606
nas score	fibrosis	group in	disease:	Stage: ea	total RNA	Frozen ti	RNA libra	9606
nas score	fibrosis	group in	disease:	Stage: mo	total RNA	Frozen ti	RNA libra	9606
nas score	fibrosis	group in	disease:	Stage: ea	total RNA	Frozen ti	RNA libra	9606
nas score	fibrosis	group in	disease:	Stage: ea	total RNA	Frozen ti	RNA libra	9606
nas score	fibrosis	group in	disease:	Stage: mo	total RNA	Frozen ti	RNA libra	9606
nas score	fibrosis	group in	disease:	Stage: ea	total RNA	Frozen ti	RNA libra	9606
nas score	fibrosis	group in	disease:	Stage: mo	total RNA	Frozen ti	RNA libra	9606
nas score	fibrosis	group in	disease:	Stage: mo	total RNA	Frozen ti	RNA libra	9606
nas score	fibrosis	group in	disease:	Stage: ea	total RNA	Frozen ti	RNA libra	9606
nas score	fibrosis	group in	disease:	Stage: mo	total RNA	Frozen ti	RNA libra	9606
nas score	fibrosis	group in	disease:	Stage: ea	total RNA	Frozen ti	RNA libra	9606
nas score	fibrosis	group in	disease:	Stage: mo	total RNA	Frozen ti	RNA libra	9606
nas score	fibrosis	group in	disease:	Stage: ea	total RNA	Frozen ti	RNA libra	9606
nas score	fibrosis	group in	disease:	Stage: ea	total RNA	Frozen ti	RNA libra	9606
nas score	fibrosis	group in	disease:	Stage: ea	total RNA	Frozen ti	RNA libra	9606
nas score	fibrosis	group in	disease:	Stage: ea	total RNA	Frozen ti	RNA libra	9606
nas score	fibrosis	group in	disease:	Stage: mo	total RNA	Frozen ti	RNA libra	9606
nas score	fibrosis	group in	disease:	Stage: mo	total RNA	Frozen ti	RNA libra	9606
nas score	fibrosis	group in	disease:	Stage: ea	total RNA	Frozen ti	RNA libra	9606
nas score	fibrosis	group in	disease:	Stage: mo	total RNA	Frozen ti	RNA libra	9606
nas score	fibrosis	group in	disease:	Stage: ea	total RNA	Frozen ti	RNA libra	9606
nas score	fibrosis	group in	disease:	Stage: ea	total RNA	Frozen ti	RNA libra	9606
nas score	fibrosis	group in	disease:	Stage: ea	total RNA	Frozen ti	RNA libra	9606
nas score	fibrosis	group in	disease:	Stage: mo	total RNA	Frozen ti	RNA libra	9606
nas score	fibrosis	group in	disease:	Stage: mo	total RNA	Frozen ti	RNA libra	9606
nas score	fibrosis	group in	disease:	Stage: mo	total RNA	Frozen ti	RNA libra	9606
nas score	fibrosis	group in	disease:	Stage: mo	total RNA	Frozen ti	RNA libra	9606
nas score	fibrosis	group in	disease:	Stage: ea	total RNA	Frozen ti	RNA libra	9606
nas score	fibrosis	group in	disease:	Stage: ea	total RNA	Frozen ti	RNA libra	9606
nas score	fibrosis	group in	disease:	Stage: ea	total RNA	Frozen ti	RNA libra	9606
nas score	fibrosis	group in	disease:	Stage: mo	total RNA	Frozen ti	RNA libra	9606
nas score	fibrosis	group in	disease:	Stage: ea	total RNA	Frozen ti	RNA libra	9606
nas score	fibrosis	group in	disease:	Stage: co	total RNA	Frozen ti	RNA libra	9606
nas score	fibrosis	group in	disease:	Stage: co	total RNA	Frozen ti	RNA libra	9606
nas score	fibrosis	group in	disease:	Stage: co	total RNA	Frozen ti	RNA libra	9606
nas score	fibrosis	group in	disease:	Stage: ea	total RNA	Frozen ti	RNA libra	9606
nas score	fibrosis	group in	disease:	Stage: co	total RNA	Frozen ti	RNA libra	9606
nas score	fibrosis	group in	disease:	Stage: co	total RNA	Frozen ti	RNA libra	9606
nas score	fibrosis	group in	disease:	Stage: co	total RNA	Frozen ti	RNA libra	9606

For Peer Review

nas score	fibrosis group	in disease:	Stage: co	total RNA	Frozen ti	RNA libra	9606
nas score	fibrosis group	in disease:	Stage: co	total RNA	Frozen ti	RNA libra	9606
nas score	fibrosis group	in disease:	Stage: mo	total RNA	Frozen ti	RNA libra	9606
nas score	fibrosis group	in disease:	Stage: mo	total RNA	Frozen ti	RNA libra	9606
nas score	fibrosis group	in disease:	Stage: mo	total RNA	Frozen ti	RNA libra	9606
nas score	fibrosis group	in disease:	Stage: ea	total RNA	Frozen ti	RNA libra	9606
nas score	fibrosis group	in disease:	Stage: mo	total RNA	Frozen ti	RNA libra	9606
nas score	fibrosis group	in disease:	Stage: mo	total RNA	Frozen ti	RNA libra	9606
nas score	fibrosis group	in disease:	Stage: ea	total RNA	Frozen ti	RNA libra	9606
nas score	fibrosis group	in disease:	Stage: ea	total RNA	Frozen ti	RNA libra	9606
nas score	fibrosis group	in disease:	Stage: mo	total RNA	Frozen ti	RNA libra	9606
nas score	fibrosis group	in disease:	Stage: ea	total RNA	Frozen ti	RNA libra	9606
nas score	fibrosis group	in disease:	Stage: ea	total RNA	Frozen ti	RNA libra	9606
nas score	fibrosis group	in disease:	Stage: ea	total RNA	Frozen ti	RNA libra	9606
nas score	fibrosis group	in disease:	Stage: ea	total RNA	Frozen ti	RNA libra	9606
nas score	fibrosis group	in disease:	Stage: ea	total RNA	Frozen ti	RNA libra	9606
nas score	fibrosis group	in disease:	Stage: mo	total RNA	Frozen ti	RNA libra	9606
nas score	fibrosis group	in disease:	Stage: ea	total RNA	Frozen ti	RNA libra	9606
nas score	fibrosis group	in disease:	Stage: mo	total RNA	Frozen ti	RNA libra	9606
nas score	fibrosis group	in disease:	Stage: mo	total RNA	Frozen ti	RNA libra	9606
nas score	fibrosis group	in disease:	Stage: ea	total RNA	Frozen ti	RNA libra	9606
nas score	fibrosis group	in disease:	Stage: ea	total RNA	Frozen ti	RNA libra	9606
nas score	fibrosis group	in disease:	Stage: ea	total RNA	Frozen ti	RNA libra	9606
nas score	fibrosis group	in disease:	Stage: ea	total RNA	Frozen ti	RNA libra	9606
nas score	fibrosis group	in disease:	Stage: ea	total RNA	Frozen ti	RNA libra	9606
nas score	fibrosis group	in disease:	Stage: ea	total RNA	Frozen ti	RNA libra	9606
nas score	fibrosis group	in disease:	Stage: ea	total RNA	Frozen ti	RNA libra	9606
nas score	fibrosis group	in disease:	Stage: mo	total RNA	Frozen ti	RNA libra	9606
nas score	fibrosis group	in disease:	Stage: mo	total RNA	Frozen ti	RNA libra	9606
nas score	fibrosis group	in disease:	Stage: ea	total RNA	Frozen ti	RNA libra	9606
nas score	fibrosis group	in disease:	Stage: mo	total RNA	Frozen ti	RNA libra	9606
nas score	fibrosis group	in disease:	Stage: ea	total RNA	Frozen ti	RNA libra	9606
nas score	fibrosis group	in disease:	Stage: ea	total RNA	Frozen ti	RNA libra	9606
nas score	fibrosis group	in disease:	Stage: ea	total RNA	Frozen ti	RNA libra	9606
nas score	fibrosis group	in disease:	Stage: ea	total RNA	Frozen ti	RNA libra	9606
nas score	fibrosis group	in disease:	Stage: mo	total RNA	Frozen ti	RNA libra	9606
nas score	fibrosis group	in disease:	Stage: ea	total RNA	Frozen ti	RNA libra	9606
nas score	fibrosis group	in disease:	Stage: ea	total RNA	Frozen ti	RNA libra	9606
nas score	fibrosis group	in disease:	Stage: mo	total RNA	Frozen ti	RNA libra	9606
nas score	fibrosis group	in disease:	Stage: mo	total RNA	Frozen ti	RNA libra	9606
nas score	fibrosis group	in disease:	Stage: ea	total RNA	Frozen ti	RNA libra	9606
nas score	fibrosis group	in disease:	Stage: ea	total RNA	Frozen ti	RNA libra	9606
nas score	fibrosis group	in disease:	Stage: mo	total RNA	Frozen ti	RNA libra	9606
nas score	fibrosis group	in disease:	Stage: ea	total RNA	Frozen ti	RNA libra	9606
nas score	fibrosis group	in disease:	Stage: ea	total RNA	Frozen ti	RNA libra	9606
nas score	fibrosis group	in disease:	Stage: mo	total RNA	Frozen ti	RNA libra	9606
nas score	fibrosis group	in disease:	Stage: ea	total RNA	Frozen ti	RNA libra	9606
nas score	fibrosis group	in disease:	Stage: ea	total RNA	Frozen ti	RNA libra	9606
nas score	fibrosis group	in disease:	Stage: ea	total RNA	Frozen ti	RNA libra	9606
nas score	fibrosis group	in disease:	Stage: ea	total RNA	Frozen ti	RNA libra	9606
nas score	fibrosis group	in disease:	Stage: ea	total RNA	Frozen ti	RNA libra	9606
nas score	fibrosis group	in disease:	Stage: mo	total RNA	Frozen ti	RNA libra	9606
nas score	fibrosis group	in disease:	Stage: mo	total RNA	Frozen ti	RNA libra	9606
nas score	fibrosis group	in disease:	Stage: ea	total RNA	Frozen ti	RNA libra	9606
nas score	fibrosis group	in disease:	Stage: ea	total RNA	Frozen ti	RNA libra	9606
nas score	fibrosis group	in disease:	Stage: ea	total RNA	Frozen ti	RNA libra	9606
nas score	fibrosis group	in disease:	Stage: ea	total RNA	Frozen ti	RNA libra	9606
nas score	fibrosis group	in disease:	Stage: mo	total RNA	Frozen ti	RNA libra	9606

For Peer Review

nas score	fibrosis	group	in	disease:	Stage: ea	total	RNA	Frozen ti	RNA libra	9606
nas score	fibrosis	group	in	disease:	Stage: mo	total	RNA	Frozen ti	RNA libra	9606
nas score	fibrosis	group	in	disease:	Stage: ea	total	RNA	Frozen ti	RNA libra	9606
nas score	fibrosis	group	in	disease:	Stage: ea	total	RNA	Frozen ti	RNA libra	9606
nas score	fibrosis	group	in	disease:	Stage: mo	total	RNA	Frozen ti	RNA libra	9606
nas score	fibrosis	group	in	disease:	Stage: ea	total	RNA	Frozen ti	RNA libra	9606
nas score	fibrosis	group	in	disease:	Stage: ea	total	RNA	Frozen ti	RNA libra	9606
nas score	fibrosis	group	in	disease:	Stage: ea	total	RNA	Frozen ti	RNA libra	9606
nas score	fibrosis	group	in	disease:	Stage: ea	total	RNA	Frozen ti	RNA libra	9606
nas score	fibrosis	group	in	disease:	Stage: ea	total	RNA	Frozen ti	RNA libra	9606
nas score	fibrosis	group	in	disease:	Stage: ea	total	RNA	Frozen ti	RNA libra	9606
nas score	fibrosis	group	in	disease:	Stage: ea	total	RNA	Frozen ti	RNA libra	9606
nas score	fibrosis	group	in	disease:	Stage: ea	total	RNA	Frozen ti	RNA libra	9606
nas score	fibrosis	group	in	disease:	Stage: ea	total	RNA	Frozen ti	RNA libra	9606
nas score	fibrosis	group	in	disease:	Stage: ea	total	RNA	Frozen ti	RNA libra	9606
nas score	fibrosis	group	in	disease:	Stage: ea	total	RNA	Frozen ti	RNA libra	9606
nas score	fibrosis	group	in	disease:	Stage: ea	total	RNA	Frozen ti	RNA libra	9606
nas score	fibrosis	group	in	disease:	Stage: mo	total	RNA	Frozen ti	RNA libra	9606
nas score	fibrosis	group	in	disease:	Stage: ea	total	RNA	Frozen ti	RNA libra	9606
nas score	fibrosis	group	in	disease:	Stage: ea	total	RNA	Frozen ti	RNA libra	9606
nas score	fibrosis	group	in	disease:	Stage: mo	total	RNA	Frozen ti	RNA libra	9606
nas score	fibrosis	group	in	disease:	Stage: ea	total	RNA	Frozen ti	RNA libra	9606
nas score	fibrosis	group	in	disease:	Stage: ea	total	RNA	Frozen ti	RNA libra	9606
nas score	fibrosis	group	in	disease:	Stage: ea	total	RNA	Frozen ti	RNA libra	9606
nas score	fibrosis	group	in	disease:	Stage: ea	total	RNA	Frozen ti	RNA libra	9606
nas score	fibrosis	group	in	disease:	Stage: ea	total	RNA	Frozen ti	RNA libra	9606
nas score	fibrosis	group	in	disease:	Stage: ea	total	RNA	Frozen ti	RNA libra	9606
nas score	fibrosis	group	in	disease:	Stage: mo	total	RNA	Frozen ti	RNA libra	9606
nas score	fibrosis	group	in	disease:	Stage: mo	total	RNA	Frozen ti	RNA libra	9606
nas score	fibrosis	group	in	disease:	Stage: ea	total	RNA	Frozen ti	RNA libra	9606
nas score	fibrosis	group	in	disease:	Stage: ea	total	RNA	Frozen ti	RNA libra	9606
nas score	fibrosis	group	in	disease:	Stage: ea	total	RNA	Frozen ti	RNA libra	9606
nas score	fibrosis	group	in	disease:	Stage: ea	total	RNA	Frozen ti	RNA libra	9606
nas score	fibrosis	group	in	disease:	Stage: mo	total	RNA	Frozen ti	RNA libra	9606
nas score	fibrosis	group	in	disease:	Stage: ea	total	RNA	Frozen ti	RNA libra	9606
nas score	fibrosis	group	in	disease:	Stage: ea	total	RNA	Frozen ti	RNA libra	9606
nas score	fibrosis	group	in	disease:	Stage: ea	total	RNA	Frozen ti	RNA libra	9606
nas score	fibrosis	group	in	disease:	Stage: ea	total	RNA	Frozen ti	RNA libra	9606
nas score	fibrosis	group	in	disease:	Stage: mo	total	RNA	Frozen ti	RNA libra	9606
nas score	fibrosis	group	in	disease:	Stage: ea	total	RNA	Frozen ti	RNA libra	9606
nas score	fibrosis	group	in	disease:	Stage: ea	total	RNA	Frozen ti	RNA libra	9606
nas score	fibrosis	group	in	disease:	Stage: mo	total	RNA	Frozen ti	RNA libra	9606
nas score	fibrosis	group	in	disease:	Stage: ea	total	RNA	Frozen ti	RNA libra	9606
nas score	fibrosis	group	in	disease:	Stage: mo	total	RNA	Frozen ti	RNA libra	9606
nas score	fibrosis	group	in	disease:	Stage: ea	total	RNA	Frozen ti	RNA libra	9606
nas score	fibrosis	group	in	disease:	Stage: ea	total	RNA	Frozen ti	RNA libra	9606
nas score	fibrosis	group	in	disease:	Stage: mo	total	RNA	Frozen ti	RNA libra	9606
nas score	fibrosis	group	in	disease:	Stage: mo	total	RNA	Frozen ti	RNA libra	9606
nas score	fibrosis	group	in	disease:	Stage: mo	total	RNA	Frozen ti	RNA libra	9606
nas score	fibrosis	group	in	disease:	Stage: mo	total	RNA	Frozen ti	RNA libra	9606
nas score	fibrosis	group	in	disease:	Stage: ea	total	RNA	Frozen ti	RNA libra	9606
nas score	fibrosis	group	in	disease:	Stage: mo	total	RNA	Frozen ti	RNA libra	9606
nas score	fibrosis	group	in	disease:	Stage: mo	total	RNA	Frozen ti	RNA libra	9606
nas score	fibrosis	group	in	disease:	Stage: mo	total	RNA	Frozen ti	RNA libra	9606
nas score	fibrosis	group	in	disease:	Stage: ea	total	RNA	Frozen ti	RNA libra	9606
nas score	fibrosis	group	in	disease:	Stage: ea	total	RNA	Frozen ti	RNA libra	9606
nas score	fibrosis	group	in	disease:	Stage: ea	total	RNA	Frozen ti	RNA libra	9606
nas score	fibrosis	group	in	disease:	Stage: mo	total	RNA	Frozen ti	RNA libra	9606

For Peer Review

nas score	fibrosis	group in	disease:	Stage: ea	total RNA	Frozen ti	RNA libra	9606
nas score	fibrosis	group in	disease:	Stage: mo	total RNA	Frozen ti	RNA libra	9606
nas score	fibrosis	group in	disease:	Stage: co	total RNA	Frozen ti	RNA libra	9606
nas score	fibrosis	group in	disease:	Stage: co	total RNA	Frozen ti	RNA libra	9606
nas score	fibrosis	group in	disease:	Stage: ea	total RNA	Frozen ti	RNA libra	9606
nas score	fibrosis	group in	disease:	Stage: ea	total RNA	Frozen ti	RNA libra	9606
nas score	fibrosis	group in	disease:	Stage: ea	total RNA	Frozen ti	RNA libra	9606
nas score	fibrosis	group in	disease:	Stage: ea	total RNA	Frozen ti	RNA libra	9606
nas score	fibrosis	group in	disease:	Stage: ea	total RNA	Frozen ti	RNA libra	9606
nas score	fibrosis	group in	disease:	Stage: ea	total RNA	Frozen ti	RNA libra	9606
nas score	fibrosis	group in	disease:	Stage: ea	total RNA	Frozen ti	RNA libra	9606
nas score	fibrosis	group in	disease:	Stage: ea	total RNA	Frozen ti	RNA libra	9606
nas score	fibrosis	group in	disease:	Stage: ea	total RNA	Frozen ti	RNA libra	9606
nas score	fibrosis	group in	disease:	Stage: ea	total RNA	Frozen ti	RNA libra	9606
nas score	fibrosis	group in	disease:	Stage: ea	total RNA	Frozen ti	RNA libra	9606
nas score	fibrosis	group in	disease:	Stage: mo	total RNA	Frozen ti	RNA libra	9606
nas score	fibrosis	group in	disease:	Stage: ea	total RNA	Frozen ti	RNA libra	9606
nas score	fibrosis	group in	disease:	Stage: ea	total RNA	Frozen ti	RNA libra	9606
nas score	fibrosis	group in	disease:	Stage: ea	total RNA	Frozen ti	RNA libra	9606
nas score	fibrosis	group in	disease:	Stage: ea	total RNA	Frozen ti	RNA libra	9606
nas score	fibrosis	group in	disease:	Stage: mo	total RNA	Frozen ti	RNA libra	9606
nas score	fibrosis	group in	disease:	Stage: ea	total RNA	Frozen ti	RNA libra	9606
nas score	fibrosis	group in	disease:	Stage: ea	total RNA	Frozen ti	RNA libra	9606
nas score	fibrosis	group in	disease:	Stage: ea	total RNA	Frozen ti	RNA libra	9606
nas score	fibrosis	group in	disease:	Stage: ea	total RNA	Frozen ti	RNA libra	9606
nas score	fibrosis	group in	disease:	Stage: mo	total RNA	Frozen ti	RNA libra	9606
nas score	fibrosis	group in	disease:	Stage: mo	total RNA	Frozen ti	RNA libra	9606
nas score	fibrosis	group in	disease:	Stage: ea	total RNA	Frozen ti	RNA libra	9606
nas score	fibrosis	group in	disease:	Stage: ea	total RNA	Frozen ti	RNA libra	9606
nas score	fibrosis	group in	disease:	Stage: ea	total RNA	Frozen ti	RNA libra	9606
nas score	fibrosis	group in	disease:	Stage: mo	total RNA	Frozen ti	RNA libra	9606
nas score	fibrosis	group in	disease:	Stage: ea	total RNA	Frozen ti	RNA libra	9606
nas score	fibrosis	group in	disease:	Stage: ea	total RNA	Frozen ti	RNA libra	9606
nas score	fibrosis	group in	disease:	Stage: mo	total RNA	Frozen ti	RNA libra	9606
nas score	fibrosis	group in	disease:	Stage: mo	total RNA	Frozen ti	RNA libra	9606
nas score	fibrosis	group in	disease:	Stage: mo	total RNA	Frozen ti	RNA libra	9606
nas score	fibrosis	group in	disease:	Stage: ea	total RNA	Frozen ti	RNA libra	9606
nas score	fibrosis	group in	disease:	Stage: ea	total RNA	Frozen ti	RNA libra	9606
nas score	fibrosis	group in	disease:	Stage: ea	total RNA	Frozen ti	RNA libra	9606
nas score	fibrosis	group in	disease:	Stage: ea	total RNA	Frozen ti	RNA libra	9606
nas score	fibrosis	group in	disease:	Stage: mo	total RNA	Frozen ti	RNA libra	9606
nas score	fibrosis	group in	disease:	Stage: mo	total RNA	Frozen ti	RNA libra	9606
nas score	fibrosis	group in	disease:	Stage: ea	total RNA	Frozen ti	RNA libra	9606
nas score	fibrosis	group in	disease:	Stage: ea	total RNA	Frozen ti	RNA libra	9606
nas score	fibrosis	group in	disease:	Stage: mo	total RNA	Frozen ti	RNA libra	9606

For Peer Review

!Sample_d	!Sample_d	!Sample_d	!Sample_d	!Sample_d	!Sample_d	!Sample_p	!Sample_c	!Sample_c
017-Ann-D	Reads wer	Reads wer	Gene coun	Genome_bu	Supplemen	GPL18573	Simon, , Co	simon.coc
018-Ann-D	Reads wer	Reads wer	Gene coun	Genome_bu	Supplemen	GPL18573	Simon, , Co	simon.coc
029-Ann-D	Reads wer	Reads wer	Gene coun	Genome_bu	Supplemen	GPL18573	Simon, , Co	simon.coc
030-Ann-D	Reads wer	Reads wer	Gene coun	Genome_bu	Supplemen	GPL18573	Simon, , Co	simon.coc
031-Ann-D	Reads wer	Reads wer	Gene coun	Genome_bu	Supplemen	GPL18573	Simon, , Co	simon.coc
032-Ann-D	Reads wer	Reads wer	Gene coun	Genome_bu	Supplemen	GPL18573	Simon, , Co	simon.coc
033-Ann-D	Reads wer	Reads wer	Gene coun	Genome_bu	Supplemen	GPL18573	Simon, , Co	simon.coc
035-Ann-D	Reads wer	Reads wer	Gene coun	Genome_bu	Supplemen	GPL18573	Simon, , Co	simon.coc
036-Ann-D	Reads wer	Reads wer	Gene coun	Genome_bu	Supplemen	GPL18573	Simon, , Co	simon.coc
037-Ann-D	Reads wer	Reads wer	Gene coun	Genome_bu	Supplemen	GPL18573	Simon, , Co	simon.coc
038-Ann-D	Reads wer	Reads wer	Gene coun	Genome_bu	Supplemen	GPL18573	Simon, , Co	simon.coc
040-Ann-D	Reads wer	Reads wer	Gene coun	Genome_bu	Supplemen	GPL18573	Simon, , Co	simon.coc
041-Ann-D	Reads wer	Reads wer	Gene coun	Genome_bu	Supplemen	GPL18573	Simon, , Co	simon.coc
042-Ann-D	Reads wer	Reads wer	Gene coun	Genome_bu	Supplemen	GPL18573	Simon, , Co	simon.coc
043-Ann-D	Reads wer	Reads wer	Gene coun	Genome_bu	Supplemen	GPL18573	Simon, , Co	simon.coc
044-Ann-D	Reads wer	Reads wer	Gene coun	Genome_bu	Supplemen	GPL18573	Simon, , Co	simon.coc
046-Ann-D	Reads wer	Reads wer	Gene coun	Genome_bu	Supplemen	GPL18573	Simon, , Co	simon.coc
047-Ann-D	Reads wer	Reads wer	Gene coun	Genome_bu	Supplemen	GPL18573	Simon, , Co	simon.coc
048-Ann-D	Reads wer	Reads wer	Gene coun	Genome_bu	Supplemen	GPL18573	Simon, , Co	simon.coc
049-Ann-D	Reads wer	Reads wer	Gene coun	Genome_bu	Supplemen	GPL18573	Simon, , Co	simon.coc
051-Ann-D	Reads wer	Reads wer	Gene coun	Genome_bu	Supplemen	GPL18573	Simon, , Co	simon.coc
053-Ann-D	Reads wer	Reads wer	Gene coun	Genome_bu	Supplemen	GPL18573	Simon, , Co	simon.coc
054-Ann-D	Reads wer	Reads wer	Gene coun	Genome_bu	Supplemen	GPL18573	Simon, , Co	simon.coc
055-Ann-D	Reads wer	Reads wer	Gene coun	Genome_bu	Supplemen	GPL18573	Simon, , Co	simon.coc
059-Ann-D	Reads wer	Reads wer	Gene coun	Genome_bu	Supplemen	GPL18573	Simon, , Co	simon.coc
060-Ann-D	Reads wer	Reads wer	Gene coun	Genome_bu	Supplemen	GPL18573	Simon, , Co	simon.coc
061-Ann-D	Reads wer	Reads wer	Gene coun	Genome_bu	Supplemen	GPL18573	Simon, , Co	simon.coc
062-Ann-D	Reads wer	Reads wer	Gene coun	Genome_bu	Supplemen	GPL18573	Simon, , Co	simon.coc
075-Ann-D	Reads wer	Reads wer	Gene coun	Genome_bu	Supplemen	GPL18573	Simon, , Co	simon.coc
076-Ann-D	Reads wer	Reads wer	Gene coun	Genome_bu	Supplemen	GPL18573	Simon, , Co	simon.coc
1_20_R1.f	Reads wer	Reads wer	Gene coun	Genome_bu	Supplemen	GPL18573	Simon, , Co	simon.coc
10_352_R1	Reads wer	Reads wer	Gene coun	Genome_bu	Supplemen	GPL18573	Simon, , Co	simon.coc
101-Ann-D	Reads wer	Reads wer	Gene coun	Genome_bu	Supplemen	GPL18573	Simon, , Co	simon.coc
102-Ann-D	Reads wer	Reads wer	Gene coun	Genome_bu	Supplemen	GPL18573	Simon, , Co	simon.coc
103-Ann-D	Reads wer	Reads wer	Gene coun	Genome_bu	Supplemen	GPL18573	Simon, , Co	simon.coc
104-Ann-D	Reads wer	Reads wer	Gene coun	Genome_bu	Supplemen	GPL18573	Simon, , Co	simon.coc
105-Ann-D	Reads wer	Reads wer	Gene coun	Genome_bu	Supplemen	GPL18573	Simon, , Co	simon.coc
106-Ann-D	Reads wer	Reads wer	Gene coun	Genome_bu	Supplemen	GPL18573	Simon, , Co	simon.coc
108-Ann-D	Reads wer	Reads wer	Gene coun	Genome_bu	Supplemen	GPL18573	Simon, , Co	simon.coc
109-Ann-D	Reads wer	Reads wer	Gene coun	Genome_bu	Supplemen	GPL18573	Simon, , Co	simon.coc
110-Ann-D	Reads wer	Reads wer	Gene coun	Genome_bu	Supplemen	GPL18573	Simon, , Co	simon.coc
111-Ann-D	Reads wer	Reads wer	Gene coun	Genome_bu	Supplemen	GPL18573	Simon, , Co	simon.coc
112-Ann-D	Reads wer	Reads wer	Gene coun	Genome_bu	Supplemen	GPL18573	Simon, , Co	simon.coc
114-Ann-D	Reads wer	Reads wer	Gene coun	Genome_bu	Supplemen	GPL18573	Simon, , Co	simon.coc
115-Ann-D	Reads wer	Reads wer	Gene coun	Genome_bu	Supplemen	GPL18573	Simon, , Co	simon.coc
116-Ann-D	Reads wer	Reads wer	Gene coun	Genome_bu	Supplemen	GPL18573	Simon, , Co	simon.coc
12_469_R1	Reads wer	Reads wer	Gene coun	Genome_bu	Supplemen	GPL18573	Simon, , Co	simon.coc
120-Ann-D	Reads wer	Reads wer	Gene coun	Genome_bu	Supplemen	GPL18573	Simon, , Co	simon.coc
124-Ann-D	Reads wer	Reads wer	Gene coun	Genome_bu	Supplemen	GPL18573	Simon, , Co	simon.coc
127-Ann-D	Reads wer	Reads wer	Gene coun	Genome_bu	Supplemen	GPL18573	Simon, , Co	simon.coc
128-Ann-D	Reads wer	Reads wer	Gene coun	Genome_bu	Supplemen	GPL18573	Simon, , Co	simon.coc
129-Ann-D	Reads wer	Reads wer	Gene coun	Genome_bu	Supplemen	GPL18573	Simon, , Co	simon.coc
13_470_R1	Reads wer	Reads wer	Gene coun	Genome_bu	Supplemen	GPL18573	Simon, , Co	simon.coc
130-Ann-D	Reads wer	Reads wer	Gene coun	Genome_bu	Supplemen	GPL18573	Simon, , Co	simon.coc
132-Ann-D	Reads wer	Reads wer	Gene coun	Genome_bu	Supplemen	GPL18573	Simon, , Co	simon.coc
133-Ann-D	Reads wer	Reads wer	Gene coun	Genome_bu	Supplemen	GPL18573	Simon, , Co	simon.coc

For Peer Review

134-Ann-D	Reads wer	Reads wer	Gene coun	Genome_bu	Supplemen	GPL18573	Simon, , Co	simon.coc
135-Ann-D	Reads wer	Reads wer	Gene coun	Genome_bu	Supplemen	GPL18573	Simon, , Co	simon.coc
136-Ann-D	Reads wer	Reads wer	Gene coun	Genome_bu	Supplemen	GPL18573	Simon, , Co	simon.coc
14_472_R1	Reads wer	Reads wer	Gene coun	Genome_bu	Supplemen	GPL18573	Simon, , Co	simon.coc
141-Ann-D	Reads wer	Reads wer	Gene coun	Genome_bu	Supplemen	GPL18573	Simon, , Co	simon.coc
15_473_R1	Reads wer	Reads wer	Gene coun	Genome_bu	Supplemen	GPL18573	Simon, , Co	simon.coc
152-Ann-D	Reads wer	Reads wer	Gene coun	Genome_bu	Supplemen	GPL18573	Simon, , Co	simon.coc
155-Ann-D	Reads wer	Reads wer	Gene coun	Genome_bu	Supplemen	GPL18573	Simon, , Co	simon.coc
169-Ann-D	Reads wer	Reads wer	Gene coun	Genome_bu	Supplemen	GPL18573	Simon, , Co	simon.coc
17_82_R1.	Reads wer	Reads wer	Gene coun	Genome_bu	Supplemen	GPL18573	Simon, , Co	simon.coc
172-Ann-D	Reads wer	Reads wer	Gene coun	Genome_bu	Supplemen	GPL18573	Simon, , Co	simon.coc
176-Ann-D	Reads wer	Reads wer	Gene coun	Genome_bu	Supplemen	GPL18573	Simon, , Co	simon.coc
177-Ann-D	Reads wer	Reads wer	Gene coun	Genome_bu	Supplemen	GPL18573	Simon, , Co	simon.coc
179-Ann-D	Reads wer	Reads wer	Gene coun	Genome_bu	Supplemen	GPL18573	Simon, , Co	simon.coc
184-Ann-D	Reads wer	Reads wer	Gene coun	Genome_bu	Supplemen	GPL18573	Simon, , Co	simon.coc
185-Ann-D	Reads wer	Reads wer	Gene coun	Genome_bu	Supplemen	GPL18573	Simon, , Co	simon.coc
186-Ann-D	Reads wer	Reads wer	Gene coun	Genome_bu	Supplemen	GPL18573	Simon, , Co	simon.coc
187-Ann-D	Reads wer	Reads wer	Gene coun	Genome_bu	Supplemen	GPL18573	Simon, , Co	simon.coc
188-Ann-D	Reads wer	Reads wer	Gene coun	Genome_bu	Supplemen	GPL18573	Simon, , Co	simon.coc
19_138_R1	Reads wer	Reads wer	Gene coun	Genome_bu	Supplemen	GPL18573	Simon, , Co	simon.coc
191-Ann-D	Reads wer	Reads wer	Gene coun	Genome_bu	Supplemen	GPL18573	Simon, , Co	simon.coc
193-Ann-D	Reads wer	Reads wer	Gene coun	Genome_bu	Supplemen	GPL18573	Simon, , Co	simon.coc
197-Ann-D	Reads wer	Reads wer	Gene coun	Genome_bu	Supplemen	GPL18573	Simon, , Co	simon.coc
199-Ann-D	Reads wer	Reads wer	Gene coun	Genome_bu	Supplemen	GPL18573	Simon, , Co	simon.coc
2_23_R1.f	Reads wer	Reads wer	Gene coun	Genome_bu	Supplemen	GPL18573	Simon, , Co	simon.coc
200-Ann-D	Reads wer	Reads wer	Gene coun	Genome_bu	Supplemen	GPL18573	Simon, , Co	simon.coc
201-Ann-D	Reads wer	Reads wer	Gene coun	Genome_bu	Supplemen	GPL18573	Simon, , Co	simon.coc
202-Ann-D	Reads wer	Reads wer	Gene coun	Genome_bu	Supplemen	GPL18573	Simon, , Co	simon.coc
204-Ann-D	Reads wer	Reads wer	Gene coun	Genome_bu	Supplemen	GPL18573	Simon, , Co	simon.coc
205-Ann-D	Reads wer	Reads wer	Gene coun	Genome_bu	Supplemen	GPL18573	Simon, , Co	simon.coc
207-Ann-D	Reads wer	Reads wer	Gene coun	Genome_bu	Supplemen	GPL18573	Simon, , Co	simon.coc
208-Ann-D	Reads wer	Reads wer	Gene coun	Genome_bu	Supplemen	GPL18573	Simon, , Co	simon.coc
211-Ann-D	Reads wer	Reads wer	Gene coun	Genome_bu	Supplemen	GPL18573	Simon, , Co	simon.coc
214-Ann-D	Reads wer	Reads wer	Gene coun	Genome_bu	Supplemen	GPL18573	Simon, , Co	simon.coc
215-Ann-D	Reads wer	Reads wer	Gene coun	Genome_bu	Supplemen	GPL18573	Simon, , Co	simon.coc
216-Ann-D	Reads wer	Reads wer	Gene coun	Genome_bu	Supplemen	GPL18573	Simon, , Co	simon.coc
22_151_R1	Reads wer	Reads wer	Gene coun	Genome_bu	Supplemen	GPL18573	Simon, , Co	simon.coc
23_154_R1	Reads wer	Reads wer	Gene coun	Genome_bu	Supplemen	GPL18573	Simon, , Co	simon.coc
233-Ann-D	Reads wer	Reads wer	Gene coun	Genome_bu	Supplemen	GPL18573	Simon, , Co	simon.coc
234-Ann-D	Reads wer	Reads wer	Gene coun	Genome_bu	Supplemen	GPL18573	Simon, , Co	simon.coc
235-Ann-D	Reads wer	Reads wer	Gene coun	Genome_bu	Supplemen	GPL18573	Simon, , Co	simon.coc
236-Ann-D	Reads wer	Reads wer	Gene coun	Genome_bu	Supplemen	GPL18573	Simon, , Co	simon.coc
237-Ann-D	Reads wer	Reads wer	Gene coun	Genome_bu	Supplemen	GPL18573	Simon, , Co	simon.coc
238-Ann-D	Reads wer	Reads wer	Gene coun	Genome_bu	Supplemen	GPL18573	Simon, , Co	simon.coc
239-Ann-D	Reads wer	Reads wer	Gene coun	Genome_bu	Supplemen	GPL18573	Simon, , Co	simon.coc
24_160_R1	Reads wer	Reads wer	Gene coun	Genome_bu	Supplemen	GPL18573	Simon, , Co	simon.coc
240-Ann-D	Reads wer	Reads wer	Gene coun	Genome_bu	Supplemen	GPL18573	Simon, , Co	simon.coc
241-Ann-D	Reads wer	Reads wer	Gene coun	Genome_bu	Supplemen	GPL18573	Simon, , Co	simon.coc
242-Ann-D	Reads wer	Reads wer	Gene coun	Genome_bu	Supplemen	GPL18573	Simon, , Co	simon.coc
243-Ann-D	Reads wer	Reads wer	Gene coun	Genome_bu	Supplemen	GPL18573	Simon, , Co	simon.coc
244-Ann-D	Reads wer	Reads wer	Gene coun	Genome_bu	Supplemen	GPL18573	Simon, , Co	simon.coc
245-Ann-D	Reads wer	Reads wer	Gene coun	Genome_bu	Supplemen	GPL18573	Simon, , Co	simon.coc
246-Ann-D	Reads wer	Reads wer	Gene coun	Genome_bu	Supplemen	GPL18573	Simon, , Co	simon.coc
247-Ann-D	Reads wer	Reads wer	Gene coun	Genome_bu	Supplemen	GPL18573	Simon, , Co	simon.coc
25_164_R1	Reads wer	Reads wer	Gene coun	Genome_bu	Supplemen	GPL18573	Simon, , Co	simon.coc
252-Ann-D	Reads wer	Reads wer	Gene coun	Genome_bu	Supplemen	GPL18573	Simon, , Co	simon.coc
255-Ann-D	Reads wer	Reads wer	Gene coun	Genome_bu	Supplemen	GPL18573	Simon, , Co	simon.coc
26_171_R1	Reads wer	Reads wer	Gene coun	Genome_bu	Supplemen	GPL18573	Simon, , Co	simon.coc

For Peer Review

260-Ann-D	Reads wer	Reads wer	Gene coun	Genome_bu	Supplemen	GPL18573	Simon, , Co	simon. coc
264-Ann-D	Reads wer	Reads wer	Gene coun	Genome_bu	Supplemen	GPL18573	Simon, , Co	simon. coc
266-Ann-D	Reads wer	Reads wer	Gene coun	Genome_bu	Supplemen	GPL18573	Simon, , Co	simon. coc
268-Ann-D	Reads wer	Reads wer	Gene coun	Genome_bu	Supplemen	GPL18573	Simon, , Co	simon. coc
27_181_R1	Reads wer	Reads wer	Gene coun	Genome_bu	Supplemen	GPL18573	Simon, , Co	simon. coc
274-Ann-D	Reads wer	Reads wer	Gene coun	Genome_bu	Supplemen	GPL18573	Simon, , Co	simon. coc
277-Ann-D	Reads wer	Reads wer	Gene coun	Genome_bu	Supplemen	GPL18573	Simon, , Co	simon. coc
278-Ann-D	Reads wer	Reads wer	Gene coun	Genome_bu	Supplemen	GPL18573	Simon, , Co	simon. coc
279-Ann-D	Reads wer	Reads wer	Gene coun	Genome_bu	Supplemen	GPL18573	Simon, , Co	simon. coc
280-Ann-D	Reads wer	Reads wer	Gene coun	Genome_bu	Supplemen	GPL18573	Simon, , Co	simon. coc
281-Ann-D	Reads wer	Reads wer	Gene coun	Genome_bu	Supplemen	GPL18573	Simon, , Co	simon. coc
282-Ann-D	Reads wer	Reads wer	Gene coun	Genome_bu	Supplemen	GPL18573	Simon, , Co	simon. coc
284-Ann-D	Reads wer	Reads wer	Gene coun	Genome_bu	Supplemen	GPL18573	Simon, , Co	simon. coc
285-Ann-D	Reads wer	Reads wer	Gene coun	Genome_bu	Supplemen	GPL18573	Simon, , Co	simon. coc
286-Ann-D	Reads wer	Reads wer	Gene coun	Genome_bu	Supplemen	GPL18573	Simon, , Co	simon. coc
287-Ann-D	Reads wer	Reads wer	Gene coun	Genome_bu	Supplemen	GPL18573	Simon, , Co	simon. coc
288-Ann-D	Reads wer	Reads wer	Gene coun	Genome_bu	Supplemen	GPL18573	Simon, , Co	simon. coc
289-Ann-D	Reads wer	Reads wer	Gene coun	Genome_bu	Supplemen	GPL18573	Simon, , Co	simon. coc
29_474_R1	Reads wer	Reads wer	Gene coun	Genome_bu	Supplemen	GPL18573	Simon, , Co	simon. coc
290-Ann-D	Reads wer	Reads wer	Gene coun	Genome_bu	Supplemen	GPL18573	Simon, , Co	simon. coc
291-Ann-D	Reads wer	Reads wer	Gene coun	Genome_bu	Supplemen	GPL18573	Simon, , Co	simon. coc
293-Ann-D	Reads wer	Reads wer	Gene coun	Genome_bu	Supplemen	GPL18573	Simon, , Co	simon. coc
295-Ann-D	Reads wer	Reads wer	Gene coun	Genome_bu	Supplemen	GPL18573	Simon, , Co	simon. coc
296-Ann-D	Reads wer	Reads wer	Gene coun	Genome_bu	Supplemen	GPL18573	Simon, , Co	simon. coc
299-Ann-D	Reads wer	Reads wer	Gene coun	Genome_bu	Supplemen	GPL18573	Simon, , Co	simon. coc
3_45_R1. f	Reads wer	Reads wer	Gene coun	Genome_bu	Supplemen	GPL18573	Simon, , Co	simon. coc
300-Ann-D	Reads wer	Reads wer	Gene coun	Genome_bu	Supplemen	GPL18573	Simon, , Co	simon. coc
303-Ann-D	Reads wer	Reads wer	Gene coun	Genome_bu	Supplemen	GPL18573	Simon, , Co	simon. coc
304-Ann-D	Reads wer	Reads wer	Gene coun	Genome_bu	Supplemen	GPL18573	Simon, , Co	simon. coc
306-Ann-D	Reads wer	Reads wer	Gene coun	Genome_bu	Supplemen	GPL18573	Simon, , Co	simon. coc
31_477_R1	Reads wer	Reads wer	Gene coun	Genome_bu	Supplemen	GPL18573	Simon, , Co	simon. coc
319-Ann-D	Reads wer	Reads wer	Gene coun	Genome_bu	Supplemen	GPL18573	Simon, , Co	simon. coc
32_478_R1	Reads wer	Reads wer	Gene coun	Genome_bu	Supplemen	GPL18573	Simon, , Co	simon. coc
345-Ann-D	Reads wer	Reads wer	Gene coun	Genome_bu	Supplemen	GPL18573	Simon, , Co	simon. coc
350-Ann-D	Reads wer	Reads wer	Gene coun	Genome_bu	Supplemen	GPL18573	Simon, , Co	simon. coc
354-Ann-D	Reads wer	Reads wer	Gene coun	Genome_bu	Supplemen	GPL18573	Simon, , Co	simon. coc
355-Ann-D	Reads wer	Reads wer	Gene coun	Genome_bu	Supplemen	GPL18573	Simon, , Co	simon. coc
361-Ann-D	Reads wer	Reads wer	Gene coun	Genome_bu	Supplemen	GPL18573	Simon, , Co	simon. coc
373-Ann-D	Reads wer	Reads wer	Gene coun	Genome_bu	Supplemen	GPL18573	Simon, , Co	simon. coc
384-Ann-D	Reads wer	Reads wer	Gene coun	Genome_bu	Supplemen	GPL18573	Simon, , Co	simon. coc
4_67_R1. f	Reads wer	Reads wer	Gene coun	Genome_bu	Supplemen	GPL18573	Simon, , Co	simon. coc
402-Ann-D	Reads wer	Reads wer	Gene coun	Genome_bu	Supplemen	GPL18573	Simon, , Co	simon. coc
407-Ann-D	Reads wer	Reads wer	Gene coun	Genome_bu	Supplemen	GPL18573	Simon, , Co	simon. coc
408-Ann-D	Reads wer	Reads wer	Gene coun	Genome_bu	Supplemen	GPL18573	Simon, , Co	simon. coc
416-Ann-D	Reads wer	Reads wer	Gene coun	Genome_bu	Supplemen	GPL18573	Simon, , Co	simon. coc
418-Ann-D	Reads wer	Reads wer	Gene coun	Genome_bu	Supplemen	GPL18573	Simon, , Co	simon. coc
424-Ann-D	Reads wer	Reads wer	Gene coun	Genome_bu	Supplemen	GPL18573	Simon, , Co	simon. coc
425-Ann-D	Reads wer	Reads wer	Gene coun	Genome_bu	Supplemen	GPL18573	Simon, , Co	simon. coc
431-Ann-D	Reads wer	Reads wer	Gene coun	Genome_bu	Supplemen	GPL18573	Simon, , Co	simon. coc
443-Ann-D	Reads wer	Reads wer	Gene coun	Genome_bu	Supplemen	GPL18573	Simon, , Co	simon. coc
5_74_R1. f	Reads wer	Reads wer	Gene coun	Genome_bu	Supplemen	GPL18573	Simon, , Co	simon. coc
6_139_R1.	Reads wer	Reads wer	Gene coun	Genome_bu	Supplemen	GPL18573	Simon, , Co	simon. coc
7_162_R1.	Reads wer	Reads wer	Gene coun	Genome_bu	Supplemen	GPL18573	Simon, , Co	simon. coc
8_344_R1.	Reads wer	Reads wer	Gene coun	Genome_bu	Supplemen	GPL18573	Simon, , Co	simon. coc
84-Ann-Da	Reads wer	Reads wer	Gene coun	Genome_bu	Supplemen	GPL18573	Simon, , Co	simon. coc
85-Ann-Da	Reads wer	Reads wer	Gene coun	Genome_bu	Supplemen	GPL18573	Simon, , Co	simon. coc
9_346_R1.	Reads wer	Reads wer	Gene coun	Genome_bu	Supplemen	GPL18573	Simon, , Co	simon. coc
98-Ann-Da	Reads wer	Reads wer	Gene coun	Genome_bu	Supplemen	GPL18573	Simon, , Co	simon. coc

For Peer Review

s5_Olivie	Reads wer	Reads wer	Gene coun	Genome_bu	Supplemen	GPL18573	Simon,,Co	simon.coc
s2_Olivie	Reads wer	Reads wer	Gene coun	Genome_bu	Supplemen	GPL18573	Simon,,Co	simon.coc
078-Ann-D	Reads wer	Reads wer	Gene coun	Genome_bu	Supplemen	GPL18573	Simon,,Co	simon.coc
085-Ann-D	Reads wer	Reads wer	Gene coun	Genome_bu	Supplemen	GPL18573	Simon,,Co	simon.coc
C100-Ann-	Reads wer	Reads wer	Gene coun	Genome_bu	Supplemen	GPL18573	Simon,,Co	simon.coc
C101-Ann-	Reads wer	Reads wer	Gene coun	Genome_bu	Supplemen	GPL18573	Simon,,Co	simon.coc
C102-Ann-	Reads wer	Reads wer	Gene coun	Genome_bu	Supplemen	GPL18573	Simon,,Co	simon.coc
C103-Ann-	Reads wer	Reads wer	Gene coun	Genome_bu	Supplemen	GPL18573	Simon,,Co	simon.coc
C107-Ann-	Reads wer	Reads wer	Gene coun	Genome_bu	Supplemen	GPL18573	Simon,,Co	simon.coc
C108-Ann-	Reads wer	Reads wer	Gene coun	Genome_bu	Supplemen	GPL18573	Simon,,Co	simon.coc
C109-Ann-	Reads wer	Reads wer	Gene coun	Genome_bu	Supplemen	GPL18573	Simon,,Co	simon.coc
C110-Ann-	Reads wer	Reads wer	Gene coun	Genome_bu	Supplemen	GPL18573	Simon,,Co	simon.coc
C122-Ann-	Reads wer	Reads wer	Gene coun	Genome_bu	Supplemen	GPL18573	Simon,,Co	simon.coc
C123-Ann-	Reads wer	Reads wer	Gene coun	Genome_bu	Supplemen	GPL18573	Simon,,Co	simon.coc
C124-Ann-	Reads wer	Reads wer	Gene coun	Genome_bu	Supplemen	GPL18573	Simon,,Co	simon.coc
C125-Ann-	Reads wer	Reads wer	Gene coun	Genome_bu	Supplemen	GPL18573	Simon,,Co	simon.coc
C126-Ann-	Reads wer	Reads wer	Gene coun	Genome_bu	Supplemen	GPL18573	Simon,,Co	simon.coc
C128-Ann-	Reads wer	Reads wer	Gene coun	Genome_bu	Supplemen	GPL18573	Simon,,Co	simon.coc
C129-Ann-	Reads wer	Reads wer	Gene coun	Genome_bu	Supplemen	GPL18573	Simon,,Co	simon.coc
C131-Ann-	Reads wer	Reads wer	Gene coun	Genome_bu	Supplemen	GPL18573	Simon,,Co	simon.coc
C133-Ann-	Reads wer	Reads wer	Gene coun	Genome_bu	Supplemen	GPL18573	Simon,,Co	simon.coc
C134_S5	Reads wer	Reads wer	Gene coun	Genome_bu	Supplemen	GPL18573	Simon,,Co	simon.coc
C135-Ann-	Reads wer	Reads wer	Gene coun	Genome_bu	Supplemen	GPL18573	Simon,,Co	simon.coc
C136-Ann-	Reads wer	Reads wer	Gene coun	Genome_bu	Supplemen	GPL18573	Simon,,Co	simon.coc
C137-Ann-	Reads wer	Reads wer	Gene coun	Genome_bu	Supplemen	GPL18573	Simon,,Co	simon.coc
C139-Ann-	Reads wer	Reads wer	Gene coun	Genome_bu	Supplemen	GPL18573	Simon,,Co	simon.coc
C143-Ann-	Reads wer	Reads wer	Gene coun	Genome_bu	Supplemen	GPL18573	Simon,,Co	simon.coc
C145-Ann-	Reads wer	Reads wer	Gene coun	Genome_bu	Supplemen	GPL18573	Simon,,Co	simon.coc
C150-Ann-	Reads wer	Reads wer	Gene coun	Genome_bu	Supplemen	GPL18573	Simon,,Co	simon.coc
C171-Ann-	Reads wer	Reads wer	Gene coun	Genome_bu	Supplemen	GPL18573	Simon,,Co	simon.coc
C173-Ann-	Reads wer	Reads wer	Gene coun	Genome_bu	Supplemen	GPL18573	Simon,,Co	simon.coc
C18-Ann-D	Reads wer	Reads wer	Gene coun	Genome_bu	Supplemen	GPL18573	Simon,,Co	simon.coc
C29-Ann-D	Reads wer	Reads wer	Gene coun	Genome_bu	Supplemen	GPL18573	Simon,,Co	simon.coc
C61-Ann-D	Reads wer	Reads wer	Gene coun	Genome_bu	Supplemen	GPL18573	Simon,,Co	simon.coc
C68-Ann-D	Reads wer	Reads wer	Gene coun	Genome_bu	Supplemen	GPL18573	Simon,,Co	simon.coc
C69-Ann-D	Reads wer	Reads wer	Gene coun	Genome_bu	Supplemen	GPL18573	Simon,,Co	simon.coc
C73-Ann-D	Reads wer	Reads wer	Gene coun	Genome_bu	Supplemen	GPL18573	Simon,,Co	simon.coc
C76-Ann-D	Reads wer	Reads wer	Gene coun	Genome_bu	Supplemen	GPL18573	Simon,,Co	simon.coc
C81-Ann-D	Reads wer	Reads wer	Gene coun	Genome_bu	Supplemen	GPL18573	Simon,,Co	simon.coc
C82-Ann-D	Reads wer	Reads wer	Gene coun	Genome_bu	Supplemen	GPL18573	Simon,,Co	simon.coc
C90-Ann-D	Reads wer	Reads wer	Gene coun	Genome_bu	Supplemen	GPL18573	Simon,,Co	simon.coc
C93-Ann-D	Reads wer	Reads wer	Gene coun	Genome_bu	Supplemen	GPL18573	Simon,,Co	simon.coc
C94-Ann-D	Reads wer	Reads wer	Gene coun	Genome_bu	Supplemen	GPL18573	Simon,,Co	simon.coc
C99-Ann-D	Reads wer	Reads wer	Gene coun	Genome_bu	Supplemen	GPL18573	Simon,,Co	simon.coc

For Peer Review

!Sample_c	!Sample_c	!Sample_c	!Sample_c	!Sample_c	!Sample_d	!Sample_i	!Sample_l	!Sample_l
Newcastle	Framlingt	Newcastle	NE2 4HH	United Ki	0	Illumina	cDNA	transcrip
Newcastle	Framlingt	Newcastle	NE2 4HH	United Ki	0	Illumina	cDNA	transcrip
Newcastle	Framlingt	Newcastle	NE2 4HH	United Ki	0	Illumina	cDNA	transcrip
Newcastle	Framlingt	Newcastle	NE2 4HH	United Ki	0	Illumina	cDNA	transcrip
Newcastle	Framlingt	Newcastle	NE2 4HH	United Ki	0	Illumina	cDNA	transcrip
Newcastle	Framlingt	Newcastle	NE2 4HH	United Ki	0	Illumina	cDNA	transcrip
Newcastle	Framlingt	Newcastle	NE2 4HH	United Ki	0	Illumina	cDNA	transcrip
Newcastle	Framlingt	Newcastle	NE2 4HH	United Ki	0	Illumina	cDNA	transcrip
Newcastle	Framlingt	Newcastle	NE2 4HH	United Ki	0	Illumina	cDNA	transcrip
Newcastle	Framlingt	Newcastle	NE2 4HH	United Ki	0	Illumina	cDNA	transcrip
Newcastle	Framlingt	Newcastle	NE2 4HH	United Ki	0	Illumina	cDNA	transcrip
Newcastle	Framlingt	Newcastle	NE2 4HH	United Ki	0	Illumina	cDNA	transcrip
Newcastle	Framlingt	Newcastle	NE2 4HH	United Ki	0	Illumina	cDNA	transcrip
Newcastle	Framlingt	Newcastle	NE2 4HH	United Ki	0	Illumina	cDNA	transcrip
Newcastle	Framlingt	Newcastle	NE2 4HH	United Ki	0	Illumina	cDNA	transcrip
Newcastle	Framlingt	Newcastle	NE2 4HH	United Ki	0	Illumina	cDNA	transcrip
Newcastle	Framlingt	Newcastle	NE2 4HH	United Ki	0	Illumina	cDNA	transcrip
Newcastle	Framlingt	Newcastle	NE2 4HH	United Ki	0	Illumina	cDNA	transcrip
Newcastle	Framlingt	Newcastle	NE2 4HH	United Ki	0	Illumina	cDNA	transcrip
Newcastle	Framlingt	Newcastle	NE2 4HH	United Ki	0	Illumina	cDNA	transcrip
Newcastle	Framlingt	Newcastle	NE2 4HH	United Ki	0	Illumina	cDNA	transcrip
Newcastle	Framlingt	Newcastle	NE2 4HH	United Ki	0	Illumina	cDNA	transcrip
Newcastle	Framlingt	Newcastle	NE2 4HH	United Ki	0	Illumina	cDNA	transcrip
Newcastle	Framlingt	Newcastle	NE2 4HH	United Ki	0	Illumina	cDNA	transcrip
Newcastle	Framlingt	Newcastle	NE2 4HH	United Ki	0	Illumina	cDNA	transcrip
Newcastle	Framlingt	Newcastle	NE2 4HH	United Ki	0	Illumina	cDNA	transcrip
Newcastle	Framlingt	Newcastle	NE2 4HH	United Ki	0	Illumina	cDNA	transcrip
Newcastle	Framlingt	Newcastle	NE2 4HH	United Ki	0	Illumina	cDNA	transcrip
Newcastle	Framlingt	Newcastle	NE2 4HH	United Ki	0	Illumina	cDNA	transcrip
Newcastle	Framlingt	Newcastle	NE2 4HH	United Ki	0	Illumina	cDNA	transcrip
Newcastle	Framlingt	Newcastle	NE2 4HH	United Ki	0	Illumina	cDNA	transcrip
Newcastle	Framlingt	Newcastle	NE2 4HH	United Ki	0	Illumina	cDNA	transcrip
Newcastle	Framlingt	Newcastle	NE2 4HH	United Ki	0	Illumina	cDNA	transcrip
Newcastle	Framlingt	Newcastle	NE2 4HH	United Ki	0	Illumina	cDNA	transcrip
Newcastle	Framlingt	Newcastle	NE2 4HH	United Ki	0	Illumina	cDNA	transcrip
Newcastle	Framlingt	Newcastle	NE2 4HH	United Ki	0	Illumina	cDNA	transcrip
Newcastle	Framlingt	Newcastle	NE2 4HH	United Ki	0	Illumina	cDNA	transcrip
Newcastle	Framlingt	Newcastle	NE2 4HH	United Ki	0	Illumina	cDNA	transcrip
Newcastle	Framlingt	Newcastle	NE2 4HH	United Ki	0	Illumina	cDNA	transcrip
Newcastle	Framlingt	Newcastle	NE2 4HH	United Ki	0	Illumina	cDNA	transcrip
Newcastle	Framlingt	Newcastle	NE2 4HH	United Ki	0	Illumina	cDNA	transcrip
Newcastle	Framlingt	Newcastle	NE2 4HH	United Ki	0	Illumina	cDNA	transcrip
Newcastle	Framlingt	Newcastle	NE2 4HH	United Ki	0	Illumina	cDNA	transcrip
Newcastle	Framlingt	Newcastle	NE2 4HH	United Ki	0	Illumina	cDNA	transcrip
Newcastle	Framlingt	Newcastle	NE2 4HH	United Ki	0	Illumina	cDNA	transcrip
Newcastle	Framlingt	Newcastle	NE2 4HH	United Ki	0	Illumina	cDNA	transcrip
Newcastle	Framlingt	Newcastle	NE2 4HH	United Ki	0	Illumina	cDNA	transcrip
Newcastle	Framlingt	Newcastle	NE2 4HH	United Ki	0	Illumina	cDNA	transcrip
Newcastle	Framlingt	Newcastle	NE2 4HH	United Ki	0	Illumina	cDNA	transcrip
Newcastle	Framlingt	Newcastle	NE2 4HH	United Ki	0	Illumina	cDNA	transcrip
Newcastle	Framlingt	Newcastle	NE2 4HH	United Ki	0	Illumina	cDNA	transcrip
Newcastle	Framlingt	Newcastle	NE2 4HH	United Ki	0	Illumina	cDNA	transcrip
Newcastle	Framlingt	Newcastle	NE2 4HH	United Ki	0	Illumina	cDNA	transcrip
Newcastle	Framlingt	Newcastle	NE2 4HH	United Ki	0	Illumina	cDNA	transcrip
Newcastle	Framlingt	Newcastle	NE2 4HH	United Ki	0	Illumina	cDNA	transcrip
Newcastle	Framlingt	Newcastle	NE2 4HH	United Ki	0	Illumina	cDNA	transcrip

For Peer Review

Newcastle	Framlingt	Newcastle	NE2 4HH	United Ki	0	Illumina	cDNA	transcrip
Newcastle	Framlingt	Newcastle	NE2 4HH	United Ki	0	Illumina	cDNA	transcrip
Newcastle	Framlingt	Newcastle	NE2 4HH	United Ki	0	Illumina	cDNA	transcrip
Newcastle	Framlingt	Newcastle	NE2 4HH	United Ki	0	Illumina	cDNA	transcrip
Newcastle	Framlingt	Newcastle	NE2 4HH	United Ki	0	Illumina	cDNA	transcrip
Newcastle	Framlingt	Newcastle	NE2 4HH	United Ki	0	Illumina	cDNA	transcrip
Newcastle	Framlingt	Newcastle	NE2 4HH	United Ki	0	Illumina	cDNA	transcrip
Newcastle	Framlingt	Newcastle	NE2 4HH	United Ki	0	Illumina	cDNA	transcrip
Newcastle	Framlingt	Newcastle	NE2 4HH	United Ki	0	Illumina	cDNA	transcrip
Newcastle	Framlingt	Newcastle	NE2 4HH	United Ki	0	Illumina	cDNA	transcrip
Newcastle	Framlingt	Newcastle	NE2 4HH	United Ki	0	Illumina	cDNA	transcrip
Newcastle	Framlingt	Newcastle	NE2 4HH	United Ki	0	Illumina	cDNA	transcrip
Newcastle	Framlingt	Newcastle	NE2 4HH	United Ki	0	Illumina	cDNA	transcrip
Newcastle	Framlingt	Newcastle	NE2 4HH	United Ki	0	Illumina	cDNA	transcrip
Newcastle	Framlingt	Newcastle	NE2 4HH	United Ki	0	Illumina	cDNA	transcrip
Newcastle	Framlingt	Newcastle	NE2 4HH	United Ki	0	Illumina	cDNA	transcrip
Newcastle	Framlingt	Newcastle	NE2 4HH	United Ki	0	Illumina	cDNA	transcrip
Newcastle	Framlingt	Newcastle	NE2 4HH	United Ki	0	Illumina	cDNA	transcrip
Newcastle	Framlingt	Newcastle	NE2 4HH	United Ki	0	Illumina	cDNA	transcrip
Newcastle	Framlingt	Newcastle	NE2 4HH	United Ki	0	Illumina	cDNA	transcrip
Newcastle	Framlingt	Newcastle	NE2 4HH	United Ki	0	Illumina	cDNA	transcrip
Newcastle	Framlingt	Newcastle	NE2 4HH	United Ki	0	Illumina	cDNA	transcrip
Newcastle	Framlingt	Newcastle	NE2 4HH	United Ki	0	Illumina	cDNA	transcrip
Newcastle	Framlingt	Newcastle	NE2 4HH	United Ki	0	Illumina	cDNA	transcrip
Newcastle	Framlingt	Newcastle	NE2 4HH	United Ki	0	Illumina	cDNA	transcrip
Newcastle	Framlingt	Newcastle	NE2 4HH	United Ki	0	Illumina	cDNA	transcrip
Newcastle	Framlingt	Newcastle	NE2 4HH	United Ki	0	Illumina	cDNA	transcrip
Newcastle	Framlingt	Newcastle	NE2 4HH	United Ki	0	Illumina	cDNA	transcrip
Newcastle	Framlingt	Newcastle	NE2 4HH	United Ki	0	Illumina	cDNA	transcrip
Newcastle	Framlingt	Newcastle	NE2 4HH	United Ki	0	Illumina	cDNA	transcrip
Newcastle	Framlingt	Newcastle	NE2 4HH	United Ki	0	Illumina	cDNA	transcrip
Newcastle	Framlingt	Newcastle	NE2 4HH	United Ki	0	Illumina	cDNA	transcrip
Newcastle	Framlingt	Newcastle	NE2 4HH	United Ki	0	Illumina	cDNA	transcrip
Newcastle	Framlingt	Newcastle	NE2 4HH	United Ki	0	Illumina	cDNA	transcrip
Newcastle	Framlingt	Newcastle	NE2 4HH	United Ki	0	Illumina	cDNA	transcrip
Newcastle	Framlingt	Newcastle	NE2 4HH	United Ki	0	Illumina	cDNA	transcrip
Newcastle	Framlingt	Newcastle	NE2 4HH	United Ki	0	Illumina	cDNA	transcrip
Newcastle	Framlingt	Newcastle	NE2 4HH	United Ki	0	Illumina	cDNA	transcrip
Newcastle	Framlingt	Newcastle	NE2 4HH	United Ki	0	Illumina	cDNA	transcrip
Newcastle	Framlingt	Newcastle	NE2 4HH	United Ki	0	Illumina	cDNA	transcrip
Newcastle	Framlingt	Newcastle	NE2 4HH	United Ki	0	Illumina	cDNA	transcrip
Newcastle	Framlingt	Newcastle	NE2 4HH	United Ki	0	Illumina	cDNA	transcrip
Newcastle	Framlingt	Newcastle	NE2 4HH	United Ki	0	Illumina	cDNA	transcrip
Newcastle	Framlingt	Newcastle	NE2 4HH	United Ki	0	Illumina	cDNA	transcrip
Newcastle	Framlingt	Newcastle	NE2 4HH	United Ki	0	Illumina	cDNA	transcrip
Newcastle	Framlingt	Newcastle	NE2 4HH	United Ki	0	Illumina	cDNA	transcrip
Newcastle	Framlingt	Newcastle	NE2 4HH	United Ki	0	Illumina	cDNA	transcrip
Newcastle	Framlingt	Newcastle	NE2 4HH	United Ki	0	Illumina	cDNA	transcrip
Newcastle	Framlingt	Newcastle	NE2 4HH	United Ki	0	Illumina	cDNA	transcrip
Newcastle	Framlingt	Newcastle	NE2 4HH	United Ki	0	Illumina	cDNA	transcrip
Newcastle	Framlingt	Newcastle	NE2 4HH	United Ki	0	Illumina	cDNA	transcrip
Newcastle	Framlingt	Newcastle	NE2 4HH	United Ki	0	Illumina	cDNA	transcrip
Newcastle	Framlingt	Newcastle	NE2 4HH	United Ki	0	Illumina	cDNA	transcrip
Newcastle	Framlingt	Newcastle	NE2 4HH	United Ki	0	Illumina	cDNA	transcrip
Newcastle	Framlingt	Newcastle	NE2 4HH	United Ki	0	Illumina	cDNA	transcrip
Newcastle	Framlingt	Newcastle	NE2 4HH	United Ki	0	Illumina	cDNA	transcrip
Newcastle	Framlingt	Newcastle	NE2 4HH	United Ki	0	Illumina	cDNA	transcrip
Newcastle	Framlingt	Newcastle	NE2 4HH	United Ki	0	Illumina	cDNA	transcrip

For Peer Review

Newcastle	Framlingt	Newcastle	NE2 4HH	United Ki	0	Illumina	cDNA	transcrip
Newcastle	Framlingt	Newcastle	NE2 4HH	United Ki	0	Illumina	cDNA	transcrip
Newcastle	Framlingt	Newcastle	NE2 4HH	United Ki	0	Illumina	cDNA	transcrip
Newcastle	Framlingt	Newcastle	NE2 4HH	United Ki	0	Illumina	cDNA	transcrip
Newcastle	Framlingt	Newcastle	NE2 4HH	United Ki	0	Illumina	cDNA	transcrip
Newcastle	Framlingt	Newcastle	NE2 4HH	United Ki	0	Illumina	cDNA	transcrip
Newcastle	Framlingt	Newcastle	NE2 4HH	United Ki	0	Illumina	cDNA	transcrip
Newcastle	Framlingt	Newcastle	NE2 4HH	United Ki	0	Illumina	cDNA	transcrip
Newcastle	Framlingt	Newcastle	NE2 4HH	United Ki	0	Illumina	cDNA	transcrip
Newcastle	Framlingt	Newcastle	NE2 4HH	United Ki	0	Illumina	cDNA	transcrip
Newcastle	Framlingt	Newcastle	NE2 4HH	United Ki	0	Illumina	cDNA	transcrip
Newcastle	Framlingt	Newcastle	NE2 4HH	United Ki	0	Illumina	cDNA	transcrip
Newcastle	Framlingt	Newcastle	NE2 4HH	United Ki	0	Illumina	cDNA	transcrip
Newcastle	Framlingt	Newcastle	NE2 4HH	United Ki	0	Illumina	cDNA	transcrip
Newcastle	Framlingt	Newcastle	NE2 4HH	United Ki	0	Illumina	cDNA	transcrip
Newcastle	Framlingt	Newcastle	NE2 4HH	United Ki	0	Illumina	cDNA	transcrip
Newcastle	Framlingt	Newcastle	NE2 4HH	United Ki	0	Illumina	cDNA	transcrip
Newcastle	Framlingt	Newcastle	NE2 4HH	United Ki	0	Illumina	cDNA	transcrip
Newcastle	Framlingt	Newcastle	NE2 4HH	United Ki	0	Illumina	cDNA	transcrip
Newcastle	Framlingt	Newcastle	NE2 4HH	United Ki	0	Illumina	cDNA	transcrip
Newcastle	Framlingt	Newcastle	NE2 4HH	United Ki	0	Illumina	cDNA	transcrip
Newcastle	Framlingt	Newcastle	NE2 4HH	United Ki	0	Illumina	cDNA	transcrip
Newcastle	Framlingt	Newcastle	NE2 4HH	United Ki	0	Illumina	cDNA	transcrip
Newcastle	Framlingt	Newcastle	NE2 4HH	United Ki	0	Illumina	cDNA	transcrip
Newcastle	Framlingt	Newcastle	NE2 4HH	United Ki	0	Illumina	cDNA	transcrip
Newcastle	Framlingt	Newcastle	NE2 4HH	United Ki	0	Illumina	cDNA	transcrip
Newcastle	Framlingt	Newcastle	NE2 4HH	United Ki	0	Illumina	cDNA	transcrip
Newcastle	Framlingt	Newcastle	NE2 4HH	United Ki	0	Illumina	cDNA	transcrip
Newcastle	Framlingt	Newcastle	NE2 4HH	United Ki	0	Illumina	cDNA	transcrip
Newcastle	Framlingt	Newcastle	NE2 4HH	United Ki	0	Illumina	cDNA	transcrip
Newcastle	Framlingt	Newcastle	NE2 4HH	United Ki	0	Illumina	cDNA	transcrip
Newcastle	Framlingt	Newcastle	NE2 4HH	United Ki	0	Illumina	cDNA	transcrip
Newcastle	Framlingt	Newcastle	NE2 4HH	United Ki	0	Illumina	cDNA	transcrip
Newcastle	Framlingt	Newcastle	NE2 4HH	United Ki	0	Illumina	cDNA	transcrip
Newcastle	Framlingt	Newcastle	NE2 4HH	United Ki	0	Illumina	cDNA	transcrip
Newcastle	Framlingt	Newcastle	NE2 4HH	United Ki	0	Illumina	cDNA	transcrip
Newcastle	Framlingt	Newcastle	NE2 4HH	United Ki	0	Illumina	cDNA	transcrip
Newcastle	Framlingt	Newcastle	NE2 4HH	United Ki	0	Illumina	cDNA	transcrip
Newcastle	Framlingt	Newcastle	NE2 4HH	United Ki	0	Illumina	cDNA	transcrip
Newcastle	Framlingt	Newcastle	NE2 4HH	United Ki	0	Illumina	cDNA	transcrip
Newcastle	Framlingt	Newcastle	NE2 4HH	United Ki	0	Illumina	cDNA	transcrip
Newcastle	Framlingt	Newcastle	NE2 4HH	United Ki	0	Illumina	cDNA	transcrip
Newcastle	Framlingt	Newcastle	NE2 4HH	United Ki	0	Illumina	cDNA	transcrip
Newcastle	Framlingt	Newcastle	NE2 4HH	United Ki	0	Illumina	cDNA	transcrip
Newcastle	Framlingt	Newcastle	NE2 4HH	United Ki	0	Illumina	cDNA	transcrip
Newcastle	Framlingt	Newcastle	NE2 4HH	United Ki	0	Illumina	cDNA	transcrip
Newcastle	Framlingt	Newcastle	NE2 4HH	United Ki	0	Illumina	cDNA	transcrip
Newcastle	Framlingt	Newcastle	NE2 4HH	United Ki	0	Illumina	cDNA	transcrip
Newcastle	Framlingt	Newcastle	NE2 4HH	United Ki	0	Illumina	cDNA	transcrip
Newcastle	Framlingt	Newcastle	NE2 4HH	United Ki	0	Illumina	cDNA	transcrip
Newcastle	Framlingt	Newcastle	NE2 4HH	United Ki	0	Illumina	cDNA	transcrip
Newcastle	Framlingt	Newcastle	NE2 4HH	United Ki	0	Illumina	cDNA	transcrip
Newcastle	Framlingt	Newcastle	NE2 4HH	United Ki	0	Illumina	cDNA	transcrip
Newcastle	Framlingt	Newcastle	NE2 4HH	United Ki	0	Illumina	cDNA	transcrip
Newcastle	Framlingt	Newcastle	NE2 4HH	United Ki	0	Illumina	cDNA	transcrip
Newcastle	Framlingt	Newcastle	NE2 4HH	United Ki	0	Illumina	cDNA	transcrip
Newcastle	Framlingt	Newcastle	NE2 4HH	United Ki	0	Illumina	cDNA	transcrip
Newcastle	Framlingt	Newcastle	NE2 4HH	United Ki	0	Illumina	cDNA	transcrip

For Peer Review

Newcastle	Framlingt	Newcastle	NE2 4HH	United Ki	0	Illumina	cDNA	transcrip
Newcastle	Framlingt	Newcastle	NE2 4HH	United Ki	0	Illumina	cDNA	transcrip
Newcastle	Framlingt	Newcastle	NE2 4HH	United Ki	0	Illumina	cDNA	transcrip
Newcastle	Framlingt	Newcastle	NE2 4HH	United Ki	0	Illumina	cDNA	transcrip
Newcastle	Framlingt	Newcastle	NE2 4HH	United Ki	0	Illumina	cDNA	transcrip
Newcastle	Framlingt	Newcastle	NE2 4HH	United Ki	0	Illumina	cDNA	transcrip
Newcastle	Framlingt	Newcastle	NE2 4HH	United Ki	0	Illumina	cDNA	transcrip
Newcastle	Framlingt	Newcastle	NE2 4HH	United Ki	0	Illumina	cDNA	transcrip
Newcastle	Framlingt	Newcastle	NE2 4HH	United Ki	0	Illumina	cDNA	transcrip
Newcastle	Framlingt	Newcastle	NE2 4HH	United Ki	0	Illumina	cDNA	transcrip
Newcastle	Framlingt	Newcastle	NE2 4HH	United Ki	0	Illumina	cDNA	transcrip
Newcastle	Framlingt	Newcastle	NE2 4HH	United Ki	0	Illumina	cDNA	transcrip
Newcastle	Framlingt	Newcastle	NE2 4HH	United Ki	0	Illumina	cDNA	transcrip
Newcastle	Framlingt	Newcastle	NE2 4HH	United Ki	0	Illumina	cDNA	transcrip
Newcastle	Framlingt	Newcastle	NE2 4HH	United Ki	0	Illumina	cDNA	transcrip
Newcastle	Framlingt	Newcastle	NE2 4HH	United Ki	0	Illumina	cDNA	transcrip
Newcastle	Framlingt	Newcastle	NE2 4HH	United Ki	0	Illumina	cDNA	transcrip
Newcastle	Framlingt	Newcastle	NE2 4HH	United Ki	0	Illumina	cDNA	transcrip
Newcastle	Framlingt	Newcastle	NE2 4HH	United Ki	0	Illumina	cDNA	transcrip
Newcastle	Framlingt	Newcastle	NE2 4HH	United Ki	0	Illumina	cDNA	transcrip
Newcastle	Framlingt	Newcastle	NE2 4HH	United Ki	0	Illumina	cDNA	transcrip
Newcastle	Framlingt	Newcastle	NE2 4HH	United Ki	0	Illumina	cDNA	transcrip
Newcastle	Framlingt	Newcastle	NE2 4HH	United Ki	0	Illumina	cDNA	transcrip
Newcastle	Framlingt	Newcastle	NE2 4HH	United Ki	0	Illumina	cDNA	transcrip
Newcastle	Framlingt	Newcastle	NE2 4HH	United Ki	0	Illumina	cDNA	transcrip
Newcastle	Framlingt	Newcastle	NE2 4HH	United Ki	0	Illumina	cDNA	transcrip
Newcastle	Framlingt	Newcastle	NE2 4HH	United Ki	0	Illumina	cDNA	transcrip
Newcastle	Framlingt	Newcastle	NE2 4HH	United Ki	0	Illumina	cDNA	transcrip
Newcastle	Framlingt	Newcastle	NE2 4HH	United Ki	0	Illumina	cDNA	transcrip
Newcastle	Framlingt	Newcastle	NE2 4HH	United Ki	0	Illumina	cDNA	transcrip
Newcastle	Framlingt	Newcastle	NE2 4HH	United Ki	0	Illumina	cDNA	transcrip
Newcastle	Framlingt	Newcastle	NE2 4HH	United Ki	0	Illumina	cDNA	transcrip
Newcastle	Framlingt	Newcastle	NE2 4HH	United Ki	0	Illumina	cDNA	transcrip
Newcastle	Framlingt	Newcastle	NE2 4HH	United Ki	0	Illumina	cDNA	transcrip
Newcastle	Framlingt	Newcastle	NE2 4HH	United Ki	0	Illumina	cDNA	transcrip
Newcastle	Framlingt	Newcastle	NE2 4HH	United Ki	0	Illumina	cDNA	transcrip
Newcastle	Framlingt	Newcastle	NE2 4HH	United Ki	0	Illumina	cDNA	transcrip
Newcastle	Framlingt	Newcastle	NE2 4HH	United Ki	0	Illumina	cDNA	transcrip
Newcastle	Framlingt	Newcastle	NE2 4HH	United Ki	0	Illumina	cDNA	transcrip
Newcastle	Framlingt	Newcastle	NE2 4HH	United Ki	0	Illumina	cDNA	transcrip
Newcastle	Framlingt	Newcastle	NE2 4HH	United Ki	0	Illumina	cDNA	transcrip
Newcastle	Framlingt	Newcastle	NE2 4HH	United Ki	0	Illumina	cDNA	transcrip
Newcastle	Framlingt	Newcastle	NE2 4HH	United Ki	0	Illumina	cDNA	transcrip
Newcastle	Framlingt	Newcastle	NE2 4HH	United Ki	0	Illumina	cDNA	transcrip
Newcastle	Framlingt	Newcastle	NE2 4HH	United Ki	0	Illumina	cDNA	transcrip
Newcastle	Framlingt	Newcastle	NE2 4HH	United Ki	0	Illumina	cDNA	transcrip

For Peer Review

!Sample_l	!Sample_r	!Sample_r	!Sample_supplementary_file_1
RNA-Seq	BioSample	SRA: http	ftp://ftp.ncbi.nlm.nih.gov/geo/samples/GSM3998nnn/GSM399[illegible]
RNA-Seq	BioSample	SRA: http	ftp://ftp.ncbi.nlm.nih.gov/geo/samples/GSM3998nnn/GSM399[illegible]
RNA-Seq	BioSample	SRA: http	ftp://ftp.ncbi.nlm.nih.gov/geo/samples/GSM3998nnn/GSM399[illegible]
RNA-Seq	BioSample	SRA: http	ftp://ftp.ncbi.nlm.nih.gov/geo/samples/GSM3998nnn/GSM399[illegible]
RNA-Seq	BioSample	SRA: http	ftp://ftp.ncbi.nlm.nih.gov/geo/samples/GSM3998nnn/GSM399[illegible]
RNA-Seq	BioSample	SRA: http	ftp://ftp.ncbi.nlm.nih.gov/geo/samples/GSM3998nnn/GSM399[illegible]
RNA-Seq	BioSample	SRA: http	ftp://ftp.ncbi.nlm.nih.gov/geo/samples/GSM3998nnn/GSM399[illegible]
RNA-Seq	BioSample	SRA: http	ftp://ftp.ncbi.nlm.nih.gov/geo/samples/GSM3998nnn/GSM399[illegible]
RNA-Seq	BioSample	SRA: http	ftp://ftp.ncbi.nlm.nih.gov/geo/samples/GSM3998nnn/GSM399[illegible]
RNA-Seq	BioSample	SRA: http	ftp://ftp.ncbi.nlm.nih.gov/geo/samples/GSM3998nnn/GSM399[illegible]
RNA-Seq	BioSample	SRA: http	ftp://ftp.ncbi.nlm.nih.gov/geo/samples/GSM3998nnn/GSM399[illegible]
RNA-Seq	BioSample	SRA: http	ftp://ftp.ncbi.nlm.nih.gov/geo/samples/GSM3998nnn/GSM399[illegible]
RNA-Seq	BioSample	SRA: http	ftp://ftp.ncbi.nlm.nih.gov/geo/samples/GSM3998nnn/GSM399[illegible]
RNA-Seq	BioSample	SRA: http	ftp://ftp.ncbi.nlm.nih.gov/geo/samples/GSM3998nnn/GSM399[illegible]
RNA-Seq	BioSample	SRA: http	ftp://ftp.ncbi.nlm.nih.gov/geo/samples/GSM3998nnn/GSM399[illegible]
RNA-Seq	BioSample	SRA: http	ftp://ftp.ncbi.nlm.nih.gov/geo/samples/GSM3998nnn/GSM399[illegible]
RNA-Seq	BioSample	SRA: http	ftp://ftp.ncbi.nlm.nih.gov/geo/samples/GSM3998nnn/GSM399[illegible]
RNA-Seq	BioSample	SRA: http	ftp://ftp.ncbi.nlm.nih.gov/geo/samples/GSM3998nnn/GSM399[illegible]
RNA-Seq	BioSample	SRA: http	ftp://ftp.ncbi.nlm.nih.gov/geo/samples/GSM3998nnn/GSM399[illegible]
RNA-Seq	BioSample	SRA: http	ftp://ftp.ncbi.nlm.nih.gov/geo/samples/GSM3998nnn/GSM399[illegible]
RNA-Seq	BioSample	SRA: http	ftp://ftp.ncbi.nlm.nih.gov/geo/samples/GSM3998nnn/GSM399[illegible]
RNA-Seq	BioSample	SRA: http	ftp://ftp.ncbi.nlm.nih.gov/geo/samples/GSM3998nnn/GSM399[illegible]
RNA-Seq	BioSample	SRA: http	ftp://ftp.ncbi.nlm.nih.gov/geo/samples/GSM3998nnn/GSM399[illegible]
RNA-Seq	BioSample	SRA: http	ftp://ftp.ncbi.nlm.nih.gov/geo/samples/GSM3998nnn/GSM399[illegible]
RNA-Seq	BioSample	SRA: http	ftp://ftp.ncbi.nlm.nih.gov/geo/samples/GSM3998nnn/GSM399[illegible]
RNA-Seq	BioSample	SRA: http	ftp://ftp.ncbi.nlm.nih.gov/geo/samples/GSM3998nnn/GSM399[illegible]
RNA-Seq	BioSample	SRA: http	ftp://ftp.ncbi.nlm.nih.gov/geo/samples/GSM3998nnn/GSM399[illegible]
RNA-Seq	BioSample	SRA: http	ftp://ftp.ncbi.nlm.nih.gov/geo/samples/GSM3998nnn/GSM399[illegible]
RNA-Seq	BioSample	SRA: http	ftp://ftp.ncbi.nlm.nih.gov/geo/samples/GSM3998nnn/GSM399[illegible]
RNA-Seq	BioSample	SRA: http	ftp://ftp.ncbi.nlm.nih.gov/geo/samples/GSM3998nnn/GSM399[illegible]
RNA-Seq	BioSample	SRA: http	ftp://ftp.ncbi.nlm.nih.gov/geo/samples/GSM3998nnn/GSM399[illegible]
RNA-Seq	BioSample	SRA: http	ftp://ftp.ncbi.nlm.nih.gov/geo/samples/GSM3998nnn/GSM399[illegible]
RNA-Seq	BioSample	SRA: http	ftp://ftp.ncbi.nlm.nih.gov/geo/samples/GSM3998nnn/GSM399[illegible]
RNA-Seq	BioSample	SRA: http	ftp://ftp.ncbi.nlm.nih.gov/geo/samples/GSM3998nnn/GSM399[illegible]
RNA-Seq	BioSample	SRA: http	ftp://ftp.ncbi.nlm.nih.gov/geo/samples/GSM3998nnn/GSM399[illegible]
RNA-Seq	BioSample	SRA: http	ftp://ftp.ncbi.nlm.nih.gov/geo/samples/GSM3998nnn/GSM399[illegible]
RNA-Seq	BioSample	SRA: http	ftp://ftp.ncbi.nlm.nih.gov/geo/samples/GSM3998nnn/GSM399[illegible]
RNA-Seq	BioSample	SRA: http	ftp://ftp.ncbi.nlm.nih.gov/geo/samples/GSM3998nnn/GSM399[illegible]
RNA-Seq	BioSample	SRA: http	ftp://ftp.ncbi.nlm.nih.gov/geo/samples/GSM3998nnn/GSM399[illegible]
RNA-Seq	BioSample	SRA: http	ftp://ftp.ncbi.nlm.nih.gov/geo/samples/GSM3998nnn/GSM399[illegible]
RNA-Seq	BioSample	SRA: http	ftp://ftp.ncbi.nlm.nih.gov/geo/samples/GSM3998nnn/GSM399[illegible]
RNA-Seq	BioSample	SRA: http	ftp://ftp.ncbi.nlm.nih.gov/geo/samples/GSM3998nnn/GSM399[illegible]
RNA-Seq	BioSample	SRA: http	ftp://ftp.ncbi.nlm.nih.gov/geo/samples/GSM3998nnn/GSM399[illegible]
RNA-Seq	BioSample	SRA: http	ftp://ftp.ncbi.nlm.nih.gov/geo/samples/GSM3998nnn/GSM399[illegible]
RNA-Seq	BioSample	SRA: http	ftp://ftp.ncbi.nlm.nih.gov/geo/samples/GSM3998nnn/GSM399[illegible]
RNA-Seq	BioSample	SRA: http	ftp://ftp.ncbi.nlm.nih.gov/geo/samples/GSM3998nnn/GSM399[illegible]
RNA-Seq	BioSample	SRA: http	ftp://ftp.ncbi.nlm.nih.gov/geo/samples/GSM3998nnn/GSM399[illegible]
RNA-Seq	BioSample	SRA: http	ftp://ftp.ncbi.nlm.nih.gov/geo/samples/GSM3998nnn/GSM399[illegible]
RNA-Seq	BioSample	SRA: http	ftp://ftp.ncbi.nlm.nih.gov/geo/samples/GSM3998nnn/GSM399[illegible]
RNA-Seq	BioSample	SRA: http	ftp://ftp.ncbi.nlm.nih.gov/geo/samples/GSM3998nnn/GSM399[illegible]
RNA-Seq	BioSample	SRA: http	ftp://ftp.ncbi.nlm.nih.gov/geo/samples/GSM3998nnn/GSM399[illegible]
RNA-Seq	BioSample	SRA: http	ftp://ftp.ncbi.nlm.nih.gov/geo/samples/GSM3998nnn/GSM399[illegible]
RNA-Seq	BioSample	SRA: http	ftp://ftp.ncbi.nlm.nih.gov/geo/samples/GSM3998nnn/GSM399[illegible]
RNA-Seq	BioSample	SRA: http	ftp://ftp.ncbi.nlm.nih.gov/geo/samples/GSM3998nnn/GSM399[illegible]
RNA-Seq	BioSample	SRA: http	ftp://ftp.ncbi.nlm.nih.gov/geo/samples/GSM3998nnn/GSM399[illegible]
RNA-Seq	BioSample	SRA: http	ftp://ftp.ncbi.nlm.nih.gov/geo/samples/GSM3998nnn/GSM399[illegible]

For Peer Review

RNA-Seq	BioSample	SRA: http	ftp://ftp.ncbi.nlm.nih.gov/geo/samples/GSM3998nnn/GSM399
RNA-Seq	BioSample	SRA: http	ftp://ftp.ncbi.nlm.nih.gov/geo/samples/GSM3998nnn/GSM399
RNA-Seq	BioSample	SRA: http	ftp://ftp.ncbi.nlm.nih.gov/geo/samples/GSM3998nnn/GSM399
RNA-Seq	BioSample	SRA: http	ftp://ftp.ncbi.nlm.nih.gov/geo/samples/GSM3998nnn/GSM399
RNA-Seq	BioSample	SRA: http	ftp://ftp.ncbi.nlm.nih.gov/geo/samples/GSM3998nnn/GSM399
RNA-Seq	BioSample	SRA: http	ftp://ftp.ncbi.nlm.nih.gov/geo/samples/GSM3998nnn/GSM399
RNA-Seq	BioSample	SRA: http	ftp://ftp.ncbi.nlm.nih.gov/geo/samples/GSM3998nnn/GSM399
RNA-Seq	BioSample	SRA: http	ftp://ftp.ncbi.nlm.nih.gov/geo/samples/GSM3998nnn/GSM399
RNA-Seq	BioSample	SRA: http	ftp://ftp.ncbi.nlm.nih.gov/geo/samples/GSM3998nnn/GSM399
RNA-Seq	BioSample	SRA: http	ftp://ftp.ncbi.nlm.nih.gov/geo/samples/GSM3998nnn/GSM399
RNA-Seq	BioSample	SRA: http	ftp://ftp.ncbi.nlm.nih.gov/geo/samples/GSM3998nnn/GSM399
RNA-Seq	BioSample	SRA: http	ftp://ftp.ncbi.nlm.nih.gov/geo/samples/GSM3998nnn/GSM399
RNA-Seq	BioSample	SRA: http	ftp://ftp.ncbi.nlm.nih.gov/geo/samples/GSM3998nnn/GSM399
RNA-Seq	BioSample	SRA: http	ftp://ftp.ncbi.nlm.nih.gov/geo/samples/GSM3998nnn/GSM399
RNA-Seq	BioSample	SRA: http	ftp://ftp.ncbi.nlm.nih.gov/geo/samples/GSM3998nnn/GSM399
RNA-Seq	BioSample	SRA: http	ftp://ftp.ncbi.nlm.nih.gov/geo/samples/GSM3998nnn/GSM399
RNA-Seq	BioSample	SRA: http	ftp://ftp.ncbi.nlm.nih.gov/geo/samples/GSM3998nnn/GSM399
RNA-Seq	BioSample	SRA: http	ftp://ftp.ncbi.nlm.nih.gov/geo/samples/GSM3998nnn/GSM399
RNA-Seq	BioSample	SRA: http	ftp://ftp.ncbi.nlm.nih.gov/geo/samples/GSM3998nnn/GSM399
RNA-Seq	BioSample	SRA: http	ftp://ftp.ncbi.nlm.nih.gov/geo/samples/GSM3998nnn/GSM399
RNA-Seq	BioSample	SRA: http	ftp://ftp.ncbi.nlm.nih.gov/geo/samples/GSM3998nnn/GSM399
RNA-Seq	BioSample	SRA: http	ftp://ftp.ncbi.nlm.nih.gov/geo/samples/GSM3998nnn/GSM399
RNA-Seq	BioSample	SRA: http	ftp://ftp.ncbi.nlm.nih.gov/geo/samples/GSM3998nnn/GSM399
RNA-Seq	BioSample	SRA: http	ftp://ftp.ncbi.nlm.nih.gov/geo/samples/GSM3998nnn/GSM399
RNA-Seq	BioSample	SRA: http	ftp://ftp.ncbi.nlm.nih.gov/geo/samples/GSM3998nnn/GSM399
RNA-Seq	BioSample	SRA: http	ftp://ftp.ncbi.nlm.nih.gov/geo/samples/GSM3998nnn/GSM399
RNA-Seq	BioSample	SRA: http	ftp://ftp.ncbi.nlm.nih.gov/geo/samples/GSM3998nnn/GSM399
RNA-Seq	BioSample	SRA: http	ftp://ftp.ncbi.nlm.nih.gov/geo/samples/GSM3998nnn/GSM399
RNA-Seq	BioSample	SRA: http	ftp://ftp.ncbi.nlm.nih.gov/geo/samples/GSM3998nnn/GSM399
RNA-Seq	BioSample	SRA: http	ftp://ftp.ncbi.nlm.nih.gov/geo/samples/GSM3998nnn/GSM399
RNA-Seq	BioSample	SRA: http	ftp://ftp.ncbi.nlm.nih.gov/geo/samples/GSM3998nnn/GSM399
RNA-Seq	BioSample	SRA: http	ftp://ftp.ncbi.nlm.nih.gov/geo/samples/GSM3998nnn/GSM399
RNA-Seq	BioSample	SRA: http	ftp://ftp.ncbi.nlm.nih.gov/geo/samples/GSM3998nnn/GSM399
RNA-Seq	BioSample	SRA: http	ftp://ftp.ncbi.nlm.nih.gov/geo/samples/GSM3998nnn/GSM399
RNA-Seq	BioSample	SRA: http	ftp://ftp.ncbi.nlm.nih.gov/geo/samples/GSM3998nnn/GSM399
RNA-Seq	BioSample	SRA: http	ftp://ftp.ncbi.nlm.nih.gov/geo/samples/GSM3998nnn/GSM399
RNA-Seq	BioSample	SRA: http	ftp://ftp.ncbi.nlm.nih.gov/geo/samples/GSM3998nnn/GSM399
RNA-Seq	BioSample	SRA: http	ftp://ftp.ncbi.nlm.nih.gov/geo/samples/GSM3998nnn/GSM399
RNA-Seq	BioSample	SRA: http	ftp://ftp.ncbi.nlm.nih.gov/geo/samples/GSM3998nnn/GSM399
RNA-Seq	BioSample	SRA: http	ftp://ftp.ncbi.nlm.nih.gov/geo/samples/GSM3998nnn/GSM399
RNA-Seq	BioSample	SRA: http	ftp://ftp.ncbi.nlm.nih.gov/geo/samples/GSM3998nnn/GSM399
RNA-Seq	BioSample	SRA: http	ftp://ftp.ncbi.nlm.nih.gov/geo/samples/GSM3998nnn/GSM399
RNA-Seq	BioSample	SRA: http	ftp://ftp.ncbi.nlm.nih.gov/geo/samples/GSM3998nnn/GSM399
RNA-Seq	BioSample	SRA: http	ftp://ftp.ncbi.nlm.nih.gov/geo/samples/GSM3998nnn/GSM399
RNA-Seq	BioSample	SRA: http	ftp://ftp.ncbi.nlm.nih.gov/geo/samples/GSM3998nnn/GSM399
RNA-Seq	BioSample	SRA: http	ftp://ftp.ncbi.nlm.nih.gov/geo/samples/GSM3998nnn/GSM399
RNA-Seq	BioSample	SRA: http	ftp://ftp.ncbi.nlm.nih.gov/geo/samples/GSM3998nnn/GSM399
RNA-Seq	BioSample	SRA: http	ftp://ftp.ncbi.nlm.nih.gov/geo/samples/GSM3998nnn/GSM399
RNA-Seq	BioSample	SRA: http	ftp://ftp.ncbi.nlm.nih.gov/geo/samples/GSM3998nnn/GSM399
RNA-Seq	BioSample	SRA: http	ftp://ftp.ncbi.nlm.nih.gov/geo/samples/GSM3998nnn/GSM399
RNA-Seq	BioSample	SRA: http	ftp://ftp.ncbi.nlm.nih.gov/geo/samples/GSM3998nnn/GSM399
RNA-Seq	BioSample	SRA: http	ftp://ftp.ncbi.nlm.nih.gov/geo/samples/GSM3998nnn/GSM399
RNA-Seq	BioSample	SRA: http	ftp://ftp.ncbi.nlm.nih.gov/geo/samples/GSM3998nnn/GSM399
RNA-Seq	BioSample	SRA: http	ftp://ftp.ncbi.nlm.nih.gov/geo/samples/GSM3998nnn/GSM399
RNA-Seq	BioSample	SRA: http	ftp://ftp.ncbi.nlm.nih.gov/geo/samples/GSM3998nnn/GSM399
RNA-Seq	BioSample	SRA: http	ftp://ftp.ncbi.nlm.nih.gov/geo/samples/GSM3998nnn/GSM399
RNA-Seq	BioSample	SRA: http	ftp://ftp.ncbi.nlm.nih.gov/geo/samples/GSM3998nnn/GSM399
RNA-Seq	BioSample	SRA: http	ftp://ftp.ncbi.nlm.nih.gov/geo/samples/GSM3998nnn/GSM399

For Peer Review

RNA-Seq	BioSample	SRA:	http	ftp://ftp.ncbi.nlm.nih.gov/geo/samples/GSM3998nnn/GSM399
RNA-Seq	BioSample	SRA:	http	ftp://ftp.ncbi.nlm.nih.gov/geo/samples/GSM3998nnn/GSM399
RNA-Seq	BioSample	SRA:	http	ftp://ftp.ncbi.nlm.nih.gov/geo/samples/GSM3998nnn/GSM399
RNA-Seq	BioSample	SRA:	http	ftp://ftp.ncbi.nlm.nih.gov/geo/samples/GSM3998nnn/GSM399
RNA-Seq	BioSample	SRA:	http	ftp://ftp.ncbi.nlm.nih.gov/geo/samples/GSM3998nnn/GSM399
RNA-Seq	BioSample	SRA:	http	ftp://ftp.ncbi.nlm.nih.gov/geo/samples/GSM3998nnn/GSM399
RNA-Seq	BioSample	SRA:	http	ftp://ftp.ncbi.nlm.nih.gov/geo/samples/GSM3998nnn/GSM399
RNA-Seq	BioSample	SRA:	http	ftp://ftp.ncbi.nlm.nih.gov/geo/samples/GSM3998nnn/GSM399
RNA-Seq	BioSample	SRA:	http	ftp://ftp.ncbi.nlm.nih.gov/geo/samples/GSM3998nnn/GSM399
RNA-Seq	BioSample	SRA:	http	ftp://ftp.ncbi.nlm.nih.gov/geo/samples/GSM3998nnn/GSM399
RNA-Seq	BioSample	SRA:	http	ftp://ftp.ncbi.nlm.nih.gov/geo/samples/GSM3998nnn/GSM399
RNA-Seq	BioSample	SRA:	http	ftp://ftp.ncbi.nlm.nih.gov/geo/samples/GSM3998nnn/GSM399
RNA-Seq	BioSample	SRA:	http	ftp://ftp.ncbi.nlm.nih.gov/geo/samples/GSM3998nnn/GSM399
RNA-Seq	BioSample	SRA:	http	ftp://ftp.ncbi.nlm.nih.gov/geo/samples/GSM3998nnn/GSM399
RNA-Seq	BioSample	SRA:	http	ftp://ftp.ncbi.nlm.nih.gov/geo/samples/GSM3998nnn/GSM399
RNA-Seq	BioSample	SRA:	http	ftp://ftp.ncbi.nlm.nih.gov/geo/samples/GSM3998nnn/GSM399
RNA-Seq	BioSample	SRA:	http	ftp://ftp.ncbi.nlm.nih.gov/geo/samples/GSM3998nnn/GSM399
RNA-Seq	BioSample	SRA:	http	ftp://ftp.ncbi.nlm.nih.gov/geo/samples/GSM3998nnn/GSM399
RNA-Seq	BioSample	SRA:	http	ftp://ftp.ncbi.nlm.nih.gov/geo/samples/GSM3998nnn/GSM399
RNA-Seq	BioSample	SRA:	http	ftp://ftp.ncbi.nlm.nih.gov/geo/samples/GSM3998nnn/GSM399
RNA-Seq	BioSample	SRA:	http	ftp://ftp.ncbi.nlm.nih.gov/geo/samples/GSM3998nnn/GSM399
RNA-Seq	BioSample	SRA:	http	ftp://ftp.ncbi.nlm.nih.gov/geo/samples/GSM3998nnn/GSM399
RNA-Seq	BioSample	SRA:	http	ftp://ftp.ncbi.nlm.nih.gov/geo/samples/GSM3998nnn/GSM399
RNA-Seq	BioSample	SRA:	http	ftp://ftp.ncbi.nlm.nih.gov/geo/samples/GSM3998nnn/GSM399
RNA-Seq	BioSample	SRA:	http	ftp://ftp.ncbi.nlm.nih.gov/geo/samples/GSM3998nnn/GSM399
RNA-Seq	BioSample	SRA:	http	ftp://ftp.ncbi.nlm.nih.gov/geo/samples/GSM3998nnn/GSM399
RNA-Seq	BioSample	SRA:	http	ftp://ftp.ncbi.nlm.nih.gov/geo/samples/GSM3998nnn/GSM399
RNA-Seq	BioSample	SRA:	http	ftp://ftp.ncbi.nlm.nih.gov/geo/samples/GSM3998nnn/GSM399
RNA-Seq	BioSample	SRA:	http	ftp://ftp.ncbi.nlm.nih.gov/geo/samples/GSM3998nnn/GSM399
RNA-Seq	BioSample	SRA:	http	ftp://ftp.ncbi.nlm.nih.gov/geo/samples/GSM3998nnn/GSM399
RNA-Seq	BioSample	SRA:	http	ftp://ftp.ncbi.nlm.nih.gov/geo/samples/GSM3998nnn/GSM399
RNA-Seq	BioSample	SRA:	http	ftp://ftp.ncbi.nlm.nih.gov/geo/samples/GSM3998nnn/GSM399
RNA-Seq	BioSample	SRA:	http	ftp://ftp.ncbi.nlm.nih.gov/geo/samples/GSM3998nnn/GSM399
RNA-Seq	BioSample	SRA:	http	ftp://ftp.ncbi.nlm.nih.gov/geo/samples/GSM3998nnn/GSM399
RNA-Seq	BioSample	SRA:	http	ftp://ftp.ncbi.nlm.nih.gov/geo/samples/GSM3998nnn/GSM399
RNA-Seq	BioSample	SRA:	http	ftp://ftp.ncbi.nlm.nih.gov/geo/samples/GSM3998nnn/GSM399
RNA-Seq	BioSample	SRA:	http	ftp://ftp.ncbi.nlm.nih.gov/geo/samples/GSM3998nnn/GSM399
RNA-Seq	BioSample	SRA:	http	ftp://ftp.ncbi.nlm.nih.gov/geo/samples/GSM3998nnn/GSM399
RNA-Seq	BioSample	SRA:	http	ftp://ftp.ncbi.nlm.nih.gov/geo/samples/GSM3998nnn/GSM399
RNA-Seq	BioSample	SRA:	http	ftp://ftp.ncbi.nlm.nih.gov/geo/samples/GSM3998nnn/GSM399
RNA-Seq	BioSample	SRA:	http	ftp://ftp.ncbi.nlm.nih.gov/geo/samples/GSM3998nnn/GSM399
RNA-Seq	BioSample	SRA:	http	ftp://ftp.ncbi.nlm.nih.gov/geo/samples/GSM3998nnn/GSM399
RNA-Seq	BioSample	SRA:	http	ftp://ftp.ncbi.nlm.nih.gov/geo/samples/GSM3998nnn/GSM399
RNA-Seq	BioSample	SRA:	http	ftp://ftp.ncbi.nlm.nih.gov/geo/samples/GSM3998nnn/GSM399
RNA-Seq	BioSample	SRA:	http	ftp://ftp.ncbi.nlm.nih.gov/geo/samples/GSM3998nnn/GSM399
RNA-Seq	BioSample	SRA:	http	ftp://ftp.ncbi.nlm.nih.gov/geo/samples/GSM3998nnn/GSM399
RNA-Seq	BioSample	SRA:	http	ftp://ftp.ncbi.nlm.nih.gov/geo/samples/GSM3998nnn/GSM399
RNA-Seq	BioSample	SRA:	http	ftp://ftp.ncbi.nlm.nih.gov/geo/samples/GSM3998nnn/GSM399
RNA-Seq	BioSample	SRA:	http	ftp://ftp.ncbi.nlm.nih.gov/geo/samples/GSM3998nnn/GSM399
RNA-Seq	BioSample	SRA:	http	ftp://ftp.ncbi.nlm.nih.gov/geo/samples/GSM3998nnn/GSM399
RNA-Seq	BioSample	SRA:	http	ftp://ftp.ncbi.nlm.nih.gov/geo/samples/GSM3998nnn/GSM399
RNA-Seq	BioSample	SRA:	http	ftp://ftp.ncbi.nlm.nih.gov/geo/samples/GSM3998nnn/GSM399
RNA-Seq	BioSample	SRA:	http	ftp://ftp.ncbi.nlm.nih.gov/geo/samples/GSM3998nnn/GSM399
RNA-Seq	BioSample	SRA:	http	ftp://ftp.ncbi.nlm.nih.gov/geo/samples/GSM3998nnn/GSM399
RNA-Seq	BioSample	SRA:	http	ftp://ftp.ncbi.nlm.nih.gov/geo/samples/GSM3998nnn/GSM399
RNA-Seq	BioSample	SRA:	http	ftp://ftp.ncbi.nlm.nih.gov/geo/samples/GSM3998nnn/GSM399
RNA-Seq	BioSample	SRA:	http	ftp://ftp.ncbi.nlm.nih.gov/geo/samples/GSM3998nnn/GSM399
RNA-Seq	BioSample	SRA:	http	ftp://ftp.ncbi.nlm.nih.gov/geo/samples/GSM3998nnn/GSM399

For Peer Review

RNA-Seq	BioSample	SRA: http	ftp://ftp.ncbi.nlm.nih.gov/geo/samples/GSM3998nnn/GSM399[illegible]
RNA-Seq	BioSample	SRA: http	ftp://ftp.ncbi.nlm.nih.gov/geo/samples/GSM3998nnn/GSM399[illegible]
RNA-Seq	BioSample	SRA: http	ftp://ftp.ncbi.nlm.nih.gov/geo/samples/GSM3998nnn/GSM399[illegible]
RNA-Seq	BioSample	SRA: http	ftp://ftp.ncbi.nlm.nih.gov/geo/samples/GSM3998nnn/GSM399[illegible]
RNA-Seq	BioSample	SRA: http	ftp://ftp.ncbi.nlm.nih.gov/geo/samples/GSM3998nnn/GSM399[illegible]
RNA-Seq	BioSample	SRA: http	ftp://ftp.ncbi.nlm.nih.gov/geo/samples/GSM3998nnn/GSM399[illegible]
RNA-Seq	BioSample	SRA: http	ftp://ftp.ncbi.nlm.nih.gov/geo/samples/GSM3998nnn/GSM399[illegible]
RNA-Seq	BioSample	SRA: http	ftp://ftp.ncbi.nlm.nih.gov/geo/samples/GSM3998nnn/GSM399[illegible]
RNA-Seq	BioSample	SRA: http	ftp://ftp.ncbi.nlm.nih.gov/geo/samples/GSM3998nnn/GSM399[illegible]
RNA-Seq	BioSample	SRA: http	ftp://ftp.ncbi.nlm.nih.gov/geo/samples/GSM3998nnn/GSM399[illegible]
RNA-Seq	BioSample	SRA: http	ftp://ftp.ncbi.nlm.nih.gov/geo/samples/GSM3998nnn/GSM399[illegible]
RNA-Seq	BioSample	SRA: http	ftp://ftp.ncbi.nlm.nih.gov/geo/samples/GSM3998nnn/GSM399[illegible]
RNA-Seq	BioSample	SRA: http	ftp://ftp.ncbi.nlm.nih.gov/geo/samples/GSM3998nnn/GSM399[illegible]
RNA-Seq	BioSample	SRA: http	ftp://ftp.ncbi.nlm.nih.gov/geo/samples/GSM3998nnn/GSM399[illegible]
RNA-Seq	BioSample	SRA: http	ftp://ftp.ncbi.nlm.nih.gov/geo/samples/GSM3998nnn/GSM399[illegible]
RNA-Seq	BioSample	SRA: http	ftp://ftp.ncbi.nlm.nih.gov/geo/samples/GSM3998nnn/GSM399[illegible]
RNA-Seq	BioSample	SRA: http	ftp://ftp.ncbi.nlm.nih.gov/geo/samples/GSM3998nnn/GSM399[illegible]
RNA-Seq	BioSample	SRA: http	ftp://ftp.ncbi.nlm.nih.gov/geo/samples/GSM3998nnn/GSM399[illegible]
RNA-Seq	BioSample	SRA: http	ftp://ftp.ncbi.nlm.nih.gov/geo/samples/GSM3998nnn/GSM399[illegible]
RNA-Seq	BioSample	SRA: http	ftp://ftp.ncbi.nlm.nih.gov/geo/samples/GSM3998nnn/GSM399[illegible]
RNA-Seq	BioSample	SRA: http	ftp://ftp.ncbi.nlm.nih.gov/geo/samples/GSM3998nnn/GSM399[illegible]
RNA-Seq	BioSample	SRA: http	ftp://ftp.ncbi.nlm.nih.gov/geo/samples/GSM3998nnn/GSM399[illegible]
RNA-Seq	BioSample	SRA: http	ftp://ftp.ncbi.nlm.nih.gov/geo/samples/GSM3998nnn/GSM399[illegible]
RNA-Seq	BioSample	SRA: http	ftp://ftp.ncbi.nlm.nih.gov/geo/samples/GSM3998nnn/GSM399[illegible]
RNA-Seq	BioSample	SRA: http	ftp://ftp.ncbi.nlm.nih.gov/geo/samples/GSM3998nnn/GSM399[illegible]
RNA-Seq	BioSample	SRA: http	ftp://ftp.ncbi.nlm.nih.gov/geo/samples/GSM3998nnn/GSM399[illegible]
RNA-Seq	BioSample	SRA: http	ftp://ftp.ncbi.nlm.nih.gov/geo/samples/GSM3998nnn/GSM399[illegible]
RNA-Seq	BioSample	SRA: http	ftp://ftp.ncbi.nlm.nih.gov/geo/samples/GSM3998nnn/GSM399[illegible]
RNA-Seq	BioSample	SRA: http	ftp://ftp.ncbi.nlm.nih.gov/geo/samples/GSM3998nnn/GSM399[illegible]
RNA-Seq	BioSample	SRA: http	ftp://ftp.ncbi.nlm.nih.gov/geo/samples/GSM3998nnn/GSM399[illegible]
RNA-Seq	BioSample	SRA: http	ftp://ftp.ncbi.nlm.nih.gov/geo/samples/GSM3998nnn/GSM399[illegible]
RNA-Seq	BioSample	SRA: http	ftp://ftp.ncbi.nlm.nih.gov/geo/samples/GSM3998nnn/GSM399[illegible]
RNA-Seq	BioSample	SRA: http	ftp://ftp.ncbi.nlm.nih.gov/geo/samples/GSM3998nnn/GSM399[illegible]
RNA-Seq	BioSample	SRA: http	ftp://ftp.ncbi.nlm.nih.gov/geo/samples/GSM3998nnn/GSM399[illegible]
RNA-Seq	BioSample	SRA: http	ftp://ftp.ncbi.nlm.nih.gov/geo/samples/GSM3998nnn/GSM399[illegible]
RNA-Seq	BioSample	SRA: http	ftp://ftp.ncbi.nlm.nih.gov/geo/samples/GSM3998nnn/GSM399[illegible]
RNA-Seq	BioSample	SRA: http	ftp://ftp.ncbi.nlm.nih.gov/geo/samples/GSM3998nnn/GSM399[illegible]
RNA-Seq	BioSample	SRA: http	ftp://ftp.ncbi.nlm.nih.gov/geo/samples/GSM3998nnn/GSM399[illegible]
RNA-Seq	BioSample	SRA: http	ftp://ftp.ncbi.nlm.nih.gov/geo/samples/GSM3998nnn/GSM399[illegible]
RNA-Seq	BioSample	SRA: http	ftp://ftp.ncbi.nlm.nih.gov/geo/samples/GSM3998nnn/GSM399[illegible]
RNA-Seq	BioSample	SRA: http	ftp://ftp.ncbi.nlm.nih.gov/geo/samples/GSM3998nnn/GSM399[illegible]
RNA-Seq	BioSample	SRA: http	ftp://ftp.ncbi.nlm.nih.gov/geo/samples/GSM3998nnn/GSM399[illegible]
RNA-Seq	BioSample	SRA: http	ftp://ftp.ncbi.nlm.nih.gov/geo/samples/GSM3998nnn/GSM399[illegible]
RNA-Seq	BioSample	SRA: http	ftp://ftp.ncbi.nlm.nih.gov/geo/samples/GSM3998nnn/GSM399[illegible]
RNA-Seq	BioSample	SRA: http	ftp://ftp.ncbi.nlm.nih.gov/geo/samples/GSM3998nnn/GSM399[illegible]
RNA-Seq	BioSample	SRA: http	ftp://ftp.ncbi.nlm.nih.gov/geo/samples/GSM3998nnn/GSM399[illegible]
RNA-Seq	BioSample	SRA: http	ftp://ftp.ncbi.nlm.nih.gov/geo/samples/GSM3998nnn/GSM399[illegible]

For Peer Review

8167/suppl/GSM3998167_017-Ann-Daly_S1.counts.txt.gz
8168/suppl/GSM3998168_018-Ann-Daly_S2.counts.txt.gz
8169/suppl/GSM3998169_029-Ann-Daly_S4.counts.txt.gz
8170/suppl/GSM3998170_030-Ann-Daly_S5.counts.txt.gz
8171/suppl/GSM3998171_031-Ann-Daly_S6.counts.txt.gz
8172/suppl/GSM3998172_032-Ann-Daly_S7.counts.txt.gz
8173/suppl/GSM3998173_033-Ann-Daly_S8.counts.txt.gz
8174/suppl/GSM3998174_035-Ann-Daly_S10.counts.txt.gz
8175/suppl/GSM3998175_036-Ann-Daly_S11.counts.txt.gz
8176/suppl/GSM3998176_037-Ann-Daly_S12.counts.txt.gz
8177/suppl/GSM3998177_038-Ann-Daly_S13.counts.txt.gz
8178/suppl/GSM3998178_040-Ann-Daly_S14.counts.txt.gz
8179/suppl/GSM3998179_041-Ann-Daly_S15.counts.txt.gz
8180/suppl/GSM3998180_042-Ann-Daly_S16.counts.txt.gz
8181/suppl/GSM3998181_043-Ann-Daly_S1.counts.txt.gz
8182/suppl/GSM3998182_044-Ann-Daly_S2.counts.txt.gz
8183/suppl/GSM3998183_046-Ann-Daly_S3.counts.txt.gz
8184/suppl/GSM3998184_047-Ann-Daly_S4.counts.txt.gz
8185/suppl/GSM3998185_048-Ann-Daly_S5.counts.txt.gz
8186/suppl/GSM3998186_049-Ann-Daly_S6.counts.txt.gz
8187/suppl/GSM3998187_051-Ann-Daly_S7.counts.txt.gz
8188/suppl/GSM3998188_053-Ann-Daly_S9.counts.txt.gz
8189/suppl/GSM3998189_054-Ann-Daly_S10.counts.txt.gz
8190/suppl/GSM3998190_055-Ann-Daly_S11.counts.txt.gz
8191/suppl/GSM3998191_059-Ann-Daly_S12.counts.txt.gz
8192/suppl/GSM3998192_060-Ann-Daly_S13.counts.txt.gz
8193/suppl/GSM3998193_061-Ann-Daly_S14.counts.txt.gz
8194/suppl/GSM3998194_062-Ann-Daly_S15.counts.txt.gz
8195/suppl/GSM3998195_075-Ann-Daly_S16.counts.txt.gz
8196/suppl/GSM3998196_076-Ann-Daly_S3.counts.txt.gz
8197/suppl/GSM3998197_1_20.counts.txt.gz
8198/suppl/GSM3998198_10_352.counts.txt.gz
8199/suppl/GSM3998199_101-Ann-Daly_S1.counts.txt.gz
8200/suppl/GSM3998200_102-Ann-Daly_S2.counts.txt.gz
8201/suppl/GSM3998201_103-Ann-Daly_S3.counts.txt.gz
8202/suppl/GSM3998202_104-Ann-Daly_S4.counts.txt.gz
8203/suppl/GSM3998203_105-Ann-Daly_S5.counts.txt.gz
8204/suppl/GSM3998204_106-Ann-Daly_S6.counts.txt.gz
8205/suppl/GSM3998205_108-Ann-Daly_S8.counts.txt.gz
8206/suppl/GSM3998206_109-Ann-Daly_S1.counts.txt.gz
8207/suppl/GSM3998207_110-Ann-Daly_S2.counts.txt.gz
8208/suppl/GSM3998208_111-Ann-Daly_S3.counts.txt.gz
8209/suppl/GSM3998209_112-Ann-Daly_S4.counts.txt.gz
8210/suppl/GSM3998210_114-Ann-Daly_S5.counts.txt.gz
8211/suppl/GSM3998211_115-Ann-Daly_S6.counts.txt.gz
8212/suppl/GSM3998212_116-Ann-Daly_S7.counts.txt.gz
8213/suppl/GSM3998213_12_469.counts.txt.gz
8214/suppl/GSM3998214_120-Ann-Daly_S9.counts.txt.gz
8215/suppl/GSM3998215_124-Ann-Daly_S10.counts.txt.gz
8216/suppl/GSM3998216_127-Ann-Daly_S11.counts.txt.gz
8217/suppl/GSM3998217_128-Ann-Daly_S12.counts.txt.gz
8218/suppl/GSM3998218_129-Ann-Daly_S13.counts.txt.gz
8219/suppl/GSM3998219_13_470.counts.txt.gz
8220/suppl/GSM3998220_130-Ann-Daly_S14.counts.txt.gz
8221/suppl/GSM3998221_132-Ann-Daly_S15.counts.txt.gz
8222/suppl/GSM3998222_133-Ann-Daly_S16.counts.txt.gz

For Peer Review

8223/suppl/GSM3998223_134-Ann-Daly_S1.counts.txt.gz
8224/suppl/GSM3998224_135-Ann-Daly_S2.counts.txt.gz
8225/suppl/GSM3998225_136-Ann-Daly_S1.counts.txt.gz
8226/suppl/GSM3998226_14_472.counts.txt.gz
8227/suppl/GSM3998227_141-Ann-Daly_S2.counts.txt.gz
8228/suppl/GSM3998228_15_473.counts.txt.gz
8229/suppl/GSM3998229_152-Ann-Daly_S3.counts.txt.gz
8230/suppl/GSM3998230_155-Ann-Daly_S4.counts.txt.gz
8231/suppl/GSM3998231_169-Ann-Daly_S5.counts.txt.gz
8232/suppl/GSM3998232_17_82.counts.txt.gz
8233/suppl/GSM3998233_172-Ann-Daly_S6.counts.txt.gz
8234/suppl/GSM3998234_176-Ann-Daly_S7.counts.txt.gz
8235/suppl/GSM3998235_177-Ann-Daly_S8.counts.txt.gz
8236/suppl/GSM3998236_179-Ann-Daly_S10.counts.txt.gz
8237/suppl/GSM3998237_184-Ann-Daly_S11.counts.txt.gz
8238/suppl/GSM3998238_185-Ann-Daly_S12.counts.txt.gz
8239/suppl/GSM3998239_186-Ann-Daly_S13.counts.txt.gz
8240/suppl/GSM3998240_187-Ann-Daly_S14.counts.txt.gz
8241/suppl/GSM3998241_188-Ann-Daly_S15.counts.txt.gz
8242/suppl/GSM3998242_19_138.counts.txt.gz
8243/suppl/GSM3998243_191-Ann-Daly_S1.counts.txt.gz
8244/suppl/GSM3998244_193-Ann-Daly_S2.counts.txt.gz
8245/suppl/GSM3998245_197-Ann-Daly_S3.counts.txt.gz
8246/suppl/GSM3998246_199-Ann-Daly_S4.counts.txt.gz
8247/suppl/GSM3998247_2_23.counts.txt.gz
8248/suppl/GSM3998248_200-Ann-Daly_S5.counts.txt.gz
8249/suppl/GSM3998249_201-Ann-Daly_S6.counts.txt.gz
8250/suppl/GSM3998250_202-Ann-Daly_S7.counts.txt.gz
8251/suppl/GSM3998251_204-Ann-Daly_S8.counts.txt.gz
8252/suppl/GSM3998252_205-Ann-Daly_S9.counts.txt.gz
8253/suppl/GSM3998253_207-Ann-Daly_S10.counts.txt.gz
8254/suppl/GSM3998254_208-Ann-Daly_S11.counts.txt.gz
8255/suppl/GSM3998255_211-Ann-Daly_S13.counts.txt.gz
8256/suppl/GSM3998256_214-Ann-Daly_S15.counts.txt.gz
8257/suppl/GSM3998257_215-Ann-Daly_S16.counts.txt.gz
8258/suppl/GSM3998258_216-Ann-Daly_S1.counts.txt.gz
8259/suppl/GSM3998259_22_151.counts.txt.gz
8260/suppl/GSM3998260_23_154.counts.txt.gz
8261/suppl/GSM3998261_233-Ann-Daly_S3.counts.txt.gz
8262/suppl/GSM3998262_234-Ann-Daly_S4.counts.txt.gz
8263/suppl/GSM3998263_235-Ann-Daly_S5.counts.txt.gz
8264/suppl/GSM3998264_236-Ann-Daly_S6.counts.txt.gz
8265/suppl/GSM3998265_237-Ann-Daly_S7.counts.txt.gz
8266/suppl/GSM3998266_238-Ann-Daly_S8.counts.txt.gz
8267/suppl/GSM3998267_239-Ann-Daly_S9.counts.txt.gz
8268/suppl/GSM3998268_24_160.counts.txt.gz
8269/suppl/GSM3998269_240-Ann-Daly_S1.counts.txt.gz
8270/suppl/GSM3998270_241-Ann-Daly_S2.counts.txt.gz
8271/suppl/GSM3998271_242-Ann-Daly_S10.counts.txt.gz
8272/suppl/GSM3998272_243-Ann-Daly_S11.counts.txt.gz
8273/suppl/GSM3998273_244-Ann-Daly_S12.counts.txt.gz
8274/suppl/GSM3998274_245-Ann-Daly_S13.counts.txt.gz
8275/suppl/GSM3998275_246-Ann-Daly_S14.counts.txt.gz
8276/suppl/GSM3998276_247-Ann-Daly_S15.counts.txt.gz
8277/suppl/GSM3998277_25_164.counts.txt.gz
8278/suppl/GSM3998278_252-Ann-Daly_S16.counts.txt.gz
8279/suppl/GSM3998279_255-Ann-Daly_S3.counts.txt.gz
8280/suppl/GSM3998280_26_171.counts.txt.gz

For Peer Review

8281/suppl/GSM3998281_260-Ann-Daly_S4.counts.txt.gz
8282/suppl/GSM3998282_264-Ann-Daly_S5.counts.txt.gz
8283/suppl/GSM3998283_266-Ann-Daly_S7.counts.txt.gz
8284/suppl/GSM3998284_268-Ann-Daly_S8.counts.txt.gz
8285/suppl/GSM3998285_27_181.counts.txt.gz
8286/suppl/GSM3998286_274-Ann-Daly_S10.counts.txt.gz
8287/suppl/GSM3998287_277-Ann-Daly_S11.counts.txt.gz
8288/suppl/GSM3998288_278-Ann-Daly_S12.counts.txt.gz
8289/suppl/GSM3998289_279-Ann-Daly_S13.counts.txt.gz
8290/suppl/GSM3998290_280-Ann-Daly_S14.counts.txt.gz
8291/suppl/GSM3998291_281-Ann-Daly_S15.counts.txt.gz
8292/suppl/GSM3998292_282-Ann-Daly_S16.counts.txt.gz
8293/suppl/GSM3998293_284-Ann-Daly_S2.counts.txt.gz
8294/suppl/GSM3998294_285-Ann-Daly_S3.counts.txt.gz
8295/suppl/GSM3998295_286-Ann-Daly_S4.counts.txt.gz
8296/suppl/GSM3998296_287-Ann-Daly_S5.counts.txt.gz
8297/suppl/GSM3998297_288-Ann-Daly_S6.counts.txt.gz
8298/suppl/GSM3998298_289-Ann-Daly_S7.counts.txt.gz
8299/suppl/GSM3998299_29_474.counts.txt.gz
8300/suppl/GSM3998300_290-Ann-Daly_S8.counts.txt.gz
8301/suppl/GSM3998301_291-Ann-Daly_S9.counts.txt.gz
8302/suppl/GSM3998302_293-Ann-Daly_S10.counts.txt.gz
8303/suppl/GSM3998303_295-Ann-Daly_S11.counts.txt.gz
8304/suppl/GSM3998304_296-Ann-Daly_S12.counts.txt.gz
8305/suppl/GSM3998305_299-Ann-Daly_S13.counts.txt.gz
8306/suppl/GSM3998306_3_45.counts.txt.gz
8307/suppl/GSM3998307_300-Ann-Daly_S14.counts.txt.gz
8308/suppl/GSM3998308_303-Ann-Daly_S15.counts.txt.gz
8309/suppl/GSM3998309_304-Ann-Daly_S1.counts.txt.gz
8310/suppl/GSM3998310_306-Ann-Daly_S1.counts.txt.gz
8311/suppl/GSM3998311_31_477.counts.txt.gz
8312/suppl/GSM3998312_319-Ann-Daly_S2.counts.txt.gz
8313/suppl/GSM3998313_32_478.counts.txt.gz
8314/suppl/GSM3998314_345-Ann-Daly_S3.counts.txt.gz
8315/suppl/GSM3998315_350-Ann-Daly_S4.counts.txt.gz
8316/suppl/GSM3998316_354-Ann-Daly_S5.counts.txt.gz
8317/suppl/GSM3998317_355-Ann-Daly_S6.counts.txt.gz
8318/suppl/GSM3998318_361-Ann-Daly_S7.counts.txt.gz
8319/suppl/GSM3998319_373-Ann-Daly_S8.counts.txt.gz
8320/suppl/GSM3998320_384-Ann-Daly_S10.counts.txt.gz
8321/suppl/GSM3998321_4_67.counts.txt.gz
8322/suppl/GSM3998322_402-Ann-Daly_S11.counts.txt.gz
8323/suppl/GSM3998323_407-Ann-Daly_S13.counts.txt.gz
8324/suppl/GSM3998324_408-Ann-Daly_S14.counts.txt.gz
8325/suppl/GSM3998325_416-Ann-Daly_S2.counts.txt.gz
8326/suppl/GSM3998326_418-Ann-Daly_S15.counts.txt.gz
8327/suppl/GSM3998327_424-Ann-Daly_S16.counts.txt.gz
8328/suppl/GSM3998328_425-Ann-Daly_S3.counts.txt.gz
8329/suppl/GSM3998329_431-Ann-Daly_S4.counts.txt.gz
8330/suppl/GSM3998330_443-Ann-Daly_S5.counts.txt.gz
8331/suppl/GSM3998331_5_74.counts.txt.gz
8332/suppl/GSM3998332_6_139.counts.txt.gz
8333/suppl/GSM3998333_7_162.counts.txt.gz
8334/suppl/GSM3998334_8_344.counts.txt.gz
8335/suppl/GSM3998335_84-Ann-Daly_S9.counts.txt.gz
8336/suppl/GSM3998336_85-Ann-Daly_S10.counts.txt.gz
8337/suppl/GSM3998337_9_346.counts.txt.gz
8338/suppl/GSM3998338_98-Ann-Daly_S13.counts.txt.gz

For Peer Review

8339/suppl/GSM3998339_s5_Olivier-Govaere_S5.counts.txt.gz
8340/suppl/GSM3998340_s2_Olivier-Govaere_S2.counts.txt.gz
8341/suppl/GSM3998341_078-Ann-Daly_S14.counts.txt.gz
8342/suppl/GSM3998342_085-Ann-Daly_S16.counts.txt.gz
8343/suppl/GSM3998343_C100-Ann-Daly_S1.counts.txt.gz
8344/suppl/GSM3998344_C101-Ann-Daly_S2.counts.txt.gz
8345/suppl/GSM3998345_C102-Ann-Daly_S3.counts.txt.gz
8346/suppl/GSM3998346_C103-Ann-Daly_S4.counts.txt.gz
8347/suppl/GSM3998347_C107-Ann-Daly_S5.counts.txt.gz
8348/suppl/GSM3998348_C108-Ann-Daly_S6.counts.txt.gz
8349/suppl/GSM3998349_C109-Ann-Daly_S7.counts.txt.gz
8350/suppl/GSM3998350_C110-Ann-Daly_S8.counts.txt.gz
8351/suppl/GSM3998351_C122-Ann-Daly_S10.counts.txt.gz
8352/suppl/GSM3998352_C123-Ann-Daly_S11.counts.txt.gz
8353/suppl/GSM3998353_C124-Ann-Daly_S12.counts.txt.gz
8354/suppl/GSM3998354_C125-Ann-Daly_S13.counts.txt.gz
8355/suppl/GSM3998355_C126-Ann-Daly_S14.counts.txt.gz
8356/suppl/GSM3998356_C128-Ann-Daly_S1.counts.txt.gz
8357/suppl/GSM3998357_C129-Ann-Daly_S2.counts.txt.gz
8358/suppl/GSM3998358_C131-Ann-Daly_S3.counts.txt.gz
8359/suppl/GSM3998359_C133-Ann-Daly_S4.counts.txt.gz
8360/suppl/GSM3998360_C134_S5.counts.txt.gz
8361/suppl/GSM3998361_C135-Ann-Daly_S6.counts.txt.gz
8362/suppl/GSM3998362_C136-Ann-Daly_S7.counts.txt.gz
8363/suppl/GSM3998363_C137-Ann-Daly_S8.counts.txt.gz
8364/suppl/GSM3998364_C139-Ann-Daly_S9.counts.txt.gz
8365/suppl/GSM3998365_C143-Ann-Daly_S11.counts.txt.gz
8366/suppl/GSM3998366_C145-Ann-Daly_S12.counts.txt.gz
8367/suppl/GSM3998367_C150-Ann-Daly_S13.counts.txt.gz
8368/suppl/GSM3998368_C171-Ann-Daly_S16.counts.txt.gz
8369/suppl/GSM3998369_C173-Ann-Daly_S16.counts.txt.gz
8370/suppl/GSM3998370_C18-Ann-Daly_S3.counts.txt.gz
8371/suppl/GSM3998371_C29-Ann-Daly_S4.counts.txt.gz
8372/suppl/GSM3998372_C61-Ann-Daly_S5.counts.txt.gz
8373/suppl/GSM3998373_C68-Ann-Daly_S6.counts.txt.gz
8374/suppl/GSM3998374_C69-Ann-Daly_S7.counts.txt.gz
8375/suppl/GSM3998375_C73-Ann-Daly_S8.counts.txt.gz
8376/suppl/GSM3998376_C76-Ann-Daly_S9.counts.txt.gz
8377/suppl/GSM3998377_C81-Ann-Daly_S10.counts.txt.gz
8378/suppl/GSM3998378_C82-Ann-Daly_S11.counts.txt.gz
8379/suppl/GSM3998379_C90-Ann-Daly_S12.counts.txt.gz
8380/suppl/GSM3998380_C93-Ann-Daly_S13.counts.txt.gz
8381/suppl/GSM3998381_C94-Ann-Daly_S15.counts.txt.gz
8382/suppl/GSM3998382_C99-Ann-Daly_S16.counts.txt.gz

For Peer Review

Table S1c. Sample Information of GSE162694

!Sample_t	!Sample_g	!Sample_s	!Sample_s	!Sample_l	!Sample_t	!Sample_c	!Sample_s	!Sample_o
nash1_F0	GSM495732	Public on	Dec 04 20	Jun 11 20	SRA	1	Liver bio	Homo sapi
nash10_F1	GSM495732	Public on	Dec 04 20	Jun 11 20	SRA	1	Liver bio	Homo sapi
nash100_N	GSM495732	Public on	Dec 04 20	Jun 11 20	SRA	1	Liver bio	Homo sapi
nash101_F	GSM495732	Public on	Dec 04 20	Jun 11 20	SRA	1	Liver bio	Homo sapi
nash102_F	GSM495732	Public on	Dec 04 20	Jun 11 20	SRA	1	Liver bio	Homo sapi
nash103_N	GSM495732	Public on	Dec 04 20	Jun 11 20	SRA	1	Liver bio	Homo sapi
nash10_F0	GSM495732	Public on	Dec 04 20	Jun 11 20	SRA	1	Liver bio	Homo sapi
nash10_F2	GSM495732	Public on	Dec 04 20	Jun 11 20	SRA	1	Liver bio	Homo sapi
nash106_F	GSM495732	Public on	Dec 04 20	Jun 11 20	SRA	1	Liver bio	Homo sapi
nash10_N	GSM495733	Public on	Dec 04 20	Jun 11 20	SRA	1	Liver bio	Homo sapi
nash109_N	GSM495733	Public on	Dec 04 20	Jun 11 20	SRA	1	Liver bio	Homo sapi
nash11_F1	GSM495733	Public on	Dec 04 20	Jun 11 20	SRA	1	Liver bio	Homo sapi
nash110_F	GSM495733	Public on	Dec 04 20	Jun 11 20	SRA	1	Liver bio	Homo sapi
nash111_F	GSM495733	Public on	Dec 04 20	Jun 11 20	SRA	1	Liver bio	Homo sapi
nash1119_	GSM495733	Public on	Dec 04 20	Jun 11 20	SRA	1	Liver bio	Homo sapi
nash112_N	GSM495733	Public on	Dec 04 20	Jun 11 20	SRA	1	Liver bio	Homo sapi
nash1120_	GSM495733	Public on	Dec 04 20	Jun 11 20	SRA	1	Liver bio	Homo sapi
nash1121_	GSM495733	Public on	Dec 04 20	Jun 11 20	SRA	1	Liver bio	Homo sapi
nash1122_	GSM495733	Public on	Dec 04 20	Jun 11 20	SRA	1	Liver bio	Homo sapi
nash1123_	GSM495734	Public on	Dec 04 20	Jun 11 20	SRA	1	Liver bio	Homo sapi
nash112_F	GSM495734	Public on	Dec 04 20	Jun 11 20	SRA	1	Liver bio	Homo sapi
nash112_F	GSM495734	Public on	Dec 04 20	Jun 11 20	SRA	1	Liver bio	Homo sapi
nash1126_	GSM495734	Public on	Dec 04 20	Jun 11 20	SRA	1	Liver bio	Homo sapi
nash1127_	GSM495734	Public on	Dec 04 20	Jun 11 20	SRA	1	Liver bio	Homo sapi
nash112_F	GSM495734	Public on	Dec 04 20	Jun 11 20	SRA	1	Liver bio	Homo sapi
nash1129_	GSM495734	Public on	Dec 04 20	Jun 11 20	SRA	1	Liver bio	Homo sapi
nash113_F	GSM495734	Public on	Dec 04 20	Jun 11 20	SRA	1	Liver bio	Homo sapi
nash1130_	GSM495734	Public on	Dec 04 20	Jun 11 20	SRA	1	Liver bio	Homo sapi
nash1131_	GSM495734	Public on	Dec 04 20	Jun 11 20	SRA	1	Liver bio	Homo sapi
nash1132_	GSM495735	Public on	Dec 04 20	Jun 11 20	SRA	1	Liver bio	Homo sapi
nash1133_	GSM495735	Public on	Dec 04 20	Jun 11 20	SRA	1	Liver bio	Homo sapi
nash113_F	GSM495735	Public on	Dec 04 20	Jun 11 20	SRA	1	Liver bio	Homo sapi
nash113_F	GSM495735	Public on	Dec 04 20	Jun 11 20	SRA	1	Liver bio	Homo sapi
nash1136_	GSM495735	Public on	Dec 04 20	Jun 11 20	SRA	1	Liver bio	Homo sapi
nash1137_	GSM495735	Public on	Dec 04 20	Jun 11 20	SRA	1	Liver bio	Homo sapi
nash113_F	GSM495735	Public on	Dec 04 20	Jun 11 20	SRA	1	Liver bio	Homo sapi
nash1139_	GSM495735	Public on	Dec 04 20	Jun 11 20	SRA	1	Liver bio	Homo sapi
nash11_N	GSM495735	Public on	Dec 04 20	Jun 11 20	SRA	1	Liver bio	Homo sapi
nash11_F1	GSM495735	Public on	Dec 04 20	Jun 11 20	SRA	1	Liver bio	Homo sapi
nash116_F	GSM495736	Public on	Dec 04 20	Jun 11 20	SRA	1	Liver bio	Homo sapi
nash117_F	GSM495736	Public on	Dec 04 20	Jun 11 20	SRA	1	Liver bio	Homo sapi
nash12_F2	GSM495736	Public on	Dec 04 20	Jun 11 20	SRA	1	Liver bio	Homo sapi
nash13_F0	GSM495736	Public on	Dec 04 20	Jun 11 20	SRA	1	Liver bio	Homo sapi
nash1_F0	GSM495736	Public on	Dec 04 20	Jun 11 20	SRA	1	Liver bio	Homo sapi
nash143_F	GSM495736	Public on	Dec 04 20	Jun 11 20	SRA	1	Liver bio	Homo sapi
nash1_F4	GSM495736	Public on	Dec 04 20	Jun 11 20	SRA	1	Liver bio	Homo sapi
nash1_F1	GSM495736	Public on	Dec 04 20	Jun 11 20	SRA	1	Liver bio	Homo sapi
nash146_F	GSM495736	Public on	Dec 04 20	Jun 11 20	SRA	1	Liver bio	Homo sapi
nash1_F0	GSM495736	Public on	Dec 04 20	Jun 11 20	SRA	1	Liver bio	Homo sapi
nash149_F	GSM495737	Public on	Dec 04 20	Jun 11 20	SRA	1	Liver bio	Homo sapi
nash1_F0	GSM495737	Public on	Dec 04 20	Jun 11 20	SRA	1	Liver bio	Homo sapi
nash150_F	GSM495737	Public on	Dec 04 20	Jun 11 20	SRA	1	Liver bio	Homo sapi
nash151_F	GSM495737	Public on	Dec 04 20	Jun 11 20	SRA	1	Liver bio	Homo sapi
nash153_F	GSM495737	Public on	Dec 04 20	Jun 11 20	SRA	1	Liver bio	Homo sapi
nash1_F0	GSM495737	Public on	Dec 04 20	Jun 11 20	SRA	1	Liver bio	Homo sapi
nash1_F1	GSM495737	Public on	Dec 04 20	Jun 11 20	SRA	1	Liver bio	Homo sapi

For Peer Review

nash156_F	GSM495737	Public	on	Dec	04	20	Jun	11	20	SRA	1	Liver	bio	Homo	sapi
nash157_F	GSM495737	Public	on	Dec	04	20	Jun	11	20	SRA	1	Liver	bio	Homo	sapi
nash1_F0	GSM495737	Public	on	Dec	04	20	Jun	11	20	SRA	1	Liver	bio	Homo	sapi
nash159_F	GSM495738	Public	on	Dec	04	20	Jun	11	20	SRA	1	Liver	bio	Homo	sapi
nash16_F1	GSM495738	Public	on	Dec	04	20	Jun	11	20	SRA	1	Liver	bio	Homo	sapi
nash160_F	GSM495738	Public	on	Dec	04	20	Jun	11	20	SRA	1	Liver	bio	Homo	sapi
nash17_F1	GSM495738	Public	on	Dec	04	20	Jun	11	20	SRA	1	Liver	bio	Homo	sapi
nash19_F2	GSM495738	Public	on	Dec	04	20	Jun	11	20	SRA	1	Liver	bio	Homo	sapi
nash2_F2	GSM495738	Public	on	Dec	04	20	Jun	11	20	SRA	1	Liver	bio	Homo	sapi
nash21_F0	GSM495738	Public	on	Dec	04	20	Jun	11	20	SRA	1	Liver	bio	Homo	sapi
nash22_F1	GSM495738	Public	on	Dec	04	20	Jun	11	20	SRA	1	Liver	bio	Homo	sapi
nash23_F0	GSM495738	Public	on	Dec	04	20	Jun	11	20	SRA	1	Liver	bio	Homo	sapi
nash2_F0	GSM495738	Public	on	Dec	04	20	Jun	11	20	SRA	1	Liver	bio	Homo	sapi
nash2_F2	GSM495739	Public	on	Dec	04	20	Jun	11	20	SRA	1	Liver	bio	Homo	sapi
nash26_N	GSM495739	Public	on	Dec	04	20	Jun	11	20	SRA	1	Liver	bio	Homo	sapi
nash27_F1	GSM495739	Public	on	Dec	04	20	Jun	11	20	SRA	1	Liver	bio	Homo	sapi
nash2_F1	GSM495739	Public	on	Dec	04	20	Jun	11	20	SRA	1	Liver	bio	Homo	sapi
nash29_F0	GSM495739	Public	on	Dec	04	20	Jun	11	20	SRA	1	Liver	bio	Homo	sapi
nash3_F4	GSM495739	Public	on	Dec	04	20	Jun	11	20	SRA	1	Liver	bio	Homo	sapi
nash30_F2	GSM495739	Public	on	Dec	04	20	Jun	11	20	SRA	1	Liver	bio	Homo	sapi
nash31_F0	GSM495739	Public	on	Dec	04	20	Jun	11	20	SRA	1	Liver	bio	Homo	sapi
nash32_N	GSM495739	Public	on	Dec	04	20	Jun	11	20	SRA	1	Liver	bio	Homo	sapi
nash33_F0	GSM495739	Public	on	Dec	04	20	Jun	11	20	SRA	1	Liver	bio	Homo	sapi
nash3_F2	GSM495740	Public	on	Dec	04	20	Jun	11	20	SRA	1	Liver	bio	Homo	sapi
nash3_F0	GSM495740	Public	on	Dec	04	20	Jun	11	20	SRA	1	Liver	bio	Homo	sapi
nash36_N	GSM495740	Public	on	Dec	04	20	Jun	11	20	SRA	1	Liver	bio	Homo	sapi
nash37_F3	GSM495740	Public	on	Dec	04	20	Jun	11	20	SRA	1	Liver	bio	Homo	sapi
nash3_N 5	GSM495740	Public	on	Dec	04	20	Jun	11	20	SRA	1	Liver	bio	Homo	sapi
nash39_F0	GSM495740	Public	on	Dec	04	20	Jun	11	20	SRA	1	Liver	bio	Homo	sapi
nash40_N	GSM495740	Public	on	Dec	04	20	Jun	11	20	SRA	1	Liver	bio	Homo	sapi
nash41_F3	GSM495740	Public	on	Dec	04	20	Jun	11	20	SRA	1	Liver	bio	Homo	sapi
nash_F0 5	GSM495740	Public	on	Dec	04	20	Jun	11	20	SRA	1	Liver	bio	Homo	sapi
nash_N 54	GSM495740	Public	on	Dec	04	20	Jun	11	20	SRA	1	Liver	bio	Homo	sapi
nash46_F0	GSM495741	Public	on	Dec	04	20	Jun	11	20	SRA	1	Liver	bio	Homo	sapi
nash47_F1	GSM495741	Public	on	Dec	04	20	Jun	11	20	SRA	1	Liver	bio	Homo	sapi
nash_F1 5	GSM495741	Public	on	Dec	04	20	Jun	11	20	SRA	1	Liver	bio	Homo	sapi
nash49_N	GSM495741	Public	on	Dec	04	20	Jun	11	20	SRA	1	Liver	bio	Homo	sapi
nash_F0 5	GSM495741	Public	on	Dec	04	20	Jun	11	20	SRA	1	Liver	bio	Homo	sapi
nash51_F2	GSM495741	Public	on	Dec	04	20	Jun	11	20	SRA	1	Liver	bio	Homo	sapi
nash52_F0	GSM495741	Public	on	Dec	04	20	Jun	11	20	SRA	1	Liver	bio	Homo	sapi
nash53_F1	GSM495741	Public	on	Dec	04	20	Jun	11	20	SRA	1	Liver	bio	Homo	sapi
nash_N 54	GSM495741	Public	on	Dec	04	20	Jun	11	20	SRA	1	Liver	bio	Homo	sapi
nash_F1 5	GSM495741	Public	on	Dec	04	20	Jun	11	20	SRA	1	Liver	bio	Homo	sapi
nash56_F1	GSM495742	Public	on	Dec	04	20	Jun	11	20	SRA	1	Liver	bio	Homo	sapi
nash57_N	GSM495742	Public	on	Dec	04	20	Jun	11	20	SRA	1	Liver	bio	Homo	sapi
nash_F0 5	GSM495742	Public	on	Dec	04	20	Jun	11	20	SRA	1	Liver	bio	Homo	sapi
nash59_N	GSM495742	Public	on	Dec	04	20	Jun	11	20	SRA	1	Liver	bio	Homo	sapi
nash6_F1	GSM495742	Public	on	Dec	04	20	Jun	11	20	SRA	1	Liver	bio	Homo	sapi
nash60_N	GSM495742	Public	on	Dec	04	20	Jun	11	20	SRA	1	Liver	bio	Homo	sapi
nash61_F1	GSM495742	Public	on	Dec	04	20	Jun	11	20	SRA	1	Liver	bio	Homo	sapi
nash62_N	GSM495742	Public	on	Dec	04	20	Jun	11	20	SRA	1	Liver	bio	Homo	sapi
nash63_F0	GSM495742	Public	on	Dec	04	20	Jun	11	20	SRA	1	Liver	bio	Homo	sapi
nash6_N 5	GSM495742	Public	on	Dec	04	20	Jun	11	20	SRA	1	Liver	bio	Homo	sapi
nash6_N 5	GSM495743	Public	on	Dec	04	20	Jun	11	20	SRA	1	Liver	bio	Homo	sapi
nash66_F1	GSM495743	Public	on	Dec	04	20	Jun	11	20	SRA	1	Liver	bio	Homo	sapi
nash67_F2	GSM495743	Public	on	Dec	04	20	Jun	11	20	SRA	1	Liver	bio	Homo	sapi
nash6_F2	GSM495743	Public	on	Dec	04	20	Jun	11	20	SRA	1	Liver	bio	Homo	sapi
nash69_N	GSM495743	Public	on	Dec	04	20	Jun	11	20	SRA	1	Liver	bio	Homo	sapi

For Peer Review

nash7_N 5	GSM495743	Public	on	Dec	04	20	Jun	11	20	SRA	1	Liver	bio	Homo	sapi
nash70_F0	GSM495743	Public	on	Dec	04	20	Jun	11	20	SRA	1	Liver	bio	Homo	sapi
nash71_N	GSM495743	Public	on	Dec	04	20	Jun	11	20	SRA	1	Liver	bio	Homo	sapi
nash72_N	GSM495743	Public	on	Dec	04	20	Jun	11	20	SRA	1	Liver	bio	Homo	sapi
nash73_N	GSM495743	Public	on	Dec	04	20	Jun	11	20	SRA	1	Liver	bio	Homo	sapi
nash7_N 5	GSM495744	Public	on	Dec	04	20	Jun	11	20	SRA	1	Liver	bio	Homo	sapi
nash76_N	GSM495744	Public	on	Dec	04	20	Jun	11	20	SRA	1	Liver	bio	Homo	sapi
nash77_F0	GSM495744	Public	on	Dec	04	20	Jun	11	20	SRA	1	Liver	bio	Homo	sapi
nash7_F1	GSM495744	Public	on	Dec	04	20	Jun	11	20	SRA	1	Liver	bio	Homo	sapi
nash79_N	GSM495744	Public	on	Dec	04	20	Jun	11	20	SRA	1	Liver	bio	Homo	sapi
nash80_F2	GSM495744	Public	on	Dec	04	20	Jun	11	20	SRA	1	Liver	bio	Homo	sapi
nash81_F2	GSM495744	Public	on	Dec	04	20	Jun	11	20	SRA	1	Liver	bio	Homo	sapi
nash82_F1	GSM495744	Public	on	Dec	04	20	Jun	11	20	SRA	1	Liver	bio	Homo	sapi
nash83_F2	GSM495744	Public	on	Dec	04	20	Jun	11	20	SRA	1	Liver	bio	Homo	sapi
nash_F0 5	GSM495744	Public	on	Dec	04	20	Jun	11	20	SRA	1	Liver	bio	Homo	sapi
nash_F0 5	GSM495745	Public	on	Dec	04	20	Jun	11	20	SRA	1	Liver	bio	Homo	sapi
nash86_F4	GSM495745	Public	on	Dec	04	20	Jun	11	20	SRA	1	Liver	bio	Homo	sapi
nash87_F0	GSM495745	Public	on	Dec	04	20	Jun	11	20	SRA	1	Liver	bio	Homo	sapi
nash_N 54	GSM495745	Public	on	Dec	04	20	Jun	11	20	SRA	1	Liver	bio	Homo	sapi
nash89_F1	GSM495745	Public	on	Dec	04	20	Jun	11	20	SRA	1	Liver	bio	Homo	sapi
nash9_F1	GSM495745	Public	on	Dec	04	20	Jun	11	20	SRA	1	Liver	bio	Homo	sapi
nash90_N	GSM495745	Public	on	Dec	04	20	Jun	11	20	SRA	1	Liver	bio	Homo	sapi
nash91_F2	GSM495745	Public	on	Dec	04	20	Jun	11	20	SRA	1	Liver	bio	Homo	sapi
nash92_F3	GSM495745	Public	on	Dec	04	20	Jun	11	20	SRA	1	Liver	bio	Homo	sapi
nash93_F0	GSM495745	Public	on	Dec	04	20	Jun	11	20	SRA	1	Liver	bio	Homo	sapi
nash9_F2	GSM495746	Public	on	Dec	04	20	Jun	11	20	SRA	1	Liver	bio	Homo	sapi
nash96_F0	GSM495746	Public	on	Dec	04	20	Jun	11	20	SRA	1	Liver	bio	Homo	sapi
nash9_F1	GSM495746	Public	on	Dec	04	20	Jun	11	20	SRA	1	Liver	bio	Homo	sapi
nash99_N	GSM495746	Public	on	Dec	04	20	Jun	11	20	SRA	1	Liver	bio	Homo	sapi

For Peer Review

!Sample_c	!Sample_c	!Sample_c	!Sample_c	!Sample_c	!Sample_m	!Sample_e	!Sample_t	!Sample_d
tissue: L	age: 36	Sex: Male	fibrosis	nas score	total RNA	none prov	9606	nash1_F0
tissue: L	age: 55	Sex: Fema	fibrosis	nas score	total RNA	none prov	9606	nash10_F1
tissue: L	age: 25	Sex: Fema	fibrosis	nas score	total RNA	none prov	9606	nash100_N
tissue: L	age: 46	Sex: Fema	fibrosis	nas score	total RNA	none prov	9606	nash101_F
tissue: L	age: 47	Sex: Male	fibrosis	nas score	total RNA	none prov	9606	nash102_F
tissue: L	age: 54	Sex: Fema	fibrosis	nas score	total RNA	none prov	9606	nash103_N
tissue: L	age: 65	Sex: Male	fibrosis	nas score	total RNA	none prov	9606	nash10_F0
tissue: L	age: 45	Sex: Male	fibrosis	nas score	total RNA	none prov	9606	nash10_F2
tissue: L	age: 22	Sex: Fema	fibrosis	nas score	total RNA	none prov	9606	nash106_F
tissue: L	age: 55	Sex: Fema	fibrosis	nas score	total RNA	none prov	9606	nash10_N
tissue: L	age: 34	Sex: Fema	fibrosis	nas score	total RNA	none prov	9606	nash109_N
tissue: L	age: 25	Sex: Male	fibrosis	nas score	total RNA	none prov	9606	nash11_F1
tissue: L	age: 59	Sex: Fema	fibrosis	nas score	total RNA	none prov	9606	nash110_F
tissue: L	age: 61	Sex: Fema	fibrosis	nas score	total RNA	none prov	9606	nash111_F
tissue: L	age: 59	Sex: Fema	fibrosis	nas score	total RNA	none prov	9606	nash1119_
tissue: L	age: 35	Sex: Fema	fibrosis	nas score	total RNA	none prov	9606	nash112_N
tissue: L	age: 68	Sex: Fema	fibrosis	nas score	total RNA	none prov	9606	nash1120_
tissue: L	age: 64	Sex: Fema	fibrosis	nas score	total RNA	none prov	9606	nash1121_
tissue: L	age: 55	Sex: Male	fibrosis	nas score	total RNA	none prov	9606	nash1122_
tissue: L	age: 56	Sex: Fema	fibrosis	nas score	total RNA	none prov	9606	nash1123_
tissue: L	age: 69	Sex: Male	fibrosis	nas score	total RNA	none prov	9606	nash112_F
tissue: L	age: 48	Sex: Male	fibrosis	nas score	total RNA	none prov	9606	nash112_F
tissue: L	age: 49	Sex: Fema	fibrosis	nas score	total RNA	none prov	9606	nash1126_
tissue: L	age: 46	Sex: Fema	fibrosis	nas score	total RNA	none prov	9606	nash1127_
tissue: L	age: 50	Sex: Fema	fibrosis	nas score	total RNA	none prov	9606	nash112_F
tissue: L	age: 18	Sex: Fema	fibrosis	nas score	total RNA	none prov	9606	nash1129_
tissue: L	age: 34	Sex: Male	fibrosis	nas score	total RNA	none prov	9606	nash113_F
tissue: L	age: 57	Sex: Fema	fibrosis	nas score	total RNA	none prov	9606	nash1130_
tissue: L	age: 65	Sex: Fema	fibrosis	nas score	total RNA	none prov	9606	nash1131_
tissue: L	age: 59	Sex: Male	fibrosis	nas score	total RNA	none prov	9606	nash1132_
tissue: L	age: 64	Sex: Male	fibrosis	nas score	total RNA	none prov	9606	nash1133_
tissue: L	age: 51	Sex: Male	fibrosis	nas score	total RNA	none prov	9606	nash113_F
tissue: L	age: 35	Sex: Fema	fibrosis	nas score	total RNA	none prov	9606	nash113_F
tissue: L	age: 51	Sex: Fema	fibrosis	nas score	total RNA	none prov	9606	nash1136_
tissue: L	age: 61	Sex: Male	fibrosis	nas score	total RNA	none prov	9606	nash1137_
tissue: L	age: 62	Sex: Male	fibrosis	nas score	total RNA	none prov	9606	nash113_F
tissue: L	age: 54	Sex: Male	fibrosis	nas score	total RNA	none prov	9606	nash1139_
tissue: L	age: 58	Sex: Fema	fibrosis	nas score	total RNA	none prov	9606	nash11_N
tissue: L	age: 58	Sex: Male	fibrosis	nas score	total RNA	none prov	9606	nash11_F1
tissue: L	age: 64	Sex: Male	fibrosis	nas score	total RNA	none prov	9606	nash116_F
tissue: L	age: 22	Sex: Fema	fibrosis	nas score	total RNA	none prov	9606	nash117_F
tissue: L	age: 32	Sex: Fema	fibrosis	nas score	total RNA	none prov	9606	nash12_F2
tissue: L	age: 48	Sex: Fema	fibrosis	nas score	total RNA	none prov	9606	nash13_F0
tissue: L	age: 32	Sex: Male	fibrosis	nas score	total RNA	none prov	9606	nash1_F0
tissue: L	age: 37	Sex: Fema	fibrosis	nas score	total RNA	none prov	9606	nash143_F
tissue: L	age: 59	Sex: Fema	fibrosis	nas score	total RNA	none prov	9606	nash1_F4
tissue: L	age: 41	Sex: Fema	fibrosis	nas score	total RNA	none prov	9606	nash1_F1
tissue: L	age: 56	Sex: Fema	fibrosis	nas score	total RNA	none prov	9606	nash146_F
tissue: L	age: 42	Sex: Fema	fibrosis	nas score	total RNA	none prov	9606	nash1_F0
tissue: L	age: 26	Sex: Fema	fibrosis	nas score	total RNA	none prov	9606	nash149_F
tissue: L	age: 35	Sex: Male	fibrosis	nas score	total RNA	none prov	9606	nash1_F0
tissue: L	age: 53	Sex: Fema	fibrosis	nas score	total RNA	none prov	9606	nash150_F
tissue: L	age: 45	Sex: Fema	fibrosis	nas score	total RNA	none prov	9606	nash151_F
tissue: L	age: 55	Sex: Fema	fibrosis	nas score	total RNA	none prov	9606	nash153_F
tissue: L	age: 52	Sex: Fema	fibrosis	nas score	total RNA	none prov	9606	nash1_F0
tissue: L	age: 25	Sex: Male	fibrosis	nas score	total RNA	none prov	9606	nash1_F1

For Peer Review

tissue: L	age: 44	Sex: Male	fibrosis	nas	score	total	RNA	none	prov	9606	nash156_F
tissue: L	age: 51	Sex: Male	fibrosis	nas	score	total	RNA	none	prov	9606	nash157_F
tissue: L	age: 28	Sex: Fema	fibrosis	nas	score	total	RNA	none	prov	9606	nash1_F0
tissue: L	age: 57	Sex: Fema	fibrosis	nas	score	total	RNA	none	prov	9606	nash159_F
tissue: L	age: 37	Sex: Male	fibrosis	nas	score	total	RNA	none	prov	9606	nash16_F1
tissue: L	age: 57	Sex: Fema	fibrosis	nas	score	total	RNA	none	prov	9606	nash160_F
tissue: L	age: 66	Sex: Fema	fibrosis	nas	score	total	RNA	none	prov	9606	nash17_F1
tissue: L	age: 42	Sex: Male	fibrosis	nas	score	total	RNA	none	prov	9606	nash19_F2
tissue: L	age: 57	Sex: Fema	fibrosis	nas	score	total	RNA	none	prov	9606	nash2_F2
tissue: L	age: 65	Sex: Male	fibrosis	nas	score	total	RNA	none	prov	9606	nash21_F0
tissue: L	age: 54	Sex: Male	fibrosis	nas	score	total	RNA	none	prov	9606	nash22_F1
tissue: L	age: 53	Sex: Fema	fibrosis	nas	score	total	RNA	none	prov	9606	nash23_F0
tissue: L	age: 20	Sex: Fema	fibrosis	nas	score	total	RNA	none	prov	9606	nash2_F0
tissue: L	age: 20	Sex: Fema	fibrosis	nas	score	total	RNA	none	prov	9606	nash2_F2
tissue: L	age: 61	Sex: Fema	fibrosis	nas	score	total	RNA	none	prov	9606	nash26_N
tissue: L	age: 40	Sex: Male	fibrosis	nas	score	total	RNA	none	prov	9606	nash27_F1
tissue: L	age: 64	Sex: Fema	fibrosis	nas	score	total	RNA	none	prov	9606	nash2_F1
tissue: L	age: 30	Sex: Fema	fibrosis	nas	score	total	RNA	none	prov	9606	nash29_F0
tissue: L	age: 61	Sex: Male	fibrosis	nas	score	total	RNA	none	prov	9606	nash3_F4
tissue: L	age: 25	Sex: Fema	fibrosis	nas	score	total	RNA	none	prov	9606	nash30_F2
tissue: L	age: 54	Sex: Fema	fibrosis	nas	score	total	RNA	none	prov	9606	nash31_F0
tissue: L	age: 50	Sex: Male	fibrosis	nas	score	total	RNA	none	prov	9606	nash32_N
tissue: L	age: 28	Sex: Fema	fibrosis	nas	score	total	RNA	none	prov	9606	nash33_F0
tissue: L	age: 33	Sex: Fema	fibrosis	nas	score	total	RNA	none	prov	9606	nash3_F2
tissue: L	age: 72	Sex: Fema	fibrosis	nas	score	total	RNA	none	prov	9606	nash3_F0
tissue: L	age: 44	Sex: Fema	fibrosis	nas	score	total	RNA	none	prov	9606	nash36_N
tissue: L	age: 45	Sex: Fema	fibrosis	nas	score	total	RNA	none	prov	9606	nash37_F3
tissue: L	age: 28	Sex: Fema	fibrosis	nas	score	total	RNA	none	prov	9606	nash3_N
tissue: L	age: 50	Sex: Male	fibrosis	nas	score	total	RNA	none	prov	9606	nash39_F0
tissue: L	age: 22	Sex: Fema	fibrosis	nas	score	total	RNA	none	prov	9606	nash40_N
tissue: L	age: 32	Sex: Male	fibrosis	nas	score	total	RNA	none	prov	9606	nash41_F3
tissue: L	age: 53	Sex: Fema	fibrosis	nas	score	total	RNA	none	prov	9606	nash_F0
tissue: L	age: 37	Sex: Fema	fibrosis	nas	score	total	RNA	none	prov	9606	nash_N
tissue: L	age: 41	Sex: Fema	fibrosis	nas	score	total	RNA	none	prov	9606	nash46_F0
tissue: L	age: 60	Sex: Fema	fibrosis	nas	score	total	RNA	none	prov	9606	nash47_F1
tissue: L	age: 49	Sex: Fema	fibrosis	nas	score	total	RNA	none	prov	9606	nash_F1
tissue: L	age: 47	Sex: Fema	fibrosis	nas	score	total	RNA	none	prov	9606	nash49_N
tissue: L	age: 40	Sex: Fema	fibrosis	nas	score	total	RNA	none	prov	9606	nash_F0
tissue: L	age: 34	Sex: Fema	fibrosis	nas	score	total	RNA	none	prov	9606	nash51_F2
tissue: L	age: 25	Sex: Fema	fibrosis	nas	score	total	RNA	none	prov	9606	nash52_F0
tissue: L	age: 32	Sex: Fema	fibrosis	nas	score	total	RNA	none	prov	9606	nash53_F1
tissue: L	age: 44	Sex: Fema	fibrosis	nas	score	total	RNA	none	prov	9606	nash_N
tissue: L	age: 34	Sex: Fema	fibrosis	nas	score	total	RNA	none	prov	9606	nash_F1
tissue: L	age: 62	Sex: Fema	fibrosis	nas	score	total	RNA	none	prov	9606	nash56_F1
tissue: L	age: 60	Sex: Fema	fibrosis	nas	score	total	RNA	none	prov	9606	nash57_N
tissue: L	age: 46	Sex: Fema	fibrosis	nas	score	total	RNA	none	prov	9606	nash_F0
tissue: L	age: 47	Sex: Fema	fibrosis	nas	score	total	RNA	none	prov	9606	nash59_N
tissue: L	age: 42	Sex: Male	fibrosis	nas	score	total	RNA	none	prov	9606	nash6_F1
tissue: L	age: 28	Sex: Fema	fibrosis	nas	score	total	RNA	none	prov	9606	nash60_N
tissue: L	age: 56	Sex: Fema	fibrosis	nas	score	total	RNA	none	prov	9606	nash61_F1
tissue: L	age: 29	Sex: Fema	fibrosis	nas	score	total	RNA	none	prov	9606	nash62_N
tissue: L	age: 49	Sex: Fema	fibrosis	nas	score	total	RNA	none	prov	9606	nash63_F0
tissue: L	age: 49	Sex: Fema	fibrosis	nas	score	total	RNA	none	prov	9606	nash6_N
tissue: L	age: 41	Sex: Fema	fibrosis	nas	score	total	RNA	none	prov	9606	nash6_N
tissue: L	age: 46	Sex: Fema	fibrosis	nas	score	total	RNA	none	prov	9606	nash66_F1
tissue: L	age: 60	Sex: Fema	fibrosis	nas	score	total	RNA	none	prov	9606	nash67_F2
tissue: L	age: 43	Sex: Fema	fibrosis	nas	score	total	RNA	none	prov	9606	nash6_F2
tissue: L	age: 54	Sex: Fema	fibrosis	nas	score	total	RNA	none	prov	9606	nash69_N

For Peer Review

tissue: L	age: 51	Sex: Fema	fibrosis	nas	score	total	RNA	none	prov	9606 nash7_N
tissue: L	age: 39	Sex: Fema	fibrosis	nas	score	total	RNA	none	prov	9606 nash70_F0
tissue: L	age: 37	Sex: Fema	fibrosis	nas	score	total	RNA	none	prov	9606 nash71_N
tissue: L	age: 57	Sex: Fema	fibrosis	nas	score	total	RNA	none	prov	9606 nash72_N
tissue: L	age: 49	Sex: Fema	fibrosis	nas	score	total	RNA	none	prov	9606 nash73_N
tissue: L	age: 33	Sex: Fema	fibrosis	nas	score	total	RNA	none	prov	9606 nash7_N
tissue: L	age: 32	Sex: Fema	fibrosis	nas	score	total	RNA	none	prov	9606 nash76_N
tissue: L	age: 53	Sex: Fema	fibrosis	nas	score	total	RNA	none	prov	9606 nash77_F0
tissue: L	age: 21	Sex: Fema	fibrosis	nas	score	total	RNA	none	prov	9606 nash7_F1
tissue: L	age: 34	Sex: Fema	fibrosis	nas	score	total	RNA	none	prov	9606 nash79_N
tissue: L	age: 42	Sex: Fema	fibrosis	nas	score	total	RNA	none	prov	9606 nash80_F2
tissue: L	age: 33	Sex: Fema	fibrosis	nas	score	total	RNA	none	prov	9606 nash81_F2
tissue: L	age: 60	Sex: Fema	fibrosis	nas	score	total	RNA	none	prov	9606 nash82_F1
tissue: L	age: 39	Sex: Fema	fibrosis	nas	score	total	RNA	none	prov	9606 nash83_F2
tissue: L	age: 54	Sex: Fema	fibrosis	nas	score	total	RNA	none	prov	9606 nash_F0
tissue: L	age: 44	Sex: Fema	fibrosis	nas	score	total	RNA	none	prov	9606 nash_F0
tissue: L	age: 62	Sex: Fema	fibrosis	nas	score	total	RNA	none	prov	9606 nash86_F4
tissue: L	age: 36	Sex: Male	fibrosis	nas	score	total	RNA	none	prov	9606 nash87_F0
tissue: L	age: 52	Sex: Fema	fibrosis	nas	score	total	RNA	none	prov	9606 nash_N
tissue: L	age: 68	Sex: Fema	fibrosis	nas	score	total	RNA	none	prov	9606 nash89_F1
tissue: L	age: 58	Sex: Fema	fibrosis	nas	score	total	RNA	none	prov	9606 nash9_F1
tissue: L	age: 55	Sex: Male	fibrosis	nas	score	total	RNA	none	prov	9606 nash90_N
tissue: L	age: 30	Sex: Male	fibrosis	nas	score	total	RNA	none	prov	9606 nash91_F2
tissue: L	age: 52	Sex: Male	fibrosis	nas	score	total	RNA	none	prov	9606 nash92_F3
tissue: L	age: 20	Sex: Male	fibrosis	nas	score	total	RNA	none	prov	9606 nash93_F0
tissue: L	age: 48	Sex: Male	fibrosis	nas	score	total	RNA	none	prov	9606 nash9_F2
tissue: L	age: 48	Sex: Male	fibrosis	nas	score	total	RNA	none	prov	9606 nash96_F0
tissue: L	age: 48	Sex: Fema	fibrosis	nas	score	total	RNA	none	prov	9606 nash9_F1
tissue: L	age: 52	Sex: Male	fibrosis	nas	score	total	RNA	none	prov	9606 nash99_N

For Peer Review

!Sample_d	!Sample_d	!Sample_d	!Sample_d	!Sample_p	!Sample_c	!Sample_c	!Sample_c	!Sample_c
All sampl	Reads wer	Genome_bu	Supplemen	GPL21290	Zhu,,Zhuo	zzhuo@hsp	Harvard B	Departmen
All sampl	Reads wer	Genome_bu	Supplemen	GPL21290	Zhu,,Zhuo	zzhuo@hsp	Harvard B	Departmen
All sampl	Reads wer	Genome_bu	Supplemen	GPL21290	Zhu,,Zhuo	zzhuo@hsp	Harvard B	Departmen
All sampl	Reads wer	Genome_bu	Supplemen	GPL21290	Zhu,,Zhuo	zzhuo@hsp	Harvard B	Departmen
All sampl	Reads wer	Genome_bu	Supplemen	GPL21290	Zhu,,Zhuo	zzhuo@hsp	Harvard B	Departmen
All sampl	Reads wer	Genome_bu	Supplemen	GPL21290	Zhu,,Zhuo	zzhuo@hsp	Harvard B	Departmen
All sampl	Reads wer	Genome_bu	Supplemen	GPL21290	Zhu,,Zhuo	zzhuo@hsp	Harvard B	Departmen
All sampl	Reads wer	Genome_bu	Supplemen	GPL21290	Zhu,,Zhuo	zzhuo@hsp	Harvard B	Departmen
All sampl	Reads wer	Genome_bu	Supplemen	GPL21290	Zhu,,Zhuo	zzhuo@hsp	Harvard B	Departmen
All sampl	Reads wer	Genome_bu	Supplemen	GPL21290	Zhu,,Zhuo	zzhuo@hsp	Harvard B	Departmen
All sampl	Reads wer	Genome_bu	Supplemen	GPL21290	Zhu,,Zhuo	zzhuo@hsp	Harvard B	Departmen
All sampl	Reads wer	Genome_bu	Supplemen	GPL21290	Zhu,,Zhuo	zzhuo@hsp	Harvard B	Departmen
All sampl	Reads wer	Genome_bu	Supplemen	GPL21290	Zhu,,Zhuo	zzhuo@hsp	Harvard B	Departmen
All sampl	Reads wer	Genome_bu	Supplemen	GPL21290	Zhu,,Zhuo	zzhuo@hsp	Harvard B	Departmen
All sampl	Reads wer	Genome_bu	Supplemen	GPL21290	Zhu,,Zhuo	zzhuo@hsp	Harvard B	Departmen
All sampl	Reads wer	Genome_bu	Supplemen	GPL21290	Zhu,,Zhuo	zzhuo@hsp	Harvard B	Departmen
All sampl	Reads wer	Genome_bu	Supplemen	GPL21290	Zhu,,Zhuo	zzhuo@hsp	Harvard B	Departmen
All sampl	Reads wer	Genome_bu	Supplemen	GPL21290	Zhu,,Zhuo	zzhuo@hsp	Harvard B	Departmen
All sampl	Reads wer	Genome_bu	Supplemen	GPL21290	Zhu,,Zhuo	zzhuo@hsp	Harvard B	Departmen
All sampl	Reads wer	Genome_bu	Supplemen	GPL21290	Zhu,,Zhuo	zzhuo@hsp	Harvard B	Departmen
All sampl	Reads wer	Genome_bu	Supplemen	GPL21290	Zhu,,Zhuo	zzhuo@hsp	Harvard B	Departmen
All sampl	Reads wer	Genome_bu	Supplemen	GPL21290	Zhu,,Zhuo	zzhuo@hsp	Harvard B	Departmen
All sampl	Reads wer	Genome_bu	Supplemen	GPL21290	Zhu,,Zhuo	zzhuo@hsp	Harvard B	Departmen
All sampl	Reads wer	Genome_bu	Supplemen	GPL21290	Zhu,,Zhuo	zzhuo@hsp	Harvard B	Departmen
All sampl	Reads wer	Genome_bu	Supplemen	GPL21290	Zhu,,Zhuo	zzhuo@hsp	Harvard B	Departmen
All sampl	Reads wer	Genome_bu	Supplemen	GPL21290	Zhu,,Zhuo	zzhuo@hsp	Harvard B	Departmen
All sampl	Reads wer	Genome_bu	Supplemen	GPL21290	Zhu,,Zhuo	zzhuo@hsp	Harvard B	Departmen
All sampl	Reads wer	Genome_bu	Supplemen	GPL21290	Zhu,,Zhuo	zzhuo@hsp	Harvard B	Departmen
All sampl	Reads wer	Genome_bu	Supplemen	GPL21290	Zhu,,Zhuo	zzhuo@hsp	Harvard B	Departmen
All sampl	Reads wer	Genome_bu	Supplemen	GPL21290	Zhu,,Zhuo	zzhuo@hsp	Harvard B	Departmen
All sampl	Reads wer	Genome_bu	Supplemen	GPL21290	Zhu,,Zhuo	zzhuo@hsp	Harvard B	Departmen
All sampl	Reads wer	Genome_bu	Supplemen	GPL21290	Zhu,,Zhuo	zzhuo@hsp	Harvard B	Departmen
All sampl	Reads wer	Genome_bu	Supplemen	GPL21290	Zhu,,Zhuo	zzhuo@hsp	Harvard B	Departmen
All sampl	Reads wer	Genome_bu	Supplemen	GPL21290	Zhu,,Zhuo	zzhuo@hsp	Harvard B	Departmen
All sampl	Reads wer	Genome_bu	Supplemen	GPL21290	Zhu,,Zhuo	zzhuo@hsp	Harvard B	Departmen
All sampl	Reads wer	Genome_bu	Supplemen	GPL21290	Zhu,,Zhuo	zzhuo@hsp	Harvard B	Departmen
All sampl	Reads wer	Genome_bu	Supplemen	GPL21290	Zhu,,Zhuo	zzhuo@hsp	Harvard B	Departmen
All sampl	Reads wer	Genome_bu	Supplemen	GPL21290	Zhu,,Zhuo	zzhuo@hsp	Harvard B	Departmen
All sampl	Reads wer	Genome_bu	Supplemen	GPL21290	Zhu,,Zhuo	zzhuo@hsp	Harvard B	Departmen
All sampl	Reads wer	Genome_bu	Supplemen	GPL21290	Zhu,,Zhuo	zzhuo@hsp	Harvard B	Departmen
All sampl	Reads wer	Genome_bu	Supplemen	GPL21290	Zhu,,Zhuo	zzhuo@hsp	Harvard B	Departmen
All sampl	Reads wer	Genome_bu	Supplemen	GPL21290	Zhu,,Zhuo	zzhuo@hsp	Harvard B	Departmen
All sampl	Reads wer	Genome_bu	Supplemen	GPL21290	Zhu,,Zhuo	zzhuo@hsp	Harvard B	Departmen
All sampl	Reads wer	Genome_bu	Supplemen	GPL21290	Zhu,,Zhuo	zzhuo@hsp	Harvard B	Departmen
All sampl	Reads wer	Genome_bu	Supplemen	GPL21290	Zhu,,Zhuo	zzhuo@hsp	Harvard B	Departmen
All sampl	Reads wer	Genome_bu	Supplemen	GPL21290	Zhu,,Zhuo	zzhuo@hsp	Harvard B	Departmen
All sampl	Reads wer	Genome_bu	Supplemen	GPL21290	Zhu,,Zhuo	zzhuo@hsp	Harvard B	Departmen
All sampl	Reads wer	Genome_bu	Supplemen	GPL21290	Zhu,,Zhuo	zzhuo@hsp	Harvard B	Departmen
All sampl	Reads wer	Genome_bu	Supplemen	GPL21290	Zhu,,Zhuo	zzhuo@hsp	Harvard B	Departmen
All sampl	Reads wer	Genome_bu	Supplemen	GPL21290	Zhu,,Zhuo	zzhuo@hsp	Harvard B	Departmen
All sampl	Reads wer	Genome_bu	Supplemen	GPL21290	Zhu,,Zhuo	zzhuo@hsp	Harvard B	Departmen
All sampl	Reads wer	Genome_bu	Supplemen	GPL21290	Zhu,,Zhuo	zzhuo@hsp	Harvard B	Departmen
All sampl	Reads wer	Genome_bu	Supplemen	GPL21290	Zhu,,Zhuo	zzhuo@hsp	Harvard B	Departmen
All sampl	Reads wer	Genome_bu	Supplemen	GPL21290	Zhu,,Zhuo	zzhuo@hsp	Harvard B	Departmen
All sampl	Reads wer	Genome_bu	Supplemen	GPL21290	Zhu,,Zhuo	zzhuo@hsp	Harvard B	Departmen
All sampl	Reads wer	Genome_bu	Supplemen	GPL21290	Zhu,,Zhuo	zzhuo@hsp	Harvard B	Departmen

For Peer Review

All sampl	Reads wer	Genome_bu	Supplemen	GPL21290	Zhu, , Zhuo	zzhuo@hsp	Harvard B	Departmen
All sampl	Reads wer	Genome_bu	Supplemen	GPL21290	Zhu, , Zhuo	zzhuo@hsp	Harvard B	Departmen
All sampl	Reads wer	Genome_bu	Supplemen	GPL21290	Zhu, , Zhuo	zzhuo@hsp	Harvard B	Departmen
All sampl	Reads wer	Genome_bu	Supplemen	GPL21290	Zhu, , Zhuo	zzhuo@hsp	Harvard B	Departmen
All sampl	Reads wer	Genome_bu	Supplemen	GPL21290	Zhu, , Zhuo	zzhuo@hsp	Harvard B	Departmen
All sampl	Reads wer	Genome_bu	Supplemen	GPL21290	Zhu, , Zhuo	zzhuo@hsp	Harvard B	Departmen
All sampl	Reads wer	Genome_bu	Supplemen	GPL21290	Zhu, , Zhuo	zzhuo@hsp	Harvard B	Departmen
All sampl	Reads wer	Genome_bu	Supplemen	GPL21290	Zhu, , Zhuo	zzhuo@hsp	Harvard B	Departmen
All sampl	Reads wer	Genome_bu	Supplemen	GPL21290	Zhu, , Zhuo	zzhuo@hsp	Harvard B	Departmen
All sampl	Reads wer	Genome_bu	Supplemen	GPL21290	Zhu, , Zhuo	zzhuo@hsp	Harvard B	Departmen
All sampl	Reads wer	Genome_bu	Supplemen	GPL21290	Zhu, , Zhuo	zzhuo@hsp	Harvard B	Departmen
All sampl	Reads wer	Genome_bu	Supplemen	GPL21290	Zhu, , Zhuo	zzhuo@hsp	Harvard B	Departmen
All sampl	Reads wer	Genome_bu	Supplemen	GPL21290	Zhu, , Zhuo	zzhuo@hsp	Harvard B	Departmen
All sampl	Reads wer	Genome_bu	Supplemen	GPL21290	Zhu, , Zhuo	zzhuo@hsp	Harvard B	Departmen
All sampl	Reads wer	Genome_bu	Supplemen	GPL21290	Zhu, , Zhuo	zzhuo@hsp	Harvard B	Departmen
All sampl	Reads wer	Genome_bu	Supplemen	GPL21290	Zhu, , Zhuo	zzhuo@hsp	Harvard B	Departmen
All sampl	Reads wer	Genome_bu	Supplemen	GPL21290	Zhu, , Zhuo	zzhuo@hsp	Harvard B	Departmen
All sampl	Reads wer	Genome_bu	Supplemen	GPL21290	Zhu, , Zhuo	zzhuo@hsp	Harvard B	Departmen
All sampl	Reads wer	Genome_bu	Supplemen	GPL21290	Zhu, , Zhuo	zzhuo@hsp	Harvard B	Departmen
All sampl	Reads wer	Genome_bu	Supplemen	GPL21290	Zhu, , Zhuo	zzhuo@hsp	Harvard B	Departmen
All sampl	Reads wer	Genome_bu	Supplemen	GPL21290	Zhu, , Zhuo	zzhuo@hsp	Harvard B	Departmen
All sampl	Reads wer	Genome_bu	Supplemen	GPL21290	Zhu, , Zhuo	zzhuo@hsp	Harvard B	Departmen
All sampl	Reads wer	Genome_bu	Supplemen	GPL21290	Zhu, , Zhuo	zzhuo@hsp	Harvard B	Departmen
All sampl	Reads wer	Genome_bu	Supplemen	GPL21290	Zhu, , Zhuo	zzhuo@hsp	Harvard B	Departmen
All sampl	Reads wer	Genome_bu	Supplemen	GPL21290	Zhu, , Zhuo	zzhuo@hsp	Harvard B	Departmen
All sampl	Reads wer	Genome_bu	Supplemen	GPL21290	Zhu, , Zhuo	zzhuo@hsp	Harvard B	Departmen
All sampl	Reads wer	Genome_bu	Supplemen	GPL21290	Zhu, , Zhuo	zzhuo@hsp	Harvard B	Departmen
All sampl	Reads wer	Genome_bu	Supplemen	GPL21290	Zhu, , Zhuo	zzhuo@hsp	Harvard B	Departmen
All sampl	Reads wer	Genome_bu	Supplemen	GPL21290	Zhu, , Zhuo	zzhuo@hsp	Harvard B	Departmen
All sampl	Reads wer	Genome_bu	Supplemen	GPL21290	Zhu, , Zhuo	zzhuo@hsp	Harvard B	Departmen
All sampl	Reads wer	Genome_bu	Supplemen	GPL21290	Zhu, , Zhuo	zzhuo@hsp	Harvard B	Departmen
All sampl	Reads wer	Genome_bu	Supplemen	GPL21290	Zhu, , Zhuo	zzhuo@hsp	Harvard B	Departmen
All sampl	Reads wer	Genome_bu	Supplemen	GPL21290	Zhu, , Zhuo	zzhuo@hsp	Harvard B	Departmen
All sampl	Reads wer	Genome_bu	Supplemen	GPL21290	Zhu, , Zhuo	zzhuo@hsp	Harvard B	Departmen
All sampl	Reads wer	Genome_bu	Supplemen	GPL21290	Zhu, , Zhuo	zzhuo@hsp	Harvard B	Departmen
All sampl	Reads wer	Genome_bu	Supplemen	GPL21290	Zhu, , Zhuo	zzhuo@hsp	Harvard B	Departmen
All sampl	Reads wer	Genome_bu	Supplemen	GPL21290	Zhu, , Zhuo	zzhuo@hsp	Harvard B	Departmen
All sampl	Reads wer	Genome_bu	Supplemen	GPL21290	Zhu, , Zhuo	zzhuo@hsp	Harvard B	Departmen
All sampl	Reads wer	Genome_bu	Supplemen	GPL21290	Zhu, , Zhuo	zzhuo@hsp	Harvard B	Departmen
All sampl	Reads wer	Genome_bu	Supplemen	GPL21290	Zhu, , Zhuo	zzhuo@hsp	Harvard B	Departmen
All sampl	Reads wer	Genome_bu	Supplemen	GPL21290	Zhu, , Zhuo	zzhuo@hsp	Harvard B	Departmen
All sampl	Reads wer	Genome_bu	Supplemen	GPL21290	Zhu, , Zhuo	zzhuo@hsp	Harvard B	Departmen
All sampl	Reads wer	Genome_bu	Supplemen	GPL21290	Zhu, , Zhuo	zzhuo@hsp	Harvard B	Departmen
All sampl	Reads wer	Genome_bu	Supplemen	GPL21290	Zhu, , Zhuo	zzhuo@hsp	Harvard B	Departmen
All sampl	Reads wer	Genome_bu	Supplemen	GPL21290	Zhu, , Zhuo	zzhuo@hsp	Harvard B	Departmen
All sampl	Reads wer	Genome_bu	Supplemen	GPL21290	Zhu, , Zhuo	zzhuo@hsp	Harvard B	Departmen
All sampl	Reads wer	Genome_bu	Supplemen	GPL21290	Zhu, , Zhuo	zzhuo@hsp	Harvard B	Departmen
All sampl	Reads wer	Genome_bu	Supplemen	GPL21290	Zhu, , Zhuo	zzhuo@hsp	Harvard B	Departmen
All sampl	Reads wer	Genome_bu	Supplemen	GPL21290	Zhu, , Zhuo	zzhuo@hsp	Harvard B	Departmen
All sampl	Reads wer	Genome_bu	Supplemen	GPL21290	Zhu, , Zhuo	zzhuo@hsp	Harvard B	Departmen
All sampl	Reads wer	Genome_bu	Supplemen	GPL21290	Zhu, , Zhuo	zzhuo@hsp	Harvard B	Departmen
All sampl	Reads wer	Genome_bu	Supplemen	GPL21290	Zhu, , Zhuo	zzhuo@hsp	Harvard B	Departmen
All sampl	Reads wer	Genome_bu	Supplemen	GPL21290	Zhu, , Zhuo	zzhuo@hsp	Harvard B	Departmen
All sampl	Reads wer	Genome_bu	Supplemen	GPL21290	Zhu, , Zhuo	zzhuo@hsp	Harvard B	Departmen
All sampl	Reads wer	Genome_bu	Supplemen	GPL21290	Zhu, , Zhuo	zzhuo@hsp	Harvard B	Departmen
All sampl	Reads wer	Genome_bu	Supplemen	GPL21290	Zhu, , Zhuo	zzhuo@hsp	Harvard B	Departmen
All sampl	Reads wer	Genome_bu	Supplemen	GPL21290	Zhu, , Zhuo	zzhuo@hsp	Harvard B	Departmen
All sampl	Reads wer	Genome_bu	Supplemen	GPL21290	Zhu, , Zhuo	zzhuo@hsp	Harvard B	Departmen

For Peer Review

All sampl	Reads wer	Genome_bu	Supplemen	GPL21290	Zhu,,Zhuo	zzhuo@hsp	Harvard B	Departmen
All sampl	Reads wer	Genome_bu	Supplemen	GPL21290	Zhu,,Zhuo	zzhuo@hsp	Harvard B	Departmen
All sampl	Reads wer	Genome_bu	Supplemen	GPL21290	Zhu,,Zhuo	zzhuo@hsp	Harvard B	Departmen
All sampl	Reads wer	Genome_bu	Supplemen	GPL21290	Zhu,,Zhuo	zzhuo@hsp	Harvard B	Departmen
All sampl	Reads wer	Genome_bu	Supplemen	GPL21290	Zhu,,Zhuo	zzhuo@hsp	Harvard B	Departmen
All sampl	Reads wer	Genome_bu	Supplemen	GPL21290	Zhu,,Zhuo	zzhuo@hsp	Harvard B	Departmen
All sampl	Reads wer	Genome_bu	Supplemen	GPL21290	Zhu,,Zhuo	zzhuo@hsp	Harvard B	Departmen
All sampl	Reads wer	Genome_bu	Supplemen	GPL21290	Zhu,,Zhuo	zzhuo@hsp	Harvard B	Departmen
All sampl	Reads wer	Genome_bu	Supplemen	GPL21290	Zhu,,Zhuo	zzhuo@hsp	Harvard B	Departmen
All sampl	Reads wer	Genome_bu	Supplemen	GPL21290	Zhu,,Zhuo	zzhuo@hsp	Harvard B	Departmen
All sampl	Reads wer	Genome_bu	Supplemen	GPL21290	Zhu,,Zhuo	zzhuo@hsp	Harvard B	Departmen
All sampl	Reads wer	Genome_bu	Supplemen	GPL21290	Zhu,,Zhuo	zzhuo@hsp	Harvard B	Departmen
All sampl	Reads wer	Genome_bu	Supplemen	GPL21290	Zhu,,Zhuo	zzhuo@hsp	Harvard B	Departmen
All sampl	Reads wer	Genome_bu	Supplemen	GPL21290	Zhu,,Zhuo	zzhuo@hsp	Harvard B	Departmen
All sampl	Reads wer	Genome_bu	Supplemen	GPL21290	Zhu,,Zhuo	zzhuo@hsp	Harvard B	Departmen
All sampl	Reads wer	Genome_bu	Supplemen	GPL21290	Zhu,,Zhuo	zzhuo@hsp	Harvard B	Departmen
All sampl	Reads wer	Genome_bu	Supplemen	GPL21290	Zhu,,Zhuo	zzhuo@hsp	Harvard B	Departmen
All sampl	Reads wer	Genome_bu	Supplemen	GPL21290	Zhu,,Zhuo	zzhuo@hsp	Harvard B	Departmen
All sampl	Reads wer	Genome_bu	Supplemen	GPL21290	Zhu,,Zhuo	zzhuo@hsp	Harvard B	Departmen
All sampl	Reads wer	Genome_bu	Supplemen	GPL21290	Zhu,,Zhuo	zzhuo@hsp	Harvard B	Departmen
All sampl	Reads wer	Genome_bu	Supplemen	GPL21290	Zhu,,Zhuo	zzhuo@hsp	Harvard B	Departmen
All sampl	Reads wer	Genome_bu	Supplemen	GPL21290	Zhu,,Zhuo	zzhuo@hsp	Harvard B	Departmen
All sampl	Reads wer	Genome_bu	Supplemen	GPL21290	Zhu,,Zhuo	zzhuo@hsp	Harvard B	Departmen
All sampl	Reads wer	Genome_bu	Supplemen	GPL21290	Zhu,,Zhuo	zzhuo@hsp	Harvard B	Departmen
All sampl	Reads wer	Genome_bu	Supplemen	GPL21290	Zhu,,Zhuo	zzhuo@hsp	Harvard B	Departmen
All sampl	Reads wer	Genome_bu	Supplemen	GPL21290	Zhu,,Zhuo	zzhuo@hsp	Harvard B	Departmen
All sampl	Reads wer	Genome_bu	Supplemen	GPL21290	Zhu,,Zhuo	zzhuo@hsp	Harvard B	Departmen
All sampl	Reads wer	Genome_bu	Supplemen	GPL21290	Zhu,,Zhuo	zzhuo@hsp	Harvard B	Departmen
All sampl	Reads wer	Genome_bu	Supplemen	GPL21290	Zhu,,Zhuo	zzhuo@hsp	Harvard B	Departmen
All sampl	Reads wer	Genome_bu	Supplemen	GPL21290	Zhu,,Zhuo	zzhuo@hsp	Harvard B	Departmen

For Peer Review

!Sample_c	!Sample_c	!Sample_c	!Sample_c	!Sample_c	!Sample_c	!Sample_d	!Sample_i	!Sample_l
Harvard T	401 Park	Boston	MA	02130	USA	0	Illumina	cDNA
Harvard T	401 Park	Boston	MA	02130	USA	0	Illumina	cDNA
Harvard T	401 Park	Boston	MA	02130	USA	0	Illumina	cDNA
Harvard T	401 Park	Boston	MA	02130	USA	0	Illumina	cDNA
Harvard T	401 Park	Boston	MA	02130	USA	0	Illumina	cDNA
Harvard T	401 Park	Boston	MA	02130	USA	0	Illumina	cDNA
Harvard T	401 Park	Boston	MA	02130	USA	0	Illumina	cDNA
Harvard T	401 Park	Boston	MA	02130	USA	0	Illumina	cDNA
Harvard T	401 Park	Boston	MA	02130	USA	0	Illumina	cDNA
Harvard T	401 Park	Boston	MA	02130	USA	0	Illumina	cDNA
Harvard T	401 Park	Boston	MA	02130	USA	0	Illumina	cDNA
Harvard T	401 Park	Boston	MA	02130	USA	0	Illumina	cDNA
Harvard T	401 Park	Boston	MA	02130	USA	0	Illumina	cDNA
Harvard T	401 Park	Boston	MA	02130	USA	0	Illumina	cDNA
Harvard T	401 Park	Boston	MA	02130	USA	0	Illumina	cDNA
Harvard T	401 Park	Boston	MA	02130	USA	0	Illumina	cDNA
Harvard T	401 Park	Boston	MA	02130	USA	0	Illumina	cDNA
Harvard T	401 Park	Boston	MA	02130	USA	0	Illumina	cDNA
Harvard T	401 Park	Boston	MA	02130	USA	0	Illumina	cDNA
Harvard T	401 Park	Boston	MA	02130	USA	0	Illumina	cDNA
Harvard T	401 Park	Boston	MA	02130	USA	0	Illumina	cDNA
Harvard T	401 Park	Boston	MA	02130	USA	0	Illumina	cDNA
Harvard T	401 Park	Boston	MA	02130	USA	0	Illumina	cDNA
Harvard T	401 Park	Boston	MA	02130	USA	0	Illumina	cDNA
Harvard T	401 Park	Boston	MA	02130	USA	0	Illumina	cDNA
Harvard T	401 Park	Boston	MA	02130	USA	0	Illumina	cDNA
Harvard T	401 Park	Boston	MA	02130	USA	0	Illumina	cDNA
Harvard T	401 Park	Boston	MA	02130	USA	0	Illumina	cDNA
Harvard T	401 Park	Boston	MA	02130	USA	0	Illumina	cDNA
Harvard T	401 Park	Boston	MA	02130	USA	0	Illumina	cDNA
Harvard T	401 Park	Boston	MA	02130	USA	0	Illumina	cDNA
Harvard T	401 Park	Boston	MA	02130	USA	0	Illumina	cDNA
Harvard T	401 Park	Boston	MA	02130	USA	0	Illumina	cDNA
Harvard T	401 Park	Boston	MA	02130	USA	0	Illumina	cDNA
Harvard T	401 Park	Boston	MA	02130	USA	0	Illumina	cDNA
Harvard T	401 Park	Boston	MA	02130	USA	0	Illumina	cDNA
Harvard T	401 Park	Boston	MA	02130	USA	0	Illumina	cDNA
Harvard T	401 Park	Boston	MA	02130	USA	0	Illumina	cDNA
Harvard T	401 Park	Boston	MA	02130	USA	0	Illumina	cDNA
Harvard T	401 Park	Boston	MA	02130	USA	0	Illumina	cDNA
Harvard T	401 Park	Boston	MA	02130	USA	0	Illumina	cDNA
Harvard T	401 Park	Boston	MA	02130	USA	0	Illumina	cDNA
Harvard T	401 Park	Boston	MA	02130	USA	0	Illumina	cDNA
Harvard T	401 Park	Boston	MA	02130	USA	0	Illumina	cDNA
Harvard T	401 Park	Boston	MA	02130	USA	0	Illumina	cDNA
Harvard T	401 Park	Boston	MA	02130	USA	0	Illumina	cDNA
Harvard T	401 Park	Boston	MA	02130	USA	0	Illumina	cDNA
Harvard T	401 Park	Boston	MA	02130	USA	0	Illumina	cDNA
Harvard T	401 Park	Boston	MA	02130	USA	0	Illumina	cDNA
Harvard T	401 Park	Boston	MA	02130	USA	0	Illumina	cDNA
Harvard T	401 Park	Boston	MA	02130	USA	0	Illumina	cDNA
Harvard T	401 Park	Boston	MA	02130	USA	0	Illumina	cDNA
Harvard T	401 Park	Boston	MA	02130	USA	0	Illumina	cDNA
Harvard T	401 Park	Boston	MA	02130	USA	0	Illumina	cDNA
Harvard T	401 Park	Boston	MA	02130	USA	0	Illumina	cDNA
Harvard T	401 Park	Boston	MA	02130	USA	0	Illumina	cDNA

For Peer Review

Harvard T	401 Park	Boston	MA	02130	USA	0	Illumina	cDNA
Harvard T	401 Park	Boston	MA	02130	USA	0	Illumina	cDNA
Harvard T	401 Park	Boston	MA	02130	USA	0	Illumina	cDNA
Harvard T	401 Park	Boston	MA	02130	USA	0	Illumina	cDNA
Harvard T	401 Park	Boston	MA	02130	USA	0	Illumina	cDNA
Harvard T	401 Park	Boston	MA	02130	USA	0	Illumina	cDNA
Harvard T	401 Park	Boston	MA	02130	USA	0	Illumina	cDNA
Harvard T	401 Park	Boston	MA	02130	USA	0	Illumina	cDNA
Harvard T	401 Park	Boston	MA	02130	USA	0	Illumina	cDNA
Harvard T	401 Park	Boston	MA	02130	USA	0	Illumina	cDNA
Harvard T	401 Park	Boston	MA	02130	USA	0	Illumina	cDNA
Harvard T	401 Park	Boston	MA	02130	USA	0	Illumina	cDNA
Harvard T	401 Park	Boston	MA	02130	USA	0	Illumina	cDNA
Harvard T	401 Park	Boston	MA	02130	USA	0	Illumina	cDNA
Harvard T	401 Park	Boston	MA	02130	USA	0	Illumina	cDNA
Harvard T	401 Park	Boston	MA	02130	USA	0	Illumina	cDNA
Harvard T	401 Park	Boston	MA	02130	USA	0	Illumina	cDNA
Harvard T	401 Park	Boston	MA	02130	USA	0	Illumina	cDNA
Harvard T	401 Park	Boston	MA	02130	USA	0	Illumina	cDNA
Harvard T	401 Park	Boston	MA	02130	USA	0	Illumina	cDNA
Harvard T	401 Park	Boston	MA	02130	USA	0	Illumina	cDNA
Harvard T	401 Park	Boston	MA	02130	USA	0	Illumina	cDNA
Harvard T	401 Park	Boston	MA	02130	USA	0	Illumina	cDNA
Harvard T	401 Park	Boston	MA	02130	USA	0	Illumina	cDNA
Harvard T	401 Park	Boston	MA	02130	USA	0	Illumina	cDNA
Harvard T	401 Park	Boston	MA	02130	USA	0	Illumina	cDNA
Harvard T	401 Park	Boston	MA	02130	USA	0	Illumina	cDNA
Harvard T	401 Park	Boston	MA	02130	USA	0	Illumina	cDNA
Harvard T	401 Park	Boston	MA	02130	USA	0	Illumina	cDNA
Harvard T	401 Park	Boston	MA	02130	USA	0	Illumina	cDNA
Harvard T	401 Park	Boston	MA	02130	USA	0	Illumina	cDNA
Harvard T	401 Park	Boston	MA	02130	USA	0	Illumina	cDNA
Harvard T	401 Park	Boston	MA	02130	USA	0	Illumina	cDNA
Harvard T	401 Park	Boston	MA	02130	USA	0	Illumina	cDNA
Harvard T	401 Park	Boston	MA	02130	USA	0	Illumina	cDNA
Harvard T	401 Park	Boston	MA	02130	USA	0	Illumina	cDNA
Harvard T	401 Park	Boston	MA	02130	USA	0	Illumina	cDNA
Harvard T	401 Park	Boston	MA	02130	USA	0	Illumina	cDNA
Harvard T	401 Park	Boston	MA	02130	USA	0	Illumina	cDNA
Harvard T	401 Park	Boston	MA	02130	USA	0	Illumina	cDNA
Harvard T	401 Park	Boston	MA	02130	USA	0	Illumina	cDNA
Harvard T	401 Park	Boston	MA	02130	USA	0	Illumina	cDNA
Harvard T	401 Park	Boston	MA	02130	USA	0	Illumina	cDNA
Harvard T	401 Park	Boston	MA	02130	USA	0	Illumina	cDNA
Harvard T	401 Park	Boston	MA	02130	USA	0	Illumina	cDNA
Harvard T	401 Park	Boston	MA	02130	USA	0	Illumina	cDNA
Harvard T	401 Park	Boston	MA	02130	USA	0	Illumina	cDNA
Harvard T	401 Park	Boston	MA	02130	USA	0	Illumina	cDNA
Harvard T	401 Park	Boston	MA	02130	USA	0	Illumina	cDNA
Harvard T	401 Park	Boston	MA	02130	USA	0	Illumina	cDNA
Harvard T	401 Park	Boston	MA	02130	USA	0	Illumina	cDNA
Harvard T	401 Park	Boston	MA	02130	USA	0	Illumina	cDNA
Harvard T	401 Park	Boston	MA	02130	USA	0	Illumina	cDNA
Harvard T	401 Park	Boston	MA	02130	USA	0	Illumina	cDNA
Harvard T	401 Park	Boston	MA	02130	USA	0	Illumina	cDNA
Harvard T	401 Park	Boston	MA	02130	USA	0	Illumina	cDNA
Harvard T	401 Park	Boston	MA	02130	USA	0	Illumina	cDNA
Harvard T	401 Park	Boston	MA	02130	USA			

For Peer Review

Harvard	T	401 Park	Boston	MA	02130	USA	0	Illumina	cDNA
Harvard	T	401 Park	Boston	MA	02130	USA	0	Illumina	cDNA
Harvard	T	401 Park	Boston	MA	02130	USA	0	Illumina	cDNA
Harvard	T	401 Park	Boston	MA	02130	USA	0	Illumina	cDNA
Harvard	T	401 Park	Boston	MA	02130	USA	0	Illumina	cDNA
Harvard	T	401 Park	Boston	MA	02130	USA	0	Illumina	cDNA
Harvard	T	401 Park	Boston	MA	02130	USA	0	Illumina	cDNA
Harvard	T	401 Park	Boston	MA	02130	USA	0	Illumina	cDNA
Harvard	T	401 Park	Boston	MA	02130	USA	0	Illumina	cDNA
Harvard	T	401 Park	Boston	MA	02130	USA	0	Illumina	cDNA
Harvard	T	401 Park	Boston	MA	02130	USA	0	Illumina	cDNA
Harvard	T	401 Park	Boston	MA	02130	USA	0	Illumina	cDNA
Harvard	T	401 Park	Boston	MA	02130	USA	0	Illumina	cDNA
Harvard	T	401 Park	Boston	MA	02130	USA	0	Illumina	cDNA
Harvard	T	401 Park	Boston	MA	02130	USA	0	Illumina	cDNA
Harvard	T	401 Park	Boston	MA	02130	USA	0	Illumina	cDNA
Harvard	T	401 Park	Boston	MA	02130	USA	0	Illumina	cDNA
Harvard	T	401 Park	Boston	MA	02130	USA	0	Illumina	cDNA
Harvard	T	401 Park	Boston	MA	02130	USA	0	Illumina	cDNA
Harvard	T	401 Park	Boston	MA	02130	USA	0	Illumina	cDNA
Harvard	T	401 Park	Boston	MA	02130	USA	0	Illumina	cDNA
Harvard	T	401 Park	Boston	MA	02130	USA	0	Illumina	cDNA
Harvard	T	401 Park	Boston	MA	02130	USA	0	Illumina	cDNA
Harvard	T	401 Park	Boston	MA	02130	USA	0	Illumina	cDNA
Harvard	T	401 Park	Boston	MA	02130	USA	0	Illumina	cDNA
Harvard	T	401 Park	Boston	MA	02130	USA	0	Illumina	cDNA
Harvard	T	401 Park	Boston	MA	02130	USA	0	Illumina	cDNA
Harvard	T	401 Park	Boston	MA	02130	USA	0	Illumina	cDNA
Harvard	T	401 Park	Boston	MA	02130	USA	0	Illumina	cDNA
Harvard	T	401 Park	Boston	MA	02130	USA	0	Illumina	cDNA

For Peer Review

!Sample_l	!Sample_l	!Sample_r	!Sample_r	!Sample_supplementary_file_1
transcrip	RNA-Seq	BioSample	SRA:	http NONE
transcrip	RNA-Seq	BioSample	SRA:	http NONE
transcrip	RNA-Seq	BioSample	SRA:	http NONE
transcrip	RNA-Seq	BioSample	SRA:	http NONE
transcrip	RNA-Seq	BioSample	SRA:	http NONE
transcrip	RNA-Seq	BioSample	SRA:	http NONE
transcrip	RNA-Seq	BioSample	SRA:	http NONE
transcrip	RNA-Seq	BioSample	SRA:	http NONE
transcrip	RNA-Seq	BioSample	SRA:	http NONE
transcrip	RNA-Seq	BioSample	SRA:	http NONE
transcrip	RNA-Seq	BioSample	SRA:	http NONE
transcrip	RNA-Seq	BioSample	SRA:	http NONE
transcrip	RNA-Seq	BioSample	SRA:	http NONE
transcrip	RNA-Seq	BioSample	SRA:	http NONE
transcrip	RNA-Seq	BioSample	SRA:	http NONE
transcrip	RNA-Seq	BioSample	SRA:	http NONE
transcrip	RNA-Seq	BioSample	SRA:	http NONE
transcrip	RNA-Seq	BioSample	SRA:	http NONE
transcrip	RNA-Seq	BioSample	SRA:	http NONE
transcrip	RNA-Seq	BioSample	SRA:	http NONE
transcrip	RNA-Seq	BioSample	SRA:	http NONE
transcrip	RNA-Seq	BioSample	SRA:	http NONE
transcrip	RNA-Seq	BioSample	SRA:	http NONE
transcrip	RNA-Seq	BioSample	SRA:	http NONE
transcrip	RNA-Seq	BioSample	SRA:	http NONE
transcrip	RNA-Seq	BioSample	SRA:	http NONE
transcrip	RNA-Seq	BioSample	SRA:	http NONE
transcrip	RNA-Seq	BioSample	SRA:	http NONE
transcrip	RNA-Seq	BioSample	SRA:	http NONE
transcrip	RNA-Seq	BioSample	SRA:	http NONE
transcrip	RNA-Seq	BioSample	SRA:	http NONE
transcrip	RNA-Seq	BioSample	SRA:	http NONE
transcrip	RNA-Seq	BioSample	SRA:	http NONE
transcrip	RNA-Seq	BioSample	SRA:	http NONE
transcrip	RNA-Seq	BioSample	SRA:	http NONE
transcrip	RNA-Seq	BioSample	SRA:	http NONE
transcrip	RNA-Seq	BioSample	SRA:	http NONE
transcrip	RNA-Seq	BioSample	SRA:	http NONE
transcrip	RNA-Seq	BioSample	SRA:	http NONE
transcrip	RNA-Seq	BioSample	SRA:	http NONE
transcrip	RNA-Seq	BioSample	SRA:	http NONE
transcrip	RNA-Seq	BioSample	SRA:	http NONE
transcrip	RNA-Seq	BioSample	SRA:	http NONE
transcrip	RNA-Seq	BioSample	SRA:	http NONE
transcrip	RNA-Seq	BioSample	SRA:	http NONE
transcrip	RNA-Seq	BioSample	SRA:	http NONE
transcrip	RNA-Seq	BioSample	SRA:	http NONE
transcrip	RNA-Seq	BioSample	SRA:	http NONE
transcrip	RNA-Seq	BioSample	SRA:	http NONE
transcrip	RNA-Seq	BioSample	SRA:	http NONE
transcrip	RNA-Seq	BioSample	SRA:	http NONE
transcrip	RNA-Seq	BioSample	SRA:	http NONE
transcrip	RNA-Seq	BioSample	SRA:	http NONE
transcrip	RNA-Seq	BioSample	SRA:	http NONE
transcrip	RNA-Seq	BioSample	SRA:	http NONE
transcrip	RNA-Seq	BioSample	SRA:	http NONE

For Peer Review

transcrip	RNA-Seq	BioSample	SRA:	http	NONE
transcrip	RNA-Seq	BioSample	SRA:	http	NONE
transcrip	RNA-Seq	BioSample	SRA:	http	NONE
transcrip	RNA-Seq	BioSample	SRA:	http	NONE
transcrip	RNA-Seq	BioSample	SRA:	http	NONE
transcrip	RNA-Seq	BioSample	SRA:	http	NONE
transcrip	RNA-Seq	BioSample	SRA:	http	NONE
transcrip	RNA-Seq	BioSample	SRA:	http	NONE
transcrip	RNA-Seq	BioSample	SRA:	http	NONE
transcrip	RNA-Seq	BioSample	SRA:	http	NONE
transcrip	RNA-Seq	BioSample	SRA:	http	NONE
transcrip	RNA-Seq	BioSample	SRA:	http	NONE
transcrip	RNA-Seq	BioSample	SRA:	http	NONE
transcrip	RNA-Seq	BioSample	SRA:	http	NONE
transcrip	RNA-Seq	BioSample	SRA:	http	NONE
transcrip	RNA-Seq	BioSample	SRA:	http	NONE
transcrip	RNA-Seq	BioSample	SRA:	http	NONE
transcrip	RNA-Seq	BioSample	SRA:	http	NONE
transcrip	RNA-Seq	BioSample	SRA:	http	NONE
transcrip	RNA-Seq	BioSample	SRA:	http	NONE
transcrip	RNA-Seq	BioSample	SRA:	http	NONE
transcrip	RNA-Seq	BioSample	SRA:	http	NONE
transcrip	RNA-Seq	BioSample	SRA:	http	NONE
transcrip	RNA-Seq	BioSample	SRA:	http	NONE
transcrip	RNA-Seq	BioSample	SRA:	http	NONE
transcrip	RNA-Seq	BioSample	SRA:	http	NONE
transcrip	RNA-Seq	BioSample	SRA:	http	NONE
transcrip	RNA-Seq	BioSample	SRA:	http	NONE
transcrip	RNA-Seq	BioSample	SRA:	http	NONE
transcrip	RNA-Seq	BioSample	SRA:	http	NONE
transcrip	RNA-Seq	BioSample	SRA:	http	NONE
transcrip	RNA-Seq	BioSample	SRA:	http	NONE
transcrip	RNA-Seq	BioSample	SRA:	http	NONE
transcrip	RNA-Seq	BioSample	SRA:	http	NONE
transcrip	RNA-Seq	BioSample	SRA:	http	NONE
transcrip	RNA-Seq	BioSample	SRA:	http	NONE
transcrip	RNA-Seq	BioSample	SRA:	http	NONE
transcrip	RNA-Seq	BioSample	SRA:	http	NONE
transcrip	RNA-Seq	BioSample	SRA:	http	NONE
transcrip	RNA-Seq	BioSample	SRA:	http	NONE
transcrip	RNA-Seq	BioSample	SRA:	http	NONE
transcrip	RNA-Seq	BioSample	SRA:	http	NONE
transcrip	RNA-Seq	BioSample	SRA:	http	NONE
transcrip	RNA-Seq	BioSample	SRA:	http	NONE
transcrip	RNA-Seq	BioSample	SRA:	http	NONE
transcrip	RNA-Seq	BioSample	SRA:	http	NONE
transcrip	RNA-Seq	BioSample	SRA:	http	NONE
transcrip	RNA-Seq	BioSample	SRA:	http	NONE
transcrip	RNA-Seq	BioSample	SRA:	http	NONE
transcrip	RNA-Seq	BioSample	SRA:	http	NONE
transcrip	RNA-Seq	BioSample	SRA:	http	NONE
transcrip	RNA-Seq	BioSample	SRA:	http	NONE
transcrip	RNA-Seq	BioSample	SRA:	http	NONE
transcrip	RNA-Seq	BioSample	SRA:	http	NONE
transcrip	RNA-Seq	BioSample	SRA:	http	NONE
transcrip	RNA-Seq	BioSample	SRA:	http	NONE
transcrip	RNA-Seq	BioSample	SRA:	http	NONE
transcrip	RNA-Seq	BioSample	SRA:	http	NONE

For Peer Review

transcrip	RNA-Seq	BioSample	SRA:	http	NONE
transcrip	RNA-Seq	BioSample	SRA:	http	NONE
transcrip	RNA-Seq	BioSample	SRA:	http	NONE
transcrip	RNA-Seq	BioSample	SRA:	http	NONE
transcrip	RNA-Seq	BioSample	SRA:	http	NONE
transcrip	RNA-Seq	BioSample	SRA:	http	NONE
transcrip	RNA-Seq	BioSample	SRA:	http	NONE
transcrip	RNA-Seq	BioSample	SRA:	http	NONE
transcrip	RNA-Seq	BioSample	SRA:	http	NONE
transcrip	RNA-Seq	BioSample	SRA:	http	NONE
transcrip	RNA-Seq	BioSample	SRA:	http	NONE
transcrip	RNA-Seq	BioSample	SRA:	http	NONE
transcrip	RNA-Seq	BioSample	SRA:	http	NONE
transcrip	RNA-Seq	BioSample	SRA:	http	NONE
transcrip	RNA-Seq	BioSample	SRA:	http	NONE
transcrip	RNA-Seq	BioSample	SRA:	http	NONE
transcrip	RNA-Seq	BioSample	SRA:	http	NONE
transcrip	RNA-Seq	BioSample	SRA:	http	NONE
transcrip	RNA-Seq	BioSample	SRA:	http	NONE
transcrip	RNA-Seq	BioSample	SRA:	http	NONE
transcrip	RNA-Seq	BioSample	SRA:	http	NONE
transcrip	RNA-Seq	BioSample	SRA:	http	NONE
transcrip	RNA-Seq	BioSample	SRA:	http	NONE
transcrip	RNA-Seq	BioSample	SRA:	http	NONE
transcrip	RNA-Seq	BioSample	SRA:	http	NONE
transcrip	RNA-Seq	BioSample	SRA:	http	NONE
transcrip	RNA-Seq	BioSample	SRA:	http	NONE
transcrip	RNA-Seq	BioSample	SRA:	http	NONE
transcrip	RNA-Seq	BioSample	SRA:	http	NONE
transcrip	RNA-Seq	BioSample	SRA:	http	NONE

Table S1d. Sample Information of GSE84044

!Sample_t	!Sample_g	!Sample_s	!Sample_s	!Sample_l	!Sample_t	!Sample_c	!Sample_s	!Sample_o
Patient 0	GSM222628	Public on	Jul 05 20	Aug 21 20	RNA	1	Hepatic t	Homo sapi
Patient 0	GSM222628	Public on	Jul 05 20	Aug 21 20	RNA	1	Hepatic t	Homo sapi
Patient 0	GSM222628	Public on	Jul 05 20	Aug 21 20	RNA	1	Hepatic t	Homo sapi
Patient 0	GSM222628	Public on	Jul 05 20	Aug 21 20	RNA	1	Hepatic t	Homo sapi
Patient 0	GSM222628	Public on	Jul 05 20	Aug 21 20	RNA	1	Hepatic t	Homo sapi
Patient 0	GSM222628	Public on	Jul 05 20	Aug 21 20	RNA	1	Hepatic t	Homo sapi
Patient 0	GSM222628	Public on	Jul 05 20	Aug 21 20	RNA	1	Hepatic t	Homo sapi
Patient 0	GSM222628	Public on	Jul 05 20	Aug 21 20	RNA	1	Hepatic t	Homo sapi
Patient 0	GSM222628	Public on	Jul 05 20	Aug 21 20	RNA	1	Hepatic t	Homo sapi
Patient 0	GSM222628	Public on	Jul 05 20	Aug 21 20	RNA	1	Hepatic t	Homo sapi
Patient 0	GSM222629	Public on	Jul 05 20	Aug 21 20	RNA	1	Hepatic t	Homo sapi
Patient 0	GSM222629	Public on	Jul 05 20	Aug 21 20	RNA	1	Hepatic t	Homo sapi
Patient 0	GSM222629	Public on	Jul 05 20	Aug 21 20	RNA	1	Hepatic t	Homo sapi
Patient 0	GSM222629	Public on	Jul 05 20	Aug 21 20	RNA	1	Hepatic t	Homo sapi
Patient 0	GSM222629	Public on	Jul 05 20	Aug 21 20	RNA	1	Hepatic t	Homo sapi
Patient 0	GSM222629	Public on	Jul 05 20	Aug 21 20	RNA	1	Hepatic t	Homo sapi
Patient 0	GSM222629	Public on	Jul 05 20	Aug 21 20	RNA	1	Hepatic t	Homo sapi
Patient 0	GSM222629	Public on	Jul 05 20	Aug 21 20	RNA	1	Hepatic t	Homo sapi
Patient 0	GSM222629	Public on	Jul 05 20	Aug 21 20	RNA	1	Hepatic t	Homo sapi
Patient 0	GSM222629	Public on	Jul 05 20	Aug 21 20	RNA	1	Hepatic t	Homo sapi
Patient 0	GSM222630	Public on	Jul 05 20	Aug 21 20	RNA	1	Hepatic t	Homo sapi
Patient 0	GSM222630	Public on	Jul 05 20	Aug 21 20	RNA	1	Hepatic t	Homo sapi
Patient 0	GSM222630	Public on	Jul 05 20	Aug 21 20	RNA	1	Hepatic t	Homo sapi
Patient 0	GSM222630	Public on	Jul 05 20	Aug 21 20	RNA	1	Hepatic t	Homo sapi
Patient 0	GSM222630	Public on	Jul 05 20	Aug 21 20	RNA	1	Hepatic t	Homo sapi
Patient 0	GSM222630	Public on	Jul 05 20	Aug 21 20	RNA	1	Hepatic t	Homo sapi
Patient 0	GSM222630	Public on	Jul 05 20	Aug 21 20	RNA	1	Hepatic t	Homo sapi
Patient 0	GSM222630	Public on	Jul 05 20	Aug 21 20	RNA	1	Hepatic t	Homo sapi
Patient 0	GSM222630	Public on	Jul 05 20	Aug 21 20	RNA	1	Hepatic t	Homo sapi
Patient 0	GSM222630	Public on	Jul 05 20	Aug 21 20	RNA	1	Hepatic t	Homo sapi
Patient 0	GSM222631	Public on	Jul 05 20	Aug 21 20	RNA	1	Hepatic t	Homo sapi
Patient 0	GSM222631	Public on	Jul 05 20	Aug 21 20	RNA	1	Hepatic t	Homo sapi
Patient 0	GSM222631	Public on	Jul 05 20	Aug 21 20	RNA	1	Hepatic t	Homo sapi
Patient 0	GSM222631	Public on	Jul 05 20	Aug 21 20	RNA	1	Hepatic t	Homo sapi
Patient 0	GSM222631	Public on	Jul 05 20	Aug 21 20	RNA	1	Hepatic t	Homo sapi
Patient 0	GSM222631	Public on	Jul 05 20	Aug 21 20	RNA	1	Hepatic t	Homo sapi
Patient 0	GSM222631	Public on	Jul 05 20	Aug 21 20	RNA	1	Hepatic t	Homo sapi
Patient 0	GSM222631	Public on	Jul 05 20	Aug 21 20	RNA	1	Hepatic t	Homo sapi
Patient 0	GSM222631	Public on	Jul 05 20	Aug 21 20	RNA	1	Hepatic t	Homo sapi
Patient 0	GSM222631	Public on	Jul 05 20	Aug 21 20	RNA	1	Hepatic t	Homo sapi
Patient 0	GSM222632	Public on	Jul 05 20	Aug 21 20	RNA	1	Hepatic t	Homo sapi
Patient 0	GSM222632	Public on	Jul 05 20	Aug 21 20	RNA	1	Hepatic t	Homo sapi
Patient 0	GSM222632	Public on	Jul 05 20	Aug 21 20	RNA	1	Hepatic t	Homo sapi
Patient 0	GSM222632	Public on	Jul 05 20	Aug 21 20	RNA	1	Hepatic t	Homo sapi
Patient 0	GSM222632	Public on	Jul 05 20	Aug 21 20	RNA	1	Hepatic t	Homo sapi
Patient 0	GSM222632	Public on	Jul 05 20	Aug 21 20	RNA	1	Hepatic t	Homo sapi
Patient 0	GSM222632	Public on	Jul 05 20	Aug 21 20	RNA	1	Hepatic t	Homo sapi
Patient 0	GSM222632	Public on	Jul 05 20	Aug 21 20	RNA	1	Hepatic t	Homo sapi
Patient 0	GSM222632	Public on	Jul 05 20	Aug 21 20	RNA	1	Hepatic t	Homo sapi
Patient 0	GSM222632	Public on	Jul 05 20	Aug 21 20	RNA	1	Hepatic t	Homo sapi
Patient 0	GSM222633	Public on	Jul 05 20	Aug 21 20	RNA	1	Hepatic t	Homo sapi
Patient 0	GSM222633	Public on	Jul 05 20	Aug 21 20	RNA	1	Hepatic t	Homo sapi
Patient 0	GSM222633	Public on	Jul 05 20	Aug 21 20	RNA	1	Hepatic t	Homo sapi
Patient 0	GSM222633	Public on	Jul 05 20	Aug 21 20	RNA	1	Hepatic t	Homo sapi
Patient 0	GSM222633	Public on	Jul 05 20	Aug 21 20	RNA	1	Hepatic t	Homo sapi
Patient 0	GSM222633	Public on	Jul 05 20	Aug 21 20	RNA	1	Hepatic t	Homo sapi

For Peer Review

Patient 0	GSM222633	Public on Jul 05 20	Aug 21 20	RNA		1	Hepatic t	Homo sapi
Patient 0	GSM222633	Public on Jul 05 20	Aug 21 20	RNA		1	Hepatic t	Homo sapi
Patient 0	GSM222633	Public on Jul 05 20	Aug 21 20	RNA		1	Hepatic t	Homo sapi
Patient 0	GSM222633	Public on Jul 05 20	Aug 21 20	RNA		1	Hepatic t	Homo sapi
Patient 0	GSM222634	Public on Jul 05 20	Aug 21 20	RNA		1	Hepatic t	Homo sapi
Patient 0	GSM222634	Public on Jul 05 20	Aug 21 20	RNA		1	Hepatic t	Homo sapi
Patient 0	GSM222634	Public on Jul 05 20	Aug 21 20	RNA		1	Hepatic t	Homo sapi
Patient 0	GSM222634	Public on Jul 05 20	Aug 21 20	RNA		1	Hepatic t	Homo sapi
Patient 0	GSM222634	Public on Jul 05 20	Aug 21 20	RNA		1	Hepatic t	Homo sapi
Patient 0	GSM222634	Public on Jul 05 20	Aug 21 20	RNA		1	Hepatic t	Homo sapi
Patient 0	GSM222634	Public on Jul 05 20	Aug 21 20	RNA		1	Hepatic t	Homo sapi
Patient 0	GSM222634	Public on Jul 05 20	Aug 21 20	RNA		1	Hepatic t	Homo sapi
Patient 0	GSM222634	Public on Jul 05 20	Aug 21 20	RNA		1	Hepatic t	Homo sapi
Patient 0	GSM222634	Public on Jul 05 20	Aug 21 20	RNA		1	Hepatic t	Homo sapi
Patient 0	GSM222635	Public on Jul 05 20	Aug 21 20	RNA		1	Hepatic t	Homo sapi
Patient 0	GSM222635	Public on Jul 05 20	Aug 21 20	RNA		1	Hepatic t	Homo sapi
Patient 0	GSM222635	Public on Jul 05 20	Aug 21 20	RNA		1	Hepatic t	Homo sapi
Patient 0	GSM222635	Public on Jul 05 20	Aug 21 20	RNA		1	Hepatic t	Homo sapi
Patient 0	GSM222635	Public on Jul 05 20	Aug 21 20	RNA		1	Hepatic t	Homo sapi
Patient 0	GSM222635	Public on Jul 05 20	Aug 21 20	RNA		1	Hepatic t	Homo sapi
Patient 0	GSM222635	Public on Jul 05 20	Aug 21 20	RNA		1	Hepatic t	Homo sapi
Patient 0	GSM222635	Public on Jul 05 20	Aug 21 20	RNA		1	Hepatic t	Homo sapi
Patient 0	GSM222635	Public on Jul 05 20	Aug 21 20	RNA		1	Hepatic t	Homo sapi
Patient 0	GSM222635	Public on Jul 05 20	Aug 21 20	RNA		1	Hepatic t	Homo sapi
Patient 0	GSM222636	Public on Jul 05 20	Aug 21 20	RNA		1	Hepatic t	Homo sapi
Patient 0	GSM222636	Public on Jul 05 20	Aug 21 20	RNA		1	Hepatic t	Homo sapi
Patient 0	GSM222636	Public on Jul 05 20	Aug 21 20	RNA		1	Hepatic t	Homo sapi
Patient 0	GSM222636	Public on Jul 05 20	Aug 21 20	RNA		1	Hepatic t	Homo sapi
Patient 0	GSM222636	Public on Jul 05 20	Aug 21 20	RNA		1	Hepatic t	Homo sapi
Patient 0	GSM222636	Public on Jul 05 20	Aug 21 20	RNA		1	Hepatic t	Homo sapi
Patient 0	GSM222636	Public on Jul 05 20	Aug 21 20	RNA		1	Hepatic t	Homo sapi
Patient 0	GSM222636	Public on Jul 05 20	Aug 21 20	RNA		1	Hepatic t	Homo sapi
Patient 0	GSM222636	Public on Jul 05 20	Aug 21 20	RNA		1	Hepatic t	Homo sapi
Patient 0	GSM222636	Public on Jul 05 20	Aug 21 20	RNA		1	Hepatic t	Homo sapi
Patient 0	GSM222637	Public on Jul 05 20	Aug 21 20	RNA		1	Hepatic t	Homo sapi
Patient 0	GSM222637	Public on Jul 05 20	Aug 21 20	RNA		1	Hepatic t	Homo sapi
Patient 0	GSM222637	Public on Jul 05 20	Aug 21 20	RNA		1	Hepatic t	Homo sapi
Patient 0	GSM222637	Public on Jul 05 20	Aug 21 20	RNA		1	Hepatic t	Homo sapi
Patient 0	GSM222637	Public on Jul 05 20	Aug 21 20	RNA		1	Hepatic t	Homo sapi
Patient 0	GSM222637	Public on Jul 05 20	Aug 21 20	RNA		1	Hepatic t	Homo sapi
Patient 0	GSM222637	Public on Jul 05 20	Aug 21 20	RNA		1	Hepatic t	Homo sapi
Patient 0	GSM222637	Public on Jul 05 20	Aug 21 20	RNA		1	Hepatic t	Homo sapi
Patient 0	GSM222637	Public on Jul 05 20	Aug 21 20	RNA		1	Hepatic t	Homo sapi
Patient 0	GSM222637	Public on Jul 05 20	Aug 21 20	RNA		1	Hepatic t	Homo sapi
Patient 0	GSM222638	Public on Jul 05 20	Aug 21 20	RNA		1	Hepatic t	Homo sapi
Patient 0	GSM222638	Public on Jul 05 20	Aug 21 20	RNA		1	Hepatic t	Homo sapi
Patient 0	GSM222638	Public on Jul 05 20	Aug 21 20	RNA		1	Hepatic t	Homo sapi
Patient 0	GSM222638	Public on Jul 05 20	Aug 21 20	RNA		1	Hepatic t	Homo sapi
Patient 0	GSM222638	Public on Jul 05 20	Aug 21 20	RNA		1	Hepatic t	Homo sapi
Patient 0	GSM222638	Public on Jul 05 20	Aug 21 20	RNA		1	Hepatic t	Homo sapi
Patient 0	GSM222638	Public on Jul 05 20	Aug 21 20	RNA		1	Hepatic t	Homo sapi
Patient 0	GSM222638	Public on Jul 05 20	Aug 21 20	RNA		1	Hepatic t	Homo sapi
Patient 0	GSM222638	Public on Jul 05 20	Aug 21 20	RNA		1	Hepatic t	Homo sapi
Patient 0	GSM222638	Public on Jul 05 20	Aug 21 20	RNA		1	Hepatic t	Homo sapi
Patient 0	GSM222639	Public on Jul 05 20	Aug 21 20	RNA		1	Hepatic t	Homo sapi
Patient 0	GSM222639	Public on Jul 05 20	Aug 21 20	RNA		1	Hepatic t	Homo sapi
Patient 0	GSM222639	Public on Jul 05 20	Aug 21 20	RNA		1	Hepatic t	Homo sapi
Patient 0	GSM222639	Public on Jul 05 20	Aug 21 20	RNA		1	Hepatic t	Homo sapi

For Peer Review

Patient 0	GSM222639	Public on	Jul 05 20	Aug 21 20	RNA	1	Hepatic t	Homo sapi
Patient 0	GSM222639	Public on	Jul 05 20	Aug 21 20	RNA	1	Hepatic t	Homo sapi
Patient 0	GSM222639	Public on	Jul 05 20	Aug 21 20	RNA	1	Hepatic t	Homo sapi
Patient 0	GSM222639	Public on	Jul 05 20	Aug 21 20	RNA	1	Hepatic t	Homo sapi
Patient 0	GSM222639	Public on	Jul 05 20	Aug 21 20	RNA	1	Hepatic t	Homo sapi
Patient 0	GSM222639	Public on	Jul 05 20	Aug 21 20	RNA	1	Hepatic t	Homo sapi
Patient 0	GSM222640	Public on	Jul 05 20	Aug 21 20	RNA	1	Hepatic t	Homo sapi
Patient 0	GSM222640	Public on	Jul 05 20	Aug 21 20	RNA	1	Hepatic t	Homo sapi
Patient 0	GSM222640	Public on	Jul 05 20	Aug 21 20	RNA	1	Hepatic t	Homo sapi
Patient 0	GSM222640	Public on	Jul 05 20	Aug 21 20	RNA	1	Hepatic t	Homo sapi

For Peer Review

!Sample_c	!Sample_c	!Sample_c	!Sample_c	!Sample_c	!Sample_c	!Sample_m	!Sample_e	!Sample_l
tissue: H	gender: M	age: 42	batch: 1	scheuer s	scheuer s	total RNA	Total RNA	Cy3
tissue: H	gender: M	age: 56	batch: 1	scheuer s	scheuer s	total RNA	Total RNA	Cy3
tissue: H	gender: F	age: 54	batch: 1	scheuer s	scheuer s	total RNA	Total RNA	Cy3
tissue: H	gender: F	age: 41	batch: 3	scheuer s	scheuer s	total RNA	Total RNA	Cy3
tissue: H	gender: F	age: 37	batch: 1	scheuer s	scheuer s	total RNA	Total RNA	Cy3
tissue: H	gender: F	age: 33	batch: 1	scheuer s	scheuer s	total RNA	Total RNA	Cy3
tissue: H	gender: M	age: 52	batch: 1	scheuer s	scheuer s	total RNA	Total RNA	Cy3
tissue: H	gender: M	age: 35	batch: 1	scheuer s	scheuer s	total RNA	Total RNA	Cy3
tissue: H	gender: M	age: 43	batch: 1	scheuer s	scheuer s	total RNA	Total RNA	Cy3
tissue: H	gender: M	age: 44	batch: 1	scheuer s	scheuer s	total RNA	Total RNA	Cy3
tissue: H	gender: F	age: 35	batch: 1	scheuer s	scheuer s	total RNA	Total RNA	Cy3
tissue: H	gender: M	age: 58	batch: 1	scheuer s	scheuer s	total RNA	Total RNA	Cy3
tissue: H	gender: M	age: 43	batch: 1	scheuer s	scheuer s	total RNA	Total RNA	Cy3
tissue: H	gender: M	age: 34	batch: 1	scheuer s	scheuer s	total RNA	Total RNA	Cy3
tissue: H	gender: M	age: 34	batch: 1	scheuer s	scheuer s	total RNA	Total RNA	Cy3
tissue: H	gender: M	age: 58	batch: 4	scheuer s	scheuer s	total RNA	Total RNA	Cy3
tissue: H	gender: M	age: 48	batch: 3	scheuer s	scheuer s	total RNA	Total RNA	Cy3
tissue: H	gender: F	age: 48	batch: 3	scheuer s	scheuer s	total RNA	Total RNA	Cy3
tissue: H	gender: F	age: 33	batch: 3	scheuer s	scheuer s	total RNA	Total RNA	Cy3
tissue: H	gender: M	age: 37	batch: 3	scheuer s	scheuer s	total RNA	Total RNA	Cy3
tissue: H	gender: M	age: 59	batch: 4	scheuer s	scheuer s	total RNA	Total RNA	Cy3
tissue: H	gender: F	age: 37	batch: 3	scheuer s	scheuer s	total RNA	Total RNA	Cy3
tissue: H	gender: F	age: 64	batch: 3	scheuer s	scheuer s	total RNA	Total RNA	Cy3
tissue: H	gender: F	age: 37	batch: 3	scheuer s	scheuer s	total RNA	Total RNA	Cy3
tissue: H	gender: M	age: 45	batch: 4	scheuer s	scheuer s	total RNA	Total RNA	Cy3
tissue: H	gender: F	age: 40	batch: 4	scheuer s	scheuer s	total RNA	Total RNA	Cy3
tissue: H	gender: F	age: 40	batch: 4	scheuer s	scheuer s	total RNA	Total RNA	Cy3
tissue: H	gender: M	age: 60	batch: 1	scheuer s	scheuer s	total RNA	Total RNA	Cy3
tissue: H	gender: F	age: 61	batch: 1	scheuer s	scheuer s	total RNA	Total RNA	Cy3
tissue: H	gender: M	age: 29	batch: 1	scheuer s	scheuer s	total RNA	Total RNA	Cy3
tissue: H	gender: M	age: 44	batch: 1	scheuer s	scheuer s	total RNA	Total RNA	Cy3
tissue: H	gender: M	age: 46	batch: 1	scheuer s	scheuer s	total RNA	Total RNA	Cy3
tissue: H	gender: M	age: 32	batch: 1	scheuer s	scheuer s	total RNA	Total RNA	Cy3
tissue: H	gender: M	age: 33	batch: 1	scheuer s	scheuer s	total RNA	Total RNA	Cy3
tissue: H	gender: M	age: 28	batch: 3	scheuer s	scheuer s	total RNA	Total RNA	Cy3
tissue: H	gender: M	age: 46	batch: 3	scheuer s	scheuer s	total RNA	Total RNA	Cy3
tissue: H	gender: M	age: 51	batch: 1	scheuer s	scheuer s	total RNA	Total RNA	Cy3
tissue: H	gender: M	age: 47	batch: 1	scheuer s	scheuer s	total RNA	Total RNA	Cy3
tissue: H	gender: M	age: 52	batch: 4	scheuer s	scheuer s	total RNA	Total RNA	Cy3
tissue: H	gender: M	age: 44	batch: 1	scheuer s	scheuer s	total RNA	Total RNA	Cy3
tissue: H	gender: F	age: 55	batch: 4	scheuer s	scheuer s	total RNA	Total RNA	Cy3
tissue: H	gender: M	age: 38	batch: 1	scheuer s	scheuer s	total RNA	Total RNA	Cy3
tissue: H	gender: F	age: 56	batch: 1	scheuer s	scheuer s	total RNA	Total RNA	Cy3
tissue: H	gender: M	age: 26	batch: 1	scheuer s	scheuer s	total RNA	Total RNA	Cy3
tissue: H	gender: M	age: 42	batch: 1	scheuer s	scheuer s	total RNA	Total RNA	Cy3
tissue: H	gender: F	age: 39	batch: 1	scheuer s	scheuer s	total RNA	Total RNA	Cy3
tissue: H	gender: F	age: 71	batch: 1	scheuer s	scheuer s	total RNA	Total RNA	Cy3
tissue: H	gender: M	age: 57	batch: 1	scheuer s	scheuer s	total RNA	Total RNA	Cy3
tissue: H	gender: M	age: 33	batch: 1	scheuer s	scheuer s	total RNA	Total RNA	Cy3
tissue: H	gender: M	age: 60	batch: 1	scheuer s	scheuer s	total RNA	Total RNA	Cy3
tissue: H	gender: M	age: 34	batch: 1	scheuer s	scheuer s	total RNA	Total RNA	Cy3
tissue: H	gender: F	age: 42	batch: 1	scheuer s	scheuer s	total RNA	Total RNA	Cy3
tissue: H	gender: M	age: 62	batch: 1	scheuer s	scheuer s	total RNA	Total RNA	Cy3
tissue: H	gender: M	age: 71	batch: 1	scheuer s	scheuer s	total RNA	Total RNA	Cy3
tissue: H	gender: F	age: 48	batch: 1	scheuer s	scheuer s	total RNA	Total RNA	Cy3
tissue: H	gender: M	age: 36	batch: 1	scheuer s	scheuer s	total RNA	Total RNA	Cy3

For Peer Review

tissue: H	gender: F	age: 32	batch: 2	scheuer s	scheuer s	total RNA	Total RNA	Cy3
tissue: H	gender: M	age: 66	batch: 2	scheuer s	scheuer s	total RNA	Total RNA	Cy3
tissue: H	gender: M	age: 31	batch: 3	scheuer s	scheuer s	total RNA	Total RNA	Cy3
tissue: H	gender: M	age: 28	batch: 3	scheuer s	scheuer s	total RNA	Total RNA	Cy3
tissue: H	gender: M	age: 30	batch: 3	scheuer s	scheuer s	total RNA	Total RNA	Cy3
tissue: H	gender: M	age: 47	batch: 4	scheuer s	scheuer s	total RNA	Total RNA	Cy3
tissue: H	gender: M	age: 36	batch: 3	scheuer s	scheuer s	total RNA	Total RNA	Cy3
tissue: H	gender: M	age: 43	batch: 3	scheuer s	scheuer s	total RNA	Total RNA	Cy3
tissue: H	gender: M	age: 35	batch: 3	scheuer s	scheuer s	total RNA	Total RNA	Cy3
tissue: H	gender: M	age: 34	batch: 3	scheuer s	scheuer s	total RNA	Total RNA	Cy3
tissue: H	gender: M	age: 43	batch: 3	scheuer s	scheuer s	total RNA	Total RNA	Cy3
tissue: H	gender: F	age: 47	batch: 3	scheuer s	scheuer s	total RNA	Total RNA	Cy3
tissue: H	gender: F	age: 37	batch: 3	scheuer s	scheuer s	total RNA	Total RNA	Cy3
tissue: H	gender: F	age: 53	batch: 3	scheuer s	scheuer s	total RNA	Total RNA	Cy3
tissue: H	gender: M	age: 58	batch: 3	scheuer s	scheuer s	total RNA	Total RNA	Cy3
tissue: H	gender: M	age: 35	batch: 1	scheuer s	scheuer s	total RNA	Total RNA	Cy3
tissue: H	gender: F	age: 33	batch: 1	scheuer s	scheuer s	total RNA	Total RNA	Cy3
tissue: H	gender: M	age: 54	batch: 1	scheuer s	scheuer s	total RNA	Total RNA	Cy3
tissue: H	gender: F	age: 37	batch: 1	scheuer s	scheuer s	total RNA	Total RNA	Cy3
tissue: H	gender: M	age: 27	batch: 1	scheuer s	scheuer s	total RNA	Total RNA	Cy3
tissue: H	gender: M	age: 38	batch: 1	scheuer s	scheuer s	total RNA	Total RNA	Cy3
tissue: H	gender: F	age: 33	batch: 1	scheuer s	scheuer s	total RNA	Total RNA	Cy3
tissue: H	gender: M	age: 58	batch: 1	scheuer s	scheuer s	total RNA	Total RNA	Cy3
tissue: H	gender: M	age: 22	batch: 4	scheuer s	scheuer s	total RNA	Total RNA	Cy3
tissue: H	gender: M	age: 28	batch: 1	scheuer s	scheuer s	total RNA	Total RNA	Cy3
tissue: H	gender: M	age: 41	batch: 1	scheuer s	scheuer s	total RNA	Total RNA	Cy3
tissue: H	gender: M	age: 42	batch: 1	scheuer s	scheuer s	total RNA	Total RNA	Cy3
tissue: H	gender: M	age: 43	batch: 1	scheuer s	scheuer s	total RNA	Total RNA	Cy3
tissue: H	gender: M	age: 24	batch: 1	scheuer s	scheuer s	total RNA	Total RNA	Cy3
tissue: H	gender: M	age: 50	batch: 4	scheuer s	scheuer s	total RNA	Total RNA	Cy3
tissue: H	gender: M	age: 23	batch: 3	scheuer s	scheuer s	total RNA	Total RNA	Cy3
tissue: H	gender: F	age: 38	batch: 3	scheuer s	scheuer s	total RNA	Total RNA	Cy3
tissue: H	gender: M	age: 31	batch: 3	scheuer s	scheuer s	total RNA	Total RNA	Cy3
tissue: H	gender: M	age: 31	batch: 3	scheuer s	scheuer s	total RNA	Total RNA	Cy3
tissue: H	gender: F	age: 28	batch: 3	scheuer s	scheuer s	total RNA	Total RNA	Cy3
tissue: H	gender: M	age: 35	batch: 3	scheuer s	scheuer s	total RNA	Total RNA	Cy3
tissue: H	gender: M	age: 31	batch: 3	scheuer s	scheuer s	total RNA	Total RNA	Cy3
tissue: H	gender: F	age: 44	batch: 3	scheuer s	scheuer s	total RNA	Total RNA	Cy3
tissue: H	gender: M	age: 52	batch: 3	scheuer s	scheuer s	total RNA	Total RNA	Cy3
tissue: H	gender: M	age: 66	batch: 3	scheuer s	scheuer s	total RNA	Total RNA	Cy3
tissue: H	gender: F	age: 36	batch: 3	scheuer s	scheuer s	total RNA	Total RNA	Cy3
tissue: H	gender: M	age: 74	batch: 3	scheuer s	scheuer s	total RNA	Total RNA	Cy3
tissue: H	gender: F	age: 37	batch: 3	scheuer s	scheuer s	total RNA	Total RNA	Cy3
tissue: H	gender: M	age: 53	batch: 3	scheuer s	scheuer s	total RNA	Total RNA	Cy3
tissue: H	gender: F	age: 33	batch: 3	scheuer s	scheuer s	total RNA	Total RNA	Cy3
tissue: H	gender: M	age: 37	batch: 3	scheuer s	scheuer s	total RNA	Total RNA	Cy3
tissue: H	gender: M	age: 29	batch: 3	scheuer s	scheuer s	total RNA	Total RNA	Cy3
tissue: H	gender: M	age: 63	batch: 3	scheuer s	scheuer s	total RNA	Total RNA	Cy3
tissue: H	gender: F	age: 69	batch: 3	scheuer s	scheuer s	total RNA	Total RNA	Cy3
tissue: H	gender: F	age: 71	batch: 3	scheuer s	scheuer s	total RNA	Total RNA	Cy3
tissue: H	gender: M	age: 32	batch: 3	scheuer s	scheuer s	total RNA	Total RNA	Cy3
tissue: H	gender: M	age: 51	batch: 3	scheuer s	scheuer s	total RNA	Total RNA	Cy3
tissue: H	gender: M	age: 34	batch: 3	scheuer s	scheuer s	total RNA	Total RNA	Cy3
tissue: H	gender: M	age: 40	batch: 3	scheuer s	scheuer s	total RNA	Total RNA	Cy3
tissue: H	gender: M	age: 30	batch: 3	scheuer s	scheuer s	total RNA	Total RNA	Cy3
tissue: H	gender: M	age: 36	batch: 3	scheuer s	scheuer s	total RNA	Total RNA	Cy3
tissue: H	gender: F	age: 47	batch: 3	scheuer s	scheuer s	total RNA	Total RNA	Cy3
tissue: H	gender: M	age: 44	batch: 3	scheuer s	scheuer s	total RNA	Total RNA	Cy3

For Peer Review

tissue: H	gender: M	age: 35	batch: 3	scheuer s	scheuer s	total RNA	Total RNA	Cy3
tissue: H	gender: M	age: 49	batch: 3	scheuer s	scheuer s	total RNA	Total RNA	Cy3
tissue: H	gender: M	age: 29	batch: 3	scheuer s	scheuer s	total RNA	Total RNA	Cy3
tissue: H	gender: M	age: 29	batch: 3	scheuer s	scheuer s	total RNA	Total RNA	Cy3
tissue: H	gender: M	age: 37	batch: 3	scheuer s	scheuer s	total RNA	Total RNA	Cy3
tissue: H	gender: M	age: 32	batch: 3	scheuer s	scheuer s	total RNA	Total RNA	Cy3
tissue: H	gender: M	age: 26	batch: 3	scheuer s	scheuer s	total RNA	Total RNA	Cy3
tissue: H	gender: M	age: 27	batch: 3	scheuer s	scheuer s	total RNA	Total RNA	Cy3
tissue: H	gender: M	age: 49	batch: 3	scheuer s	scheuer s	total RNA	Total RNA	Cy3
tissue: H	gender: F	age: 61	batch: 3	scheuer s	scheuer s	total RNA	Total RNA	Cy3

For Peer Review

!Sample_l	!Sample_t	!Sample_h	!Sample_s	!Sample_d	!Sample_d	!Sample_p	!Sample_c	!Sample_c
Cy3	9606	After fra	GeneChips	Gene expr	All raw d	GPL570	Mingjie,,	huzai9206
Cy3	9606	After fra	GeneChips	Gene expr	All raw d	GPL570	Mingjie,,	huzai9206
Cy3	9606	After fra	GeneChips	Gene expr	All raw d	GPL570	Mingjie,,	huzai9206
Cy3	9606	After fra	GeneChips	Gene expr	All raw d	GPL570	Mingjie,,	huzai9206
Cy3	9606	After fra	GeneChips	Gene expr	All raw d	GPL570	Mingjie,,	huzai9206
Cy3	9606	After fra	GeneChips	Gene expr	All raw d	GPL570	Mingjie,,	huzai9206
Cy3	9606	After fra	GeneChips	Gene expr	All raw d	GPL570	Mingjie,,	huzai9206
Cy3	9606	After fra	GeneChips	Gene expr	All raw d	GPL570	Mingjie,,	huzai9206
Cy3	9606	After fra	GeneChips	Gene expr	All raw d	GPL570	Mingjie,,	huzai9206
Cy3	9606	After fra	GeneChips	Gene expr	All raw d	GPL570	Mingjie,,	huzai9206
Cy3	9606	After fra	GeneChips	Gene expr	All raw d	GPL570	Mingjie,,	huzai9206
Cy3	9606	After fra	GeneChips	Gene expr	All raw d	GPL570	Mingjie,,	huzai9206
Cy3	9606	After fra	GeneChips	Gene expr	All raw d	GPL570	Mingjie,,	huzai9206
Cy3	9606	After fra	GeneChips	Gene expr	All raw d	GPL570	Mingjie,,	huzai9206
Cy3	9606	After fra	GeneChips	Gene expr	All raw d	GPL570	Mingjie,,	huzai9206
Cy3	9606	After fra	GeneChips	Gene expr	All raw d	GPL570	Mingjie,,	huzai9206
Cy3	9606	After fra	GeneChips	Gene expr	All raw d	GPL570	Mingjie,,	huzai9206
Cy3	9606	After fra	GeneChips	Gene expr	All raw d	GPL570	Mingjie,,	huzai9206
Cy3	9606	After fra	GeneChips	Gene expr	All raw d	GPL570	Mingjie,,	huzai9206
Cy3	9606	After fra	GeneChips	Gene expr	All raw d	GPL570	Mingjie,,	huzai9206
Cy3	9606	After fra	GeneChips	Gene expr	All raw d	GPL570	Mingjie,,	huzai9206
Cy3	9606	After fra	GeneChips	Gene expr	All raw d	GPL570	Mingjie,,	huzai9206
Cy3	9606	After fra	GeneChips	Gene expr	All raw d	GPL570	Mingjie,,	huzai9206
Cy3	9606	After fra	GeneChips	Gene expr	All raw d	GPL570	Mingjie,,	huzai9206
Cy3	9606	After fra	GeneChips	Gene expr	All raw d	GPL570	Mingjie,,	huzai9206
Cy3	9606	After fra	GeneChips	Gene expr	All raw d	GPL570	Mingjie,,	huzai9206
Cy3	9606	After fra	GeneChips	Gene expr	All raw d	GPL570	Mingjie,,	huzai9206
Cy3	9606	After fra	GeneChips	Gene expr	All raw d	GPL570	Mingjie,,	huzai9206
Cy3	9606	After fra	GeneChips	Gene expr	All raw d	GPL570	Mingjie,,	huzai9206
Cy3	9606	After fra	GeneChips	Gene expr	All raw d	GPL570	Mingjie,,	huzai9206
Cy3	9606	After fra	GeneChips	Gene expr	All raw d	GPL570	Mingjie,,	huzai9206
Cy3	9606	After fra	GeneChips	Gene expr	All raw d	GPL570	Mingjie,,	huzai9206
Cy3	9606	After fra	GeneChips	Gene expr	All raw d	GPL570	Mingjie,,	huzai9206
Cy3	9606	After fra	GeneChips	Gene expr	All raw d	GPL570	Mingjie,,	huzai9206
Cy3	9606	After fra	GeneChips	Gene expr	All raw d	GPL570	Mingjie,,	huzai9206
Cy3	9606	After fra	GeneChips	Gene expr	All raw d	GPL570	Mingjie,,	huzai9206
Cy3	9606	After fra	GeneChips	Gene expr	All raw d	GPL570	Mingjie,,	huzai9206
Cy3	9606	After fra	GeneChips	Gene expr	All raw d	GPL570	Mingjie,,	huzai9206
Cy3	9606	After fra	GeneChips	Gene expr	All raw d	GPL570	Mingjie,,	huzai9206
Cy3	9606	After fra	GeneChips	Gene expr	All raw d	GPL570	Mingjie,,	huzai9206
Cy3	9606	After fra	GeneChips	Gene expr	All raw d	GPL570	Mingjie,,	huzai9206
Cy3	9606	After fra	GeneChips	Gene expr	All raw d	GPL570	Mingjie,,	huzai9206
Cy3	9606	After fra	GeneChips	Gene expr	All raw d	GPL570	Mingjie,,	huzai9206
Cy3	9606	After fra	GeneChips	Gene expr	All raw d	GPL570	Mingjie,,	huzai9206
Cy3	9606	After fra	GeneChips	Gene expr	All raw d	GPL570	Mingjie,,	huzai9206
Cy3	9606	After fra	GeneChips	Gene expr	All raw d	GPL570	Mingjie,,	huzai9206
Cy3	9606	After fra	GeneChips	Gene expr	All raw d	GPL570	Mingjie,,	huzai9206
Cy3	9606	After fra	GeneChips	Gene expr	All raw d	GPL570	Mingjie,,	huzai9206
Cy3	9606	After fra	GeneChips	Gene expr	All raw d	GPL570	Mingjie,,	huzai9206
Cy3	9606	After fra	GeneChips	Gene expr	All raw d	GPL570	Mingjie,,	huzai9206
Cy3	9606	After fra	GeneChips	Gene expr	All raw d	GPL570	Mingjie,,	huzai9206
Cy3	9606	After fra	GeneChips	Gene expr	All raw d	GPL570	Mingjie,,	huzai9206
Cy3	9606	After fra	GeneChips	Gene expr	All raw d	GPL570	Mingjie,,	huzai9206
Cy3	9606	After fra	GeneChips	Gene expr	All raw d	GPL570	Mingjie,,	huzai9206
Cy3	9606	After fra	GeneChips	Gene expr	All raw d	GPL570	Mingjie,,	huzai9206
Cy3	9606	After fra	GeneChips	Gene expr	All raw d	GPL570	Mingjie,,	huzai9206

For Peer Review

Cy3	9606	After fra	GeneChips	Gene expr	All raw d	GPL570	Mingjie,,	huzai9206
Cy3	9606	After fra	GeneChips	Gene expr	All raw d	GPL570	Mingjie,,	huzai9206
Cy3	9606	After fra	GeneChips	Gene expr	All raw d	GPL570	Mingjie,,	huzai9206
Cy3	9606	After fra	GeneChips	Gene expr	All raw d	GPL570	Mingjie,,	huzai9206
Cy3	9606	After fra	GeneChips	Gene expr	All raw d	GPL570	Mingjie,,	huzai9206
Cy3	9606	After fra	GeneChips	Gene expr	All raw d	GPL570	Mingjie,,	huzai9206
Cy3	9606	After fra	GeneChips	Gene expr	All raw d	GPL570	Mingjie,,	huzai9206
Cy3	9606	After fra	GeneChips	Gene expr	All raw d	GPL570	Mingjie,,	huzai9206
Cy3	9606	After fra	GeneChips	Gene expr	All raw d	GPL570	Mingjie,,	huzai9206
Cy3	9606	After fra	GeneChips	Gene expr	All raw d	GPL570	Mingjie,,	huzai9206
Cy3	9606	After fra	GeneChips	Gene expr	All raw d	GPL570	Mingjie,,	huzai9206
Cy3	9606	After fra	GeneChips	Gene expr	All raw d	GPL570	Mingjie,,	huzai9206
Cy3	9606	After fra	GeneChips	Gene expr	All raw d	GPL570	Mingjie,,	huzai9206
Cy3	9606	After fra	GeneChips	Gene expr	All raw d	GPL570	Mingjie,,	huzai9206
Cy3	9606	After fra	GeneChips	Gene expr	All raw d	GPL570	Mingjie,,	huzai9206
Cy3	9606	After fra	GeneChips	Gene expr	All raw d	GPL570	Mingjie,,	huzai9206
Cy3	9606	After fra	GeneChips	Gene expr	All raw d	GPL570	Mingjie,,	huzai9206
Cy3	9606	After fra	GeneChips	Gene expr	All raw d	GPL570	Mingjie,,	huzai9206
Cy3	9606	After fra	GeneChips	Gene expr	All raw d	GPL570	Mingjie,,	huzai9206
Cy3	9606	After fra	GeneChips	Gene expr	All raw d	GPL570	Mingjie,,	huzai9206
Cy3	9606	After fra	GeneChips	Gene expr	All raw d	GPL570	Mingjie,,	huzai9206
Cy3	9606	After fra	GeneChips	Gene expr	All raw d	GPL570	Mingjie,,	huzai9206
Cy3	9606	After fra	GeneChips	Gene expr	All raw d	GPL570	Mingjie,,	huzai9206
Cy3	9606	After fra	GeneChips	Gene expr	All raw d	GPL570	Mingjie,,	huzai9206
Cy3	9606	After fra	GeneChips	Gene expr	All raw d	GPL570	Mingjie,,	huzai9206
Cy3	9606	After fra	GeneChips	Gene expr	All raw d	GPL570	Mingjie,,	huzai9206
Cy3	9606	After fra	GeneChips	Gene expr	All raw d	GPL570	Mingjie,,	huzai9206
Cy3	9606	After fra	GeneChips	Gene expr	All raw d	GPL570	Mingjie,,	huzai9206
Cy3	9606	After fra	GeneChips	Gene expr	All raw d	GPL570	Mingjie,,	huzai9206
Cy3	9606	After fra	GeneChips	Gene expr	All raw d	GPL570	Mingjie,,	huzai9206
Cy3	9606	After fra	GeneChips	Gene expr	All raw d	GPL570	Mingjie,,	huzai9206
Cy3	9606	After fra	GeneChips	Gene expr	All raw d	GPL570	Mingjie,,	huzai9206
Cy3	9606	After fra	GeneChips	Gene expr	All raw d	GPL570	Mingjie,,	huzai9206
Cy3	9606	After fra	GeneChips	Gene expr	All raw d	GPL570	Mingjie,,	huzai9206
Cy3	9606	After fra	GeneChips	Gene expr	All raw d	GPL570	Mingjie,,	huzai9206
Cy3	9606	After fra	GeneChips	Gene expr	All raw d	GPL570	Mingjie,,	huzai9206
Cy3	9606	After fra	GeneChips	Gene expr	All raw d	GPL570	Mingjie,,	huzai9206
Cy3	9606	After fra	GeneChips	Gene expr	All raw d	GPL570	Mingjie,,	huzai9206
Cy3	9606	After fra	GeneChips	Gene expr	All raw d	GPL570	Mingjie,,	huzai9206
Cy3	9606	After fra	GeneChips	Gene expr	All raw d	GPL570	Mingjie,,	huzai9206
Cy3	9606	After fra	GeneChips	Gene expr	All raw d	GPL570	Mingjie,,	huzai9206
Cy3	9606	After fra	GeneChips	Gene expr	All raw d	GPL570	Mingjie,,	huzai9206
Cy3	9606	After fra	GeneChips	Gene expr	All raw d	GPL570	Mingjie,,	huzai9206
Cy3	9606	After fra	GeneChips	Gene expr	All raw d	GPL570	Mingjie,,	huzai9206
Cy3	9606	After fra	GeneChips	Gene expr	All raw d	GPL570	Mingjie,,	huzai9206
Cy3	9606	After fra	GeneChips	Gene expr	All raw d	GPL570	Mingjie,,	huzai9206
Cy3	9606	After fra	GeneChips	Gene expr	All raw d	GPL570	Mingjie,,	huzai9206
Cy3	9606	After fra	GeneChips	Gene expr	All raw d	GPL570	Mingjie,,	huzai9206
Cy3	9606	After fra	GeneChips	Gene expr	All raw d	GPL570	Mingjie,,	huzai9206
Cy3	9606	After fra	GeneChips	Gene expr	All raw d	GPL570	Mingjie,,	huzai9206
Cy3	9606	After fra	GeneChips	Gene expr	All raw d	GPL570	Mingjie,,	huzai9206
Cy3	9606	After fra	GeneChips	Gene expr	All raw d	GPL570	Mingjie,,	huzai9206
Cy3	9606	After fra	GeneChips	Gene expr	All raw d	GPL570	Mingjie,,	huzai9206
Cy3	9606	After fra	GeneChips	Gene expr	All raw d	GPL570	Mingjie,,	huzai9206
Cy3	9606	After fra	GeneChips	Gene expr	All raw d	GPL570	Mingjie,,	huzai9206
Cy3	9606	After fra	GeneChips	Gene expr	All raw d	GPL570	Mingjie,,	huzai9206
Cy3	9606	After fra	GeneChips	Gene expr	All raw d	GPL570	Mingjie,,	huzai9206
Cy3	9606	After fra	GeneChips	Gene expr	All raw d	GPL570	Mingjie,,	huzai9206

For Peer Review

Cy3	9606	After	fra	GeneChips	Gene	expr	All	raw	d	GPL570	Mingjie,, huzai9206
Cy3	9606	After	fra	GeneChips	Gene	expr	All	raw	d	GPL570	Mingjie,, huzai9206
Cy3	9606	After	fra	GeneChips	Gene	expr	All	raw	d	GPL570	Mingjie,, huzai9206
Cy3	9606	After	fra	GeneChips	Gene	expr	All	raw	d	GPL570	Mingjie,, huzai9206
Cy3	9606	After	fra	GeneChips	Gene	expr	All	raw	d	GPL570	Mingjie,, huzai9206
Cy3	9606	After	fra	GeneChips	Gene	expr	All	raw	d	GPL570	Mingjie,, huzai9206
Cy3	9606	After	fra	GeneChips	Gene	expr	All	raw	d	GPL570	Mingjie,, huzai9206
Cy3	9606	After	fra	GeneChips	Gene	expr	All	raw	d	GPL570	Mingjie,, huzai9206
Cy3	9606	After	fra	GeneChips	Gene	expr	All	raw	d	GPL570	Mingjie,, huzai9206
Cy3	9606	After	fra	GeneChips	Gene	expr	All	raw	d	GPL570	Mingjie,, huzai9206

For Peer Review

!Sample_c	!Sample_c	!Sample_c	!Sample_c	!Sample_c	!Sample_s	!Sample_data_row_count
Ruijin Ho	197 Ruiji	shanghai	200025	China	ftp://ftp	54675
Ruijin Ho	197 Ruiji	shanghai	200025	China	ftp://ftp	54675
Ruijin Ho	197 Ruiji	shanghai	200025	China	ftp://ftp	54675
Ruijin Ho	197 Ruiji	shanghai	200025	China	ftp://ftp	54675
Ruijin Ho	197 Ruiji	shanghai	200025	China	ftp://ftp	54675
Ruijin Ho	197 Ruiji	shanghai	200025	China	ftp://ftp	54675
Ruijin Ho	197 Ruiji	shanghai	200025	China	ftp://ftp	54675
Ruijin Ho	197 Ruiji	shanghai	200025	China	ftp://ftp	54675
Ruijin Ho	197 Ruiji	shanghai	200025	China	ftp://ftp	54675
Ruijin Ho	197 Ruiji	shanghai	200025	China	ftp://ftp	54675
Ruijin Ho	197 Ruiji	shanghai	200025	China	ftp://ftp	54675
Ruijin Ho	197 Ruiji	shanghai	200025	China	ftp://ftp	54675
Ruijin Ho	197 Ruiji	shanghai	200025	China	ftp://ftp	54675
Ruijin Ho	197 Ruiji	shanghai	200025	China	ftp://ftp	54675
Ruijin Ho	197 Ruiji	shanghai	200025	China	ftp://ftp	54675
Ruijin Ho	197 Ruiji	shanghai	200025	China	ftp://ftp	54675
Ruijin Ho	197 Ruiji	shanghai	200025	China	ftp://ftp	54675
Ruijin Ho	197 Ruiji	shanghai	200025	China	ftp://ftp	54675
Ruijin Ho	197 Ruiji	shanghai	200025	China	ftp://ftp	54675
Ruijin Ho	197 Ruiji	shanghai	200025	China	ftp://ftp	54675
Ruijin Ho	197 Ruiji	shanghai	200025	China	ftp://ftp	54675
Ruijin Ho	197 Ruiji	shanghai	200025	China	ftp://ftp	54675
Ruijin Ho	197 Ruiji	shanghai	200025	China	ftp://ftp	54675
Ruijin Ho	197 Ruiji	shanghai	200025	China	ftp://ftp	54675
Ruijin Ho	197 Ruiji	shanghai	200025	China	ftp://ftp	54675
Ruijin Ho	197 Ruiji	shanghai	200025	China	ftp://ftp	54675
Ruijin Ho	197 Ruiji	shanghai	200025	China	ftp://ftp	54675
Ruijin Ho	197 Ruiji	shanghai	200025	China	ftp://ftp	54675
Ruijin Ho	197 Ruiji	shanghai	200025	China	ftp://ftp	54675
Ruijin Ho	197 Ruiji	shanghai	200025	China	ftp://ftp	54675
Ruijin Ho	197 Ruiji	shanghai	200025	China	ftp://ftp	54675
Ruijin Ho	197 Ruiji	shanghai	200025	China	ftp://ftp	54675
Ruijin Ho	197 Ruiji	shanghai	200025	China	ftp://ftp	54675
Ruijin Ho	197 Ruiji	shanghai	200025	China	ftp://ftp	54675
Ruijin Ho	197 Ruiji	shanghai	200025	China	ftp://ftp	54675
Ruijin Ho	197 Ruiji	shanghai	200025	China	ftp://ftp	54675
Ruijin Ho	197 Ruiji	shanghai	200025	China	ftp://ftp	54675
Ruijin Ho	197 Ruiji	shanghai	200025	China	ftp://ftp	54675
Ruijin Ho	197 Ruiji	shanghai	200025	China	ftp://ftp	54675
Ruijin Ho	197 Ruiji	shanghai	200025	China	ftp://ftp	54675
Ruijin Ho	197 Ruiji	shanghai	200025	China	ftp://ftp	54675
Ruijin Ho	197 Ruiji	shanghai	200025	China	ftp://ftp	54675
Ruijin Ho	197 Ruiji	shanghai	200025	China	ftp://ftp	54675
Ruijin Ho	197 Ruiji	shanghai	200025	China	ftp://ftp	54675
Ruijin Ho	197 Ruiji	shanghai	200025	China	ftp://ftp	54675
Ruijin Ho	197 Ruiji	shanghai	200025	China	ftp://ftp	54675
Ruijin Ho	197 Ruiji	shanghai	200025	China	ftp://ftp	54675
Ruijin Ho	197 Ruiji	shanghai	200025	China	ftp://ftp	54675
Ruijin Ho	197 Ruiji	shanghai	200025	China	ftp://ftp	54675
Ruijin Ho	197 Ruiji	shanghai	200025	China	ftp://ftp	54675
Ruijin Ho	197 Ruiji	shanghai	200025	China	ftp://ftp	54675
Ruijin Ho	197 Ruiji	shanghai	200025	China	ftp://ftp	54675
Ruijin Ho	197 Ruiji	shanghai	200025	China	ftp://ftp	54675
Ruijin Ho	197 Ruiji	shanghai	200025	China	ftp://ftp	54675
Ruijin Ho	197 Ruiji	shanghai	200025	China	ftp://ftp	54675
Ruijin Ho	197 Ruiji	shanghai	200025	China	ftp://ftp	54675

For Peer Review

Ruijin Ho	197 Ruiji	shanghai	200025	China	ftp://ftp	54675
Ruijin Ho	197 Ruiji	shanghai	200025	China	ftp://ftp	54675
Ruijin Ho	197 Ruiji	shanghai	200025	China	ftp://ftp	54675
Ruijin Ho	197 Ruiji	shanghai	200025	China	ftp://ftp	54675
Ruijin Ho	197 Ruiji	shanghai	200025	China	ftp://ftp	54675
Ruijin Ho	197 Ruiji	shanghai	200025	China	ftp://ftp	54675
Ruijin Ho	197 Ruiji	shanghai	200025	China	ftp://ftp	54675
Ruijin Ho	197 Ruiji	shanghai	200025	China	ftp://ftp	54675
Ruijin Ho	197 Ruiji	shanghai	200025	China	ftp://ftp	54675
Ruijin Ho	197 Ruiji	shanghai	200025	China	ftp://ftp	54675
Ruijin Ho	197 Ruiji	shanghai	200025	China	ftp://ftp	54675
Ruijin Ho	197 Ruiji	shanghai	200025	China	ftp://ftp	54675
Ruijin Ho	197 Ruiji	shanghai	200025	China	ftp://ftp	54675
Ruijin Ho	197 Ruiji	shanghai	200025	China	ftp://ftp	54675
Ruijin Ho	197 Ruiji	shanghai	200025	China	ftp://ftp	54675
Ruijin Ho	197 Ruiji	shanghai	200025	China	ftp://ftp	54675
Ruijin Ho	197 Ruiji	shanghai	200025	China	ftp://ftp	54675
Ruijin Ho	197 Ruiji	shanghai	200025	China	ftp://ftp	54675
Ruijin Ho	197 Ruiji	shanghai	200025	China	ftp://ftp	54675
Ruijin Ho	197 Ruiji	shanghai	200025	China	ftp://ftp	54675
Ruijin Ho	197 Ruiji	shanghai	200025	China	ftp://ftp	54675
Ruijin Ho	197 Ruiji	shanghai	200025	China	ftp://ftp	54675
Ruijin Ho	197 Ruiji	shanghai	200025	China	ftp://ftp	54675
Ruijin Ho	197 Ruiji	shanghai	200025	China	ftp://ftp	54675
Ruijin Ho	197 Ruiji	shanghai	200025	China	ftp://ftp	54675
Ruijin Ho	197 Ruiji	shanghai	200025	China	ftp://ftp	54675
Ruijin Ho	197 Ruiji	shanghai	200025	China	ftp://ftp	54675
Ruijin Ho	197 Ruiji	shanghai	200025	China	ftp://ftp	54675
Ruijin Ho	197 Ruiji	shanghai	200025	China	ftp://ftp	54675
Ruijin Ho	197 Ruiji	shanghai	200025	China	ftp://ftp	54675
Ruijin Ho	197 Ruiji	shanghai	200025	China	ftp://ftp	54675
Ruijin Ho	197 Ruiji	shanghai	200025	China	ftp://ftp	54675
Ruijin Ho	197 Ruiji	shanghai	200025	China	ftp://ftp	54675
Ruijin Ho	197 Ruiji	shanghai	200025	China	ftp://ftp	54675
Ruijin Ho	197 Ruiji	shanghai	200025	China	ftp://ftp	54675
Ruijin Ho	197 Ruiji	shanghai	200025	China	ftp://ftp	54675
Ruijin Ho	197 Ruiji	shanghai	200025	China	ftp://ftp	54675
Ruijin Ho	197 Ruiji	shanghai	200025	China	ftp://ftp	54675
Ruijin Ho	197 Ruiji	shanghai	200025	China	ftp://ftp	54675
Ruijin Ho	197 Ruiji	shanghai	200025	China	ftp://ftp	54675
Ruijin Ho	197 Ruiji	shanghai	200025	China	ftp://ftp	54675
Ruijin Ho	197 Ruiji	shanghai	200025	China	ftp://ftp	54675
Ruijin Ho	197 Ruiji	shanghai	200025	China	ftp://ftp	54675
Ruijin Ho	197 Ruiji	shanghai	200025	China	ftp://ftp	54675
Ruijin Ho	197 Ruiji	shanghai	200025	China	ftp://ftp	54675
Ruijin Ho	197 Ruiji	shanghai	200025	China	ftp://ftp	54675
Ruijin Ho	197 Ruiji	shanghai	200025	China	ftp://ftp	54675
Ruijin Ho	197 Ruiji	shanghai	200025	China	ftp://ftp	54675
Ruijin Ho	197 Ruiji	shanghai	200025	China	ftp://ftp	54675
Ruijin Ho	197 Ruiji	shanghai	200025	China	ftp://ftp	54675
Ruijin Ho	197 Ruiji	shanghai	200025	China	ftp://ftp	54675
Ruijin Ho	197 Ruiji	shanghai	200025	China	ftp://ftp	54675
Ruijin Ho	197 Ruiji	shanghai	200025	China	ftp://ftp	54675
Ruijin Ho	197 Ruiji	shanghai	200025	China	ftp://ftp	54675
Ruijin Ho	197 Ruiji	shanghai	200025	China	ftp://ftp	54675
Ruijin Ho	197 Ruiji	shanghai	200025	China	ftp://ftp	54675
Ruijin Ho	197 Ruiji	shanghai	200025	China	ftp://ftp	54675
Ruijin Ho	197 Ruiji	shanghai	200025	China	ftp://ftp	54675

For Peer Review

Ruijin Ho	197 Ruiji	shanghai	200025	China	ftp://ftp	54675
Ruijin Ho	197 Ruiji	shanghai	200025	China	ftp://ftp	54675
Ruijin Ho	197 Ruiji	shanghai	200025	China	ftp://ftp	54675
Ruijin Ho	197 Ruiji	shanghai	200025	China	ftp://ftp	54675
Ruijin Ho	197 Ruiji	shanghai	200025	China	ftp://ftp	54675
Ruijin Ho	197 Ruiji	shanghai	200025	China	ftp://ftp	54675
Ruijin Ho	197 Ruiji	shanghai	200025	China	ftp://ftp	54675
Ruijin Ho	197 Ruiji	shanghai	200025	China	ftp://ftp	54675
Ruijin Ho	197 Ruiji	shanghai	200025	China	ftp://ftp	54675
Ruijin Ho	197 Ruiji	shanghai	200025	China	ftp://ftp	54675

For Peer Review

Table S1e. Sample Information of GSE6764

!Sample_t	!Sample_g	!Sample_s	!Sample_s	!Sample_l	!Sample_t	!Sample_c	!Sample_s	!Sample_o
c_NY101	GSM155919	Public on	Jan 17 20	Aug 28 20	RNA	1	human liv	Homo sapi
ci_NY3	GSM155920	Public on	Jan 17 20	Aug 28 20	RNA	1	human liv	Homo sapi
ci_NY17	GSM155921	Public on	Jan 17 20	Aug 28 20	RNA	1	human liv	Homo sapi
ci_NY8	GSM155922	Public on	Jan 17 20	Aug 28 20	RNA	1	human liv	Homo sapi
ci_NY30	GSM155923	Public on	Jan 17 20	Aug 28 20	RNA	1	human liv	Homo sapi
lg_NY23	GSM155924	Public on	Jan 17 20	Aug 28 20	RNA	1	human liv	Homo sapi
lg_NY10	GSM155925	Public on	Jan 17 20	Aug 28 20	RNA	1	human liv	Homo sapi
c_NY102	GSM155926	Public on	Jan 17 20	Aug 28 20	RNA	1	human liv	Homo sapi
c_NY103	GSM155927	Public on	Jan 17 20	Aug 28 20	RNA	1	human liv	Homo sapi
c_NY104	GSM155928	Public on	Jan 17 20	Aug 28 20	RNA	1	human liv	Homo sapi
hg_NY15	GSM155929	Public on	Jan 17 20	Aug 28 20	RNA	1	human liv	Homo sapi
hg_NY28	GSM155930	Public on	Jan 17 20	Aug 28 20	RNA	1	human liv	Homo sapi
ci_NY19	GSM155931	Public on	Jan 17 20	Aug 28 20	RNA	1	human liv	Homo sapi
ve_NY6	GSM155932	Public on	Jan 17 20	Jan 14 20	RNA	1	human liv	Homo sapi
ve_NY26	GSM155933	Public on	Jan 17 20	Jan 14 20	RNA	1	human liv	Homo sapi
ve_NY41	GSM155934	Public on	Jan 17 20	Jan 14 20	RNA	1	human liv	Homo sapi
ve_B5	GSM155935	Public on	Jan 17 20	Aug 28 20	RNA	1	human liv	Homo sapi
aa_NY31	GSM155936	Public on	Jan 17 20	Aug 28 20	RNA	1	human liv	Homo sapi
e_NY2	GSM155937	Public on	Jan 17 20	Jan 14 20	RNA	1	human liv	Homo sapi
a_NY7	GSM155938	Public on	Jan 17 20	Jan 14 20	RNA	1	human liv	Homo sapi
e_NY16	GSM155939	Public on	Jan 17 20	Jan 14 20	RNA	1	human liv	Homo sapi
a_NY18	GSM155940	Public on	Jan 17 20	Jan 14 20	RNA	1	human liv	Homo sapi
a_NY34	GSM155941	Public on	Jan 17 20	Jan 14 20	RNA	1	human liv	Homo sapi
a_NY36	GSM155942	Public on	Jan 17 20	Jan 14 20	RNA	1	human liv	Homo sapi
aa_NY39	GSM155943	Public on	Jan 17 20	Jan 14 20	RNA	1	human liv	Homo sapi
aa_NY226	GSM155944	Public on	Jan 17 20	Jan 14 20	RNA	1	human liv	Homo sapi
e_B7	GSM155945	Public on	Jan 17 20	Aug 28 20	RNA	1	human liv	Homo sapi
e_B13	GSM155946	Public on	Jan 17 20	Jan 14 20	RNA	1	human liv	Homo sapi
c_B51	GSM155947	Public on	Jan 17 20	Aug 28 20	RNA	1	human liv	Homo sapi
c_B53	GSM155948	Public on	Jan 17 20	Aug 28 20	RNA	1	human liv	Homo sapi
aa_NY218	GSM155949	Public on	Jan 17 20	Jan 14 20	RNA	1	human liv	Homo sapi
aa_NY234	GSM155950	Public on	Jan 17 20	Aug 28 20	RNA	1	human liv	Homo sapi
cin_NY20	GSM155951	Public on	Jan 17 20	Aug 28 20	RNA	1	human liv	Homo sapi
cin_NY50	GSM155952	Public on	Jan 17 20	Aug 28 20	RNA	1	human liv	Homo sapi
aa_NY224	GSM155953	Public on	Jan 17 20	Jan 14 20	RNA	1	human liv	Homo sapi
aa_NY216	GSM155954	Public on	Jan 17 20	Jan 14 20	RNA	1	human liv	Homo sapi
e_M45B	GSM155955	Public on	Jan 17 20	Aug 28 20	RNA	1	human liv	Homo sapi
aa_NY79	GSM155956	Public on	Jan 17 20	Jan 14 20	RNA	1	human liv	Homo sapi
aa_NY82	GSM155957	Public on	Jan 17 20	Jan 14 20	RNA	1	human liv	Homo sapi
e_NY204	GSM155958	Public on	Jan 17 20	Jan 14 20	RNA	1	human liv	Homo sapi
e_NY44	GSM155959	Public on	Jan 17 20	Jan 14 20	RNA	1	human liv	Homo sapi
e_M75	GSM155960	Public on	Jan 17 20	Aug 28 20	RNA	1	human liv	Homo sapi
c_M101	GSM155961	Public on	Jan 17 20	Aug 28 20	RNA	1	human liv	Homo sapi
aa_NY74	GSM155962	Public on	Jan 17 20	Aug 28 20	RNA	1	human liv	Homo sapi
a_NY70	GSM155963	Public on	Jan 17 20	Jan 14 20	RNA	1	human liv	Homo sapi
c_NY106	GSM155964	Public on	Jan 17 20	Aug 28 20	RNA	1	human liv	Homo sapi
ci_NY78	GSM155965	Public on	Jan 17 20	Aug 28 20	RNA	1	human liv	Homo sapi
ci_NY33	GSM155966	Public on	Jan 17 20	Aug 28 20	RNA	1	human liv	Homo sapi
ci_NY35	GSM155967	Public on	Jan 17 20	Aug 28 20	RNA	1	human liv	Homo sapi
ci_NY37	GSM155968	Public on	Jan 17 20	Aug 28 20	RNA	1	human liv	Homo sapi
ci_NY43	GSM155969	Public on	Jan 17 20	Aug 28 20	RNA	1	human liv	Homo sapi
hg_NY58	GSM155970	Public on	Jan 17 20	Aug 28 20	RNA	1	human liv	Homo sapi
lg_NY48	GSM155971	Public on	Jan 17 20	Aug 28 20	RNA	1	human liv	Homo sapi
lg_NY51	GSM155972	Public on	Jan 17 20	Aug 28 20	RNA	1	human liv	Homo sapi
lg_NY56	GSM155973	Public on	Jan 17 20	Aug 28 20	RNA	1	human liv	Homo sapi
lg_NY57	GSM155974	Public on	Jan 17 20	Aug 28 20	RNA	1	human liv	Homo sapi

For Peer Review

hg_NY61	GSM155975	Public on Jan 17 20	Aug 28 20	RNA	1	human liv	Homo sapi
lg_NY49	GSM155976	Public on Jan 17 20	Aug 28 20	RNA	1	human liv	Homo sapi
hg_NY55	GSM155977	Public on Jan 17 20	Aug 28 20	RNA	1	human liv	Homo sapi
lg_NY11	GSM155978	Public on Jan 17 20	Aug 28 20	RNA	1	human liv	Homo sapi
lg_NY13	GSM155979	Public on Jan 17 20	Aug 28 20	RNA	1	human liv	Homo sapi
lg_NY24	GSM155980	Public on Jan 17 20	Aug 28 20	RNA	1	human liv	Homo sapi
hg_NY29	GSM155981	Public on Jan 17 20	Aug 28 20	RNA	1	human liv	Homo sapi
hg_NY53	GSM155982	Public on Jan 17 20	Aug 28 20	RNA	1	human liv	Homo sapi
ve_M47	GSM155983	Public on Jan 17 20	Aug 28 20	RNA	1	human liv	Homo sapi
cin_NY42B	GSM155984	Public on Jan 17 20	Aug 28 20	RNA	1	human liv	Homo sapi
a_B35B	GSM155985	Public on Jan 17 20	Jan 14 20	RNA	1	human liv	Homo sapi
e_M43B	GSM155986	Public on Jan 17 20	Jan 14 20	RNA	1	human liv	Homo sapi
ve_M63B	GSM155987	Public on Jan 17 20	Aug 28 20	RNA	1	human liv	Homo sapi
C_C6	GSM155988	Public on Jan 17 20	Aug 28 20	RNA	1	human liv	Homo sapi
C_C7	GSM155989	Public on Jan 17 20	Aug 28 20	RNA	1	human liv	Homo sapi
A_2m25	GSM155990	Public on Jan 17 20	Aug 28 20	RNA	1	human liv	Homo sapi
ve_2m41	GSM155991	Public on Jan 17 20	Aug 28 20	RNA	1	human liv	Homo sapi
e_2m51	GSM155992	Public on Jan 17 20	Aug 28 20	RNA	1	human liv	Homo sapi
ve_2m57	GSM155993	Public on Jan 17 20	Aug 28 20	RNA	1	human liv	Homo sapi

For Peer Review

!Sample_c	!Sample_m	!Sample_e	!Sample_l	!Sample_l	!Sample_t	!Sample_h	!Sample_s	!Sample_d
normal li	total RNA	Trizol ex	biotin	Biotinyla	9606	Following	Affymetri	normal li
cirrhotic	total RNA	Trizol ex	biotin	Biotinyla	9606	Following	Affymetri	cirrhotic
cirrhotic	total RNA	Trizol ex	biotin	Biotinyla	9606	Following	Affymetri	cirrhotic
cirrhotic	total RNA	Trizol ex	biotin	Biotinyla	9606	Following	Affymetri	cirrhotic
cirrhotic	total RNA	Trizol ex	biotin	Biotinyla	9606	Following	Affymetri	cirrhotic
low-grade	total RNA	Trizol ex	biotin	Biotinyla	9606	Following	Affymetri	low-grade
low-grade	total RNA	Trizol ex	biotin	Biotinyla	9606	Following	Affymetri	low-grade
normal li	total RNA	Trizol ex	biotin	Biotinyla	9606	Following	Affymetri	normal li
normal li	total RNA	Trizol ex	biotin	Biotinyla	9606	Following	Affymetri	normal li
normal li	total RNA	Trizol ex	biotin	Biotinyla	9606	Following	Affymetri	normal li
high-grad	total RNA	Trizol ex	biotin	Biotinyla	9606	Following	Affymetri	high-grad
high-grad	total RNA	Trizol ex	biotin	Biotinyla	9606	Following	Affymetri	high-grad
cirrhotic	total RNA	Trizol ex	biotin	Biotinyla	9606	Following	Affymetri	cirrhotic
very earl	total RNA	Trizol ex	biotin	Biotinyla	9606	Following	Affymetri	very earl
very earl	total RNA	Trizol ex	biotin	Biotinyla	9606	Following	Affymetri	very earl
very earl	total RNA	Trizol ex	biotin	Biotinyla	9606	Following	Affymetri	very earl
very earl	total RNA	Trizol ex	biotin	Biotinyla	9606	Following	Affymetri	very earl
very adva	total RNA	Trizol ex	biotin	Biotinyla	9606	Following	Affymetri	very adva
early HCC	total RNA	Trizol ex	biotin	Biotinyla	9606	Following	Affymetri	early HCC
advanced	total RNA	Trizol ex	biotin	Biotinyla	9606	Following	Affymetri	advanced
early HCC	total RNA	Trizol ex	biotin	Biotinyla	9606	Following	Affymetri	early HCC
advanced	total RNA	Trizol ex	biotin	Biotinyla	9606	Following	Affymetri	advanced
advanced	total RNA	Trizol ex	biotin	Biotinyla	9606	Following	Affymetri	advanced
advanced	total RNA	Trizol ex	biotin	Biotinyla	9606	Following	Affymetri	advanced
very adva	total RNA	Trizol ex	biotin	Biotinyla	9606	Following	Affymetri	very adva
very adva	total RNA	Trizol ex	biotin	Biotinyla	9606	Following	Affymetri	very adva
early HCC	total RNA	Trizol ex	biotin	Biotinyla	9606	Following	Affymetri	early HCC
early HCC	total RNA	Trizol ex	biotin	Biotinyla	9606	Following	Affymetri	early HCC
normal li	total RNA	Trizol ex	biotin	Biotinyla	9606	Following	Affymetri	normal li
normal li	total RNA	Trizol ex	biotin	Biotinyla	9606	Following	Affymetri	normal li
very adva	total RNA	Trizol ex	biotin	Biotinyla	9606	Following	Affymetri	very adva
very adva	total RNA	Trizol ex	biotin	Biotinyla	9606	Following	Affymetri	very adva
cirrhotic	total RNA	Trizol ex	biotin	Biotinyla	9606	Following	Affymetri	cirrhotic
cirrhotic	total RNA	Trizol ex	biotin	Biotinyla	9606	Following	Affymetri	cirrhotic
very adva	total RNA	Trizol ex	biotin	Biotinyla	9606	Following	Affymetri	very adva
very adva	total RNA	Trizol ex	biotin	Biotinyla	9606	Following	Affymetri	very adva
early HCC	total RNA	Trizol ex	biotin	Biotinyla	9606	Following	Affymetri	early HCC
very adva	total RNA	Trizol ex	biotin	Biotinyla	9606	Following	Affymetri	very adva
very adva	total RNA	Trizol ex	biotin	Biotinyla	9606	Following	Affymetri	very adva
early HCC	total RNA	Trizol ex	biotin	Biotinyla	9606	Following	Affymetri	early HCC
early HCC	total RNA	Trizol ex	biotin	Biotinyla	9606	Following	Affymetri	early HCC
early HCC	total RNA	Trizol ex	biotin	Biotinyla	9606	Following	Affymetri	early HCC
normal li	total RNA	Trizol ex	biotin	Biotinyla	9606	Following	Affymetri	normal li
very adva	total RNA	Trizol ex	biotin	Biotinyla	9606	Following	Affymetri	very adva
advanced	total RNA	Trizol ex	biotin	Biotinyla	9606	Following	Affymetri	advanced
normal li	total RNA	Trizol ex	biotin	Biotinyla	9606	Following	Affymetri	normal li
cirrhotic	total RNA	Trizol ex	biotin	Biotinyla	9606	Following	Affymetri	cirrhotic
cirrhotic	total RNA	Trizol ex	biotin	Biotinyla	9606	Following	Affymetri	cirrhotic
cirrhotic	total RNA	Trizol ex	biotin	Biotinyla	9606	Following	Affymetri	cirrhotic
cirrhotic	total RNA	Trizol ex	biotin	Biotinyla	9606	Following	Affymetri	cirrhotic
cirrhotic	total RNA	Trizol ex	biotin	Biotinyla	9606	Following	Affymetri	cirrhotic
high-grad	total RNA	Trizol ex	biotin	Biotinyla	9606	Following	Affymetri	high-grad
low-grade	total RNA	Trizol ex	biotin	Biotinyla	9606	Following	Affymetri	low-grade
low-grade	total RNA	Trizol ex	biotin	Biotinyla	9606	Following	Affymetri	low-grade
low-grade	total RNA	Trizol ex	biotin	Biotinyla	9606	Following	Affymetri	low-grade
low-grade	total RNA	Trizol ex	biotin	Biotinyla	9606	Following	Affymetri	low-grade

For Peer Review

high-grad	total RNA	Trizol ex	biotin	Biotinyla	9606	Following	Affymetri	high-grad
low-grade	total RNA	Trizol ex	biotin	Biotinyla	9606	Following	Affymetri	low-grade
high-grad	total RNA	Trizol ex	biotin	Biotinyla	9606	Following	Affymetri	high-grad
low-grade	total RNA	Trizol ex	biotin	Biotinyla	9606	Following	Affymetri	low-grade
low-grade	total RNA	Trizol ex	biotin	Biotinyla	9606	Following	Affymetri	low-grade
low-grade	total RNA	Trizol ex	biotin	Biotinyla	9606	Following	Affymetri	low-grade
high-grad	total RNA	Trizol ex	biotin	Biotinyla	9606	Following	Affymetri	high-grad
high-grad	total RNA	Trizol ex	biotin	Biotinyla	9606	Following	Affymetri	high-grad
very earl	total RNA	Trizol ex	biotin	Biotinyla	9606	Following	Affymetri	very earl
cirrhotic	total RNA	Trizol ex	biotin	Biotinyla	9606	Following	Affymetri	cirrhotic
advanced	total RNA	Trizol ex	biotin	Biotinyla	9606	Following	Affymetri	advanced
early HCC	total RNA	Trizol ex	biotin	Biotinyla	9606	Following	Affymetri	early HCC
very earl	total RNA	Trizol ex	biotin	Biotinyla	9606	Following	Affymetri	very earl
normal li	total RNA	Trizol ex	biotin	Biotinyla	9606	Following	Affymetri	normal li
normal li	total RNA	Trizol ex	biotin	Biotinyla	9606	Following	Affymetri	normal li
advanced	total RNA	Trizol ex	biotin	Biotinyla	9606	Following	Affymetri	advanced
very earl	total RNA	Trizol ex	biotin	Biotinyla	9606	Following	Affymetri	very earl
early HCC	total RNA	Trizol ex	biotin	Biotinyla	9606	Following	Affymetri	early HCC
very earl	total RNA	Trizol ex	biotin	Biotinyla	9606	Following	Affymetri	very earl

For Peer Review

!Sample_d	!Sample_p	!Sample_c	!Sample_c	!Sample_c	!Sample_c	!Sample_c	!Sample_c	!Sample_c
MAS probe	GPL570	Elisa,,Wu	Elisa.Wur	212-241-9	Sam Waxma	Hematolog	Mount Sin	1 Gustave
MAS probe	GPL570	Elisa,,Wu	Elisa.Wur	212-241-9	Sam Waxma	Hematolog	Mount Sin	1 Gustave
MAS probe	GPL570	Elisa,,Wu	Elisa.Wur	212-241-9	Sam Waxma	Hematolog	Mount Sin	1 Gustave
MAS probe	GPL570	Elisa,,Wu	Elisa.Wur	212-241-9	Sam Waxma	Hematolog	Mount Sin	1 Gustave
MAS probe	GPL570	Elisa,,Wu	Elisa.Wur	212-241-9	Sam Waxma	Hematolog	Mount Sin	1 Gustave
MAS probe	GPL570	Elisa,,Wu	Elisa.Wur	212-241-9	Sam Waxma	Hematolog	Mount Sin	1 Gustave
MAS probe	GPL570	Elisa,,Wu	Elisa.Wur	212-241-9	Sam Waxma	Hematolog	Mount Sin	1 Gustave
MAS probe	GPL570	Elisa,,Wu	Elisa.Wur	212-241-9	Sam Waxma	Hematolog	Mount Sin	1 Gustave
MAS probe	GPL570	Elisa,,Wu	Elisa.Wur	212-241-9	Sam Waxma	Hematolog	Mount Sin	1 Gustave
MAS probe	GPL570	Elisa,,Wu	Elisa.Wur	212-241-9	Sam Waxma	Hematolog	Mount Sin	1 Gustave
MAS probe	GPL570	Elisa,,Wu	Elisa.Wur	212-241-9	Sam Waxma	Hematolog	Mount Sin	1 Gustave
MAS probe	GPL570	Elisa,,Wu	Elisa.Wur	212-241-9	Sam Waxma	Hematolog	Mount Sin	1 Gustave
MAS probe	GPL570	Elisa,,Wu	Elisa.Wur	212-241-9	Sam Waxma	Hematolog	Mount Sin	1 Gustave
MAS probe	GPL570	Elisa,,Wu	Elisa.Wur	212-241-9	Sam Waxma	Hematolog	Mount Sin	1 Gustave
MAS probe	GPL570	Elisa,,Wu	Elisa.Wur	212-241-9	Sam Waxma	Hematolog	Mount Sin	1 Gustave
MAS probe	GPL570	Elisa,,Wu	Elisa.Wur	212-241-9	Sam Waxma	Hematolog	Mount Sin	1 Gustave
MAS probe	GPL570	Elisa,,Wu	Elisa.Wur	212-241-9	Sam Waxma	Hematolog	Mount Sin	1 Gustave
MAS probe	GPL570	Elisa,,Wu	Elisa.Wur	212-241-9	Sam Waxma	Hematolog	Mount Sin	1 Gustave
MAS probe	GPL570	Elisa,,Wu	Elisa.Wur	212-241-9	Sam Waxma	Hematolog	Mount Sin	1 Gustave
MAS probe	GPL570	Elisa,,Wu	Elisa.Wur	212-241-9	Sam Waxma	Hematolog	Mount Sin	1 Gustave
MAS probe	GPL570	Elisa,,Wu	Elisa.Wur	212-241-9	Sam Waxma	Hematolog	Mount Sin	1 Gustave
MAS probe	GPL570	Elisa,,Wu	Elisa.Wur	212-241-9	Sam Waxma	Hematolog	Mount Sin	1 Gustave
MAS probe	GPL570	Elisa,,Wu	Elisa.Wur	212-241-9	Sam Waxma	Hematolog	Mount Sin	1 Gustave
MAS probe	GPL570	Elisa,,Wu	Elisa.Wur	212-241-9	Sam Waxma	Hematolog	Mount Sin	1 Gustave
MAS probe	GPL570	Elisa,,Wu	Elisa.Wur	212-241-9	Sam Waxma	Hematolog	Mount Sin	1 Gustave
MAS probe	GPL570	Elisa,,Wu	Elisa.Wur	212-241-9	Sam Waxma	Hematolog	Mount Sin	1 Gustave
MAS probe	GPL570	Elisa,,Wu	Elisa.Wur	212-241-9	Sam Waxma	Hematolog	Mount Sin	1 Gustave
MAS probe	GPL570	Elisa,,Wu	Elisa.Wur	212-241-9	Sam Waxma	Hematolog	Mount Sin	1 Gustave
MAS probe	GPL570	Elisa,,Wu	Elisa.Wur	212-241-9	Sam Waxma	Hematolog	Mount Sin	1 Gustave
MAS probe	GPL570	Elisa,,Wu	Elisa.Wur	212-241-9	Sam Waxma	Hematolog	Mount Sin	1 Gustave
MAS probe	GPL570	Elisa,,Wu	Elisa.Wur	212-241-9	Sam Waxma	Hematolog	Mount Sin	1 Gustave
MAS probe	GPL570	Elisa,,Wu	Elisa.Wur	212-241-9	Sam Waxma	Hematolog	Mount Sin	1 Gustave
MAS probe	GPL570	Elisa,,Wu	Elisa.Wur	212-241-9	Sam Waxma	Hematolog	Mount Sin	1 Gustave
MAS probe	GPL570	Elisa,,Wu	Elisa.Wur	212-241-9	Sam Waxma	Hematolog	Mount Sin	1 Gustave
MAS probe	GPL570	Elisa,,Wu	Elisa.Wur	212-241-9	Sam Waxma	Hematolog	Mount Sin	1 Gustave
MAS probe	GPL570	Elisa,,Wu	Elisa.Wur	212-241-9	Sam Waxma	Hematolog	Mount Sin	1 Gustave
MAS probe	GPL570	Elisa,,Wu	Elisa.Wur	212-241-9	Sam Waxma	Hematolog	Mount Sin	1 Gustave
MAS probe	GPL570	Elisa,,Wu	Elisa.Wur	212-241-9	Sam Waxma	Hematolog	Mount Sin	1 Gustave
MAS probe	GPL570	Elisa,,Wu	Elisa.Wur	212-241-9	Sam Waxma	Hematolog	Mount Sin	1 Gustave
MAS probe	GPL570	Elisa,,Wu	Elisa.Wur	212-241-9	Sam Waxma	Hematolog	Mount Sin	1 Gustave
MAS probe	GPL570	Elisa,,Wu	Elisa.Wur	212-241-9	Sam Waxma	Hematolog	Mount Sin	1 Gustave
MAS probe	GPL570	Elisa,,Wu	Elisa.Wur	212-241-9	Sam Waxma	Hematolog	Mount Sin	1 Gustave
MAS probe	GPL570	Elisa,,Wu	Elisa.Wur	212-241-9	Sam Waxma	Hematolog	Mount Sin	1 Gustave
MAS probe	GPL570	Elisa,,Wu	Elisa.Wur	212-241-9	Sam Waxma	Hematolog	Mount Sin	1 Gustave
MAS probe	GPL570	Elisa,,Wu	Elisa.Wur	212-241-9	Sam Waxma	Hematolog	Mount Sin	1 Gustave
MAS probe	GPL570	Elisa,,Wu	Elisa.Wur	212-241-9	Sam Waxma	Hematolog	Mount Sin	1 Gustave
MAS probe	GPL570	Elisa,,Wu	Elisa.Wur	212-241-9	Sam Waxma	Hematolog	Mount Sin	1 Gustave
MAS probe	GPL570	Elisa,,Wu	Elisa.Wur	212-241-9	Sam Waxma	Hematolog	Mount Sin	1 Gustave
MAS probe	GPL570	Elisa,,Wu	Elisa.Wur	212-241-9	Sam Waxma	Hematolog	Mount Sin	1 Gustave
MAS probe	GPL570	Elisa,,Wu	Elisa.Wur	212-241-9	Sam Waxma	Hematolog	Mount Sin	1 Gustave
MAS probe	GPL570	Elisa,,Wu	Elisa.Wur	212-241-9	Sam Waxma	Hematolog	Mount Sin	1 Gustave
MAS probe	GPL570	Elisa,,Wu	Elisa.Wur	212-241-9	Sam Waxma	Hematolog	Mount Sin	1 Gustave
MAS probe	GPL570	Elisa,,Wu	Elisa.Wur	212-241-9	Sam Waxma	Hematolog	Mount Sin	1 Gustave
MAS probe	GPL570	Elisa,,Wu	Elisa.Wur	212-241-9	Sam Waxma	Hematolog	Mount Sin	1 Gustave
MAS probe	GPL570	Elisa,,Wu	Elisa.Wur	212-241-9	Sam Waxma	Hematolog	Mount Sin	1 Gustave
MAS probe	GPL570	Elisa,,Wu	Elisa.Wur	212-241-9	Sam Waxma	Hematolog	Mount Sin	1 Gustave

For Peer Review

MAS	probe	GPL570	Elisa,,	Wu	Elisa.	Wur	212-241-9	Sam	Waxma	Hematolog	Mount	Sin 1	Gustave
MAS	probe	GPL570	Elisa,,	Wu	Elisa.	Wur	212-241-9	Sam	Waxma	Hematolog	Mount	Sin 1	Gustave
MAS	probe	GPL570	Elisa,,	Wu	Elisa.	Wur	212-241-9	Sam	Waxma	Hematolog	Mount	Sin 1	Gustave
MAS	probe	GPL570	Elisa,,	Wu	Elisa.	Wur	212-241-9	Sam	Waxma	Hematolog	Mount	Sin 1	Gustave
MAS	probe	GPL570	Elisa,,	Wu	Elisa.	Wur	212-241-9	Sam	Waxma	Hematolog	Mount	Sin 1	Gustave
MAS	probe	GPL570	Elisa,,	Wu	Elisa.	Wur	212-241-9	Sam	Waxma	Hematolog	Mount	Sin 1	Gustave
MAS	probe	GPL570	Elisa,,	Wu	Elisa.	Wur	212-241-9	Sam	Waxma	Hematolog	Mount	Sin 1	Gustave
MAS	probe	GPL570	Elisa,,	Wu	Elisa.	Wur	212-241-9	Sam	Waxma	Hematolog	Mount	Sin 1	Gustave
MAS	probe	GPL570	Elisa,,	Wu	Elisa.	Wur	212-241-9	Sam	Waxma	Hematolog	Mount	Sin 1	Gustave
MAS	probe	GPL570	Elisa,,	Wu	Elisa.	Wur	212-241-9	Sam	Waxma	Hematolog	Mount	Sin 1	Gustave
MAS	probe	GPL570	Elisa,,	Wu	Elisa.	Wur	212-241-9	Sam	Waxma	Hematolog	Mount	Sin 1	Gustave
MAS	probe	GPL570	Elisa,,	Wu	Elisa.	Wur	212-241-9	Sam	Waxma	Hematolog	Mount	Sin 1	Gustave
MAS	probe	GPL570	Elisa,,	Wu	Elisa.	Wur	212-241-9	Sam	Waxma	Hematolog	Mount	Sin 1	Gustave
MAS	probe	GPL570	Elisa,,	Wu	Elisa.	Wur	212-241-9	Sam	Waxma	Hematolog	Mount	Sin 1	Gustave
MAS	probe	GPL570	Elisa,,	Wu	Elisa.	Wur	212-241-9	Sam	Waxma	Hematolog	Mount	Sin 1	Gustave
MAS	probe	GPL570	Elisa,,	Wu	Elisa.	Wur	212-241-9	Sam	Waxma	Hematolog	Mount	Sin 1	Gustave
MAS	probe	GPL570	Elisa,,	Wu	Elisa.	Wur	212-241-9	Sam	Waxma	Hematolog	Mount	Sin 1	Gustave
MAS	probe	GPL570	Elisa,,	Wu	Elisa.	Wur	212-241-9	Sam	Waxma	Hematolog	Mount	Sin 1	Gustave
MAS	probe	GPL570	Elisa,,	Wu	Elisa.	Wur	212-241-9	Sam	Waxma	Hematolog	Mount	Sin 1	Gustave
MAS	probe	GPL570	Elisa,,	Wu	Elisa.	Wur	212-241-9	Sam	Waxma	Hematolog	Mount	Sin 1	Gustave

For Peer Review

!Sample_c	!Sample_c	!Sample_c	!Sample_c	!Sample_s	!Sample_d	!Sample_r	!Sample_relation	
New York	NY	10029	USA	ftp://ftp	54675	Reanalyze	Reanalyzed by: GSE1	
New York	NY	10029	USA	ftp://ftp	54675	Reanalyze	Reanalyzed by: GSE1	
New York	NY	10029	USA	ftp://ftp	54675	Reanalyze	Reanalyzed by: GSE1	
New York	NY	10029	USA	ftp://ftp	54675	Reanalyze	Reanalyzed by: GSE1	
New York	NY	10029	USA	ftp://ftp	54675	Reanalyze	Reanalyzed by: GSE1	
New York	NY	10029	USA	ftp://ftp	54675	Reanalyze	Reanalyzed by: GSE1	
New York	NY	10029	USA	ftp://ftp	54675	Reanalyze	Reanalyzed by: GSE1	
New York	NY	10029	USA	ftp://ftp	54675	Reanalyze	Reanalyzed by: GSE1	
New York	NY	10029	USA	ftp://ftp	54675	Reanalyze	Reanalyzed by: GSE1	
New York	NY	10029	USA	ftp://ftp	54675	Reanalyze	Reanalyzed by: GSE1	
New York	NY	10029	USA	ftp://ftp	54675	Reanalyze	Reanalyzed by: GSE1	
New York	NY	10029	USA	ftp://ftp	54675	Reanalyze	Reanalyzed by: GSE1	
New York	NY	10029	USA	ftp://ftp	54675	Reanalyze	Reanalyzed by: GSE1	
New York	NY	10029	USA	ftp://ftp	54675	Reanalyzed by: GSE64985		
New York	NY	10029	USA	ftp://ftp	54675	Reanalyzed by: GSE64985		
New York	NY	10029	USA	ftp://ftp	54675	Reanalyzed by: GSE64985		
New York	NY	10029	USA	ftp://ftp	54675	Reanalyze	Reanalyzed by: GSE1	
New York	NY	10029	USA	ftp://ftp	54675	Reanalyze	Reanalyzed by: GSE1	
New York	NY	10029	USA	ftp://ftp	54675	Reanalyzed by: GSE64985		
New York	NY	10029	USA	ftp://ftp	54675	Reanalyzed by: GSE64985		
New York	NY	10029	USA	ftp://ftp	54675	Reanalyzed by: GSE64985		
New York	NY	10029	USA	ftp://ftp	54675	Reanalyzed by: GSE64985		
New York	NY	10029	USA	ftp://ftp	54675	Reanalyzed by: GSE64985		
New York	NY	10029	USA	ftp://ftp	54675	Reanalyzed by: GSE64985		
New York	NY	10029	USA	ftp://ftp	54675	Reanalyzed by: GSE64985		
New York	NY	10029	USA	ftp://ftp	54675	Reanalyzed by: GSE64985		
New York	NY	10029	USA	ftp://ftp	54675	Reanalyze	Reanalyzed by: GSE1	
New York	NY	10029	USA	ftp://ftp	54675	Reanalyzed by: GSE64985		
New York	NY	10029	USA	ftp://ftp	54675	Reanalyze	Reanalyzed by: GSE1	
New York	NY	10029	USA	ftp://ftp	54675	Reanalyze	Reanalyzed by: GSE1	
New York	NY	10029	USA	ftp://ftp	54675	Reanalyzed by: GSE64985		
New York	NY	10029	USA	ftp://ftp	54675	Reanalyze	Reanalyzed by: GSE1	
New York	NY	10029	USA	ftp://ftp	54675	Reanalyze	Reanalyzed by: GSE1	
New York	NY	10029	USA	ftp://ftp	54675	Reanalyze	Reanalyzed by: GSE1	
New York	NY	10029	USA	ftp://ftp	54675	Reanalyzed by: GSE64985		
New York	NY	10029	USA	ftp://ftp	54675	Reanalyzed by: GSE64985		
New York	NY	10029	USA	ftp://ftp	54675	Reanalyze	Reanalyzed by: GSE1	
New York	NY	10029	USA	ftp://ftp	54675	Reanalyzed by: GSE64985		
New York	NY	10029	USA	ftp://ftp	54675	Reanalyzed by: GSE64985		
New York	NY	10029	USA	ftp://ftp	54675	Reanalyzed by: GSE64985		
New York	NY	10029	USA	ftp://ftp	54675	Reanalyzed by: GSE64985		
New York	NY	10029	USA	ftp://ftp	54675	Reanalyze	Reanalyzed by: GSE1	
New York	NY	10029	USA	ftp://ftp	54675	Reanalyze	Reanalyzed by: GSE1	
New York	NY	10029	USA	ftp://ftp	54675	Reanalyze	Reanalyzed by: GSE1	
New York	NY	10029	USA	ftp://ftp	54675	Reanalyzed by: GSE64985		
New York	NY	10029	USA	ftp://ftp	54675	Reanalyze	Reanalyzed by: GSE1	
New York	NY	10029	USA	ftp://ftp	54675	Reanalyze	Reanalyzed by: GSE1	
New York	NY	10029	USA	ftp://ftp	54675	Reanalyze	Reanalyzed by: GSE1	
New York	NY	10029	USA	ftp://ftp	54675	Reanalyze	Reanalyzed by: GSE1	
New York	NY	10029	USA	ftp://ftp	54675	Reanalyze	Reanalyzed by: GSE1	
New York	NY	10029	USA	ftp://ftp	54675	Reanalyze	Reanalyzed by: GSE1	
New York	NY	10029	USA	ftp://ftp	54675	Reanalyze	Reanalyzed by: GSE1	
New York	NY	10029	USA	ftp://ftp	54675	Reanalyze	Reanalyzed by: GSE1	
New York	NY	10029	USA	ftp://ftp	54675	Reanalyze	Reanalyzed by: GSE1	
New York	NY	10029	USA	ftp://ftp	54675	Reanalyze	Reanalyzed by: GSE1	
New York	NY	10029	USA	ftp://ftp	54675	Reanalyze	Reanalyzed by: GSE1	

For Peer Review

New York	NY	10029	USA	ftp://ftp	54675	Reanalyze	Reanalyzed by: GSE1
New York	NY	10029	USA	ftp://ftp	54675	Reanalyze	Reanalyzed by: GSE1
New York	NY	10029	USA	ftp://ftp	54675	Reanalyze	Reanalyzed by: GSE1
New York	NY	10029	USA	ftp://ftp	54675	Reanalyze	Reanalyzed by: GSE1
New York	NY	10029	USA	ftp://ftp	54675	Reanalyze	Reanalyzed by: GSE1
New York	NY	10029	USA	ftp://ftp	54675	Reanalyze	Reanalyzed by: GSE1
New York	NY	10029	USA	ftp://ftp	54675	Reanalyze	Reanalyzed by: GSE1
New York	NY	10029	USA	ftp://ftp	54675	Reanalyze	Reanalyzed by: GSE1
New York	NY	10029	USA	ftp://ftp	54675	Reanalyze	Reanalyzed by: GSE1
New York	NY	10029	USA	ftp://ftp	54675	Reanalyze	Reanalyzed by: GSE1
New York	NY	10029	USA	ftp://ftp	54675	Reanalyzed by: GSE64985	
New York	NY	10029	USA	ftp://ftp	54675	Reanalyzed by: GSE64985	
New York	NY	10029	USA	ftp://ftp	54675	Reanalyze	Reanalyzed by: GSE1
New York	NY	10029	USA	ftp://ftp	54675	Reanalyze	Reanalyzed by: GSE1
New York	NY	10029	USA	ftp://ftp	54675	Reanalyze	Reanalyzed by: GSE1
New York	NY	10029	USA	ftp://ftp	54675	Reanalyze	Reanalyzed by: GSE1
New York	NY	10029	USA	ftp://ftp	54675	Reanalyze	Reanalyzed by: GSE1
New York	NY	10029	USA	ftp://ftp	54675	Reanalyze	Reanalyzed by: GSE1
New York	NY	10029	USA	ftp://ftp	54675	Reanalyze	Reanalyzed by: GSE1

For Peer Review

119087
119087
119087
119087
119087
119087
119087
119087
119087
119087
119087
119087
119087
119087
119087
119087
119087
119087
119087
119087
119087
119087
119087
119087
119087
119087
119087
119087
119087
119087
119087
119087
119087
119087
119087
119087
119087

For Peer Review

119087
119087
119087
119087
119087
119087
119087
119087
119087
119087

119087
119087
119087
119087
119087
119087
119087

For Peer Review

Table S1f. Sample Information of GSE136103

!Sample_t	!Sample_g	!Sample_s	!Sample_s	!Sample_l	!Sample_t	!Sample_c	!Sample_s	!Sample_o
Healthy1_	GSM404115	Public on	Aug 21 20	Sep 13 20	SRA	1	Liver	Homo sapi
Healthy1_	GSM404115	Public on	Aug 21 20	Sep 13 20	SRA	1	Liver	Homo sapi
Healthy1_	GSM404115	Public on	Aug 21 20	Sep 13 20	SRA	1	Liver	Homo sapi
Healthy2_	GSM404115	Public on	Aug 21 20	Sep 13 20	SRA	1	Liver	Homo sapi
Healthy2_	GSM404115	Public on	Aug 21 20	Sep 13 20	SRA	1	Liver	Homo sapi
Healthy3_	GSM404115	Public on	Aug 21 20	Sep 13 20	SRA	1	Liver	Homo sapi
Healthy3_	GSM404115	Public on	Aug 21 20	Sep 13 20	SRA	1	Liver	Homo sapi
Healthy3_	GSM404115	Public on	Aug 21 20	Sep 13 20	SRA	1	Liver	Homo sapi
Healthy4_	GSM404115	Public on	Aug 21 20	Sep 13 20	SRA	1	Liver	Homo sapi
Healthy4_	GSM404115	Public on	Aug 21 20	Sep 13 20	SRA	1	Liver	Homo sapi
Healthy5_	GSM404116	Public on	Aug 21 20	Sep 13 20	SRA	1	Liver	Homo sapi
Cirrhotic	GSM404116	Public on	Aug 21 20	Sep 13 20	SRA	1	Liver	Homo sapi
Cirrhotic	GSM404116	Public on	Aug 21 20	Sep 13 20	SRA	1	Liver	Homo sapi
Cirrhotic	GSM404116	Public on	Aug 21 20	Sep 13 20	SRA	1	Liver	Homo sapi
Cirrhotic	GSM404116	Public on	Aug 21 20	Sep 13 20	SRA	1	Liver	Homo sapi
Cirrhotic	GSM404116	Public on	Aug 21 20	Sep 13 20	SRA	1	Liver	Homo sapi
Cirrhotic	GSM404116	Public on	Aug 21 20	Sep 13 20	SRA	1	Liver	Homo sapi
Cirrhotic	GSM404116	Public on	Aug 21 20	Sep 13 20	SRA	1	Liver	Homo sapi
Cirrhotic	GSM404116	Public on	Aug 21 20	Sep 13 20	SRA	1	Liver	Homo sapi
Cirrhotic	GSM404116	Public on	Aug 21 20	Sep 13 20	SRA	1	Liver	Homo sapi
Blood1	GSM404117	Public on	Aug 21 20	Sep 13 20	SRA	1	PBMC	Homo sapi
Blood2	GSM404117	Public on	Aug 21 20	Sep 13 20	SRA	1	PBMC	Homo sapi
Blood3	GSM404117	Public on	Aug 21 20	Sep 13 20	SRA	1	PBMC	Homo sapi
Blood4	GSM404117	Public on	Aug 21 20	Sep 13 20	SRA	1	PBMC	Homo sapi

For Peer Review

!Sample_c	!Sample_c	!Sample_c	!Sample_c	!Sample_c	!Sample_m	!Sample_e	!Sample_e	!Sample_t
disease s	populatio	cause of	Sex: Male	cell subt	total RNA	Enzymatic	10X Genom	9606
disease s	populatio	cause of	Sex: Male	cell subt	total RNA	Enzymatic	10X Genom	9606
disease s	populatio	cause of	Sex: Male	cell subt	total RNA	Enzymatic	10X Genom	9606
disease s	populatio	cause of	Sex: Male	cell subt	total RNA	Enzymatic	10X Genom	9606
disease s	populatio	cause of	Sex: Male	cell subt	total RNA	Enzymatic	10X Genom	9606
disease s	populatio	cause of	Sex: Male	cell subt	total RNA	Enzymatic	10X Genom	9606
disease s	populatio	cause of	Sex: Male	cell subt	total RNA	Enzymatic	10X Genom	9606
disease s	populatio	cause of	Sex: Male	cell subt	total RNA	Enzymatic	10X Genom	9606
disease s	populatio	cause of	Sex: Fema	cell subt	total RNA	Enzymatic	10X Genom	9606
disease s	populatio	cause of	Sex: Fema	cell subt	total RNA	Enzymatic	10X Genom	9606
disease s	populatio	cause of	Sex: Male	cell subt	total RNA	Enzymatic	10X Genom	9606
disease s	populatio	cause of	Sex: Fema	cell subt	total RNA	Enzymatic	10X Genom	9606
disease s	populatio	cause of	Sex: Fema	cell subt	total RNA	Enzymatic	10X Genom	9606
disease s	populatio	cause of	Sex: Fema	cell subt	total RNA	Enzymatic	10X Genom	9606
disease s	populatio	cause of	Sex: Male	cell subt	total RNA	Enzymatic	10X Genom	9606
disease s	populatio	cause of	Sex: Male	cell subt	total RNA	Enzymatic	10X Genom	9606
disease s	populatio	cause of	Sex: Male	cell subt	total RNA	Enzymatic	10X Genom	9606
disease s	populatio	cause of	Sex: Male	cell subt	total RNA	Enzymatic	10X Genom	9606
disease s	populatio	cause of	Sex: Male	cell subt	total RNA	Enzymatic	10X Genom	9606
disease s	populatio	cause of	Sex: Fema	cell subt	total RNA	Enzymatic	10X Genom	9606
disease s	populatio	cause of	Sex: Male		total RNA	FACS	10X Genom	9606
disease s	populatio	cause of	Sex: Male		total RNA	FACS	10X Genom	9606
disease s	populatio	cause of	Sex: Female		total RNA	FACS	10X Genom	9606
disease s	populatio	cause of	Sex: Male		total RNA	FACS	10X Genom	9606

For Peer Review

!Sample_d	!Sample_d	!Sample_d	!Sample_d	!Sample_p	!Sample_c	!Sample_c	!Sample_c	!Sample_c
10X Genom	Demultipl	Genome_bu	Supplemen	GPL20301	John, Roge	Henderson	Centre fo	Universit
10X Genom	Demultipl	Genome_bu	Supplemen	GPL20301	John, Roge	Henderson	Centre fo	Universit
10X Genom	Demultipl	Genome_bu	Supplemen	GPL20301	John, Roge	Henderson	Centre fo	Universit
10X Genom	Demultipl	Genome_bu	Supplemen	GPL20301	John, Roge	Henderson	Centre fo	Universit
10X Genom	Demultipl	Genome_bu	Supplemen	GPL20301	John, Roge	Henderson	Centre fo	Universit
10X Genom	Demultipl	Genome_bu	Supplemen	GPL20301	John, Roge	Henderson	Centre fo	Universit
10X Genom	Demultipl	Genome_bu	Supplemen	GPL20301	John, Roge	Henderson	Centre fo	Universit
10X Genom	Demultipl	Genome_bu	Supplemen	GPL20301	John, Roge	Henderson	Centre fo	Universit
10X Genom	Demultipl	Genome_bu	Supplemen	GPL20301	John, Roge	Henderson	Centre fo	Universit
10X Genom	Demultipl	Genome_bu	Supplemen	GPL20301	John, Roge	Henderson	Centre fo	Universit
10X Genom	Demultipl	Genome_bu	Supplemen	GPL20301	John, Roge	Henderson	Centre fo	Universit
10X Genom	Demultipl	Genome_bu	Supplemen	GPL20301	John, Roge	Henderson	Centre fo	Universit
10X Genom	Demultipl	Genome_bu	Supplemen	GPL20301	John, Roge	Henderson	Centre fo	Universit
10X Genom	Demultipl	Genome_bu	Supplemen	GPL20301	John, Roge	Henderson	Centre fo	Universit
10X Genom	Demultipl	Genome_bu	Supplemen	GPL20301	John, Roge	Henderson	Centre fo	Universit
10X Genom	Demultipl	Genome_bu	Supplemen	GPL20301	John, Roge	Henderson	Centre fo	Universit
10X Genom	Demultipl	Genome_bu	Supplemen	GPL20301	John, Roge	Henderson	Centre fo	Universit
10X Genom	Demultipl	Genome_bu	Supplemen	GPL20301	John, Roge	Henderson	Centre fo	Universit
10X Genom	Demultipl	Genome_bu	Supplemen	GPL20301	John, Roge	Henderson	Centre fo	Universit
10X Genom	Demultipl	Genome_bu	Supplemen	GPL20301	John, Roge	Henderson	Centre fo	Universit
10X Genom	Demultipl	Genome_bu	Supplemen	GPL20301	John, Roge	Henderson	Centre fo	Universit
10X Genom	Demultipl	Genome_bu	Supplemen	GPL20301	John, Roge	Henderson	Centre fo	Universit
10X Genom	Demultipl	Genome_bu	Supplemen	GPL20301	John, Roge	Henderson	Centre fo	Universit
10X Genom	Demultipl	Genome_bu	Supplemen	GPL20301	John, Roge	Henderson	Centre fo	Universit
10X Genom	Demultipl	Genome_bu	Supplemen	GPL20301	John, Roge	Henderson	Centre fo	Universit

For Peer Review

!Sample_c	!Sample_c	!Sample_c	!Sample_c	!Sample_d	!Sample_i	!Sample_l	!Sample_l	!Sample_l
47 Little	Edinburgh	EH16 4TJ	United Ki	0	Illumina	cDNA	transcrip	RNA-Seq
47 Little	Edinburgh	EH16 4TJ	United Ki	0	Illumina	cDNA	transcrip	RNA-Seq
47 Little	Edinburgh	EH16 4TJ	United Ki	0	Illumina	cDNA	transcrip	RNA-Seq
47 Little	Edinburgh	EH16 4TJ	United Ki	0	Illumina	cDNA	transcrip	RNA-Seq
47 Little	Edinburgh	EH16 4TJ	United Ki	0	Illumina	cDNA	transcrip	RNA-Seq
47 Little	Edinburgh	EH16 4TJ	United Ki	0	Illumina	cDNA	transcrip	RNA-Seq
47 Little	Edinburgh	EH16 4TJ	United Ki	0	Illumina	cDNA	transcrip	RNA-Seq
47 Little	Edinburgh	EH16 4TJ	United Ki	0	Illumina	cDNA	transcrip	RNA-Seq
47 Little	Edinburgh	EH16 4TJ	United Ki	0	Illumina	cDNA	transcrip	RNA-Seq
47 Little	Edinburgh	EH16 4TJ	United Ki	0	Illumina	cDNA	transcrip	RNA-Seq
47 Little	Edinburgh	EH16 4TJ	United Ki	0	Illumina	cDNA	transcrip	RNA-Seq
47 Little	Edinburgh	EH16 4TJ	United Ki	0	Illumina	cDNA	transcrip	RNA-Seq
47 Little	Edinburgh	EH16 4TJ	United Ki	0	Illumina	cDNA	transcrip	RNA-Seq
47 Little	Edinburgh	EH16 4TJ	United Ki	0	Illumina	cDNA	transcrip	RNA-Seq
47 Little	Edinburgh	EH16 4TJ	United Ki	0	Illumina	cDNA	transcrip	RNA-Seq
47 Little	Edinburgh	EH16 4TJ	United Ki	0	Illumina	cDNA	transcrip	RNA-Seq
47 Little	Edinburgh	EH16 4TJ	United Ki	0	Illumina	cDNA	transcrip	RNA-Seq
47 Little	Edinburgh	EH16 4TJ	United Ki	0	Illumina	cDNA	transcrip	RNA-Seq
47 Little	Edinburgh	EH16 4TJ	United Ki	0	Illumina	cDNA	transcrip	RNA-Seq
47 Little	Edinburgh	EH16 4TJ	United Ki	0	Illumina	cDNA	transcrip	RNA-Seq
47 Little	Edinburgh	EH16 4TJ	United Ki	0	Illumina	cDNA	transcrip	RNA-Seq
47 Little	Edinburgh	EH16 4TJ	United Ki	0	Illumina	cDNA	transcrip	RNA-Seq
47 Little	Edinburgh	EH16 4TJ	United Ki	0	Illumina	cDNA	transcrip	RNA-Seq
47 Little	Edinburgh	EH16 4TJ	United Ki	0	Illumina	cDNA	transcrip	RNA-Seq
47 Little	Edinburgh	EH16 4TJ	United Ki	0	Illumina	cDNA	transcrip	RNA-Seq

For Peer Review

!Sample_r	!Sample_r	!Sample_s	!Sample_s	!Sample_supplementary_file_3
BioSample	SRA: http	ftp://ftp	ftp://ftp	ftp://ftp.ncbi.nlm.nih.gov/geo/samples/GSM4041n
BioSample	SRA: http	ftp://ftp	ftp://ftp	ftp://ftp.ncbi.nlm.nih.gov/geo/samples/GSM4041n
BioSample	SRA: http	ftp://ftp	ftp://ftp	ftp://ftp.ncbi.nlm.nih.gov/geo/samples/GSM4041n
BioSample	SRA: http	ftp://ftp	ftp://ftp	ftp://ftp.ncbi.nlm.nih.gov/geo/samples/GSM4041n
BioSample	SRA: http	ftp://ftp	ftp://ftp	ftp://ftp.ncbi.nlm.nih.gov/geo/samples/GSM4041n
BioSample	SRA: http	ftp://ftp	ftp://ftp	ftp://ftp.ncbi.nlm.nih.gov/geo/samples/GSM4041n
BioSample	SRA: http	ftp://ftp	ftp://ftp	ftp://ftp.ncbi.nlm.nih.gov/geo/samples/GSM4041n
BioSample	SRA: http	ftp://ftp	ftp://ftp	ftp://ftp.ncbi.nlm.nih.gov/geo/samples/GSM4041n
BioSample	SRA: http	ftp://ftp	ftp://ftp	ftp://ftp.ncbi.nlm.nih.gov/geo/samples/GSM4041n
BioSample	SRA: http	ftp://ftp	ftp://ftp	ftp://ftp.ncbi.nlm.nih.gov/geo/samples/GSM4041n
BioSample	SRA: http	ftp://ftp	ftp://ftp	ftp://ftp.ncbi.nlm.nih.gov/geo/samples/GSM4041n
BioSample	SRA: http	ftp://ftp	ftp://ftp	ftp://ftp.ncbi.nlm.nih.gov/geo/samples/GSM4041n
BioSample	SRA: http	ftp://ftp	ftp://ftp	ftp://ftp.ncbi.nlm.nih.gov/geo/samples/GSM4041n
BioSample	SRA: http	ftp://ftp	ftp://ftp	ftp://ftp.ncbi.nlm.nih.gov/geo/samples/GSM4041n
BioSample	SRA: http	ftp://ftp	ftp://ftp	ftp://ftp.ncbi.nlm.nih.gov/geo/samples/GSM4041n
BioSample	SRA: http	ftp://ftp	ftp://ftp	ftp://ftp.ncbi.nlm.nih.gov/geo/samples/GSM4041n
BioSample	SRA: http	ftp://ftp	ftp://ftp	ftp://ftp.ncbi.nlm.nih.gov/geo/samples/GSM4041n
BioSample	SRA: http	ftp://ftp	ftp://ftp	ftp://ftp.ncbi.nlm.nih.gov/geo/samples/GSM4041n
BioSample	SRA: http	ftp://ftp	ftp://ftp	ftp://ftp.ncbi.nlm.nih.gov/geo/samples/GSM4041n
BioSample	SRA: http	ftp://ftp	ftp://ftp	ftp://ftp.ncbi.nlm.nih.gov/geo/samples/GSM4041n
BioSample	SRA: http	ftp://ftp	ftp://ftp	ftp://ftp.ncbi.nlm.nih.gov/geo/samples/GSM4041n
BioSample	SRA: http	ftp://ftp	ftp://ftp	ftp://ftp.ncbi.nlm.nih.gov/geo/samples/GSM4041n
BioSample	SRA: http	ftp://ftp	ftp://ftp	ftp://ftp.ncbi.nlm.nih.gov/geo/samples/GSM4041n
BioSample	SRA: http	ftp://ftp	ftp://ftp	ftp://ftp.ncbi.nlm.nih.gov/geo/samples/GSM4041n
BioSample	SRA: http	ftp://ftp	ftp://ftp	ftp://ftp.ncbi.nlm.nih.gov/geo/samples/GSM4041n

For Peer Review

ınn/GSM4041150/suppl/GSM4041150_healthy1_cd45+_matrix.mtx.gz
ınn/GSM4041151/suppl/GSM4041151_healthy1_cd45-A_matrix.mtx.gz
ınn/GSM4041152/suppl/GSM4041152_healthy1_cd45-B_matrix.mtx.gz
ınn/GSM4041153/suppl/GSM4041153_healthy2_cd45+_matrix.mtx.gz
ınn/GSM4041154/suppl/GSM4041154_healthy2_cd45-_matrix.mtx.gz
ınn/GSM4041155/suppl/GSM4041155_healthy3_cd45+_matrix.mtx.gz
ınn/GSM4041156/suppl/GSM4041156_healthy3_cd45-A_matrix.mtx.gz
ınn/GSM4041157/suppl/GSM4041157_healthy3_cd45-B_matrix.mtx.gz
ınn/GSM4041158/suppl/GSM4041158_healthy4_cd45+_matrix.mtx.gz
ınn/GSM4041159/suppl/GSM4041159_healthy4_cd45-_matrix.mtx.gz
ınn/GSM4041160/suppl/GSM4041160_healthy5_cd45+_matrix.mtx.gz
ınn/GSM4041161/suppl/GSM4041161_cirrhotic1_cd45+_matrix.mtx.gz
ınn/GSM4041162/suppl/GSM4041162_cirrhotic1_cd45-A_matrix.mtx.gz
ınn/GSM4041163/suppl/GSM4041163_cirrhotic1_cd45-B_matrix.mtx.gz
ınn/GSM4041164/suppl/GSM4041164_cirrhotic2_cd45+_matrix.mtx.gz
ınn/GSM4041165/suppl/GSM4041165_cirrhotic2_cd45-_matrix.mtx.gz
ınn/GSM4041166/suppl/GSM4041166_cirrhotic3_cd45+_matrix.mtx.gz
ınn/GSM4041167/suppl/GSM4041167_cirrhotic3_cd45-_matrix.mtx.gz
ınn/GSM4041168/suppl/GSM4041168_cirrhotic4_cd45+_matrix.mtx.gz
ınn/GSM4041169/suppl/GSM4041169_cirrhotic5_cd45+_matrix.mtx.gz
ınn/GSM4041170/suppl/GSM4041170_blood1_matrix.mtx.gz
ınn/GSM4041171/suppl/GSM4041171_blood2_matrix.mtx.gz
ınn/GSM4041172/suppl/GSM4041172_blood3_matrix.mtx.gz
ınn/GSM4041173/suppl/GSM4041173_blood4_matrix.mtx.gz

For Peer Review

Table S2. Differential gene expression analysis across cell clusters

Legend:

DGE Analysis: We conducted differential gene expression (DGE) analysis at the single-cell level and selected genes upregulated compared to other clusters as characteristic genes for each cluster.

Upregulated Genes: Cell types that did not show upregulated genes ($log_2FC > 1$, p-value < 0.05) were filtered out.

Table Structure:

- Sheets "top_genes": Top 10 upregulated genes across each cell cluster for each disease.

For Peer Review

Table S2a. TOP 10 genes list based on single-cell data of liver cirrhosis patients and healthy inc

	p_val	avg_log2F	pct.1	pct.2	p_val_adj	cluster	gene
1	0	2.21671	0.661	0.102	0	0	IL7R
2	0	2.1902	0.83	0.353	0	0	LTB
3	0	1.95296	0.857	0.285	0	0	TRAC
4	0	1.70035	0.826	0.235	0	0	CD3D
5	0	1.49917	0.762	0.237	0	0	CD3E
6	0	1.43069	0.874	0.543	0	0	CXCR4
7	0	1.41283	0.763	0.277	0	0	CD2
8	0	1.40608	0.728	0.327	0	0	TRBC2
9	0	1.358	0.959	0.593	0	0	IL32
10	0	1.34881	0.903	0.508	0	0	CD52
11	0	3.5016	0.494	0.219	0	1	GNLY
12	0	3.01886	0.898	0.468	0	1	CCL3
13	0	2.99747	0.725	0.165	0	1	XCL1
14	0	2.9883	0.987	0.468	0	1	NKG7
15	0	2.74	0.625	0.121	0	1	XCL2
16	0	2.65845	0.804	0.289	0	1	CMC1
17	0	2.64918	0.74	0.076	0	1	KLRF1
18	0	2.55662	0.849	0.155	0	1	KLRD1
19	0	2.52947	0.989	0.654	0	1	CCL4
20	0	2.50643	0.889	0.32	0	1	CD7
21	0	1.54157	0.9	0.507	0	2	CCL5
22	0	1.46464	0.463	0.123	0	2	CD8A
23	0	1.38541	0.431	0.148	0	2	GZMH
24	0	1.37611	0.766	0.324	0	2	CD3D
25	0	1.34481	0.379	0.087	0	2	CD8B
26	0	1.33738	0.616	0.279	0	2	GZMK
27	0	1.33413	0.79	0.372	0	2	TRAC
28	0	1.22333	0.73	0.313	0	2	CD3E
29	0	1.21586	0.933	0.647	0	2	IL32
30	0	1.19713	0.708	0.385	0	2	TRBC2
31	0	3.34794	0.867	0.149	0	3	MGP
32	0	3.2712	0.844	0.098	0	3	SPARCL1
33	0	3.0815	0.818	0.056	0	3	VWF
34	0	3.01696	0.982	0.158	0	3	GNG11
35	0	3.01522	0.429	0.079	0	3	FABP4
36	0	2.9994	0.795	0.059	0	3	PLVAP
37	0	2.93102	0.99	0.273	0	3	IGFBP7
38	0	2.83533	0.382	0.019	0	3	ACKR1
39	0	2.77323	0.932	0.171	0	3	ID1
40	0	2.74181	0.931	0.155	0	3	IGFBP4
41	0	5.52333	0.85	0.169	0	4	S100A8
42	0	5.4828	0.93	0.203	0	4	S100A9
43	0	4.06523	0.971	0.245	0	4	LYZ
44	0	3.66789	0.844	0.112	0	4	RP11-1143G9.4
45	0	3.63229	0.916	0.071	0	4	FCN1
46	0	3.25303	0.502	0.019	0	4	S100A12
47	0	3.17843	0.641	0.041	0	4	VCAN
48	0	2.8145	0.952	0.256	0	4	LST1
49	0	2.75593	0.944	0.236	0	4	AIF1
50	0	2.74958	0.822	0.075	0	4	CSTA
51	0	3.10144	0.879	0.127	0	5	C1QC
52	0	2.94936	0.926	0.219	0	5	C1QA
53	0	2.89563	0.895	0.202	0	5	C1QB
54	0	2.82602	0.991	0.58	0	5	HLA-DRA
55	0	2.79583	0.972	0.504	0	5	HLA-DPA1
56	0	2.68102	0.977	0.523	0	5	HLA-DRB1
57	0	2.63173	0.977	0.531	0	5	HLA-DPB1
58	0	2.58217	0.95	0.348	0	5	HLA-DRB5

For Peer Review

59	0	2.5546	1	0.981	0	5	FTL
60	0	2.47661	0.901	0.246	0	5	HLA-DQA1
61	0	3.84926	0.942	0.036	0	6	CD79A
62	0	2.99945	0.825	0.02	0	6	MS4A1
63	0	2.74931	0.847	0.109	0	6	CD79B
64	0	2.72033	0.425	0.01	0	6	TCL1A
65	0	2.28329	0.961	0.488	0	6	CD37
66	0	2.19526	0.557	0.006	0	6	VPREB3
67	0	1.98542	0.487	0.027	0	6	IGHD
68	0	1.86963	0.999	0.849	0	6	CD74
69	0	1.84619	0.525	0.007	0	6	LINC00926
70	0	1.76732	0.545	0.013	0	6	BANK1
71	0	4.80297	0.994	0.205	0	7	C1QB
72	0	4.68173	1	0.224	0	7	C1QA
73	0	4.44052	0.988	0.131	0	7	C1QC
74	0	4.38174	0.912	0.046	0	7	CD5L
75	0	4.08985	0.832	0.06	0	7	MARCO
76	0	3.94876	0.958	0.21	0	7	APOE
77	0	3.82007	0.998	0.266	0	7	SEPP1
78	0	3.77484	0.97	0.138	0	7	SLC40A1
79	0	3.74225	0.977	0.065	0	7	CD163
80	0	3.72653	0.981	0.134	0	7	MS4A7
81	0	5.79263	0.981	0.074	0	8	TAGLN
82	0	5.70577	0.976	0.074	0	8	ACTA2
83	0	5.18317	0.983	0.093	0	8	MYL9
84	0	5.04778	0.798	0.023	0	8	RGS5
85	0	4.78951	0.952	0.15	0	8	ADIRF
86	0	4.67234	0.961	0.054	0	8	TPM2
87	0	3.84925	0.967	0.162	0	8	CALD1
88	0	3.79615	0.839	0.021	0	8	MYH11
89	0	3.68814	0.788	0.013	0	8	NDUFA4L2
90	0	3.59148	0.885	0.057	0	8	SOD3
91	0	3.32534	0.994	0.536	0	9	HLA-DPB1
92	0	3.2922	0.982	0.251	0	9	HLA-DQA1
93	0	3.17458	0.984	0.314	0	9	HLA-DQB1
94	0	3.15859	0.993	0.509	0	9	HLA-DPA1
95	0	3.0543	0.998	0.585	0	9	HLA-DRA
96	0	2.86673	0.994	0.528	0	9	HLA-DRB1
97	0	2.83317	0.634	0.03	0	9	FCER1A
98	0	2.79889	0.999	0.85	0	9	CD74
99	0	2.74548	0.995	0.575	0	9	CST3
100	0	2.67056	0.98	0.355	0	9	HLA-DRB5
101	0	4.02993	0.998	0.068	0	10	TM4SF4
102	0	4.01001	0.999	0.181	0	10	ANXA4
103	0	3.99135	0.998	0.095	0	10	DEFB1
104	0	3.75642	0.996	0.059	0	10	FXYD2
105	0	3.73343	0.984	0.094	0	10	KRT8
106	0	3.71056	0.989	0.306	0	10	ALB
107	0	3.67886	0.987	0.103	0	10	KRT18
108	0	3.66768	0.952	0.101	0	10	AMBP
109	0	3.65648	0.992	0.283	0	10	SERPINA1
110	0	3.57158	0.974	0.059	0	10	KRT7
111	0	5.85512	0.927	0.101	0	11	FCN3
112	0	4.8876	0.96	0.066	0	11	CRHBP
113	0	4.50813	0.824	0.027	0	11	CLEC4G
114	0	4.47659	0.991	0.163	0	11	DNASE1L3
115	0	4.11408	0.815	0.022	0	11	FCN2
116	0	3.80978	0.78	0.016	0	11	CLEC1B
117	0	3.80159	0.93	0.194	0	11	CTSL
118	0	3.49579	0.721	0.015	0	11	CLEC4M

For Peer Review

119	0	3.28292	0.833	0.045	0	11	AKAP12
120	0	3.23457	0.891	0.088	0	11	PLPP3
121	0	4.04995	0.968	0.099	0	12	DEFB1
122	0	3.70453	0.953	0.063	0	12	FXYD2
123	0	3.41929	0.969	0.185	0	12	ANXA4
124	0	3.37182	0.959	0.072	0	12	TM4SF4
125	0	3.02683	0.901	0.108	0	12	KRT18
126	0	3.02332	0.873	0.1	0	12	KRT8
127	0	2.97152	0.948	0.309	0	12	ALB
128	0	2.86224	0.937	0.287	0	12	SERPINA1
129	0	2.84239	0.832	0.066	0	12	KRT7
130	0	2.83671	0.954	0.198	0	12	CLU
131	0	4.19305	0.964	0.146	0	13	SLC9A3R2
132	0	3.72443	0.879	0.107	0	13	CLDN5
133	0	3.40573	0.995	0.233	0	13	IFI27
134	0	3.31234	0.984	0.143	0	13	RAMP2
135	0	3.19133	0.978	0.215	0	13	ID1
136	0	3.16843	0.966	0.21	0	13	TM4SF1
137	0	3.1575	0.865	0.124	0	13	TIMP3
138	0	3.08265	0.905	0.13	0	13	PLPP1
139	0	3.02162	0.805	0.09	0	13	CXCL12
140	0	2.89107	0.762	0.121	0	13	RBP7
141	0	7.87783	0.917	0.35	0	14	IGLC3
142	0	7.76751	0.932	0.428	0	14	IGLC2
143	0	7.47827	0.988	0.749	0	14	IGKC
144	0	7.40154	0.975	0.508	0	14	IGHA1
145	0	7.3383	0.787	0.155	0	14	IGHG1
146	0	7.26564	0.773	0.162	0	14	IGHG3
147	0	6.93848	0.951	0.222	0	14	JCHAIN
148	0	6.71858	0.524	0.043	0	14	IGHA2
149	0	6.71416	0.792	0.215	0	14	IGHM
150	0	6.54672	0.593	0.043	0	14	IGHGP
151	0	3.52859	0.969	0.262	0	15	STMN1
152	0	3.16317	0.96	0.383	0	15	HMGB2
153	#######	2.98353	0.732	0.35	#######	15	HIST1H4C
154	0	2.66125	0.977	0.671	0	15	TUBA1B
155	0	2.56231	0.946	0.571	0	15	TUBB
156	0	2.3666	0.702	0.013	0	15	TYMS
157	0	2.32313	0.766	0.035	0	15	KIAA0101
158	0	2.25855	0.546	0.008	0	15	UBE2C
159	0	2.22567	0.964	0.594	0	15	HMGN2
160	0	2.11674	0.972	0.625	0	15	H2AFZ
161	0	5.22724	0.93	0.028	0	16	DCN
162	0	4.76316	0.843	0.017	0	16	COL1A1
163	0	4.4064	0.891	0.018	0	16	COL3A1
164	#######	4.40576	0.965	0.465	#######	16	TIMP1
165	0	4.35154	0.937	0.07	0	16	BGN
166	0	4.25015	0.871	0.017	0	16	COL1A2
167	0	4.15817	0.675	0.006	0	16	LUM
168	0	3.77454	0.552	0.037	0	16	PTGDS
169	0	3.64193	0.93	0.048	0	16	C1R
170	0	3.60897	0.742	0.035	0	16	CCDC80
171	0	6.69082	0.973	0.175	0	17	APOA1
172	#######	6.45906	0.829	0.174	#######	17	APOA2
173	0	6.03884	0.866	0.132	0	17	APOC3
174	0	5.87393	0.991	0.211	0	17	APOC1
175	#######	5.87191	0.994	0.323	#######	17	ALB
176	0	5.86279	0.741	0.075	0	17	HP
177	0	5.2872	0.902	0.036	0	17	ORM1
178	0	5.21827	0.942	0.094	0	17	TTR

For Peer Review

179	0	5.07226	0.991	0.098	0	17	RBP4
180	0	5.0046	0.838	0.046	0	17	FGB
181	0	3.61688	0.978	0.159	0	18	GZMB
182	#######	3.54589	0.333	0.039	#######	18	PTGDS
183	0	3.36103	0.951	0.146	0	18	IRF7
184	0	3.30678	0.944	0.045	0	18	PLD4
185	0	3.23118	0.901	0.009	0	18	LILRA4
186	0	3.07328	0.929	0.131	0	18	ITM2C
187	0	2.92179	0.932	0.158	0	18	IRF8
188	0	2.71991	0.91	0.162	0	18	CCDC50
189	0	2.67553	0.41	0.021	0	18	TCL1A
190	0	2.62587	0.873	0.104	0	18	PPP1R14B
191	0	7.84951	0.473	0.017	0	19	TPSB2
192	0	7.82415	0.467	0.011	0	19	TPSAB1
193	0	4.02634	0.302	0.003	0	19	CTSG
194	0	3.46812	0.429	0.001	0	19	CPA3
195	0	3.01858	0.412	0.01	0	19	HPGDS
196	1.56E-25	2.71449	0.418	0.166	3.59E-21	19	AREG
197	0	2.70027	0.258	0.001	0	19	TPSD1
198	#######	2.52866	0.346	0.017	#######	19	HPGD
199	0	2.47662	0.368	0.002	0	19	MS4A2
200	#######	2.29209	0.352	0.026	#######	19	C1orf186
201	8.21E-96	3.08523	1	0.674	1.89E-91	20	TUBA1B
202	#######	2.89215	1	0.27	#######	20	STMN1
203	#######	2.64888	0.986	0.389	#######	20	HMGB2
204	4.57E-78	2.50328	1	0.586	1.05E-73	20	CST3
205	7.91E-60	2.39425	0.885	0.354	1.82E-55	20	HIST1H4C
206	0	2.38892	0.892	0.042	0	20	KIAA0101
207	4.59E-88	2.32069	1	0.575	1.06E-83	20	TUBB
208	2.06E-86	2.31707	1	0.598	4.74E-82	20	HMGN2
209	2.35E-73	2.25633	0.98	0.522	5.41E-69	20	HLA-DPA1
210	2.11E-70	2.20146	0.993	0.596	4.87E-66	20	HLA-DRA
211	2.82E-65	2.22554	0.89	0.248	6.49E-61	21	C1QA
212	1.04E-61	2.14257	0.853	0.229	2.40E-57	21	C1QB
213	2.44E-65	2.02683	0.734	0.157	5.61E-61	21	C1QC
214	6.76E-46	1.94083	1	0.981	1.56E-41	21	FTL
215	6.08E-49	1.79011	0.67	0.163	1.40E-44	21	SLC40A1
216	1.80E-66	1.71535	0.89	0.221	4.15E-62	21	MS4A6A
217	2.69E-69	1.69924	0.78	0.16	6.20E-65	21	MS4A7
218	2.41E-34	1.69086	0.706	0.233	5.56E-30	21	APOE
219	3.90E-46	1.66247	0.936	0.367	8.98E-42	21	CTSB
220	1.25E-42	1.6045	0.991	0.541	2.87E-38	21	HLA-DRB1

For Peer Review

dividuals

For Peer Review

Table S2b. TOP10 genes list based on single-cell data of liver cirrhosis patients

	p_val	avg_log2F	pct.1	pct.2	p_val_adj	cluster	gene
1	0	2.30128	0.764	0.123	0	0	IL7R
2	0	2.11263	0.896	0.366	0	0	LTB
3	0	1.63047	0.951	0.339	0	0	TRAC
4	0	1.61562	0.987	0.586	0	0	CXCR4
5	0	1.49666	0.922	0.476	0	0	CD69
6	0	1.41684	0.785	0.237	0	0	CD3E
7	0	1.39591	0.879	0.29	0	0	CD3D
8	0	1.36315	0.962	0.804	0	0	ZFP36L2
9	0	1.29912	0.874	0.617	0	0	TSC22D3
10	0	1.28274	0.968	0.542	0	0	CD52
11	0	2.02682	0.901	0.438	0	1	CCL5
12	0	2.00664	0.576	0.096	0	1	CD8A
13	#######	1.92931	0.561	0.161	#######	1	IFNG
14	0	1.75357	0.943	0.404	0	1	TRAC
15	0	1.69973	0.905	0.347	0	1	CD3D
16	#######	1.69124	0.731	0.332	#######	1	RGS1
17	0	1.67284	0.907	0.354	0	1	CD2
18	#######	1.64401	0.406	0.092	#######	1	GZMH
19	0	1.63305	0.969	0.689	0	1	IL32
20	0	1.57881	0.822	0.374	0	1	TRBC2
21	0	4.63043	0.512	0.101	0	2	GNLY
22	0	3.77178	0.942	0.437	0	2	CCL3
23	0	3.65724	1	0.383	0	2	NKG7
24	0	3.05359	0.873	0.271	0	2	CMC1
25	0	3.03985	0.5	0.072	0	2	GZMB
26	0	2.9859	0.992	0.632	0	2	CCL4
27	0	2.92943	0.848	0.03	0	2	KLRF1
28	0	2.85082	0.916	0.115	0	2	KLRD1
29	0	2.83624	0.354	0.02	0	2	FGFBP2
30	0	2.80813	0.769	0.155	0	2	XCL2
31	0	3.68494	0.999	0.211	0	3	ANXA4
32	0	3.66829	1	0.119	0	3	TM4SF4
33	0	3.51553	0.999	0.134	0	3	KRT8
34	0	3.48929	0.981	0.109	0	3	SPP1
35	0	3.32639	0.997	0.148	0	3	KRT18
36	0	3.32194	1	0.167	0	3	DEFB1
37	0	3.31277	0.995	0.093	0	3	FXYD2
38	0	3.21658	0.974	0.166	0	3	AMBP
39	0	3.21235	0.978	0.1	0	3	KRT7
40	0	3.1835	0.981	0.082	0	3	GC
41	0	4.40847	0.916	0.075	0	4	DNASE1L3
42	#######	4.29683	0.33	0.07	#######	4	CCL21
43	0	3.73204	0.518	0.076	0	4	FABP4
44	0	3.55362	0.987	0.13	0	4	GNG11
45	0	3.47275	0.791	0.013	0	4	CCL14
46	0	3.31636	0.829	0.074	0	4	SPARCL1
47	0	3.31108	0.826	0.123	0	4	MGP
48	0	3.25538	0.802	0.043	0	4	VWF
49	0	3.0544	0.948	0.195	0	4	ID1
50	#######	2.87262	0.397	0.055	#######	4	TFF3
51	0	5.72152	0.968	0.159	0	5	S100A9
52	0	5.57403	0.896	0.14	0	5	S100A8
53	0	3.85789	0.984	0.064	0	5	FCN1
54	0	3.63389	0.977	0.23	0	5	LYZ
55	0	3.27772	0.995	0.189	0	5	AIF1
56	0	3.17234	0.988	0.182	0	5	LST1
57	0	2.78583	0.825	0.095	0	5	RP11-1143G9.4
58	0	2.76223	0.94	0.073	0	5	CSTA

For Peer Review

59	0	2.67133	0.484	0.009	0	5	S100A12
60	0	2.62247	0.979	0.337	0	5	CTSS
61	0	3.45755	0.93	0.04	0	6	CD79A
62	0	2.98423	0.845	0.029	0	6	MS4A1
63	0	2.45833	0.777	0.119	0	6	CD79B
64	0	1.987	0.489	0.008	0	6	VPREB3
65	#######	1.94345	0.955	0.509	#######	6	CD37
66	#######	1.92997	1	0.821	#######	6	CD74
67	#######	1.88394	0.617	0.165	#######	6	CD83
68	0	1.85496	0.559	0.01	0	6	BANK1
69	#######	1.69785	0.32	0.019	#######	6	IGHD
70	#######	1.67745	0.998	0.544	#######	6	HLA-DRA
71	0	3.00416	0.986	0.212	0	7	DEFB1
72	0	2.95136	0.861	0.129	0	7	ELF3
73	0	2.74679	0.947	0.143	0	7	FXYD2
74	#######	2.60199	0.986	0.615	#######	7	MTRNR2L8
75	#######	2.55273	0.981	0.555	#######	7	MTRNR2L12
76	#######	2.30343	0.761	0.231	#######	7	SOX4
77	#######	2.21718	0.978	0.253	#######	7	ANXA4
78	0	2.18354	0.955	0.167	0	7	TM4SF4
79	#######	2.15507	0.416	0.084	#######	7	CXCL1
80	0	2.15073	0.928	0.183	0	7	KRT8
81	0	5.44285	0.971	0.122	0	8	C1QB
82	0	5.29852	0.981	0.077	0	8	C1QC
83	0	5.29718	0.99	0.14	0	8	C1QA
84	#######	3.7506	0.722	0.222	#######	8	SEPP1
85	#######	3.45345	1	0.988	#######	8	FTL
86	#######	3.45344	0.823	0.198	#######	8	APOE
87	0	3.29908	0.969	0.09	0	8	MS4A7
88	#######	3.18173	0.981	0.363	#######	8	CTSB
89	#######	3.14261	0.995	0.549	#######	8	HLA-DRA
90	#######	3.09143	0.986	0.471	#######	8	HLA-DPA1
91	#######	3.36657	1	0.534	#######	9	HLA-DPB1
92	#######	3.24762	1	0.551	#######	9	HLA-DRA
93	#######	3.21756	1	0.473	#######	9	HLA-DPA1
94	0	3.1265	0.997	0.229	0	9	HLA-DQA1
95	#######	3.02343	0.992	0.349	#######	9	HLA-DQB1
96	#######	2.95341	0.997	0.536	#######	9	HLA-DRB1
97	#######	2.90274	0.997	0.392	#######	9	HLA-DRB5
98	#######	2.86302	1	0.541	#######	9	CST3
99	#######	2.71642	1	0.824	#######	9	CD74
100	#######	2.66951	0.975	0.258	#######	9	LYZ
101	0	4.56923	0.94	0.088	0	10	CLDN5
102	0	4.38365	1	0.172	0	10	IFI27
103	0	4.20809	0.943	0.137	0	10	SLC9A3R2
104	0	3.64493	0.974	0.113	0	10	RAMP2
105	#######	3.61896	0.992	0.226	#######	10	TM4SF1
106	0	3.28125	0.925	0.131	0	10	PLPP1
107	0	3.07641	0.842	0.056	0	10	CXCL12
108	#######	3.05525	0.774	0.115	#######	10	RBP7
109	#######	2.99241	0.992	0.239	#######	10	ID1
110	0	2.94874	0.966	0.164	0	10	A2M
111	0	5.72879	0.953	0.073	0	11	ACTA2
112	0	5.71616	0.958	0.067	0	11	TAGLN
113	0	4.98241	0.977	0.093	0	11	MYL9
114	0	4.79333	0.696	0.015	0	11	RGS5
115	#######	4.67445	0.939	0.138	#######	11	ADIRF
116	0	4.4117	0.944	0.062	0	11	TPM2
117	#######	4.25704	1	0.279	#######	11	IGFBP7
118	0	4.16165	0.813	0.017	0	11	NDUFA4L2

For Peer Review

119	0	3.73163	0.939	0.088	0	11	GPX3
120	#######	3.68583	0.981	0.217	#######	11	CALD1
121	#######	6.04085	0.917	0.27	#######	12	APOA1
122	2.00E-85	5.71456	0.779	0.243	4.17E-81	12	APOA2
123	#######	5.57287	0.98	0.232	#######	12	APOC1
124	#######	5.34156	0.539	0.091	#######	12	HP
125	#######	5.1175	0.706	0.103	#######	12	APOC3
126	#######	4.63911	0.995	0.324	#######	12	ALB
127	0	4.61746	0.784	0.055	0	12	ORM1
128	#######	4.3573	0.922	0.138	#######	12	TTR
129	0	4.34685	0.706	0.057	0	12	FGB
130	#######	4.32275	0.985	0.208	#######	12	APOE
131	2.08E-48	7.79951	0.968	0.476	4.33E-44	13	IGLC3
132	7.39E-39	7.74051	0.929	0.495	1.54E-34	13	IGLC2
133	#######	7.63999	0.812	0.185	4.97E-99	13	IGHG1
134	1.75E-77	7.58651	0.786	0.23	3.64E-73	13	IGHG3
135	#######	7.42265	0.688	0.075	#######	13	IGHGP
136	4.73E-51	7.26304	1	0.954	9.86E-47	13	IGKC
137	1.18E-39	7.19631	0.974	0.51	2.45E-35	13	IGHA1
138	3.66E-32	6.94784	0.805	0.323	7.62E-28	13	IGHM
139	#######	6.78103	0.623	0.061	#######	13	IGHG2
140	#######	6.75811	0.961	0.212	#######	13	JCHAIN
141	7.07E-99	3.52877	0.982	0.291	1.47E-94	14	STMN1
142	1.03E-73	3.3998	0.974	0.447	2.15E-69	14	HMGB2
143	2.30E-56	2.7811	1	0.769	4.79E-52	14	TUBA1B
144	2.02E-25	2.75531	0.754	0.421	4.21E-21	14	HIST1H4C
145	2.18E-43	2.59685	0.912	0.6	4.53E-39	14	TUBB
146	0	2.55409	0.833	0.031	0	14	KIAA0101
147	4.32E-66	2.51651	1	0.678	9.00E-62	14	HMGN2
148	0	2.41773	0.614	0.003	0	14	UBE2C
149	0	2.40456	0.772	0.011	0	14	TYMS
150	7.03E-61	2.23944	0.982	0.677	1.46E-56	14	H2AFZ
151	0	5.59824	1	0.027	0	15	DCN
152	0	5.41154	0.947	0.021	0	15	COL1A1
153	0	4.73316	0.947	0.021	0	15	COL3A1
154	0	4.47163	0.968	0.015	0	15	COL1A2
155	1.58E-66	4.3283	1	0.477	3.28E-62	15	TIMP1
156	4.58E-90	4.31834	1	0.287	9.53E-86	15	IGFBP7
157	0	4.25718	0.947	0.005	0	15	LUM
158	0	4.04396	0.979	0.042	0	15	BGN
159	0	3.97383	0.853	0.025	0	15	COLEC11
160	#######	3.8744	0.989	0.081	#######	15	C1R
161	2.82E-64	4.14373	0.381	0.035	5.87E-60	16	PTGDS
162	0	3.72268	0.643	0.019	0	16	TCL1A
163	#######	3.59054	0.988	0.112	#######	16	GZMB
164	#######	3.55677	0.976	0.118	#######	16	IRF8
165	#######	3.52722	0.976	0.156	#######	16	IRF7
166	0	3.39481	0.952	0.009	0	16	LILRA4
167	0	3.37942	1	0.048	0	16	PLD4
168	#######	3.01479	0.976	0.183	#######	16	ITM2C
169	#######	2.91341	0.976	0.188	#######	16	CCDC50
170	3.00E-99	2.70331	1	0.217	6.24E-95	16	JCHAIN
171	#######	8.91917	0.8	0.035	#######	17	TPSB2
172	#######	8.69752	0.8	0.02	#######	17	TPSAB1
173	#######	4.60546	0.514	0.005	#######	17	CTSG
174	0	4.29931	0.714	0.001	0	17	TPSD1
175	0	4.22054	0.686	0.002	0	17	CPA3
176	0	4.02792	0.829	0.007	0	17	HPGDS
177	1.48E-20	3.53364	0.686	0.165	3.08E-16	17	AREG
178	#######	3.47117	0.714	0.016	#######	17	HPGD

For Peer Review

179	5.06E-91	3.35491	0.8	0.053	1.05E-86	17	C1orf186
180	#######	2.95183	0.743	0.028	#######	17	VWA5A

For Peer Review

TABLE S3 Cell type annotation

Legend:
Previous experience in cell type recognition and the ACT website annotation were used for marker identification.

Table Structure:
- Sheets "_anno": Annotate the cells based on Top 10 upregulated genes across each cell cluster.

For Peer Review

Table S3a. Cell type annotation of liver cirrhosis patients and healthy individuals

cluster				TOP10				
cluster_0	IL7R	LTB	TRAC	CD3D	CD3E	CXCR4	CD2	TRBC2
cluster_1	GNLY	CCL3	XCL1	NKG7	XCL2	CMC1	KLRF1	KLRD1
cluster_2	CCL5	CD8A	GZMH	CD3D	CD8B	GZMK	TRAC	CD3E
cluster_3	MGP	SPARCL1	VWF	GNG11	FABP4	PLVAP	IGFBP7	ACKR1
cluster_4	S100A8	S100A9	LYZ	RP11-114	FCN1	S100A12	VCAN	LST1
cluster_5	C1QC	C1QA	C1QB	HLA-DRA	HLA-DPA	HLA-DRB	HLA-DPB	HLA-DRB
cluster_6	CD79A	MS4A1	CD79B	TCL1A	CD37	VPREB3	IGHD	CD74
cluster_7	C1QB	C1QA	C1QC	CD5L	MARCO	APOE	SEPP1	SLC40A1
cluster_8	TAGLN	ACTA2	MYL9	RGS5	ADIRF	TPM2	CALD1	MYH11
cluster_9	HLA-DPB	HLA-DQA	HLA-DQB	HLA-DPA	HLA-DRA	HLA-DRB	FCER1A	CD74
cluster_10	TM4SF4	ANXA4	DEFB1	FXYD2	KRT8	ALB	KRT18	AMBP
cluster_11	FCN3	CRHBP	CLEC4G	DNASE1L	FCN2	CLEC1B	CTSL	CLEC4M
cluster_12	DEFB1	FXYD2	ANXA4	TM4SF4	KRT18	KRT8	ALB	SERPINA
cluster_13	SLC9A3R	CLDN5	IFI27	RAMP2	ID1	TM4SF1	TIMP3	PLPP1
cluster_14	IGLC3	IGLC2	IGKC	IGHA1	IGHG1	IGHG3	JCHAIN	IGHA2
cluster_15	STMN1	HMGB2	HIST1H4C	TUBA1B	TUBB	TYMS	KIAA0101	UBE2C
cluster_16	DCN	COL1A1	COL3A1	TIMP1	BGN	COL1A2	LUM	PTGDS
cluster_17	APOA1	APOA2	APOC3	APOC1	ALB	HP	ORM1	TTR
cluster_18	GZMB	PTGDS	IRF7	PLD4	LILRA4	ITM2C	IRF8	CCDC50
cluster_19	TPSB2	TPSAB1	CTSG	CPA3	HPGDS	AREG	TPSD1	HPGD
cluster_20	TUBA1B	STMN1	HMGB2	CST3	HIST1H4C	KIAA0101	TUBB	HMGN2
cluster_21	C1QA	C1QB	C1QC	FTL	SLC40A1	MS4A6A	MS4A7	APOE

[1] Wang J, Du M, Meng L, et al. Integrative analysis implicates the significance of m6A in the liver fibr
[2] Huang X, Zhang L, Luo W, et al. Endothelial anthrax toxin receptor 2 plays a protective role in liver f
[3] MacParland SA, Liu JC, Ma XZ, et al. Single cell RNA sequencing of human liver reveals distinct int
[4] Wang ZY, Keogh A, Waldt A, et al. Single-cell and bulk transcriptomics of the liver reveals potential
[5] Ramachandran P, Dobie R, Wilson-Kanamori JR, et al. Resolving the fibrotic niche of human liver ci

For Peer Review

		Article 1[1	Article 2[2	Article 3[3	Article 4[4	Article 5[5	cell_type
IL32	CD52	T					T/NK
CCL4	CD7	NK					T/NK
IL32	TRBC2	T(CD3D)	T(CD3D，CD8A)				T/NK
ID1	IGFBP4	pLSEC(MGP,SPAR(		LSEC(MG	LSEC(SPARCL1)		LSEC
AIF1	CSTA	monocyte					monocyte
FTL	HLA-DQA	macrophage cell					macrophage cell
LINC0092	BANK1	naive B					B
CD163	MS4A7	macrophage cell					macrophage cell
NDUFA4L	SOD3	HSC					HSC
CST3	HLA-DRB	macrophage cell					macrophage cell
SERPINA	KRT7	Cholangiocyte					Cholangiocyte
AKAP12	PLPP3	cLSEC					LSEC
KRT7	CLU	Cholangiocyte					Cholangiocyte
CXCL12	RBP7	pLSEC					LSEC
IGHM	IGHGP	plasma					plasma
HMGN2	H2AFZ	NKT					T/NK
C1R	CCDC80	Myofibroblast					Myofibroblast
RBP4	FGB	Hepatocyte					Hepatocyte
TCL1A	PPP1R14	pDC					DC
MS4A2	C1orf186					mast	mast
HLA-DPA	HLA-DRA	NKT					T/NK
CTSB	HLA-DRB	macrophage cell					macrophage cell

osis of biliary atresia by regulating THY1. *Hepatology Communications*. 2023;7(1):e0004. doi:10.1097/H
fibrosis. *Frontiers in Cell and Developmental Biology*. 2024;11:1278968. doi:10.3389/fcell.2023.1278968
trahepatic macrophage populations. *Nature communications*. 2018;9(1):4383. doi:10.1038/s41467-018-06
targets of NASH with fibrosis. *Scientific reports*. 2021;11(1):19396. doi:10.1038/s41598-021-98806-y
rrhosis at single-cell level. *Nature*. 2019;575(7783):512-518. doi:10.1038/s41586-019-1631-3

IC9.0000000000000004
3
5318-7

For Peer Review

Table S3b. Cell type annotation of liver cirrhosis patients

Cluster	TOP10							
cluster_0	IL7R	LTB	TRAC	CXCR4	CD69	CD3E	CD3D	ZFP36L2
cluster_1	CCL5	CD8A	IFNG	TRAC	CD3D	RGS1	CD2	GZMH
cluster_2	GNLY	CCL3	NKG7	CMC1	GZMB	CCL4	KLRF1	KLRD1
cluster_3	ANXA4	TM4SF4	KRT8	SPP1	KRT18	DEFB1	FXYD2	AMBP
cluster_4	DNASE1L	CCL21	FABP4	GNG11	CCL14	SPARCL1	MGP	VWF
cluster_5	S100A9	S100A8	FCN1	LYZ	AIF1	LST1	RP11-114	CSTA
cluster_6	CD79A	MS4A1	CD79B	VPREB3	CD37	CD74	CD83	BANK1
cluster_7	DEFB1	ELF3	FXYD2	MTRNR2L	MTRNR2L	SOX4	ANXA4	TM4SF4
cluster_8	C1QB	C1QC	C1QA	SEPP1	FTL	APOE	MS4A7	CTSB
cluster_9	HLA-DPB	HLA-DRA	HLA-DPA	HLA-DQA	HLA-DQB	HLA-DRB	HLA-DRB	CST3
cluster_10	CLDN5	IFI27	SLC9A3R	RAMP2	TM4SF1	PLPP1	CXCL12	RBP7
cluster_11	ACTA2	TAGLN	MYL9	RGS5	ADIRF	TPM2	IGFBP7	NDUFA4L
cluster_12	APOA1	APOA2	APOC1	HP	APOC3	ALB	ORM1	TTR
cluster_13	IGLC3	IGLC2	IGHG1	IGHG3	IGHGP	IGKC	IGHA1	IGHM
cluster_14	STMN1	HMGB2	TUBA1B	HIST1H4C	TUBB	KIAA0101	HMGN2	UBE2C
cluster_15	DCN	COL1A1	COL3A1	COL1A2	TIMP1	IGFBP7	LUM	BGN
cluster_16	PTGDS	TCL1A	GZMB	IRF8	IRF7	LILRA4	PLD4	ITM2C
cluster_17	TPSB2	TPSAB1	CTSG	TPSD1	CPA3	HPGDS	AREG	HPGD

[1] Wang J, Du M, Meng L, et al. Integrative analysis implicates the significance of m6A in the liver fibro
[2] Huang X, Zhang L, Luo W, et al. Endothelial anthrax toxin receptor 2 plays a protective role in liver f
[3] MacParland SA, Liu JC, Ma XZ, et al. Single cell RNA sequencing of human liver reveals distinct int
[4] Wang ZY, Keogh A, Waldt A, et al. Single-cell and bulk transcriptomics of the liver reveals potential

For Peer Review

		Article 1[1]	Article 2[2]	Article 3[3]	Article 4[4]	cell_type
TSC22D3	CD52	T				T/NK
IL32	TRBC2	T(TRAC)	T			T/NK
FGFBP2	XCL2	NK				T/NK
KRT7	GC	Cholangiocyte				Cholangiocyte
ID1	TFF3	LSEC(DNASE1L3)				LSEC
S100A12	CTSS	Monocyte				Monocyte
IGHD	HLA-DRA	Naive B				B
CXCL1	KRT8	Cholangiocyte(DEFB1)				Cholangiocyte
HLA-DRA	HLA-DPA1	Macrophage				Macrophage
CD74	LYZ	Macrophage				Macrophage
ID1	A2M	LSEC		LSEC		LSEC
GPX3	CALD1	HSC				HSC
FGB	APOE	Hepatocyte				Hepatocyte
IGHG2	JCHAIN	Plasma				B
TYMS	H2AFZ	NKT				T/NK
COLEC11	C1R	Myofibroblast				Myofibroblast
CCDC50	JCHAIN	pDC			DC	DC
C1orf186	VWA5A	Mast				Mast

osis of biliary atresia by regulating THY1. *Hepatology Communications*. 2023;7(1):e0004. doi:10.1097/H
fibrosis. *Frontiers in Cell and Developmental Biology*. 2024;11:1278968. doi:10.3389/fcell.2023.1278968
trahepatic macrophage populations. *Nature communications*. 2018;9(1):4383. doi:10.1038/s41467-018-06
targets of NASH with fibrosis. *Scientific reports*. 2021;11(1):19396. doi:10.1038/s41598-021-98806-y

For Peer Review

C9.0000000000000004
3
318-7

For Peer Review

TABLE S4 siRNA predicted sequence of T

target position
231-253

For Peer Review

THY1

target sequence 21nt target + 2nt overhang
aaccaacttcaccagcaaatacа

For Peer Review

RNA oligo sequences 21nt guide (5'→3') 21nt passenger (5'→3')
UAUUUGCUGGUGAAGUUGGUU CCAACUUCACCAGCAAAUACA

For Peer Review

TABLE S5 siRNA predicted sequence of SMOC2

target position	target sequence 21nt target + 2nt overhang
803-825	AACAAAACCAATAAGAATTCAGT
1105-1127	TGCCAAAAAGCATGAGTTTCTGA
1250-1272	TACTTCAAACTACTGGATAAAAA
1296-1318	AGGAAATCAAACCCTTCAAGAGG
1350-1372	GTGTGAAGAAGTTTGTTGAATAC
1355-1377	AAGAAGTTTGTTGAATACTGTGA
1365-1387	TTGAATACTGTGACGTGAATAAT
1377-1399	ACGTGAATAATGACAAATCCATC
1485-1507	ATGCTGAAAGTACGTCTAATAGA
1510-1532	GCCAAGGAAACAAGGATAAATGG
1577-1599	CACCAAAGAGCAATTAAGAAAAC
1605-1627	CAGAAACACATAGTATTTGCACT
1611-1633	CACATAGTATTTGCACTTTGTAC
1615-1637	TAGTATTTGCACTTTGTACTTTA
1622-1644	TACTTCAAACTACTGGATAAAAA
1624-1646	CACTTTGTACTTTAAATGTAAAT
1652-1674	TTGTAGAAATGAGCTATTTAAAC
1655-1677	TAGAAATGAGCTATTTAAACAGA
1676-1698	GACTGTTTTAATCTGTGAAAATG
1706-1728	GGCTTCAGAAAATTAATCACATA
1740-1762	TCCTCTTTTGACCTTGGAAATCT
1773-1795	GAGAAGTATTTGAATGCATTTAG
1786-1808	ATGCATTTAGGCTTAATTTCTTC
1787-1809	TGCATTTAGGCTTAATTTCTTCG
1849-1871	TGCAATCGTATGGCTTTCTCTAA
1997-2019	TTGTTTCTTGGGATTTTCTGTTA
2005-2027	TGGGATTTTCTGTTAGTTTGTCT
2040-2062	CAGAGATCTTGCTCATACAATGA
2109-2131	TGGAGGAAATAATATTTTCAAAC
2112-2134	AGGAAATAATATTTTCAAACTGT
2162-2184	ATCTTTGCTTTTCCATTTTAAGC
2172-2194	TTCCATTTTAAGCTTCTGTTTTG
2173-2195	TCCATTTTAAGCTTCTGTTTTGA
2722-2744	CAGTGTCTTAAGGAACCATTTGG
2794-2816	GACTTTTGGTTGGAAAAAGATCT
2966-2988	GCCAATGTTGTTTAAGAAACAGT
2973-2995	TTGTTTAAGAAACAGTTATGATC
2983-3005	AACAGTTATGATCCTAAACTTTT
2985-3007	CAGTTATGATCCTAAACTTTTTG
2993-3015	ATCCTAAACTTTTTGGATAATCT
3028-3050	ACCTTTGAATTTAATCATTGTTC
3032-3054	TTGAATTTAATCATTGTTCTTAG
3041-3063	ATCATTGTTCTTAGATTAAAATA
3052-3074	TAGATTAAAATAAAATATGCTAT

For Peer Review

RNA oligo sequences 21nt guide (5’→3’) 21nt passenger (5’→3’)
UGAAUUCUUAUUGGUUUUGUU CAAAACCAAUAAGAAUUCAGU
AGAAACUCAUGCUUUUUGGCA CCAAAAAGCAUGAGUUUCUGA
UUUAUCCAGUAGUUUGAAGUA CUUCAAACUACUGGAUAAAAA
UCUUGAAGGGUUUGAUUUCCU GAAAUCAAACCCUUCAAGAGG
AUUCAACAAACUUCUUCACAC GUGAAGAAGUUUGUUGAAUAC
ACAGUAUUCAACAAACUUCUU GAAGUUUGUUGAAUACUGUGA
UAUUCACGUCACAGUAUUCAA GAAUACUGUGACGUGAAUAAU
UGGAUUUGUCAUUAUUCACGU GUGAAUAAUGACAAAUCCAUC
UAUUAGACGUACUUUCAGCAU GCUGAAAGUACGUCUAAUAGA
AUUUAUCCUUGUUUCCUUGGC CAAGGAAACAAGGAUAAAUGG
UUUCUUAAUUGCUCUUUGGUG CCAAAGAGCAAUUAAGAAAAC
UGCAAAUACUAUGUGUUUCUG GAAACACAUAGUAUUUGCACU
ACAAAGUGCAAAUACUAUGUG CAUAGUAUUUGCACUUUGUAC
AAGUACAAAGUGCAAAUACUA GUAUUUGCACUUUGUACUUUA
ACAUUUAAAGUACAAAGUGCA CACUUUGUACUUUAAAUGUAA
UUACAUUUAAAGUACAAAGUG CUUUGUACUUUAAAUGUAAAU
UUAAAUAGCUCAUUUCUACAA GUAGAAAUGAGCUAUUUAAAC
UGUUUAAAUAGCUCAUUUCUA GAAAUGAGCUAUUUAAACAGA
UUUUCACAGAUUAAAACAGUC CUGUUUUAAUCUGUGAAAAUG
UGUGAUUAAUUUUCUGAAGCC CUUCAGAAAAUUAAUCACAUA
AUUUCCAAGGUCAAAAGAGGA CUCUUUUGACCUUGGAAAUCU
AAAUGCAUUCAAAUACUUCUC GAAGUAUUUGAAUGCAUUUAG
AGAAAUUAAGCCUAAAUGCAU GCAUUUAGGCUUAAUUUCUUC
AAGAAAUUAAGCCUAAAUGCA CAUUUAGGCUUAAUUUCUUCG
AGAGAAAGCCAUACGAUUGCA CAAUCGUAUGGCUUUCUCUAA
ACAGAAAAUCCCAAGAAACAA GUUUCUUGGGAUUUUCUGUUA
ACAAACUAACAGAAAAUCCCA GGAUUUUCUGUUAGUUUGUCU
AUUGUAUGAGCAAGAUCUCUG GAGAUCUUGCUCAUACAAUGA
UUGAAAAUAUUAUUUCCUCCA GAGGAAAUAAUAUUUUCAAAC
AGUUUGAAAAUAUUAUUUCCU GAAAUAAUAUUUUCAAACUGU
UUAAAAUGGAAAAGCAAAGAU CUUUGCUUUUCCAUUUUAAGC
AAACAGAAGCUUAAAAUGGAA CCAUUUUAAGCUUCUGUUUUG
AAAACAGAAGCUUAAAAUGGA CAUUUUAAGCUUCUGUUUUGA
AAAUGGUUCCUUAAGACACUG GUGUCUUAAGGAACCAUUUGG
AUCUUUUUCCAACCAAAAGUC CUUUUGGUUGGAAAAAGAUCU
UGUUUCUUAAACAACAUUGGC CAAUGUUGUUUAAGAAACAGU
UCAUAACUGUUUCUUAAACAA GUUUAAGAAACAGUUAUGAUC
AAGUUUAGGAUCAUAACUGUU CAGUUAUGAUCCUAAACUUUU
AAAAAGUUUAGGAUCAUAACUG
AUUAUCCAAAAAGUUUAGGAU CCUAAACUUUUUGGAUAAUCU
ACAAUGAUUAAAUUCAAAGGU CUUUGAAUUUAAUCAUUGUUC
AAGAACAAUGAUUAAAUUCAA GAAUUUAAUCAUUGUUCUUAG
UUUUAAUCUAAGAACAAUGAU CAUUGUUCUUAGAUUAAAAUA
AGCAUAUUUUAUUUUAAUCUA GAUUAAAAUAAAAUAUGCUAU

For Peer Review
